# New Insights into the French Paradox: Free Radical Scavenging by Resveratrol Yields Cardiovascular Protective Metabolites

**DOI:** 10.1021/acs.jmedchem.4c03061

**Published:** 2025-05-07

**Authors:** Orinamhe G. Agbadua, Norbert Kúsz, Róbert Berkecz, Elemér Vass, Antal Csámpai, Gábor Tóth, György T. Balogh, Laurence Marcourt, Jean-Luc Wolfender, Emerson Ferreira Queiroz, Attila Hunyadi

**Affiliations:** † Institute of Pharmacognosy, University of Szeged, Eötvös str. 6, H-6720 Szeged, Hungary; ‡ Institute of Pharmaceutical Analysis, University of Szeged, Somogyi str. 4, H-6720 Szeged, Hungary; § Department of Organic Chemistry, Eötvös Loránd University, Pázmány Péter sétány 1/a, H-1117 Budapest, Hungary; ∥ NMR Group, Department of Inorganic and Analytical Chemistry, Budapest University of Technology and Economics, H-1111 Budapest, Hungary; ⊥ Department of Pharmaceutical Chemistry, Semmelweis University, H-1092 Budapest, Hungary; # Center for Pharmacology and Drug Research & Development, Semmelweis University, H-1085 Budapest, Hungary; ∇ Department of Chemical and Environmental Process Engineering, Budapest University of Technology and Economics, H-1111 Budapest, Hungary; □ School of Pharmaceutical Sciences, University of Geneva, CMU, 1211 Geneva, Switzerland; ○ Institute of Pharmaceutical Sciences of Western Switzerland, University of Geneva, CMU, 1211 Geneva, Switzerland; ◆ HUN-REN-SZTE Biologically Active Natural Products Research Group, Eötvös str. 6, H-6720 Szeged, Hungary; ¶ Graduate Institute of Natural Products, Shih-Chuan first Rd. 100, Kaohsiung 807, Taiwan

## Abstract

Resveratrol was subjected to a diversity-oriented synthesis using oxidative transformations by various biorelevant, biomimetic, or biomimetic-related chemical reagents. Using a combined strategy of ultrahigh-resolution profiling, bioactivity screening, and bioactivity-guided isolation, 19 metabolites were obtained. The compounds were tested for their *in vitro* enzyme inhibitory activity on angiotensin-1 converting enzyme (ACE), cyclooxygenase-1 and -2, and 15-lipoxygenase (LOX), and evaluated for their relevant drug-like properties *in silico*. The compounds demonstrated a generally increased cardiovascular protective and anti-inflammatory potential and better drug-likeness compared to resveratrol. *Trans*-δ-viniferin (**6**) was identified as a competitive, C-domain-selective ACE inhibitor that is over 20 times more potent than resveratrol. Further, *trans*-ε-viniferin (**2**) acted as an over 40 times stronger LOX inhibitor than resveratrol. While our results cannot be directly translated to the health benefits of dietary resveratrol consumption without further studies, it is demonstrated that biologically relevant oxidative environments transform resveratrol into potent cardiovascular protective and anti-inflammatory metabolites.

## Graphical abstract

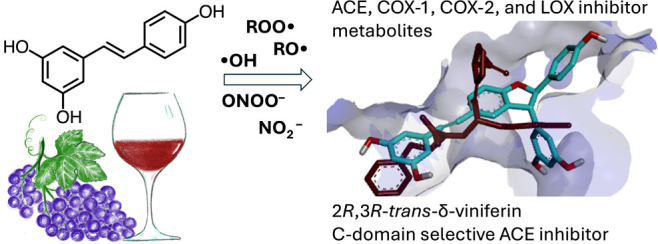

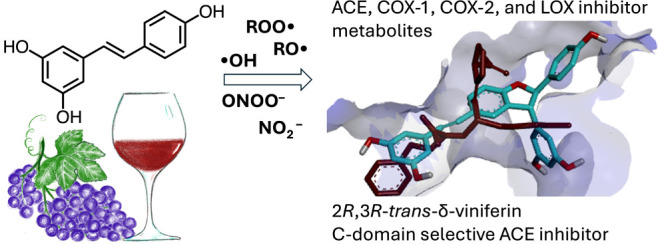

## 1 Introduction

The “French paradox” concerns the relatively low incidence of coronary heart disease mortality in the French population despite the high intake of cholesterol and saturated fat, and it is attributed to a moderate but regular consumption of red wine.^1^ Vast studies on this phenomenon show that low-to-moderate consumption of alcohol, particularly red wine, has a protective effect against coronary heart disease.^2,3^ These beneficial effects of red wine are due to the variety of polyphenols, predominantly resveratrol, present in grape skins.^3^ Resveratrol is also found in many other common foods, including a variety of fruits, peanuts, pistachios, and cocoa, and is widely acknowledged for its potential health benefits, particularly in the context of cardiovascular health.^4–7^

Regarding the development and progression of cardiovascular diseases (CVDs), the angiotensin-converting enzyme (ACE), an important component of the renin-angiotensin system (RAS), plays a critical role.^5^ Angiotensin II, formed by the action of ACE, increases the vascular activity of NADPH oxidase in the cardiovascular system, thus enhancing the development and progression of CVDs through increasing ROS levels.^8^ Angiotensin II-mediated oxidative stress initiates various redox signaling cascades,^8,9^ uncouples nitric oxide synthase (NOS),^10^ increases blood pressure,^11^ and activates several inflammatory mediators.^12^ Given the relevance and interdependence of ACE with oxidative stress and CVDs, it is not surprising that ACE inhibitors have a high polypharmacological potential. The cardioprotective effects of resveratrol have been associated with ACE inhibition,^5^ modulation of many signaling pathways regulating endothelial nitric oxide production,^13^ reduction of oxidative stress,^14^ inhibition of vascular inflammation,^15^ and prevention of platelet aggregation.^16,17^

As a well-known antioxidant, resveratrol neutralizes reactive oxygen species (ROS) and reactive nitrogen species (RNS), leading to improved cardiovascular function.^18^ ROS include molecules such as superoxide anion radical (O_2_^•–^), hydrogen peroxide (H_2_O_2_), and hydroxyl radical (^•^OH),^19–21^ while RNS primarily include nitric oxide (NO) and peroxynitrite (ONOO^–^).^22^ These species are produced endogenously as byproducts of normal cellular metabolism, particularly during mitochondrial respiration and through the activity of various oxidative enzymes such as NADPH oxidase and nitric oxide synthase.^23^ While ROS and RNS are essential for physiological processes such as immune defense, cell signaling, and vascular regulation, their overproduction or dysregulation can lead to oxidative and nitrative stress, implicated in the pathogenesis of a wide range of diseases, including cardiovascular disorders.^24^

Like most polyphenols, resveratrol exerts its antioxidant activity primarily through enzymatic pathways that regulate cellular redox balance.^25^ However, its direct free radical scavenging capacities, for example, toward superoxide anion radicals, may also be important in view of the cardiovascular system.^18^ In our previous reviews, we demonstrated that such free radical scavenging events by an antioxidant can lead to the formation of many chemically stable bioactive oxidized metabolites depending on the type of reactive oxygen or nitrogen species (ROS/RNS) scavenged.^26,27^ Our “scaveng­(e)­ome” concept was introduced to describe the chemical space of all the metabolites that can be formed from an antioxidant by scavenging ROS/RNS.^26^ We postulated that high biological performance diversity, that is, a key objective of modern diversity-oriented synthesis,^28^ may be achieved by ROS/RNS-mediated oxidative transformations of small-molecule antioxidants. This concept was successfully applied to the transformation of hydroxycinnamic acids to obtain antitumor leads,^29,30^ and resveratrol to obtain potent xanthine oxidase inhibitors.^31^

In the current study, our aim was to explore the scavengeome of resveratrol for potential cardiovascular protective metabolites, focusing primarily on ACE inhibition. Due to the interdependence of ACE with ROS and inflammation, the anti-inflammatory activities of these oxidized metabolites were also determined, evaluating the inhibitory potential of the compounds on enzymes such as 15-lipoxygenase and cyclooxygenase-1 and -2 (COX-1 & COX-2).

## 2 Results and Discussion

### 2.1 Preparation and Evaluation of Oxidized Resveratrol Mixtures

Oxidized resveratrol metabolite mixtures were prepared by subjecting resveratrol to various oxidative reactions, under several biomimetic and biorelevant oxidation models based on similarities with human physiological processes.^27^ Resveratrol was therefore subjected to described chemical models^27^ that directly provide oxidative agents/free radicals present in the body (^•^OH generated by metalloporphyrin/H_2_O_2_, Fe^2+^, or Cu^2+^, and ONOO^–^) and those with substantial experimental evidence of their suitability to model biological oxidative stress (AIBN and AAPH, which decompose to generate alkylperoxyl and alkoxyl radicals, respectively).^32,33^ In biological systems, peroxynitrite is rapidly converted to peroxynitrous acid (ONOOH), a strong oxidizing agent.^34^ Under acidic conditions present in the stomach, nitrite acts as an oxidizing agent,^35^ thus providing biomimetic evidence for the transformations of resveratrol with sodium nitrite under acid conditions, phosphate buffer (pH 3.0)^36^ and KCl-HCl (pH 2.0).^35^ Lastly, xanthine oxidase, an ubiquitous enzyme in living organisms, catalyzes xanthine and hypoxanthine oxidation to uric acid, ultimately generating ROS (O_2_^•–^ and H_2_O_2_).^37^ Chemical systems in which the oxidative reaction medium contained an aqueous component were also considered. As in our previous work,^31^ most reactions were terminated by adding reduced glutathione (GSH), an abundant intracellular antioxidant^38^ to further improve the biorelevance of the experimental procedure.

The resulting oxidized mixtures (Ox1–Ox16) were analyzed by HPLC-PDA for their chromatographic fingerprints (Supporting Information, Figures S1 and S8) and UHPLC-PDA-ELSD-MS (Supporting Information, Figures S9 and S24) to obtain a detailed overview of their metabolic profile. The mixtures were analyzed against a diverse array of resveratrol oligomers to obtain preliminary information on the oxidized metabolites formed. At first glance using Mzmine 4.3.0 (mzio, GmbH) software and following previously published protocol,^39^ the metabolite map of the oxidized mixtures, Ox1–Ox16, showed that oxidized metabolites ranged between 220–570 Da (3-D metabolic profile chart of Ox1–Ox16, Supporting Information, Figure S25), with a large number of metabolites having *m*/*z* values signifying resveratrol dimers, and ethoxy- or halogen-substituted derivatives.

Chemical libraries that are not only diverse but also have a high pharmacological hit rate are deemed important.^40,41^ Therefore, these chemically diverse oxidized mixtures were subjected to ACE and LOX inhibitory assays, which are highly relevant in view of the well-known bioactivities of resveratrol. The oxidation of resveratrol resulted in several metabolites with modulated ACE and LOX inhibitory activity when compared to the parent compound, as seen in Table 1. The observed improved LOX inhibition corroborated a study by Shingai et al.,^42^ who reported that biomimetic, Fe-catalyzed oxidation produced a mixture that exhibited potent LOX inhibitory activity, in contrast to the inactive resveratrol itself. The combination of the metabolite diversity of these mixtures and their bioactivity served as a guide for the isolation of most bioactive metabolites. Bioactivity results, along with compounds isolated from each mixture, are compiled in Table 1.

**Table 1:** ACE and 15-LOX Inhibitory Activities of the Oxidized Mixtures in Comparison with Resveratrol and the Compounds Isolated from Each Mixture^a^

ID	ACE Inh. (%)	LOX Inh. (%)	compound(s) isolated
Res.	35.9 ± 1.9	7.0 ± 1.0	**-**
Ox1	51.3 ± 1.2*	45.9 ± 9.1*	**1–3**
Ox2	69.9 ± 7.2*	43.8 ± 3.3*	**4–7**
Ox3	55.3 ± 3.3*	56.8 ± 7.0*	**6**
Ox4	66.7 ± 6.3*	39.1 ± 4.0*	**8–10**
Ox5	87.3 ± 1.6*	42.1 ± 3.8*	**5, 8, 11–15**
Ox6	87.7 ± 4.5*	18.9 ± 4.5	**5, 12, 16**
Ox7	64.7 ± 3.5*	2.9 ± 1.4	**5, 13, 14, 17**
Ox8	52.0 ± 7.2*	20.5 ± 3.6	**6**
Ox9	94.3 ± 1.9*	31.7 ± 3.8*	**2, 6, 19**
Ox10	75.4 ± 3.4*	30.4 ± 5.6*	**4, 18, 19**
Ox11	68.9 ± 0.9*	17.0 ± 1.8	**19**
Ox12	48.2 ± 2.3	44.0 ± 3.8*	**2**
Ox13	64.8 ± 4.0*	34.2 ± 2.5*	**2, 6**
Ox14	86.1 ± 1.2*	29.6 ± 5.1*	**6**
Ox15	59.2 ± 1.7*	12.7 ± 1.0	**4, 6**
Ox16	47.6 ± 4.8	18.5 ± 3.8	**2**

a Results are expressed as mean ± standard error of the mean (SEM), *n* = 3, *: *p* < 0.05 by one-way analysis of variance (ANOVA) using Dunnett’s multiple comparison test to the parent compound, resveratrol. Resveratrol was tested at 90 and 40 μM for ACE and 15-LOX inhibition screening respectively, and mixtures Ox1–Ox16 tested at corresponding concentrations in resveratrol equivalents.

### 2.2 Structure Elucidation

The ^1^H NMR spectrum of our starting material, *trans*-resveratrol (C_14_H_12_O_3_) consists of one set of three aromatic hydrogens coupled in an AX_2_ system, another set of four aromatic hydrogens coupled in an AA’XX’ system, and two doublet signals indicating the presence of a HC=CH double bond with *trans*-configuration.^43^ The structures of compounds **2**, **5**, **6**, **8**–**10**, **12**, and **16** have been elucidated and reported in our previous study.^31^ Compounds **2** and **6** are *trans*-ε-viniferin and *trans*-δ-viniferin respectively, **5** and **12** are iodine-substituted derivatives, and **16** is 2-chlororesveratrol. Compounds **8**–**10** are ethoxy-substituted compounds; with **9** and **10** being dimers of resveratrol (as seen in Figure 1). The high-resolution mass spectrometry (HRMS) and NMR spectra of **4** (see Supporting Information, Figures S39–S41) were in good agreement with values reported in the literature for 3β-(3′,5′-dihydroxyphenyl)-2α-(4″-hydroxyphenyl)­dihydrobenzofuran-5-carbaldehyde.^36,44^ Furthermore, based on the HRMS and NMR spectra (Supporting Information, Figure S32 for HRMS, and Figures S58–S63 for NMR), the structures of compounds 1**8** and **19** were in good agreement with those previously reported by Panzella et al.^36^ for regioisomer nitro-derivatives.

**Figure 1:**
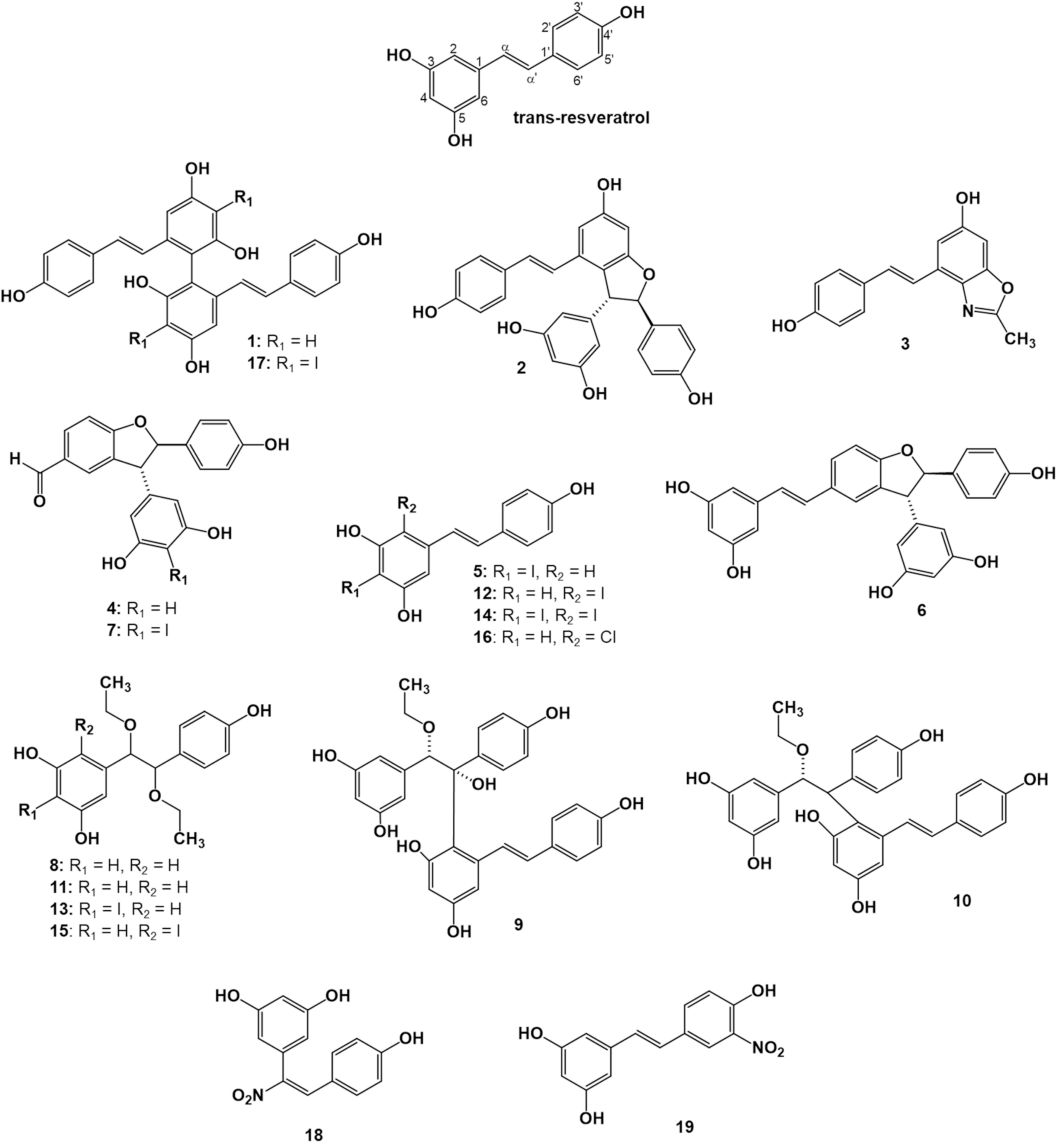
Structures of resveratrol and its ROS/RNS-oxidized metabolites (**1**–**19**).

The molecular formula of **1** was established as C_28_H_22_O_6_ based on its protonated molecular ion peak in the HRMS spectrum ([M + H]^+^, calculated: 455.14891, found 455.14919) (Supporting Information, Figure S26), which implied that compound **1** is a resveratrol dimer. The ^1^H NMR spectrum of **1** (Supporting Information, Figure S33) showed the presence of four aromatic hydrogens coupled in an AA’XX’ system (δ_H_ 7.21 d, 2H, *J* = 8.7 Hz, and δ_H_ 6.73 d, 2H, *J* = 8.7 Hz) corresponding to 4-hydroxyphenyl moiety, the signals of a *trans* double bond (δ_H_ 6.61 d, 1H, *J* = 16.3 Hz, and δ_H_ 6.91 d, 1H, *J* = 16.3 Hz) and two *meta*-coupled aromatic protons as well (δ_H_ 6.43 d, 1H, *J* = 2.2 Hz, δ_H_ 6.86 d, 1H, *J* = 2.2 Hz). The lack of the third hydrogen signal of the 3,5-dihydroxyphenyl ring of resveratrol, together with the appearance of a nonprotonated aromatic carbon resonating at δ_C_ 114.0, corroborated the above findings. This carbon atom also gave diagnostic HMBC correlations with the *meta*-coupled aromatic protons (Supporting Information, Figure S34). Considering that only one set of hydrogens was seen in the ^1^H NMR spectrum, it was concluded that compound **1** is a symmetrical dimer of resveratrol, in which the monomers are attached through C-2 of their dihydroxyphenyl moieties.

Based on the HRMS data ([M + H]^+^, calculated: 268.09682, found 268.09715), an elemental composition of C_16_H_13_NO_3_ (Supporting Information, Figure S26) was established for compound **3**. The ^1^H NMR spectrum of **3** (Supporting Information, Figure S36) consisted of the resonances characteristic to a 4-hydroxyphenyl ring (δ_H_ 7.49 d, 2H, *J* = 8.7 Hz, and δ_H_ 6.88 d, 2H, *J* = 8.7 Hz), a *trans* double bond (δ_H_ 7.26 d, 1H, *J* = 16.5 Hz, and δ_H_ 7.79 d, 1H, *J* = 16.5 Hz), and a pair of *meta*-coupled aromatic hydrogens (δ_H_ 6.86 d, 1H, *J* = 2.2 Hz, and δ_H_ 6.99 d, 1H, *J* = 2.2 Hz). Moreover, an additional deshielded methyl group was also identified at δ_H_ = 2.58 s (3H). The olefinic proton at δ_H_ 7.26 and the aromatic hydrogens of the 3,5-dihydroxyphenyl ring gave heteronuclear correlations with the markedly downfield shifted nonprotonated carbon C-2 at δ_C_ 133.4. With the correlation observed at δ_C_ 133.4, the further weak four-bond heteronuclear correlations observed between the methyl group C-3 (δ_C_ 153.2) suggested that the solvent acetonitrile reacted with resveratrol, which resulted in the formation of an oxazole ring.

The ^1^H NMR spectrum of **7** (Supporting Information, Figure S42) was very similar to that of **4**, except for the lack of H-4 aromatic hydrogen of the dihydroxyphenyl ring. The protonated molecular ion peak exhibited at *m*/*z* 475.00493 ([M + H]^+^ calculated: 475.00369) indicated the molecular formula of C_21_H_15_O_5_I (Supporting Information, Figure S27) thus it was presumed that H-4 was replaced by an iodine substituent in **7**. Its presence at C-4 was further confirmed by HMBC correlations of H-2/H-6 (δ_H_ 4.66, br s, 2H) with a shielded nonprotonated carbon seen at δ_C_ 74.0, a chemical shift characteristic for such a substituent. Therefore, compound **7** was identified as iodo-3β-(3,5-dihydroxyphenyl)-2α-(4″-hydroxyphenyl)­dihydrobenzofuran-5-carbaldehyde.

Based on the HRMS data, an elemental composition of C_18_H_22_O_5_ (Supporting Information, Figure S28) was established for compound **11**, indicating the incorporation of two ethoxy groups into the structure of resveratrol, similarly to our previously reported compound **8**.^31^ In the ^1^H and ^13^C NMR spectra of **11** (Supporting Information, Figure S45), signals of the 3,5-dihydroxyphenyl and 4-hydroxyphenyl moieties remained well identifiable, as in **8**, but the signals of a diethoxy substituted chiral H–C–C–H group were δ_H_ = 4.14 /δ_C_ = 85.9 and δ_H_ = 4.18 /δ_C_ = 85.6, *J*(H,H) = 6.6 Hz for **8** and δ_H_ = 4.20 /δ_C_ = 86.1 and δ_H_ = 4.28 /δ_C_ = 86.7, *J*(H,H) = 6.7 Hz for **11**. However, as also observed in **8**, the uniform NMR spectra were insufficient to assign **11** to either *threo* or *erythro* configuration.

The molecular formulas of both compounds **13** and **15** were established as C_18_H_21_O_5_I with the aid of HRMS data ([M – H]^−^, calculated: 443.03554, found: 443.03634) (Supporting Information, Figures S29 and S31, respectively). In the ^1^H and ^13^C NMR spectra of **13** and **15**, the H–C=C–H double bond signals of the parent compound resveratrol were replaced by signals of diethoxy substituted chiral oxymethines (δ_H_ = 4.18 d/δ_C_ = 85.5 and δ_H_ = 4.16 d/δ_C_ = 85.5, *J*(H,H) = 6.4 Hz) in **13** and (δ_H_ = 4.88 d/δ_C_ = 87.85 and δ_H_ = 4.28 d/δ_C_ = 84.93, *J*(H,H) = 5.3 Hz) in **15** (Supporting Information, Figures S48 and S53, respectively). The proton signals of 4-hydroxyphenyl moieties remained well identifiable in **13** and **15**, however, those of the 3,5-dihydroxyphenyl molecular parts were slightly different in both compounds. In the ^1^H NMR spectrum of **13**, the proton signals of the 3,5-dihydroxyphenyl moiety displayed at δ_H_ 6.45 (H-2, H-6) as a singlet revealed the presence of a substituent at C-4 of the symmetrical ring, which, according to the characteristic chemical shift of this carbon atom (δ_C_ 73.9), was determined to be iodine. Compound **15** possessed a similar aromatic substitution pattern to that of **12**,^31^ like an AX system of two doublets (^4^ *J* = 2.8 Hz) at δ_H_ 6.24 and 6.46 for H-6 and H-4, respectively. The presence of a *meta*-coupled proton pair, and the upfield shifted C-2 (δ_C_ 79.0) giving HMBC interactions with these hydrogens dictated that the iodine substitution took place at the position of C-2.

In the case of compound **14**, molecular formula C_14_H_10_O_3_I_2_ was established by HRMS data ([M + H]^+^, calculated: 480.87921, found [M + H]^+^: 480.87932, [M – H]^−^: 478.86471) (Supporting Information, Figure S30). In the ^1^H and ^13^C NMR spectra of **14** (Supporting Information, Figure S50), signals of the 4-hydroxyphenyl moiety and the H–C=C–H double bond remained well identifiable, suggesting that the substitution occurred on the 3,5-dihydroxyphenyl ring. The shielded carbons C-2 and C-4 resonating at δ_C_ 78.6 and 73.7, respectively, gave heteronuclear correlations with the singlet of H-6 (δ_H_ 6.95) (Supporting Information, Figure S51).

For compound **17**, HRMS measurements failed to yield an identifiable *m*/*z* value for the molecular ion or any clear fragments in both positive and negative modes. An elemental composition of C_28_H_20_O_6_I_2_ was proposed by using the calculated protonated molecular ion peak ([M + H]^+^, calculated: 706.94220). The ^1^H NMR spectrum (Supporting Information, Figure S56) of **17** contained the characteristic signals of a 4-hydroxyphenyl ring (δ_H_ 7.47 and 6.87, d, *J* = 8.5 Hz, 2H), a *trans* double bond (δ_H_ 7.26 and 7.08, d, *J* = 16.2 Hz, 1H) and an isolated aromatic methine seen at δ_H_ 6.97 (s, 1H). Several important carbon chemical shifts observed only in the HMBC spectra indicated the presence of an iodine substituent at C-4 (δ_C_ 74.5) forming a strong H-2/C-4 HMBC correlation and a heteronuclear 3-bond interactions of H-2 and the olefinic proton at δ_H_ 7.26 with a nonprotonated aromatic carbon at δ_C_ 110.4. The presence of the lone hydrogen, δ_H_ 6.97, unambiguously revealed that the C–C connection of iodine-substituted resveratrol monomers is formed through C-2 and C-2′. The indistinguishable monomer units of compound **17** could also be explained by the HRMS protonated molecular ion peak observed at *m*/*z* 354.16817 (Supporting Information, Figure S31).

The compounds obtained represent the structural diversity expected from our chemical approach. The occurrence of **2**, **6** and **16** in a biological environment has already been established in earlier reports.^31^ Activated XO produces large amounts of superoxide anion in the vascular system under pathophysiological conditions,^37^ and, interestingly, the O_2_^•–^ scavenging activity of resveratrol is higher in the xanthine/XO system.^18^ In vitro oxidation of resveratrol using xanthine oxidase led to the formation of minute amounts of compounds **4** and **6** as observed in the chromatographic fingerprint (Supporting Information, Figure S8). Similarly, due to the strong self-association of resveratrol fixed by strong π–π stacking in aqueous solution,^45^ compounds **1** and **4** were also expected products of resveratrol oxidation. Due to the availability of nitric oxide and its potential to form peroxynitrite in biological systems, nitro-derivatives, **18** and **19**, are also expectable products. Iodine-substituted monomers and dimers are valuable in expanding the chemical space of potentially bioactive semisynthetic metabolites. The structures of the compounds obtained are listed in Figure 1.

### 2.3 *In Silico* Evaluation of Drug-Likeness

After elucidating the structure of resveratrol’s oxidative metabolites, it became feasible to characterize their drug discovery potential. We utilized the ACD/Percepta software package^46^ emphasizing Lipinski’s rule of five (Ro5) for drug-likeness^47^ and augmenting it with the Ertl method to assess intestinal absorption.^48^ The principal physicochemical properties relevant to these compounds are summarized in Table 2, with the last column delineating the medicinal chemistry rule violations (MedChem issues) associated with any of them. Ro5 violations (Ro5!) were observed for five compounds (**1**, **9**, **10**, **14**, and **17**), with **17** designated critical due to significant deviations from the optimal druggability range for three of the parameters. The exceeding of the individual molecular weight (MW) limit and the increased lipophilicity (log *P*) in the case of compound **17** can be attributed to the presence of two incorporated iodine atoms. Given the polyphenolic characteristics of the tested resveratrol derivatives, we assessed them by the polar surface area—intestinal absorption correlation established by the Ertl method, which indicated that drugs with a PSA < 60 Å^2^ are nearly completely absorbed (over 90%), while those with a PSA > 140 Å^2^ exhibit less than 10% absorption. Consequently, a marginally reduced absorption is predicted for **1**, **10**, and **17**, however for **9**, a minimal absorption level is expected. The isolated resveratrol metabolites were categorized according to their neutral form or intrinsic aqueous solubility, which is crucial for bioavailability (moderate solubility: <0.1 mg/mL, poor solubility: <0.01 mg/mL). The predicted data demonstrated moderate aqueous solubility for **1**, **2**, **6**, **10**, and **14**, and poor solubility for **17**. Considering that the internal database of the ACD/Percepta software demonstrated the experimentally verified inhibitory effect of resveratrol on the cytochrome P450 1A2 (CyP1A2) isoenzyme,^49^ it was essential to further validate the corresponding drug–drug interaction (DDI) research *in silico*. The software identified a risk linked to the potential CyP1A2 inhibitory effect for **5**, **18**, and **19**. Considering the physicochemical and 2-D structural characteristics, the new resveratrol metabolites comply with the *in silico* drug-likeness criteria except for **1**, **9**, **10**, **14**, and **17**. However, for **5**, **18**, and **19**, further studies are necessary on their CyP1A2 inhibitory activity to allow a sound judgment on their potential as possible leads.

**Table 2:** Physicochemical Characterization of Resveratrol and Its ROS/RNS-Oxidized Metabolites (**1**–**19**) Using ACD/Percepta Suite^a^

compounds	MW	strongest p*K*_a,acid_	HBD/HBA	log *P*/log *D*_*7.4*_	TPSA Å^2^	solubility (mg/mL)	MedChem issues
**Resveratrol**	228.2	9.2	3/3	2.8/2.8	60.7	1.23	CyP1A2 inhibition
**1**	454.5	8.5	6/6	4.7/4.7	121.4	0.01	Ro5! (HBD)
**2**	454.5	9.2	5/6	4.2/4.2	110.4	0.08	-
**3**	267.3	9.0	2/4	2.9/2.9	66.5	0.17	-
**4**	348.4	9.2	3/5	3.0/3.0	87.0	0.28	-
**5**	354.1	7.8	3/3	3.5/3.4	60.7	0.35	CyP1A2 inhibition
**6**	454.5	9.2	5/6	4.1/4.1	110.4	0.04	-
**7**	474.3	7.8	3/5	3.9/3.7	87.0	0.11	-
**8**	318.4	9.2	3/5	2.9/2.8	79.2	0.31	-
**9**	516.5	9.2	7/8	3.9/3.9	150.8^##^	0.19	Ro5! (MW, HBD), TPSA!
**10**	500.5	9.2	6/7	4.5/4.5	130.6	0.02	Ro5! (MW, HBD)
**11**	318.4	9.2	3/5	2.9/2.8	79.2	0.31	-
**12**	354.1	8.1	3/3	4.1/4.0	60.7	0.24	-
**13**	444.3	7.7	3/5	3.8/3.6	79.2	0.17	-
**14**	480.0	6.6	3/3	5.1^a^/4.1	60.7	0.04^a^	Ro5! (log *P*)
**15**	444.3	8.0	3/5	4.1/4.0	79.2	0.13	-
**16**	262.7	8.1	3/3	3.7/3.6	60.7	0.36	-
**17**	706.3^##^	6.7	6/6	6.8^##^/5.7	121.4	0.002^##^	Ro5! (MW, HBD, log *P*)
**18**	273.2	8.8	3/6	2.5/2.5	106.5	0.5	CyP1A2 inhibition
**19**	273.2	6.8	3/6	3.0/2.3	106.5	0.5	CyP1A2 inhibition

a ^#^moderate or ^##^increased violations (using classical rule of five^46^) or for TPSA (^#^ > 120 Å^2^, ^##^ > 140 Å^2^) or for solubility (^#^ < 0.1 mg/mL, ^##^ < 0.01 mg/mL).

### 2.4 Cardiovascular Protective Activity

Human ACE is an excellent and clinically well-established target for the treatment of hypertension and related CVDs. Accordingly, to evaluate the cardioprotective potential of these compounds in comparison with that of resveratrol, the ACE inhibitory activity of these compounds was evaluated. Results are compiled in Table 3.

**Table 3:** ACE Inhibitory Activity and Ligand-Lipophilicity Efficiency (LLE) of Resveratrol and Isolated Pure Compounds^a^

compounds	ACE Inh. (%)	ACE IC_50_ (μM)	LLE
Resveratrol	38.6 ± 0.6	**185.8** ± 9.1	**0.9**
**1**	87.4 ± 1.6	**17.5** ± 4.8*	**0.0**
**2**	81.8 ± 1.0*	**31.8** ± 0.5*	**0.3**
**3**	33.4 ± 1.0	**106.4** ± 2.0	**1.0**
**4**	66.9 ± 2.6*	**41.6** ± 2.4*	**1.4**
**5**	70.9 ± 9.3*	**20.4** ± 2.2*	**1.2**
**6**	101.0 ± 0.4*	**9.2** ± 0.6*	**0.9**
**7**	82.0 ± 2.8*	**17.1** ± 1.8*	**0.9**
**8**	–7.9 ± 2.7*	**>1000**	**-**
**9**	91.5 ± 0.2*	**36.5** ± 0.8*	**0.5**
**10**	74.5 ± 5.3*	**33.3** ± 1.5*	**-0.1**
**11**	–2.9 ± 1.0*	**>1000**	**-**
**12**	90.5 ± 0.7*	**15.1** ± 1.5*	**0.7**
**13**	10.8 ± 2.1	**>1000**	**-**
**14**	81.8 ± 9.3*	**16.2** ± 1.7*	**-0.3**
**15**	0.5 ± 3.0*	>**1000**	**-**
**16**	62.4 ± 0.1	**61.6** ± 3.4*	**0.5**
**17**	71.4 ± 4.9*	**38.8** ± 1.1*	**-2.4**
**18**	10.7 ± 0.3*	**277.7** ± 7.8	**1.1**
**19**	28.1 ± 2.8*	**192.4** ± 2.5	**0.7**
Captopril	81.2 ± 1.0*	**0.12** ± 0.1*	**-**

a Results are expressed as mean ± SEM, *n* = 3 for % inhibition studies, and *n* = 2 for calculating IC_50_ values. % inhibition of the compounds was performed at 90 μM and captopril at 10 μM. *: *p* < 0.05 by one-way ANOVA using Dunnett’s multiple comparison test to the parent compound resveratrol. Ligand-lipophilicity efficiency values are calculated by LLE = pIC_50_ – log *P* using the log *P* values from Table 2.

It is a remarkable finding that most derivatives obtained in this study acted as more potent ACE inhibitors than otherwise moderately active resveratrol itself. Concerning structure–activity relationships, it was found that substituting one of the aromatic rings of resveratrol with iodine or chlorine increases the ACE inhibitory effect by ca. 3–12 times, in the order of **16** ≪ **5** < **14** ∼ **12**. Accordingly, the presence of a 4-iodo group seems more favorable than a 2-iodo group (**12** vs **5** and **14** vs **5**) in this regard, and much more favorable than a 2-chloro substitution (**16**). Open dimers, however, did not follow this rule (**1** vs **17**). Replacing the CH=CH double bond with ethoxy groups, as in **8** and **11**, resulted in a complete loss of ACE inhibitory activity, regardless of iodine substitution, as in **13** and **15**. Dimers, open and closed, were also observed to have improved bioactivities, and *trans*-δ-viniferin (**6**) was identified as the most potent ACE inhibitor in this study, over 20 times stronger than resveratrol. Connecting two monomers at one’s CH=CH bond, as in **9** and **10**, resulting in a decreased activity as compared to that of their aromatic ring-connected counterpart **1**. Still, ethoxy substitution of the open dimers did not result in such a dramatic loss of activity as that observed in the monomers **8** and **11**. It is worth noting that the fragmented dimers (**4** and **7**), clearly the result of subsequent oxidative transformations, were also active.

We employed the ligand-lipophilicity efficiency (LLE) metric^50^ to tackle and reduce the risk of promiscuity for ACE selective candidate selection, since a total of 11 derivatives were found to have IC_50_ values less than 50 μM. According to the method originally proposed by Leeson,^51^ derivatives **4**, **5**, **6**, and **7** with LLE ≥ 0.9 (equivalent to or larger than resveratrol) contain enthalpically more favorable binding characteristics for ACE (see Table 3). Altogether, based on its IC_50_ and LLE values and drug-like physicochemical properties, compound **6** is highlighted as the most promising ACE inhibitor candidate among the compounds prepared in this study.

ACE inhibitors exert cardioprotective activity by decreasing the production of angiotensin II while increasing bradykinin and endothelial NO levels,^52^ ACE inhibitors also inhibit ROS-generating enzyme systems,^53^ with several studies linking the potential antioxidant activity of ACE inhibitors to superoxide anion scavenging.^54,55^

### 2.5 Kinetic and Domain-Specific Studies of **6** and **12** as ACE Inhibitors

To evaluate the mode of ACE inhibition by the most active compounds **6** and **12**, enzyme kinetic studies were performed. Our results revealed, for the first time, compound **6** as a competitive, and compound **12** as a mixed-type inhibitor. Lineweaver–Burk transform plots of compounds **6** and **12** are presented in the Supporting Information, Figures S64–S65.

ACE comprises two homologous metallopeptidase domains, the N- and C-terminal domains (N-ACE and C-ACE, respectively), both of which are capable of cleaving angiotensin I but have different affinities for a range of other substrates and inhibitors.^56^ The C-domain is primarily responsible for converting angiotensin I to angiotensin II, and plays a major role in blood pressure regulation.^57,58^ Targeting the C-domain, therefore, seems to be necessary and sufficient for controlling blood pressure.^57^ Based on this, we studied the action of compounds **6** and **12** on the C-ACE and N-ACE using domain-specific substrates, and the dose-dependent inhibition of the compounds on each domain is presented in Table 4. The highly domain-selective substrates Abz-SDK­(Dnp)­P-OH (for N-ACE) and Abz-LFK­(Dnp)-OH (for C-ACE) were used to investigate domain-selective inhibition. To validate the method, angiotensin II and a highly specific C-ACE inhibitor, bradykinin potentiating peptide B (BPPb) were used as positive controls.

**Table 4:** Inhibitory Activity of Compounds **6** and **12** on the C- and N-Terminal Domain of Rabbit Lung ACE^a^

	C-domain		N-domain	
inhibitor	inhibition (%)^b^	IC_50_ (μM)	inhibition (%)	IC_50_ (μM)
**6**	46.3 ± 4.2	**17.1** ± 1.2	22.4 ± 0.7	**56.4** ± 5.2
**12**	35.0 ± 3.3	**35.1** ± 1.5	12.8 ± 1.9	**104.8** ± 3.4
BPPb	80.9 ± 3.1	**n.d.**	0.6 ± 0.4	**n.d.**
Angiotensin II	36.8 ± 1.1	**n.d.**	6.1 ± 1.1	**n.d.**

a Results are expressed as mean ± SEM, *n* = 3 for % inhibition and *n* = 2 for dose–response studies; n. d. = not determined. For both % inhibition and dose–response studies, the substrate concentration [*S*] = *K*_M_ was calculated from initial velocity studies of both substrates; Abz-SDK­(Dnp)­P-OH; [*S*] = 79 μM and Abz-LFK­(Dnp)-OH; [*S*] = 33 μM.

b Inhibition percentage was determined at 10 μM for angiotensin II, and compounds **6** and **12**, and at 200 nM for BPPb.

For both natural oligopeptides, inhibition of the C-domain was significantly higher than that of the N-domain at the same inhibitor concentration, which is in agreement with previous reports.^59,60^ Both compounds **6** and **12** also preferentially inhibited the C-domain part of the ACE. The structure of **6** bound to the ACE; C-domain (*K*_i_ = 9.9 × 10^–6^ M) and N-domain (*K*_i_ = 2.7 × 10^–5^ M) showed that compound **6** preferentially inhibits the C-domain of the enzyme, with a selectivity factor (*K*_i_N/*K*_i_C) of 2.74.

### 2.6 Chemical and Biological Evaluation of *trans*-δ-Viniferin Enantiomers

Since the preparation of the potent and favorable C-domain-specific ACE inhibitor *trans*-δ-viniferin (**6**) did not involve any chiral selector, this compound was expectedly a racemic mixture. This was confirmed by chiral HPLC using an isocratic elution of *n*-hexane-ethanol (82:18, v/v) on a cellulose-based chiral column (Chiralcel OD-H) (Figure 2A). Pure enantiomers of **6** were separated (Supporting Information, Figure S66) in a similar workflow as previously reported by Huber and co-workers.^61^ Purity of each isolated enantiomer (**6a** and **6b**) was confirmed by subsequent analytical chiral-HPLC-PDA, and they were subjected to vibrational circular dichroism (VCD) analysis to determine their absolute configuration (Figure 2B).

**Figure 2:**
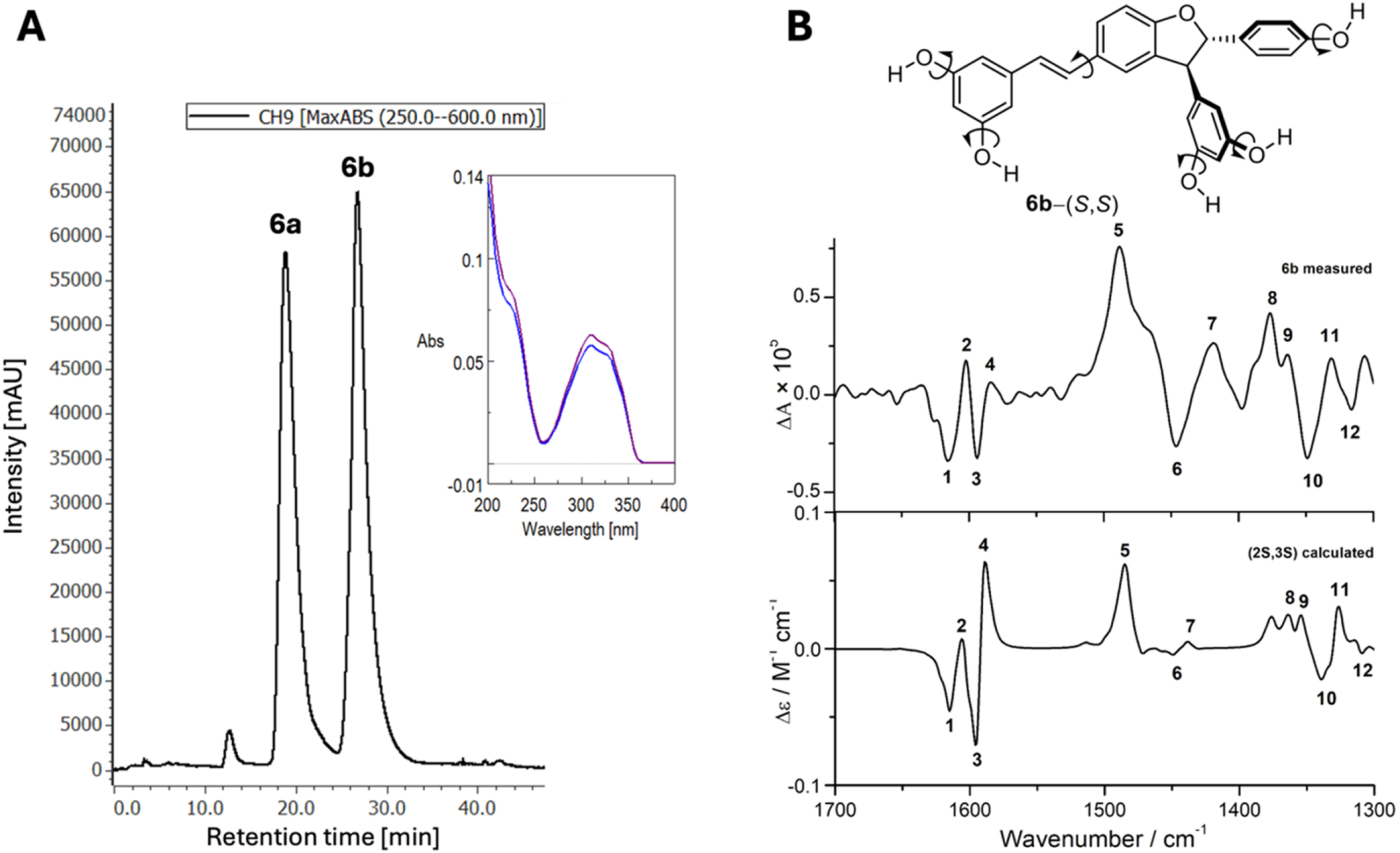
**A:** Chiral HPLC-UV fingerprint of racemate **6** using an isocratic mobile phase of hexane −CH_3_OH (82:18, v/v) on Chiralcel OD-H column (250 × 4.6 mm, 5 μm, Daicel), along with the UV spectra of both peaks. **B:** Comparison of the measured VCD spectrum of **6b** recorded in DMSO-*d*_6_ (top) with the calculated spectrum of the (2*S*,3*S*) enantiomer (bottom, obtained as a population-weighted theoretical sum VCD spectrum of 64 conformers at the B3LYP/6-311++G­(d,p) level of theory, using a PCM solvent model for DMSO). Matching VCD bands are marked with identical numbers. Curved arrows on the structure show rotation around five C–O bonds and one C–C bond.

Based on the comparison of its experimental and theoretical VCD spectra of the separated **6b**, it can be stated that the absolute configuration of this enantiomer is (*S*,*S*), as a convincing agreement is discernible between the measured spectrum and the population-weighted (average) calculated spectrum. By rotation around five C–O bonds and one C–C bond, as indicated in Figure 2B, 64 rotamers were generated and analyzed for the computational simulation of the theoretical VCD spectrum of **6b**-(*S*,*S*) (Figure 2B). It is of note that comparable populations in the range of ca. 3–0.5% without significantly abundant ones were identified for this set of rotamers, indicating their similar thermodynamic stability. However, during each optimization, the 4-hydroxyphenyl group and the 3,5-dihydroxyphenyl group attached to carbons C2 and C3, respectively, ended up in positions nearly perpendicular to the plane of the dihydrobenzofurane skeleton. Consequently, no further rotamers were constructed by rotation of these substituents on the stereogenic centers.

Subsequent evaluation of the ACE inhibitory activity showed that **6a-(** *R*,*R* **)** was significantly more potent than its stereoisomeric counterpart, **6b-(** *S*,*S* **)** (Table 5).

**Table 5:** ACE Inhibitory Activity of Resveratrol and Isolated Enantiopure Compounds^a^

compound	ACE inhibition (%)	ACE IC_50_ (μM)
**6**	96.3 ± 0.3	**10.9** ± 0.1
**6a**-(*R*,*R*)	99.4 ± 0.5	**8.7** ± 0.6
**6b**-(*S*,*S*)	91.2 ± 0.9	**12.1** ± 0.1*

a Results are expressed as mean ± SEM, *n* = 4 for % inhibition studies and IC_50_ estimation. Inhibition % of the compounds was tested at 50 μM. *: *p* < 0.05 by unpaired *t*-test assuming Gaussian distribution (parametric test) between the enantiomers.

After chemical and biological studies on the enantiomers, docking simulations of the interactions between both compounds and ACE were performed at the C- and N-terminal active sites. Results for the best-docked positions are presented in Figure 3; binding energies are presented in Supporting Information, Tables S1–S2.

**Figure 3:**
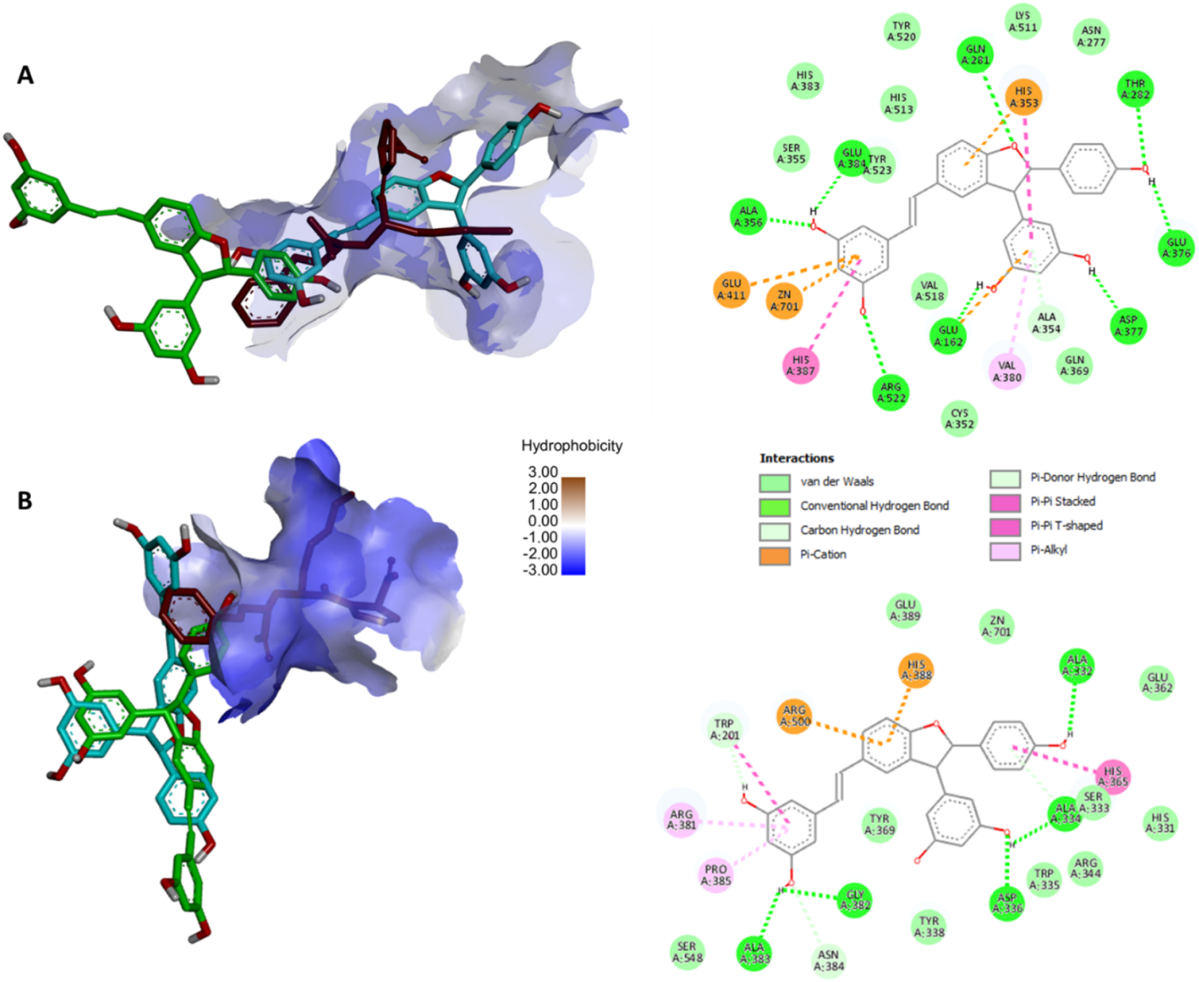
**A**: Best-docked position of enantiomers **6a-(** *R*,*R* **)** in blue and **6b-(** *S*,*S* **)** in green, along with the experimental position of lisinopril (red) in the ACE C-domain active site along with the interaction map of **6a-(** *R*,*R* **). B**: The best-docked position of enantiomers, **6a-(** *R*,*R* **)** in blue and **6a-(** *S*,*S* **)** in green, along with the experimental position of lisinopril (red) in the ACE active site along with the interaction map of **6b-(** *S*,*S* **)** in the A-chain of ACE N-domain.

Superimposing the best-docked orientation of the enantiomers with the experimentally bound lisinopril, an approved hypertension drug, in the crystal structure of ACE C- and N-domains (pdb ID: 1O86 and 2C6N, respectively)^62,63^ provided important insights into the difference observed between the bioactivities of **6a-(** *R*,*R* **)** and **6b-(** *S*,*S* **)**.

Docking calculations showed that **6a-(** *R*,*R* **)** has a higher binding affinity to the C-domain than **6b-**(*S*,*S*), −10.38 and −9.9 kcal/mol, respectively, in their best-docked position (Figure 3A). The orientation of **6a-(** *R*,*R* **)** shows that it binds to the active site of C-ACE similarly to lisinopril, which may explain its higher inhibitory potential compared to its enantiomeric pair, **6b-(** *S*,*S* **)**. Zinc, an important catalytic component of ACE, is bound at the active site to His383, His387 and Glu411,^62^ and therefore, the ionic interactions observed between **6a-(** *R*,*R* **)** and Glu411 and Zn701 might provide a structural basis to the inhibitory potential of this compound. Several hydrogen bonds were observed between the phenolic hydroxyl groups of **6a-(** *R*,*R* **)** and amino acid residues at the active site, notably with Arg522, Asp377 and Glu162. Arg522 is an important residue required for chloride activation in the active site.^64^ Compound **6a-(** *R*,*R* **)** interacted with Glu376 and Val380 that were previously found as unique residues contributing to the C-domain selectivity of ACE inhibitors lisW-S, kAW and RXPA380.^58,64^ The polar interactions of **6a-(** *R*,*R* **)** with Ala354 and His353, respectively, also contribute to the inhibitory activity of this compound as similar interactions between the pseudoproline side chain of RXPA380 and the amino acid residues was previously reported.^64^

In the best-docked orientation of both enantiomers in the A subunit of N-ACE, docking calculations showed approximately the same orientation and binding affinity of **6a-(** *R*,*R* **)** and **6b-(** *S*,*S* **)** in the active site of the N-domain (with a binding energy of −10.02 kcal/mol and −10.07 kcal/mol, respectively) as shown in Figure 3B. Several polar and nonpolar interactions were observed between each enantiomer and some N-domain residues, similarly as observed in the N-domain-selective inhibitors RXP407 and 33RE.^65^ However, no interaction was observed with Tyr369, a residue with a major contribution to the selectivity of the aforementioned compounds.^65^ Interactions observed between **6a**-(*R*,*R*) and **6b**-(*S*,*S*) and these residues provide a mechanistic background to the compounds’ ACE inhibitory activity.

### 2.7 Anti-Inflammatory Activities

Independent of their blood pressure-lowering effects, ACE inhibitors may reduce vascular inflammation^54,66,67^ that plays a pivotal role in the pathogenesis of cardiovascular disease.^14^ Angiotensin II initiates an inflammatory cascade of NADPH oxidase, ROS, and nuclear factor-κB, which in combination exert a proinflammatory effect on the cardiovascular system.^12^ Therefore, the potential anti-inflammatory activities of the isolated compounds compared to resveratrol were also determined by evaluating their inhibition on LOX, COX-1, and COX-2 enzymes; results are shown in Table 6.

**Table 6:** Anti-Inflammatory Activities of Resveratrol and Compounds **1**–**19** ^a^

compounds	COX-1 Inh.(%)/IC_50_ (μM)/LLE	COX-2 Inh.(%)/IC_50_ (μM)/LLE	LOX Inh.(%)/IC_50_ (μM)/LLE
**Resveratrol**	98.6 ± 0.2/**2.9** ± 1.0/**2.7**	95.8 ± 1.3/**5.4** ± 0.1/**2.4**	4.3 ± 6.3/>400
**1**	65.2 ± 5.2/n.d.	86.5 ± 2.8/**5.2** ± 0.3/**0.5**	59.8 ± 7.4/**53.4** ± 5.0/-**0.5**
**2**	80.0 ± 1.4/**9.1** ± 1.6/**0.9**	91.7 ± 2.5/**3.4** ± 0.1/**1.3**	85.8 ± 3.9/**9.7** ± 2.8/**0.8**
**3**	41.5 ± 4.1/n.d.	72.0 ± 5.2/n.d.	35.0 ± 7.1/**85.7** ± 20.1/**1.1**
**4**	37.9 ± 3.9/n.d.	80.1 ± 2.5/**19.6** ± 1.4/**1.7**	9.9 ± 6.9/241.1 ± 21.0/0.6
**5**	73.9 ± 5.3/21.3 ± 0.7/1.1	78.5 ± 2.1/n.d.	18.7 ± 5.0/120.6 ± 4.9/0.4
**6**	98.6 ± 0.3/4.7 ± 0.3/1.2	92.7 ± 0.9/5.6 ± 0.6/1.1	73.1 ± 4.4/22.4 ± 8.8/0.5
**7**	37.1 ± 2.8/n.d.	60.6 ± 5.7/n.d.	–14.5 ± 15.8/n.d.
**8**	16.8 ± 7.9/n.d.	38.9 ± 4.9/n.d.	–2.7 ± 4.7/n.d.
**9**	29.5 ± 6.0/n.d.	77.7 ± 2.9/n.d.	13.5 ± 14.8/99.6 ± 5.1/0.1
**10**	65.9 ± 13.1/n.d.	85.2 ± 2.0/12.2 ± 0.2/0.4	–8.6 ± 7.6/n.d.
**11**	17.4 ± 3.3/n.d.	19.2 ± 6.0/n.d.	–7.4 ± 8.6/n.d.
**12**	67.3 ± 2.9/n.d.	93.2 ± 0.9/11.1 ± 0.4/0.8	19.9 ± 12.8/97.2 ± 2.4/-0.1
**13**	29.8 ± 16.4/n.d.	70.2 ± 1.6/n.d.	–11.5 ± 9.0/n.d.
**14**	26.5 ± 0.6/n.d.	67.1 ± 3.6/n.d.	20.3 ± 5.0/97.2 ± 2.4/-1.0
**15**	9.5 ± 1.9/n.d.	37.9 ± 3.6/n.d.	–1.2 ± 6.8/n.d.
**16**	49.1 ± 3.3/n.d.	88.2 ± 1.1/13.3 ± 0.1/1.2	11.6 ± 4.7/94.4 ± 3.8/0.3
**17**	42.6 ± 17.8/n.d.	60.6 ± 2.8/n.d.	24.2 ± 8.1/174.9 ± 2.2/-3.1
**18**	55.8 ± 4.0/n.d.	48.8 ± 4.2/n.d.	6.2 ± 1.1/>400
**19**	77.0 ± 1.1/19.8 ± 0.1/1.7	57.5 ± 4.9/n.d.	–0.48 ± 3.8/n.d.
control	100.4 ± 10.6/0.011 ± 0.1	77.7 ± 1.4/0.5 ± 0.1	80.19 ± 6.4/3.5 ± 0.8

a Positive controls were SC560, Celecoxib, and NDGA for COX-1, COX-2, and LOX, respectively. Results are expressed as mean ± SEM, *n* = 3 in each case. Compounds were tested at 100 μM for LOX % inhibition and 50 μM for COX-1 and COX-2% inhibition. Dose-response studies were carried out on compounds exhibiting ≥70 and 80% inhibition for COX-1 and COX-2, respectively; n.d. = not determined. Ligand-lipophilicity efficiency values were calculated as LLE = pIC_50_ – log *P* using log *P* values from Table 2.

15-LOX appears to be important for the development of atherogenesis and its inhibition is considered an important mechanism in the treatment of heart failure.^68^ The oxidation of resveratrol resulted in a marked increase in LOX inhibitory activity, with *trans*-ε-viniferin as the most potent compound, corroborating similar studies on Fe-catalyzed oxidation of resveratrol.^42^ Open ring dimers **1** and **9** also had improved LOX inhibitory activity, and these compounds may, expectably, be formed in biological environments. However, it is also of interest that some less biorelevant iodinated compounds (**5** and **14**, with a mono- and disubstitution, respectively) also exhibited pronounced LOX inhibition.

We assessed the anti-inflammatory effects using the ligand-lipophilicity efficiency (LLE) metric, which is equivalent to the promiscuity risk evaluation utilized for the ACE IC_50_ data (see Table 6). Only the relative LLE values could be assessed since, in the case of COX-1 and COX-2, the oxidized derivatives could not be proven to possess an additional effect in comparison to resveratrol. Thus, rating the resveratrol derivatives revealed that on COX-1 they had the enthalpically beneficial order of effect as **19** > **6** > **5** > **2**, whereas in case of COX-2, this was **4** > **2** > **16** > **6** > **12** > **1**. Because resveratrol was ineffective and only IC_50_ values below 50 μM can reasonably be taken into consideration, evaluation of the LOX inhibition data was simpler. Compounds **2** and **6** are the two main enthalpically valuable candidates, which could potentially be considered as in the order related to the LLE value.

Resveratrol has been reported as an effective inhibitor of cyclooxygenase activity *in vivo* through moderately selective inhibition of COX-1 activity and/or reduction of COX-2 at the mRNA level.^69^ Inhibiting COX-1 prevents the formation of thromboxane A2 (TxA_2_), a potent inducer of platelet aggregation and a vasoconstrictor, which can lead to thrombus formation and subsequent blockages in blood vessels that result in transient myocardial infarction.^70^ Resveratrol restricts atherosclerosis-associated inflammation by suppressing the expression and activity of COX-2 and the downstream prostaglandin, PGE2, important in inflammation at the arterial wall.^71^ Most of the oxidized derivatives obtained in this study were less active COX inhibitors than resveratrol, with the exception of viniferins **2** and **6**, which were similarly potent. Interestingly, compound **2** had a nearly 3-times selectivity toward COX-2, in contrast with resveratrol that was slightly COX-1 selective in this study.

## 3 Conclusions

In this early phase drug discovery study, we demonstrated that scavenging various types of biologically relevant ROS/RNS by resveratrol leads to the formation of a wide range of chemical metabolites, many of which act as much stronger ACE inhibitors or anti-inflammatory agents than their parent compound resveratrol itself.

To the best of our knowledge, this is the first report of *trans*-δ-viniferin (**6**) acting as a potent ACE inhibitor with a competitive binding mode and a clear preference for the ACE C-domain. Based on its in vitro pharmacodynamic and *in silico* physicochemical properties, this compound can be highlighted as the best candidate for further studies among all metabolites tested in this study. This finding, together with its anti-inflammatory activities and the marked xanthine oxidase inhibitory potential previously reported by us, strongly suggests that *trans*-δ-viniferin has a high overall cardiovascular protective effect.

Based on the well-known crucial importance of ACE and vascular inflammation in cardiovascular disease, our results also provide important new insights into how resveratrol consumption may lead to cardiovascular benefits in an organism facing oxidative stress. The formation of *trans*-δ-viniferin (**6**) in nearly half of our reactions is of particular interest and deserves further studies in cellular or in vivo pharmacological models to evaluate its potential role as an oxidative stress-induced bioactive metabolite.

Our approach was purely chemical, and none of the identified derivatives were confirmed as metabolites *in vivo*. Still, our findings clearly show that free radical scavenging by resveratrol leads to a wide range of valuable metabolites significantly shifting the overall bioactivity profile toward cardiovascular benefits. Using this as a proof of concept, we suggest that a diversity-oriented and bioactivity-guided exploration of the antioxidant scaveng­(e)­ome may be used as a high-hit-rate strategy for drug discovery.

## 4 Experimental Section

### 4.1 General Information

Resveratrol, with a purity of >98% (by HPLC analysis), was purchased from Career Henan Chemical Co. (Henan province, China). Angiotensin-converting enzyme (A6778), angiotensin II (A9525), Abz-LFK­(Dnp)-OH trifluoroacetate salt (A5855), Abz-SDK­(Dnp)­P-OH trifluoroacetate (A5730), xanthine (0626) was purchased from Sigma-Aldrich. Bradykinin-Potentiator B (BPPb) (PeptaNova GmbH, Germany, Sigma) and Abz-Gly-Phe­(NO_2_)-Pro (4003531, Bachem, Switzerland) were also used. All reagents and organic solvents were analytical grade and were used as purchased from Sigma-Aldrich and Reanal Laboratory Chemicals (Budapest, Hungary). HPLC solvents were purchased from ChemLab (Zedelgem, Belgium).

### 4.2 General Procedures for Resveratrol Oxidation

Oxidative reactions were carried out on resveratrol, with reactions continuously monitored by thin-layer chromatography, solid phase: Silica 60 F254 (250 μm, Merck Co., Ltd., Darmstadt, Germany); liquid phase: chloroform/ethyl acetate/formic acid (2.5:1:0.1, v/v/v) at regular intervals with visualizations performed under UV light, λ_1_ = 254 nm and λ_2_ = 365 nm. At the end of each reaction, the mixture was evaporated, extracted with ethyl acetate, and evaporated in vacuo. As a prepurification step, each residue was filtrated through silica with hexane–acetone (1:1, v/v) and then evaporated in vacuo. To obtain the chromatographic fingerprint of each oxidized mixture, the dried residue was dissolved in CH_3_CN, and a 10 μL aliquot of each mixture was analyzed by HPLC (PU-2080 pumps; AS-2055 Plus autosampler; MD-2010 Plus PDA detector, Jasco Co., Ltd., Tokyo, Japan) under the following conditions: column, Kinetex XB-C18 (250 × 4.6 mm, 5 μm); solvent system, water (solvent A) and CH_3_CN (solvent B): elution, linear gradient from 25% solvent B to 75% solvent B for 25 min and then isocratic mode for 75% solvent B for 2 min; flow rate, 1 mL/min; detection, 199–650 nm. The purity of the isolated metabolites was also determined using the same chromatographic conditions, and all compounds were >95% pure by HPLC analysis (HPLC traces for lead compounds, Supporting Information, Figures S68–S70).

The isolation and purification of metabolites from selected oxidized mixtures was carried out using an Armen Spot Prep II integrated HPLC purification system (Gilson, Middleton, WI, USA) using a Kinetex XB-C18 100 or Biphenyl column (250 × 21.2 mm, 5 μm), and eluents chosen appropriately. Further purification of metabolites, if necessary, was performed using an Agilent 1100 Series semipreparative HPLC system (Agilent Technologies, Waldbronn, Germany), using a Gemini-NX C18 or Phenyl-hexyl column (250 × 10.0 mm, 5 μm) or a Luna Silica column (250 × 4.6 mm, 5 μm, 100 Å) and the appropriate eluent.

#### 4.2.1 Reaction with PIFA in Acetonitrile (Ox1)

The reaction was performed as previously published.^31^ Briefly, resveratrol (300 mg/150 mL in acetonitrile) was oxidized by 1 equiv of PIFA (in 150 mL of acetonitrile) for 5 h at room temperature, then evaporated under vacuum and worked up by liquid–liquid partition and solid-phase extraction. The residue was purified by preparative HPLC using an isocratic elution of CH_3_CN/H_2_O (28:72, v/v) on a biphenyl column to obtain compounds **1** (7.64 mg), **2** (39.80 mg), and **3** (15.61 mg). Compound **1** was also further purified using a semipreparative HPLC Gemini-NX C18 column (250 × 10.0 mm, 5 μm) with isocratic elution using CH_3_OH/H_2_O (43:57, v/v) to obtain 3.15 mg of pure compound. Compound **2** was further purified by HPLC on a Luna Silica column (250 × 4.6 mm, 5 μm, 100 Å) using an elution of cyclohexane-isopropanol (86:14, v/v) to obtain 10.13 mg of pure compound. Compound **3** was also further purified using a semipreparative HPLC Gemini-NX C18 column (250 × 10.0 mm, 5 μm) with isocratic elution using CH_3_OH/H_2_O (44:56, v/v) to obtain 2.93 mg of pure compound.

#### 4.2.2 Reaction of Resveratrol with AAPH and NaIO_4_ (Ox2)

The reaction was conducted according to the method reported in prior study.^31^ Briefly, resveratrol (500 mg/250 mL in acetonitrile) was oxidized by 250 mL of aqueous solution of 1.5 equiv of AAPH and 1 equiv of NaIO_4_ (in 250 mL of acetonitrile) for 23 h at 65 °C. The reaction was stopped by adding an aqueous solution of reduced glutathione (541.12 mg/100 mL), cooled, then evaporated in vacuo, and worked up by liquid–liquid partition and solid-phase extraction. The residue was purified by preparative HPLC on a C18 column with an isocratic elution of CH_3_OH/H_2_O (50:50, v/v) to give a fraction containing **4** and **5** (46.57 mg), **6** (24.00 mg) and **7** (9.21 mg). The purified fractions were further separated on a Kinetex C18 100 Å column (250 × 21.2 mm, 5 μm) using CH_3_OH/H_2_O (45:55, v/v) to obtain compounds **4** (3.24 mg), **5** (12.93 mg), **6** (11.38 mg) and **7** (2.65 mg).

#### 4.2.3 Reaction of Resveratrol with PIDA in Acetonitrile (Ox3)

The reaction was performed as previously published.^31^ Briefly, resveratrol (100 mg/25 mL in acetonitrile) was oxidized by a 75 mL acetonitrile solution of 2 equiv of PIDA for 2 h at room temperature; then, the reaction stopped with an aqueous solution of reduced glutathione (265.25 mg/30 mL), and the mixture was subsequently evaporated under vacuum and worked up by liquid–liquid partition and solid-phase extraction. The residue of the combined organic layers was purified by preparative HPLC using the biphenyl column and solvent, CH_3_OH/H_2_O (52:48, v/v) resulting in compound **6** (6.37 mg).

#### 4.2.4 Reaction with PIFA in Ethanol (Ox4)

The reaction was performed following the method previously published.^31^ Briefly, resveratrol (250 mg/50 mL in ethanol) was oxidized by 1 equiv of PIFA (in 200 mL of ethanol) for 90 min at room temperature, stopped by adding an aqueous solution of reduced glutathione (673.75 mg/187.5 mL), and then evaporated in vacuo and worked up by liquid–liquid partition and solid-phase extraction. The residue of the combined organic layers was purified by preparative HPLC on a biphenyl column with an isocratic elution of CH_3_CN/H_2_O (31:69, v/v) to give compounds **8** (24.80 mg), **9** (13.30 mg) and **10** (33.98 mg). Further purification was carried out on compounds **8** and **10** using the same column with an isocratic elution of CH_3_OH/H_2_O (52:48, v/v) to obtain compounds **8** (11.93 mg) and **10** (22.62 mg), respectively. Compound **9** was further purified by HPLC on a Luna Silica column (250 × 4.6 mm, 5 μm, 100 Å) using an elution of cyclohexane-isopropanol (85:15, v/v) to obtain 8.38 mg of pure compound.

#### 4.2.5 Reaction of Resveratrol with Oxone and H_5_IO_6_ in Ethanol (Ox5)

The reaction was performed as previously published.^31^ Briefly, resveratrol (600 mg/300 mL in ethanol) was oxidized by an ethanol solution of Oxone (4.05 mg/150 mL) and 0.66 equiv of H_5_IO_6_ in 180 mL of ethanol for 7 h at room temperature. The reaction was stopped by adding an aqueous solution of reduced glutathione (1615.50 mg/150 mL), and the solvent was evaporated under vacuo and worked up by liquid–liquid partition and solid-phase extraction. The dry residue of the combined organic layers was purified by preparative HPLC using a biphenyl column with an isocratic elution with CH_3_OH/H_2_O (54:46, v/v) to obtain compounds **8** and **11** (60.21 mg), **5** (167.30 mg), **12** (61.94 mg), **13** (59.87 mg), **14** (15.19 mg) and **15** (97.99 mg). Further purification was carried out on the fractions under the same conditions as above to obtain **11** (12.41 mg), **8** (18.66 mg), **5** (167.30 mg), **12** (18.30 mg), **13** (25.94 mg), **14** (2.70 mg) and **15** (20.09 mg).

#### 4.2.6 Reaction of Resveratrol with FeCl_3_ and H_5_IO_6_ in Acetonitrile (Ox6)

The reaction was conducted according to the method reported in prior study.^31^ Briefly, resveratrol (480 mg) dissolved in 240 mL of acetonitrile was oxidized by 0.03 equiv of FeCl_3_ in 80 mL of acetonitrile solution and 450 mL of acetonitrile solution of 0.8 equiv. H_5_IO_6_ for 17 h at room temperature. The reaction was stopped by adding an aqueous solution of reduced glutathione (1293 mg/240 mL) and then evaporated under vacuo and worked up by liquid–liquid partition and solid-phase extraction. The residue of the combined organic layers was purified by preparative HPLC using a biphenyl column and an isocratic elution of CH_3_OH/H_2_O (51:49, v/v) to obtain compounds **16** (29.94 mg), **5** (27.06 mg) and **12** (63.61 mg). Further purification was carried out on the C18 column with isocratic elution CH_3_OH/H_2_O (42:58, v/v) to obtain compounds **16** (12.01 mg), **5** (9.13 mg) and **12** (22.27 mg).

#### 4.2.7 Reaction of Resveratrol with FeCl_3_ and H_5_IO_6_ in Ethanol (Ox7)

The reaction was performed as previously published.^31^ Briefly, resveratrol (360 mg/180 mL in ethanol) was oxidized by 0.03 equiv. FeCl_3_ in a 60 mL ethanol solution and 180 mL ethanol solution of 1.1 equiv. H_5_IO_6_ for 17 h at room temperature, with the reaction stopped by adding an aqueous solution of reduced glutathione (969.9 mg/218 mL), then evaporated under vacuo and worked up by liquid–liquid partition and solid-phase extraction. The residue of the combined organic layers was purified by preparative HPLC using a biphenyl column and an isocratic elution of CH_3_OH/H_2_O (51:49, v/v) to obtain a mixture of compounds **5** and **13** (107.99 mg), **17** (57.58 mg) and **14** (64.23 mg). The fractions containing compounds **5** and **13** were further purified by preparative HPLC using the Kinetex C18 100 Å column with an isocratic elution of CH_3_OH/H_2_O (42:58, v/v) and a semipreparative HPLC Gemini-NX C18 column with CH_3_OH/H_2_O (42:58, v/v) to obtain **5** (3.11 mg) and **13** (7.67 mg). Compound **17** was further purified on the Kinetex C18 100 Å column with isocratic elution, CH_3_OH/H_2_O (47:53, v/v) to obtain **17** (6.05 mg). Compound **14** was also further purified by preparative HPLC using the Kinetex C18 100 Å column with isocratic elution, CH_3_OH/H_2_O (47:53, v/v) and a semipreparative HPLC Gemini-NX C18 column (250 × 10.0 mm, 5 μm) with isocratic elution using CH_3_OH/H_2_O (42:58, v/v) to get **14** (6.15 mg).

#### 4.2.8 Reaction of Resveratrol with AIBN in Acetonitrile (Ox8)

To a solution of resveratrol (160 mg) dissolved in DMSO-acetonitrile (7 mL, 1:6, v/v), 20 mL ethyl linoleate (L1751, Sigma-Aldrich) and an acetonitrile solution of AIBN (3586.2 mg/18 mL) was added, and the mixture stirred for 8 h at 40 °C. The reaction was cooled in an ice bath and subsequently kept at −20 °C overnight. This was filtered using a Whatman filter paper (10001213020, 90 mm, IDL GmbH & Co. KG), with the filtrate evaporated under vacuo, and the dry residue was purified using flash chromatography on a silica column (Gold 40 g) with gradient elution of hexane/ethyl acetate (10:40% ethyl acetate for a total run time of 60 min) to obtain compound **6** (23.34 mg). Further purification was carried out on the C18 column with isocratic elution of CH_3_CN/H_2_O (28:72, v/v) to obtain a pure compound, **6** (3.19 mg).

#### 4.2.9 Reaction of Resveratrol with Peroxynitrite in Acetonitrile (Ox9)

Peroxynitrite was prepared according to the method by Fási et al.^30^ The concentration of the peroxynitrite stock solution in 0.1 M NaOH was determined by measuring the absorbance at 302 nm using 1670 M^–1^ cm^–1^ as the molar extinction coefficient. Eight vials, each containing a solution of resveratrol (114.13 mg/50 mL acetonitrile) mixed with 50 mL, 0.1 mM peroxynitrite (prepared prior to the reaction by the diluting stock solution with 0.1 M NaOH), were stirred for 5 min at room temperature. The reaction was stopped by adding drops of diluted HCl until pH 2. The solvent was evaporated under vacuo and the residue was partitioned between water (200 mL) and ethyl acetate (3 × 150 mL). Dry residue of the combined organic layers was purified by silica gel column chromatography eluted with acetone/hexane (1:1, v/v) and thereafter evaporated under vacuo to give a combined residue of 518.52 mg. The residue was subsequently purified by preparative HPLC using an isocratic elution of CH_3_CN/CH_3_OH/H_2_O (23:6:71, v/v/v) on a biphenyl column to obtain compounds **2** (17.00 mg), **6** (22.63 mg) and **19** (18.61 mg).

#### 4.2.10 Reaction of Resveratrol with NaNO_2_ in Phosphate Buffer, pH 3.0 (Ox10)

This was done according to the method reported by Panzella et al.,^36^ with changes made to the method of terminating the reaction. Phosphate buffer, 0.1 M was prepared by dissolving 12 g of sodium dihydrogen phosphate in 800 mL, pH adjusted with phosphoric acid to pH 3.0 and made up to 1000 mL. Three reaction flasks, each containing a solution of resveratrol (85.5 mg in 15 mL of acetonitrile), were combined with a phosphate-buffered sodium nitrite solution (207 mg in 1485 mL), with the mixtures stirred for 1 h at 37 °C. Each reaction was stopped by adding an aqueous solution of reduced glutathione (231 mg/300 mL). Combined reaction mixtures were partitioned with ethyl acetate (2 × 1800 mL) and evaporated in vacuo to give a combined dry residue (176.87 mg). The residue was subsequently purified by preparative HPLC using an isocratic elution of CH_3_CN/CH_3_OH/H_2_O (22:6:72, v/v/v) on a biphenyl column to obtain compounds **4** (4.15 mg), **18** (13.03 mg) and **19** (2.84 mg).

#### 4.2.11 Reaction of Resveratrol with NaNO_2_ in KCl-HCl, pH 2.0 (Ox11)

Similarly to a previously described oxidation method,^35^ a solution of 3.4 mg NaNO_2_ in 400 mL 50 mM KCl-HCl (prepared by dissolving 1.45 g of KCl in 400 mL 0.02 M HCl solution) was added to a solution of resveratrol (11.84 mg/400 mL in acetonitrile), and the mixture was stirred for 2 h at 37 °C. The reaction was stopped by adding an aqueous solution of reduced glutathione (12 mg/80 mL). The solvent was evaporated, the residue partitioned between water and ethyl acetate, and the organic layer evaporated to give a combined dry residue required for HPLC analysis.

#### 4.2.12 Reaction of Resveratrol with AAPH and H_2_O_2_ in Acetonitrile (Ox12)

To a solution of resveratrol (10 mg) in acetonitrile (6 mL) was added an aqueous solution of AAPH (17.8 mg/5 mL) and 2.5 mL of hydrogen peroxide (50 mM) and the mixture was stirred for 26 h at 65 °C. The reaction was stopped by adding an aqueous solution of reduced glutathione (26.95 mg/5 mL), keeping it in the same condition for 5 more min as before. Thereafter, the reaction mixture was cooled in an ice bath, and the solvent evaporated. The residue was partitioned between water and ethyl acetate, and the organic layer was evaporated to give a combined dry residue required for HPLC analysis.

#### 4.2.13 Reaction of Resveratrol with K_3_[Fe­(CN)_6_] and Na_2_CO_3_ in Acetonitrile (Ox13)

As described by Gülşen et al.,^72^ to a solution of resveratrol (11.4 mg/4 mL in acetonitrile), an aqueous solution of potassium ferricyanide (16.5 mg/0.5 mL) and an aqueous solution of sodium carbonate (5.3 mg/0.5 mL) were added, and the mixture was stirred for 43 h at room temperature. The reaction was stopped by adding an aqueous solution of reduced glutathione (15.35 mg/1.5 mL). The solvent was evaporated, and the residue partitioned between water and ethyl acetate. Dry residue of the combined organic layers was purified by silica gel column chromatography eluted with acetone/hexane (1:1, v/v) and thereafter evaporated in vacuo to give a combined dry residue required for HPLC analysis.

#### 4.2.14 Reaction of Resveratrol with CuSO_4_ in Acetonitrile (Ox14)

Similarly to quercetin oxidation previously described,^73^ an aqueous solution of copper sulfate (13.98 mg/10 mL) was added to a solution of resveratrol (10 mg) in acetonitrile–water (10 mL; 9:1, v/v), and the mixture was stirred for 72 h at 37 °C. The reaction was stopped by adding an aqueous solution of reduced glutathione (26.93 mg/3.75 mL). The solvent was evaporated, and the residue was partitioned between water and ethyl acetate. Dry residue of the combined organic layers was purified by silica gel column chromatography eluted with acetone/hexane (1:1, v/v) and thereafter evaporated under a vacuum to obtain dry residue required for HPLC analysis.

#### 4.2.15 Reaction of Resveratrol with Xanthine Oxidase in Phosphate Buffer, pH 7.4 (Ox15)

This was done according to the XO inhibitory activity method reported in our previous study.^31^ Briefly, a DMSO solution of 50 μM resveratrol (10.25 mg/5 mL), 50 mM phosphate buffer (pH 7.5), 0.15 mM phosphate-buffered xanthine solution, and 5 μL of XO enzyme/mL phosphate buffer solution was prepared. To a 2.5 mL solution of 50 μM resveratrol, 70 mL of phosphate buffer solution, 50 mL of xanthine solution, and 12.5 mL of xanthine oxidase solution were added, and the mixture was stirred for 5 min at 37 °C. The reaction was cooled down in an ice bath and partitioned between water and ethyl acetate, and the organic layer was evaporated to give a combined dry residue required for HPLC analysis.

#### 4.2.16 Reaction of Resveratrol with H_2_O_2_ and 5,10,15,20-Tetrakis­(pentafluorophenyl)-21H,23H-porphyrin Iron­(III) Chloride (C_44_H_8_ClF_20_FeN_4_) in Acetonitrile–Methanol (Ox16)

A solution of porphyrin iron­(III) chloride (10.6 mg) dissolved in 36 mL of methanol/water (31:5, v/v) and 2.5 mL of hydrogen peroxide (160 mM) was added to an acetonitrile solution of resveratrol (11.48 mg/9 mL acetonitrile), and the mixture was stirred for 50 min at room temperature. The reaction was stopped by adding an aqueous solution of reduced glutathione (15.35 mg/5 mL). The solvent was evaporated under nitrogen, and the residue was partitioned between water and ethyl acetate. Dry residue of the combined organic layers was purified by silica gel column chromatography eluted with acetone/hexane (1:1, v/v) and thereafter evaporated in vacuo. Further purification was carried out on the C18 column with isocratic elution CH_3_CN/H_2_O (55:45, v/v) to remove remaining porphyrin oxidant in the mixture. The solvent was evaporated under vacuo, and the residue was subjected to HPLC analysis.

### 4.3 Metabolic Profile Analysis

#### 4.3.1 UHPLC-PDA-ELSD-MS Analysis

UHPLC analyses were carried out on the same instrument and under the same conditions as those described by Huber and co-workers.^61^ Analysis of the crude reaction mixtures was carried out on an ultrahigh-performance liquid chromatography system equipped with a PhotoDiode Array, an evaporative light-scattering detector, and a single quadrupole mass spectrometer detector using heated electrospray ionization (UHPLC-PDA-ELSD-MS) (Waters, Milford, MA, USA). The ESI parameters were the following: capillary voltage 800 V, cone voltage 15 V, source temperature 120 °C, and probe temperature 600 °C. The acquisition was done in positive or negative ionization mode with an *m*/*z* range of 150–1000 Da. The chromatographic separation was performed on an Acquity UPLC BEH C_18_ column (50 × 2.1 mm i.d., 1.7 μm; Waters, Milford, MA, USA) at 0.6 mL/min, 40 °C with H_2_O (A) and CH_3_CN (B) both containing 0.1% formic acid as solvents. The gradient was carried out as follows: 5–100% B in 7 min, 1 min at 100% B, and a re-equilibration step at 5% B for 2 min. The ELSD temperature was fixed at 45 °C with a gain of 9. The PDA data were acquired from 190 to 500 nm, with a resolution of 1.2 nm. The sampling rate was set at 20 points/s. All data were processed using MassLynx software (Waters, Milford, MA, USA).

#### 4.3.2 UHPLC-PDA-CAD-HRMS Analysis

UHPLC-PDA-CAD-HRMS analyses were carried according to previously described methods on the same instrument and under the same conditions.^61^ The pure compounds were analyzed on a Waters Acquity UHPLC system equipped with a Q-Exactive Focus mass spectrometer (Thermo Scientific, Bremen, Germany), using a heated electrospray ionization source (HESI-II). The chromatographic separation was carried out on an Acquity UPLC BEH C_18_ column (50 × 2.1 mm i.d., 1.7 μm; Waters) at 0.6 mL/min, 40 °C with H_2_O (A) and CH_3_CN (B) both containing 0.1% formic acid as solvents. The gradient was carried out as follows: 5 to 100% B in 7 min, 1 min at 100% B, and a re-equilibration step at 5% B in 2 min. The ionization parameters were the same as used by Rutz and co-workers.^74^

### 4.4 Structure Elucidation

Structure elucidation of the isolated compounds was based on their molecular formulas obtained by high-resolution mass spectrometry (HRMS) and nuclear magnetic resonance (NMR) studies. HRMS spectra were acquired on Q-Exactive Plus hybrid quadrupole-orbitrap mass spectrometer (Thermo Scientific, Waltham, MA, USA) equipped with a heated electrospray ionization (HESI-II) probe that was used in positive or negative mode per required (see Supporting Information, Figures S27–S33). ^1^H NMR, ^13^C, APT, HSQC, HMBC, ^1^H,^1^H–COSY, and NOESY were recorded in acetone-*d*_6_ at room temperature on a Bruker DRX-500 spectrometer. Chemical shifts (δ) are given on the δ-scale and referenced to the solvents (acetone-*d*_6_: δH = 2.05 and δC = 29.9 ppm); coupling constant (*J*) values are expressed in Hz (Supporting Information, Figures S33–S63). Full ^1^H and ^13^C signal assignment was performed by means of comprehensive one- and two-dimensional NMR methods using widely accepted strategies.

### 4.5 Enantiomer Separation by Chiral HPLC

Compound **6** racemate (5 mg) was separated into its enantiomers using an isocratic elution of *n*-hexane/EtOH (82:18, v/v) on cellulose tris­(3,5-dimethylphenylcarbamate) coated on silica gel (Chiralcel OD-H; 250 × 4.6 mm, 5 μm, Daicel, Japan) by chiral HPLC (PU-4086 and PU-4386 pumps; AS-4350 Plus autosampler; CO-4060 column oven; MD-4015 Plus PDA detector, Jasco Co., Ltd., Tokyo, Japan). Repeated 10 μL injections were performed at solutions of 2 mg/mL compound **6** to obtain pure enantiomers **6a** (1.89 mg) and **6b** (1.97 mg). The purity of the enantiomers was confirmed by injecting them into the chiral HPLC setup using the same chromatographic conditions. Absolute configuration determination and bioactivity analysis were performed on purified enantiomers.

### 4.6 Measurement of the Vibrational Circular Dichroism (VCD) Spectrum

The VCD spectrum of **6b** was recorded in DMSO-*d*_6_ (99.96 atom % D, Aldrich) solution at a resolution of 4 cm^–1^ using a Bruker PMA 37 VCD/PM-IRRAS module connected to an Equinox 55 FT-IR spectrometer (Bruker Optik GmbH, Ettlingen, Germany). The ZnSe photoelastic modulator of the instrument was set to 1400 cm^–1^ and an optical filter with a transmission range of 1830–800 cm^–1^ was used to optimize the instrument for the fingerprint region. The instrument was calibrated for the VCD intensity with a CdS multiple-wave plate. For the VCD measurement, a CaF_2_ cell of 0.207 mm path length and sample concentration of 10 mg/mL was used. The spectrum was averaged for 20 h (corresponding to ∼72,000 accumulated scans). Baseline correction was achieved by subtracting the VCD spectrum of the solvent recorded under the same conditions.

### 4.7 Molecular Modeling and Calculation of VCD Spectra

Geometry optimizations and the calculation of VCD spectra were performed for the (2*S*,3*S*) enantiomer with the Gaussian 16 software package^75^ at the B3LYP/6-311++G** level, using an IEF-PCM solvent model for DMSO. All possible coplanar arrangements of the five phenolic OH groups were considered and redundant structures, resulting from simultaneous rotation of the benzene rings and OH groups by 180°, were discarded. Two coplanar conformations (s-*cis* and s-*trans*) of the styryl substituent relative to the dihydrobenzofuran ring were obtained, while the two benzene rings, attached to the chiral centers, were found to be quasi-perpendicular to the five-membered heterocycle. A total number 64 conformers were found with populations ranging from 3.19 to 0.37%, considering a Boltzmann distribution at 298 K. The lowest-energy conformer is shown in Supporting Information, Figure S67. Theoretical VCD curves of individual conformers were simulated from the calculated wavenumber and rotatory strength data by using the Lorentzian band shape and a half-width at half-height value of 4 cm^–1^. Calculated frequencies were scaled by a factor of 0.985. The theoretical VCD curves were obtained as a population-weighted sum of the calculated spectra of all 64 conformers.

### 4.8 Bioactivity Studies of Reaction Mixtures and Pure Compounds

#### 4.8.1 Angiotensin-I-Converting Enzyme (ACE) Inhibitor Screening

Angiotensin-converting enzyme inhibitory activity of the oxidized mixtures and pure compounds was determined according to previously performed and reported method.^76^ Briefly, to 25 μL of samples diluted in methanol-assay buffer was added 25 μL of enzyme solution (each 25 μL containing 2.5 mU of the ACE enzyme). The solution was incubated for 5 min at 37 °C while being shaken, and then 50 μL of the substrate was added. Immediately after adding the substrate, the fluorescence was measured in kinetic mode at Ex/Em 290/450 nm for 5 min using a FluoStar Optima plate reader (BMG Labtech, Ortenberg, Germany). Dose–response studies on resveratrol and isolated compounds to determine their IC_50_ values for ACE activity, were conducted in a similar manner.

The percentage inhibition by each compound was calculated as

%inhibition=(values
without samples−sample values)/(values without samples)×100

To elucidate the inhibition mechanism, ACE inhibitory kinetic studies were conducted on the most potent compounds. Similarly to the percent inhibitory protocol, 25 μL of enzyme was added to plate wells containing 25 μL of several concentrations of the compounds: **6** (0, 5, 10, and 20 μM) and **12** (0, 7.5, 15, and 30 μM). After incubation for 5 min at 37 °C, 50 μL of the substrate, Abz-Gly-Phe­(NO_2_)-Pro, was added at different concentrations ranging from 125–500 μM to respective wells, and fluorescence was measured in kinetic mode at extinction values at Ex/Em 290/450 nm using the plate reader.

#### 4.8.2 ACE Domain-Specific Studies

ACE domain-specific inhibition studies were performed according to methods previously reported,^60,77^ with modifications as stated.^76^ The initial velocity of the ACE-catalyzed reaction was determined across a range of concentrations (1–128 μM) of the fluorescence resonance energy transfer (FRET) substrates (Abz-SDK­(Dnp)­P-OH and Abz-LFK­(Dnp)-OH for the N- and C-domain, respectively) at a constant enzyme concentration and in the absence of samples. To do this, 40 μL of assay buffer and 60 μL FRET substrate solutions were preincubated for 10 min at 37 °C, with the reaction started by adding 20 μL of diluted ACE solution (5 μL ACE enzyme + 15 μL 0.1 Tris buffer) and fluorescence measured at λ_ex_/λ_em_ = 290/450 nm every minute at 37 °C for 30 min.The FRET substrate *K*_M_ in this initial velocity study, determined using the Michaelis–Menten equation, was used as substrate concentration in subsequent domain-specific inhibitory studies.

To determine the inhibitory activity of compounds **6** and **12** in both domains, 40 μL inhibitor solutions and 60 μL FRET- substrate solutions were preincubated for 10 min at 37 °C, the reaction was started by adding 20 μL of diluted ACE solution and fluorescence monitored at λ_ex_/λ_em_ = 290/450 nm every minute at 37 °C for 30 min. Control samples, representing 100% enzyme activity, were prepared by substituting the inhibitor solution with a Tris buffer. All experiments were performed in triplicates. The ACE inhibitory activity was calculated using the following equation:

$$\left(\%\right)=\left(A_{b}-A_{a}\right)-\left(C_{b}-C_{a}\right)/\left(A_{b}-A_{a}\right)\times 100$$

where *A*_a_ and *A*_b_ are the absorbance of control wells at 0 and 15 min respectively, and *C*_a_ and *C*_b_ are the absorbance of the inhibitor wells at 0 and 15 min, respectively.

Dose–effect studies on compounds **6** and **12** using the FRET substrates were used to determine the IC_50_ of these compounds on both ACE domains, in a similar protocol to that described above for the percent inhibition of these compounds.

To determine the inhibition constant, domain-specific inhibitory kinetic studies were performed on **6**. Similar to the percent inhibitory protocol, 60 μL of several concentrations of Abz-LFK (7.5–120 μM) and Abz-SDK (20–200 μM) was added to plate wells containing 40 μL of several concentrations of compound **6** (0, 10, 20, and 40 μM) for the C-domain and (0, 30, 60, and 90 μM) for the N-domain, respectively. After incubation for 10 min at 37 °C, 20 μL of ACE was added to each well, and fluorescence was measured in kinetic mode at extinction values at Ex/Em 290/450 nm using the plate reader.

#### 4.8.3 ACE Molecular Docking Study

Molecular docking of **6a-(** *R*,*R* **)** and **6b-(** *S*,*S* **)** enantiomers in the active sites of ACE was done according to previously published method.^76^ This method follows the procedure outlined for selective ACE inhibitors^78^ using Autodock suite following the stepwise protocol by Forli and co-workers.^79^

The 2-D structure of both enantiomers was drawn using ChemDraw 12.0.2 software (ACD/LABORATORIES, Advanced Chemistry Development, Inc.), and the energy of **6a-(** *R*,*R* **)** and **6b-(** *S*,*S* **)** was minimized at the default mode, using a minimum RMS gradient of 0.010 in the software Chem3D Pro 12.0 (ACD/LABORATORIES, Advanced Chemistry Development, Inc.).

X-ray crystallographic structures of the C-domain and N-domain human angiotensin I-converting enzyme complexed with lisinopril were obtained from the RCSB Protein Data Bank (PDB ID: 1O86 and 2C6N, respectively).^56,62^ Prior to docking analysis, water molecules and the lisinopril ligand were removed from the 1O86 ACE protein model (C-domain) using the AutoDock 4.2 (The Scripps Research Institute, La Jolla, CA, USA), while the zinc and chlorine atoms were retained in the ACE protein model, as these have been reported to be essential for the activity of ACE.^62^ The final receptor for docking was obtained by adding polar hydrogens, merging nonpolar hydrogens, and Kollman charge using AutoDockTools. The PDB files for both the enzyme and compounds were converted to PDBQT format by using the AutoDock 4 graphical user. A zinc-centered grid box (*X*: 43.817, *Y*: 38.308, and *Z*: 46.652, covering 50 × 70 × 50 grid points of 0.375 Å spacing) was used to cover all active residues around the Zn­(II) prosthetic group to assess the inhibitor-active site interactions.^80^

Similar protein preparation was done on the 2C6N ACE protein model (N-domain), with the removal of the sugar moieties in addition to lisinopril and the water molecules. After preparing the protein and ligand as described above, the zinc-centered map for ACE 2C6N was calculated by highlighting amino acid residues that interact with two N-domain-selective inhibitors, RXP407 and its analogue 33RE, as reported by Douglas and co-workers.^65^ A grid box (*X*: −28.034, *Y*: −24.612, and *Z*: −33.992, with 70 × 70 × 60 grid points and 0.375 Å spacing) was defined to cover all active residues and the Zn­(II) heteroatom in the A chain of this domain.

The AutoDock 4.2 package was used for the docking simulation based on a Lamarckian genetic algorithm,^81^ with docking poses of the compound among 20 genetic algorithm runs and obtained at the medium level (2.5 × 10^6^). The binding energy values and the scores were used to evaluate the molecular docking and determine the best poses for the compound, with interaction visualization achieved via Biovia (Discovery Studio visualizer, Dassault Systèmes, version 21.1.0.20298) after conversion of the docked PDBQT files into PDB files using OpenBabel GUI software version 2.4.1).^82^

#### 4.8.4 Lipoxygenase (15-LOX) Inhibitory Activity Screening

15-LOX inhibitory activities of the compounds were determined using the Cayman’s lipoxygenase inhibitor screening assay kit (760700, Cayman Chemical, MI, USA), with a slight modification in the volume of concentration of linoleic acid substrate added to the wells. Briefly, in a 96-well white plate (655101, F-bottom, Grenier bio-one, Germany), 90 μL of lipoxygenase standard solution was added to 10 μL of assay buffer, 10 μL of NDGA-buffer solution, and 10 μL of sample solution (samples were dissolved in methanol and subsequently assay buffer until desired concentrations) to obtain negative control, positive control, and sample wells, respectively. After incubating for 10 min, 20 μL of arachidonic/NaOH solution was added to all wells, and the plate was placed on a shaker. The reaction was stopped after 5 min by adding 100 μL of chromogen to all wells, and the absorbance read at 485 nm. Dose–effect studies on the most bioactive compounds and resveratrol were used to determine the IC_50_ of the compounds on the LOX enzyme.

The percentage inhibition was calculated as;(IA−inhibitor)/IA×100

where IA = absorbance of the 100% initial activity wells (containing LOX and solvent used to dissolve the reaction mixtures) and inhibitor = absorbance of the inhibitor wells (containing LOX and samples).

#### 4.8.5 Cyclooxygenase (COX) Inhibitory Activity Screening

COX-1 and 2 inhibitory activities were tested based on the fluorometric method described in BioVision’s COX-1 inhibitor screening kit leaflet (K548-100, BioVision, CA, USA) and the COX-2 inhibitor screening kit leaflet (K547-100, BioVision, CA, USA), respectively. Sample solutions were prepared by dissolving in DMSO and subsequently buffer to obtain the desired concentrations. In a 96-well white plate (655101, F-bottom, Grenier bio-one, Germany), 80 μL of reaction mix (containing 76 μL of assay buffer, 1 μL of COX Probe, 2 μL of COX cofactor, and 1 μL of COX enzyme) was added to 10 μL of sample solution, DMSO and assay buffer to get test wells assigned for sample (S), negative control (N) and blank, respectively. Ten microliters of arachidonic/NaOH solution were added to each well using a multichannel pipette to initiate the reaction at the same time, and the fluorescence of each well was measured kinetically at Ex/Em 550/610 nm, at 25 °C for 10 min using a plate reader. The COX inhibitory activity of SC560 and Celecoxib, standard inhibitors of COX-1 and COX-2 respectively, was also determined.

The change in fluorescence between two points, *t*_1_ and *t*_2_ was determined, and relative inhibition was calculated as follows:

%Inhibition=(change
of N−change of S)/change of N×100

All bioactivity data processing was performed using GraphPad Prism 8.0 (La Jolla, CA, USA). The sigmoidal dose–response model was obtained using the nonlinear regression model log­(inhibitor) vs variable slope to determine the IC_50_ values of the compounds. The Michaelis constant (*K*_M_) and maximal velocity (*V*_MAX_) of ACE were determined by Lineweaver–Burk plots using the Pharmacological and biochemistry transform and simple linear regression functions of GraphPad Prism 8.0. Statistical evaluation was done by one-way ANOVA using Dunnett’s multiple comparison test with the significance level set at 0.05.

## Supplementary Material













## Notes

HPLC-PDA fingerprint of oxidized product mixtures Ox1–16 (Figures S1–S8); metabolic profiles (MS data) of Ox1–16 (Figures S9–S24); 3-D metabolic profile charts of Ox1–16 in positive and negative mode (Figure S25); HRMS spectra of compounds **1**, **3**, **4**, **7**, **11**, **13**–**15**, and **17**–**19** (Figures S26–S32); characteristic 1-D and 2-D NMR spectra of compounds **1**, **3**, **4**, **7**, **11**, **13**–**15**, and **17**–**19** (Figures S33–S63); Lineweaver–Burk plots of compounds **6** and **12** as ACE inhibitors (Figures S64 and S65, respectively); preparative HPLC chromatogram of the chiral separation of racemate **6** (Figure S66); structure of the lowest-energy conformer of (2*S*, 3*S*) enantiomer of compound **6** (Figure S67), and binding energies obtained in the docking studies of enantiopure **6a** and **6b** on C-ACE and N-ACE (Tables S1 and S2); HPLC chromatograms of compounds **2**, **6**, **6a**-**(** *R*,*R* **)**, **6b**-**(** *S*,*S* **)**, and **12** (Figures S68–S70) (PDF)
Molecular formula strings of the compounds along with their pharmacological activity (CSV)
PDB files of ligand-ACE protein model, with **6a**-**(** *R*,*R* **)** docked in 1O86 for C-ACE (PDB)
PDB files of ligand-ACE protein model, with **6a**-**(** *R*,*R* **)** docked in 2C6N for N-ACE (PDB)
PDB files of ligand-ACE protein model, with **6b**-**(** *S*,*S* **)** docked in 1O86 for C-ACE (PDB)
PDB files of ligand-ACE protein model, with **6b**-**(** *S*,*S* **)** docked in 2C6N for N-ACE (PDB)

## Abbreviations Used

^•^OH: hydroxyl radical
AAPH: 2,2′-Azobis­(2-amidinopropane) dihydrochloride
Abz-Gly-Phe­(NO_2_)-Pro: (2-Aminobenzoyl)-glycyl-(4-nitrophenylalanyl)-Proline
Abz- LFK­(Dnp)-OH: 2-Aminobenzoyl-leucine-phenylalanine-lysine­(dinitrophenyl)-hydroxyl
Abz-SDK­(Dnp)­P-OH: 2-Aminobenzoyl-Ser-Asp-Lys­(dinitrophenyl)-Pro-hydroxyl
ACD: Advanced Chemistry Development
APT: attached proton test (^13^C NMR)
BEH: bridged ethyl hybrid
BPPb: bradykinin potentiating peptide B
C-ACE: ACE C-terminal domain
CAD: charged aerosol detection
CH_3_CN: acetonitrile
CH_3_OH: methanol
CVDs: cardiovascular diseases
CyP1A2: cytochrome P450 1A2
Da: Dalton
ELSD: Evaporative light - scattering detector
EtOH: ethanol
Ex/Em: extinction/emission
GSH: reduced glutathione
H_2_O: water
H_2_O_2_: hydrogen peroxide
HESI-II: heated electrospray ionization source
IA: initial activity
IEF-PCM: integral equation formalism-polarizable continuum model
Inh.: inhibition
kcal: kilocalorie
LLE: ligand-lipophilicity efficiency
log *D*_7.4_: logarithm of the distribution coefficient at pH 7.4
LOX: lipoxygenase
mg: milligram
N-ACE: ACE N-terminal
NDGA: Nordihydroguaiaretic Acid
O_2_^•–^: superoxide anion radical
ONOO^–^: peroxynitrite
ONOOH: peroxynitrous acid
PCM: polarizable continuum model
PDBQT: protein data bank, autoDock format
PIDA: iodobenzene diacetate
PIFA: iodobenzene bis­(trifluoroacetate)
PM-IRRAS: polarization modulation-infrared reflection absorption spectroscopy
Ox: oxidized
PDA: photodiode array
PGE2: prostaglandin
ROS: reactive oxygen species
RNS: reactive nitrogen species
SEM: standard error of the mean
TPSA: topological polar surface area
TxA_2_: thromboxane A2
*V*_MAX_: maximal velocity
XO: xanthine oxidase

## References

[1] Renaud S., de Lorgeril M. (1992). Wine, alcohol, platelets, and the French paradox for
coronary heart disease. Lancet.

[2] Ronksley P. E., Brien S. E., Turner B. J., Mukamal K. J., Ghali W. A. (2011). Association
of alcohol consumption with selected cardiovascular disease outcomes:
a systematic review and meta-analysis. Br. Med.
J..

[3] Castaldo L., Narváez A., Izzo L., Graziani G., Gaspari A., Di Minno G., Ritieni A. (2019). Red wine consumption and cardiovascular
health. Molecules.

[4] Raj P., Aloud B. M., Louis X. L., Yu L., Zieroth S., Netticadan T. (2016). Resveratrol is equipotent to perindopril
in attenuating
post-infarct cardiac remodeling and contractile dysfunction in rats. J. Nutr. Biochem..

[5] Jang I.-A., Kim E. N., Lim J. H., Kim M. Y., Ban T. H., Yoon H. E., Park C. W., Chang Y. S., Choi B. S. (2018). Effects
of resveratrol on the renin-angiotensin system in the aging kidney. Nutrients.

[6] Gal R., Deres L., Toth K., Halmosi R., Habon T. (2021). The Effect
of Resveratrol on the Cardiovascular System from Molecular Mechanisms
to Clinical Results. Int. J. Mol. Sci..

[7] Cho S., Namkoong K., Shin M., Park J., Yang E., Ihm J., Thu V. T., Kim H. K., Han J. (2017). Cardiovascular protective
effects and clinical applications of resveratrol. J. Med. Food.

[8] Ramalingam L., Menikdiwela K., LeMieux M., Dufour J. M., Kaur G., Kalupahana N., Moustaid-Moussa N. (2017). The renin angiotensin system, oxidative
stress and mitochondrial function in obesity and insulin resistance. Biochim. Biophys. Acta, Mol. Basis Dis..

[9] Shi X., Guan Y., Jiang S., Li T., Sun B., Cheng H. (2019). Renin-angiotensin system inhibitor
attenuates oxidative stress induced
human coronary artery endothelial cell dysfunction via the PI3K/AKT/mTOR
pathway. Arch. Med. Sci..

[10] Hitomi H., Kiyomoto H., Nishiyama A. (2007). Angiotensin
II and oxidative stress. Curr. Opin. Cardiol..

[11] Patten G. S., Abeywardena M. Y., Bennett L. E. (2016). Inhibition of angiotensin converting
enzyme, angiotensin II receptor blocking, and blood pressure lowering
bioactivity across plant families. Crit. Rev.
Food Sci. Nutr..

[12] Dandona P., Dhindsa S., Ghanim H., Chaudhuri A. (2007). Angiotensin
II and inflammation: the effect of angiotensin-converting enzyme inhibition
and angiotensin II receptor blockade. J. Hum.
Hypertens..

[13] Xia N., Förstermann U., Li H. (2014). Resveratrol and endothelial nitric
oxide. Molecules.

[14] Baur J. A., Sinclair D. A. (2006). Therapeutic potential
of resveratrol: the in vivo evidence. Nat. Rev.
Drug Discovery.

[15] Chu H., Li H., Guan X., Yan H., Zhang X., Cui X., Li X., Cheng M. (2018). Resveratrol protects late endothelial progenitor cells
from TNF-α-induced inflammatory damage by upregulating Krüppel-like
factor-2. Mol. Med. Rep..

[16] Li H., Xia N., Förstermann U. (2012). Cardiovascular effects and molecular
targets of resveratrol. Nitric Oxide.

[17] Pannu N., Bhatnagar A. (2019). Resveratrol: from enhanced biosynthesis and bioavailability
to multitargeting chronic diseases. Biomed.
Pharmacother..

[18] Jia Z., Zhu H., Misra B. R., Mahaney J. E., Li Y., Misra H. P. (2008). EPR studies
on the superoxide-scavenging capacity of the nutraceutical resveratrol. Mol. Cell. Biochem..

[19] Bauer G. (2016). The antitumor
effect of singlet oxygen. Anticancer Res..

[20] Hayyan M., Hashim M. A., AlNashef I. M. (2016). Superoxide ion: generation and chemical
implications. Chem. Rev..

[21] Bauer G. (2018). HOCl and the
control of oncogenesis. J. Inorg. Biochem..

[22] Nimse S. B., Pal D. (2015). Free radicals, natural
antioxidants, and their reaction mechanisms. RSC Adv..

[23] Martemucci G., Costagliola C., Mariano M., D’andrea L., Napolitano P., D’Alessandro A.
G. (2022). Free Radical Properties,
Source and Targets, Antioxidant Consumption and Health. Oxygen.

[24] Phaniendra A., Jestadi D. B., Periyasamy L. (2015). Free radicals: properties, sources,
targets, and their implication in various diseases. Indian J. Clin. Biochem..

[25] Ruskovska T., Maksimova V., Milenkovic D. (2020). Polyphenols in human nutrition: from
the in vitro antioxidant capacity to the beneficial effects on cardiometabolic
health and related inter-individual variability – an overview
and perspective. Br. J. Nutr..

[26] Hunyadi A. (2019). The mechanism(s)
of action of antioxidants: From scavenging reactive oxygen/nitrogen
species to redox signaling and the generation of bioactive secondary
metabolites. Med. Res. Rev..

[27] Hunyadi, A. ; Agbadua, O. G. ; Takács, G. ; Balogh, G. T. Scavengome of an Antioxidant. In Vitamins and Hormones; Litwack, G. , Ed.; Academic Press: Cambridge, MA, USA, 2023; Vol. 121, pp 81–108 10.1016/bs.vh.2022.09.003. 36707145

[28] Pavlinov I., Gerlach E. M., Aldrich L. N. (2019). Next generation
diversity-oriented
synthesis: a paradigm shift from chemical diversity to biological
diversity. Org. Biomol. Chem..

[29] Fási L., Di Meo F., Kuo C.-Y., Stojkovic Buric S., Martins A., Kúsz N., Béni Z., Dékány M., Balogh G. T., Pesic M. (2019). Antioxidant-Inspired
Drug Discovery: Antitumor Metabolite Is Formed in Situ from a Hydroxycinnamic
Acid Derivative upon Free-Radical Scavenging. J. Med. Chem..

[30] Fási L., Latif A. D., Zupkó I., Lévai S., Dékány M., Béni Z., Könczöl Á., Balogh G. T., Hunyadi A. (2020). AAPH or Peroxynitrite-Induced
Biorelevant
Oxidation of Methyl Caffeate Yields a Potent Antitumor Metabolite. Biomolecules.

[31] Agbadua O. G., Kúsz N., Berkecz R., Gáti T., Tóth G., Hunyadi A. (2022). Oxidized Resveratrol Metabolites
as Potent Antioxidants and Xanthine Oxidase Inhibitors. Antioxidants.

[32] Kohri S., Fujii H. (2013). 2,2′-Azobis (isobutyronitrile)-derived alkylperoxyl radical
scavenging activity assay of hydrophilic antioxidants by employing
EPR spin trap method. J. Clin. Biochem. Nutr..

[33] Yoshida Y., Itoh N., Saito Y., Hayakawa M., Niki E. (2004). Application
of water-soluble radical initiator, 2,2′-azobis-[2-(2-imidazolin-2-yl)
propane] dihydrochloride, to a study of oxidative stress. Free Radical Res..

[34] Balazinski M., Schmidt-Bleker A., Winter J., von Woedtke T. (2021). Peroxynitrous
Acid Generated In Situ from Acidified H2O2 and NaNO2. A Suitable Novel
Antimicrobial Agent?. Antibiotics.

[35] Takahama U., Yamamoto A., Hirota S., Oniki T. (2003). Quercetin-Dependent
Reduction of Salivary Nitrite to Nitric Oxide under Acidic Conditions
and Interaction between Quercetin and Ascorbic Acid during the Reduction. J. Agric. Food Chem..

[36] Panzella L., De Lucia M., Amalfitano C., Pezzella A., Evidente A., Napolitano A., d’Ischia M. (2006). Acid-Promoted Reaction of the Stilbene
Antioxidant Resveratrol with Nitrite Ions: Mild Phenolic Oxidation
at the 4‘-Hydroxystiryl Sector Triggering Nitration, Dimerization,
and Aldehyde-Forming Routes. J. Org. Chem..

[37] Liu N., Xu H., Sun Q., Yu X., Chen W., Wei H., Jiang J., Xu Y., Lu W. (2021). The role of oxidative
stress in hyperuricemia and xanthine oxidoreductase (XOR) inhibitors. Oxid. Med. Cell. Longevity.

[38] Forman H. J., Zhang H., Rinna A. (2009). Glutathione:
Overview of its protective
roles, measurement, and biosynthesis. Mol. Aspects
Med..

[39] Schmid R., Heuckeroth S., Korf A., Smirnov A., Myers O., Dyrlund T. S., Bushuiev R., Murray K. J., Hoffmann N., Lu M. (2023). Integrative analysis
of multimodal mass spectrometry
data in MZmine 3. Nat. Biotechnol..

[40] Wang Y., Wach J.-Y., Sheehan P., Zhong C., Zhan C., Harris R., Almo S. C., Bishop J., Haggarty S. J., Ramek A. (2016). Diversity-oriented
synthesis as a strategy for fragment
evolution against GSK3β. ACS Med. Chem.
Lett..

[41] Kidd S. L., Osberger T. J., Mateu N., Sore H. F., Spring D. R. (2018). Recent
Applications of Diversity-Oriented Synthesis Toward Novel, 3-Dimensional
Fragment Collections. Front. Chem..

[42] Shingai Y., Fujimoto A., Nakamura M., Masuda T. (2011). Structure and function
of the oxidation products of polyphenols and identification of potent
lipoxygenase inhibitors from Fe-catalyzed oxidation of resveratrol. J. Agric. Food Chem..

[43] Lee D., Bhat K. P. L., Fong H. H. S., Farnsworth N. R., Pezzuto J. M., Kinghorn A. D. (2001). Aromatase
Inhibitors from Broussonetia
papyrifera. J. Nat. Prod..

[44] Li S. Y., Fuchino H., Kawahara N., Sekita S., Satake M. (2002). New Phenolic
Constituents from Smilax bracteata. J. Nat.
Prod..

[45] Velu S. S., Di Meo F., Trouillas P., Sancho-Garcia J.-C., Weber J.-F. F. (2013). Regio-and stereocontrolled synthesis of oligostilbenoids:
Theoretical highlights at the supramolecular level. J. Nat. Prod..

[46] Predict Molecular Properties | Percepta Software. ACD/Labs. https://www.acdlabs.com/products/percepta-platform/ (accessed July 04, 2023).

[47] Lipinski C. A., Lombardo F., Dominy B. W., Feeney P. J. (2012). Experimental and
computational approaches to estimate solubility and permeability in
drug discovery and development settings. Adv.
Drug Delivery Rev..

[48] Ertl, P. ; R, B. ; Selzer, P. Calculation of molecular polar surface area as a sum of fragment-based contributions and its application to the prediction of drug transport properties. In Rational Approaches to Drug Design; Höltje, H.-D. S. W. , Ed.; Prous: Barcelona, 2001; pp 451–455 10.1021/jm000942e.

[49] Chang T. K., Chen J., Lee W. B. (2001). Differential
Inhibition and Inactivation
of Human CYP1 Enzymes bytrans-Resveratrol: Evidence for Mechanism-Based
Inactivation of CYP1A2. J. Pharmacol. Exp. Ther..

[50] Leeson P. D. (2016). Molecular
inflation, attrition and the rule of five. Adv.
Drug Delivery Rev..

[51] Leeson P. D., Bento A. P., Gaulton A., Hersey A., Manners E. J., Radoux C. J., Leach A. R. (2021). Target-based evaluation
of “drug-like”
properties and ligand efficiencies. J. Med.
Chem..

[52] Royster, R. L. ; Groban, L. ; Locke, A. Q. ; Morris, B. N. ; Slaughter, T. F. Chapter 8 - Cardiovascular Pharmacology. In Kaplan’s Essentials of Cardiac Anesthesia, Second ed.; Kaplan, J. A. , Ed.; Elsevier, 2018; pp 132–166 10.1016/B978-0-323-49798-5.00008-5.

[53] Tanaka M., Umemoto S., Kawahara S., Kubo M., Itoh S., Umeji K., Matsuzaki M. (2005). Angiotensin
II Type 1 Receptor Antagonist
and Angiotensin-Converting Enzyme Inhibitor Altered the Activation
of Cu/Zn-Containing Superoxide Dismutase in the Heart of Stroke-Prone
Spontaneously Hypertensive Rats. Hypertens.
Res..

[54] Münzel T., Keaney Jr J. F. (2001). Are ACE inhibitors a “magic bullet” against
oxidative stress?. Circulation.

[55] Kim J. H., Kim H., Kim Y. H., Chung W.-S., Suh J. K., Kim S. J. (2013). Antioxidant
effect of captopril and enalapril on reactive oxygen species-induced
endothelial dysfunction in the rabbit abdominal aorta. Korean J. Thorac. Cardiovasc. Surg..

[56] Corradi H. R., Schwager S. L., Nchinda A. T., Sturrock E. D., Acharya K. R. (2006). Crystal
structure of the N domain of human somatic angiotensin I-converting
enzyme provides a structural basis for domain-specific inhibitor design. J. Mol. Biol..

[57] Fuchs S., Xiao H. D., Hubert C., Michaud A., Campbell D. J., Adams J. W., Capecchi M. R., Corvol P., Bernstein K. E. (2008). Angiotensin-converting
enzyme C-terminal catalytic domain is the main site of angiotensin
I cleavage in vivo. Hypertension.

[58] Watermeyer J. M., Kroeger W. L., O’Neill H. G., Sewell B. T., Sturrock E. D. (2010). Characterization
of domain-selective inhibitor binding in angiotensin-converting enzyme
using a novel derivative of lisinopril. Biochem.
J..

[59] Cotton J., Hayashi M. A., Cuniasse P., Vazeux G., Ianzer D., De Camargo A. C., Dive V. (2002). Selective inhibition of the C-domain
of angiotensin I converting enzyme by bradykinin potentiating peptides. Biochemistry.

[60] Lunow D., Kaiser S., Rückriemen J., Pohl C., Henle T. (2015). Tryptophan-containing
dipeptides are C-domain selective inhibitors of angiotensin converting
enzyme. Food Chem..

[61] Huber R., Marcourt L., Quiros-Guerrero L.-M., Luscher A., Schnee S., Michellod E., Ducret V., Kohler T., Perron K., Wolfender J.-L. (2022). Chiral Separation of Stilbene Dimers Generated
by Biotransformation for Absolute Configuration Determination and
Antibacterial Evaluation. Front. Chem..

[62] Natesh R., Schwager S. L., Sturrock E. D., Acharya K. R. (2003). Crystal structure
of the human angiotensin-converting enzyme–lisinopril complex. Nature.

[63] Wei L., Alhenc-Gelas F., Corvol P., Clauser E. (1991). The two homologous
domains of human angiotensin I-converting enzyme are both catalytically
active. J. Biol. Chem..

[64] Georgiadis D., Cuniasse P., Cotton J., Yiotakis A., Dive V. (2004). Structural
determinants of RXPA380, a potent and highly selective inhibitor of
the angiotensin-converting enzyme C-domain. Biochemistry.

[65] Douglas R. G., Sharma R. K., Masuyer G., Lubbe L., Zamora I., Acharya K. R., Chibale K., Sturrock E. D. (2014). Fragment-based design
for the development of N-domain-selective angiotensin-1-converting
enzyme inhibitors. Clin. Sci..

[66] Montecucco F., Pende A., Mach F. (2009). The renin-angiotensin system modulates
inflammatory processes in atherosclerosis: evidence from basic research
and clinical studies. Mediators Inflammation.

[67] da
Cunha V., Tham D. M., Martin-McNulty B., Deng G., Ho J. J., Wilson D. W., Rutledge J. C., Vergona R., Sullivan M. E., Wang Y.-X. J. (2005). Enalapril attenuates
angiotensin II-induced atherosclerosis and vascular inflammation. Atherosclerosis.

[68] Kayama Y., Minamino T., Toko H., Sakamoto M., Shimizu I., Takahashi H., Okada S., Tateno K., Moriya J., Yokoyama M. (2009). Cardiac 12/15 lipoxygenase–induced inflammation
is involved in heart failure. J. Exp. Med..

[69] Kanduja K. L., Hardwaj A., Kaushik G. (2004). Resveratrol
inhibits N-nitrosodiethylamine-induced
ornithine decarboxylase and cyclooxygenase in mice. J. Nutr. Sci. Vitaminol..

[70] Wang Z., Huang Y., Zou J., Cao K., Xu Y., Wu J. M. (2002). Effects of red wine and wine polyphenol
resveratrol on platelet aggregation
in vivo and in vitro. Int. J. Mol. Med..

[71] Harikumar K. B., Aggarwal B. B. (2008). Resveratrol: a multitargeted
agent for age-associated
chronic diseases. Cell Cycle.

[72] Gülşen A., Makris D. P., Kefalas P. (2007). Biomimetic oxidation of quercetin:
Isolation of a naturally occurring quercetin heterodimer and evaluation
of its in vitro antioxidant properties. Food
Res. Int..

[73] Gülşen A., Turan B., Makris D. P., Kefalas P. (2007). Copper­(II)-mediated
biomimetic oxidation of quercetin: generation of a naturally occurring
oxidation product and evaluation of its in vitro antioxidant properties. Eur. Food Res. Technol..

[74] Rutz A., Dounoue-Kubo M., Ollivier S., Bisson J., Bagheri M., Saesong T., Ebrahimi S. N., Ingkaninan K., Wolfender J.-L., Allard P.-M. (2019). Taxonomically Informed Scoring Enhances
Confidence in Natural Products Annotation. Front.
Plant Sci..

[75] Frisch, M. J. ; Trucks, G. W. ; Schlegel, H. B. ; Scuseria, G. E. Gaussian 16 Rev. C.01, Wallingford, CT, 2016.

[76] Dávid C. Z., Kúsz N., Agbadua O. G., Berkecz R., Kincses A., Spengler G., Hunyadi A., Hohmann J., Vasas A. (2024). Phytochemical
Investigation of Carex praecox Schreb. and ACE-Inhibitory Activity
of Oligomer Stilbenes of the Plant. Molecules.

[77] Carmona A. K., Schwager S. L., Juliano M. A., Juliano L., Sturrock E. D. (2006). A continuous
fluorescence resonance energy transfer angiotensin I-converting enzyme
assay. Nat. Protoc..

[78] Caballero J. (2020). Considerations
for Docking of Selective Angiotensin-Converting Enzyme Inhibitors. Molecules.

[79] Forli S., Huey R., Pique M. E., Sanner M. F., Goodsell D. S., Olson A. J. (2016). Computational protein–ligand
docking and virtual
drug screening with the AutoDock suite. Nat.
Protoc..

[80] Xie D., Du L., Lin H., Su E., Shen Y., Xie J., Wei D. (2022). In vitro-in silico screening strategy and mechanism of angiotensin
I-converting enzyme inhibitory peptides from α-lactalbumin. LWT-Food Sci. Technol..

[81] Morris G. M., Huey R., Lindstrom W., Sanner M. F., Belew R. K., Goodsell D. S., Olson A. J. (2009). AutoDock4
and AutoDockTools4: Automated
docking with selective receptor flexibility. J. Comput. Chem..

[82] O’Boyle N. M., Banck M., James C. A., Morley C., Vandermeersch T., Hutchison G. R. (2011). Open Babel:
An open chemical toolbox. J. Cheminf..

