# Supplementary material for: New Insights into the French Paradox: Free Radical Scavenging by Resveratrol Yields Cardiovascular Protective Metabolites
Source: J Med Chem. 2025 May 7;68(10):10031–47. doi: 10.1021/acs.jmedchem.4c03061 (PMC12105021; doi:10.1021/acs.jmedchem.4c03061)

Supporting information

New insights into the French paradox: Free radical scavenging by resveratrol yields cardiovascular protective metabolites

Orinamhe G. Agbadua,[†] *Norbert Kúsz,*[†] *Róbert Berkecz,*[‡] *Elemér Vass,*[§] *Antal Csámpai,*[§] *Gábor Tóth,*[‖] *György T. Balogh,*[⊥,††,#] *Laurence Marcourt,*[¶] *Jean-Luc Wolfender,*[¶] *Emerson Ferreira Queiroz,*[¶] *and Attila Hunyadi*[†,∇,○,*]

[†] Institute of Pharmacognosy, University of Szeged, Eötvös str. 6, H-6720, Szeged, Hungary; orinamhe.agbadua@uniben.edu (O.G.A.); kusznorbert@gmail.com (N.K.)

[‡] Institute of Pharmaceutical Analysis, University of Szeged, Somogyi str. 4, H-6720 Szeged, Hungary; berkecz.robert@szte.hu (R.B.)

[§] Department of Organic Chemistry of Eötvös Loránd University, Budapest, Hungary; evass@elte.hu (E.V.), antal.csampai@ttk.elte.hu (A.C.)

[‖] NMR Group, Department of Inorganic and Analytical Chemistry, Budapest University of Technology and Economics, H-1111 Budapest, Hungary; drtothgabor@t-online.hu

[⊥] Department of Pharmaceutical Chemistry, Semmelweis University, H-1092 Budapest, Hungary; balogh.gyorgy.tibor@semmelweis.hu (G.T.B)

[††] Center for Pharmacology and Drug Research & Development, Semmelweis University, H-1085 Budapest, Hungary

[#] Department of Chemical and Environmental Process Engineering, Budapest University of Technology and Economics, H-1111 Budapest, Hungary

[¶] Institute of Pharmaceutical Sciences of Western Switzerland, University of Geneva, CMU, 1211 Geneva, Switzerland;laurence.marcourt@unige.ch (L.M.), jean-luc.wolfender@Unige.ch (J.L.W.), emerson.ferreira@unige.ch (E.F.Q.)

[∇] HUN-REN-SZTE Biologically Active Natural Products Research Group, Eötvös str. 6, H-6720 Szeged, Hungary

[○] Graduate Institute of Natural Products, Shih-Chuan 1st Rd. 100, Kaohsiung 807, Taiwan

***Correspondence:** hunyadi.attila@szte.hu; Tel.: +3662545557

Table of contents **Page**

Figure S1. HPLC-PDA fingerprint of oxidized product mixture Ox1and Ox2 S4
Figure S2. HPLC-PDA fingerprint of oxidized product mixture Ox3 and Ox4 S5
Figure S3. HPLC-PDA fingerprint of oxidized product mixture Ox5 and Ox6 S6
Figure S4. HPLC-PDA fingerprint of oxidized product mixture Ox7 and Ox8 S7
Figure S5. HPLC-PDA fingerprint of oxidized product mixture Ox9 and Ox10 S8
Figure S6. HPLC-PDA fingerprint of oxidized product mixture Ox11 and Ox12 S9
Figure S7. HPLC-PDA fingerprint of oxidized product mixture Ox13 and Ox14 S10
Figure S8. HPLC-PDA fingerprint of oxidized product mixture Ox15 and Ox16 S11
Figure S9. Metabolic profile (MS Data) of oxidized product mixture Ox1 S12
Figure S10. Metabolic profile (MS Data) of oxidized product mixture Ox2 S13
Figure S11. Metabolic profile (MS Data) of oxidized product mixture Ox3 S14
Figure S12. Metabolic profile (MS Data) of oxidized product mixture Ox4 S15
Figure S13. Metabolic profile (MS Data) of oxidized product mixture Ox5 S16
Figure S14 Metabolic profile (MS Data) of oxidized product mixture Ox6 S17
Figure S15. Metabolic profile (MS Data) of oxidized product mixture Ox7 S18
Figure S16 Metabolic profile (MS Data) of oxidized product mixture Ox8 S19
Figure S17. Metabolic profile (MS Data) of oxidized product mixture Ox9 S20
Figure S18. Metabolic profile (MS Data) of oxidized product mixture Ox10 S21
Figure S19. Metabolic profile (MS Data) of oxidized product mixture Ox11 S22
Figure S20. Metabolic profile (MS Data) of oxidized product mixture Ox12 S23
Figure S21. Metabolic profile (MS Data) of oxidized product mixture Ox13 S24
Figure S22. Metabolic profile (MS Data) of oxidized product mixture Ox14 S25
Figure S23. Metabolic profile (MS Data) of oxidized product mixture Ox15 S26
Figure S24. Metabolic profile (MS Data) of oxidized product mixture Ox16 S27
Figure S25. 3-D Metabolic profile chart of Ox1-Ox16 (Positive mode) and Negative mode S28
Figure S26. Compound **1**, HRMS (positive mode) and Compound **3**, HRMS (positive mode) S29
Figure S27. Compound **4**, HRMS (negative mode) and Compound **7**, HRMS (positive mode) S30
Figure S28. Compound **11**, HRMS (negative mode) S31
Figure S29. Compound **13**,HRMS (negative mode) S32
Figure S30. Compound **14**, HRMS (positive mode) and HRMS (negative mode) S33
Figure S31. Compound **15**, HRMS (negative mode) and Compound **17**, HRMS (positive mode) S34
Figure S32. Compound **18**, HRMS (negative mode) and Compound **19**, HRMS (negative mode) S35
Figure S33. Compound **1**, ^{1}H NMR and ^{13}C, APT NMR spectra S36
Figure S34. Compound **1**, HSQC and HMBC spectra S37
Figure S35. Compound **1**, COSY and NOESY spectra S38
Figure S36. Compound **3**, ^{1}H NMR and ^{13}C, APT NMR spectra S39
Figure S37. Compound **3**, HSQC and HMBC spectra S40
Figure S38. Compound **3**, COSY and NOESY spectra S41
Figure S39. Compound **4**, ^{1}H NMR and ^{13}C, APT NMR spectra S42
Figure S40. Compound **4**, HSQC and HMBC spectra S43
Figure S41. Compound **4**, COSY and NOESY spectra S44
Figure S42. Compound **7**, ^{1}H NMR and ^{13}C, APT NMR spectra S45
Figure S43. Compound **7**, HSQC and HMBC spectra S46
Figure S44. Compound **7**, COSY and NOESY spectra S47
Figure S45. Compound **11**, ^{1}H NMR and ^{13}C, APT NMR spectra S48
Figure S46. Compound **11**, HSQC and HMBC spectra S49
Figure S47. Compound **11**, COSY and NOESY spectra S50
Figure S48. Compound **13**, ^{1}H NMR and ^{13}C, APT NMR spectra S51
Figure S49. Compound **13**,HSQC, HMBC and COSY spectra S52
Figure S50. Compound **14**, ^{1}H NMR and ^{13}C, APT NMR spectra S53
Figure S51. Compound **14**, HSQC and HMBC spectra S54
Figure S52. Compound **14** COSY and NOESY spectra S55
Figure S53. Compound **15**, ^{1}H NMR and ^{13}C, APT NMR spectra S56
Figure S54. Compound **15**, HSQC and HMBC spectra S57

Table of contents **Page**

Figure S55. Compound **15**, COSY and NOESY spectra S58
Figure S56. Compound **17**, ^{1}H NMR and ^{13}C, APT NMR spectra S59
Figure S57. Compound **17**, HSQC, HMBC and COSY spectra S60
Figure S58. Compound **18**, ^{1}H NMR and ^{13}C, APT NMR spectra S61
Figure S59. Compound **18**, HSQC and HMBC spectra S62
Figure S60. Compound **18**, COSY and NOESY spectra S63
Figure S61. Compound **19**, ^{1}H NMR and ^{13}C, APT NMR spectra S64
Figure S62. Compound **19**, HSQC and HMBC spectra S65
Figure S63. Compound **19**, COSY and NOESY spectra S66
Figure S64. Lineweaver-Burk plot of compound **6** S67
Figure S65. Lineweaver-Burk plot of compound **12** S68
Figure S66. Chiral separation of **6** S69
Figure S67. Structure of the lowest-energy conformer of the (2S, 3S) enantiomer of compound **6** S70
Table S1. Binding energies of 20 ga runs on cACE (PDB: 1O86) by compound **6a-**(*R,R*) and **6b-**(*S,S*) S71
Table S2. Binding energies of 20 ga runs on nACE (PDB: 2C6N) by compound **6a-**(*R,R*) and **6b-**(*S,S*) S72
Figure S68. HPLC chromatogram of compound **6** at UV absorbance λ = 320 nm S73
Figure S69. HPLC chromatogram of compounds **6a**-(*R*,*R*) and **6b**-(*S*,*S*) at UV absorbance λ = 320 nm S74
Figure S70. HPLC chromatogram of compounds **2** and **12**, at UV absorbance λ = 320 nm S75

Figure S1. HPLC-PDA fingerprint of oxidized product mixture Ox1(above) and Ox2(below).

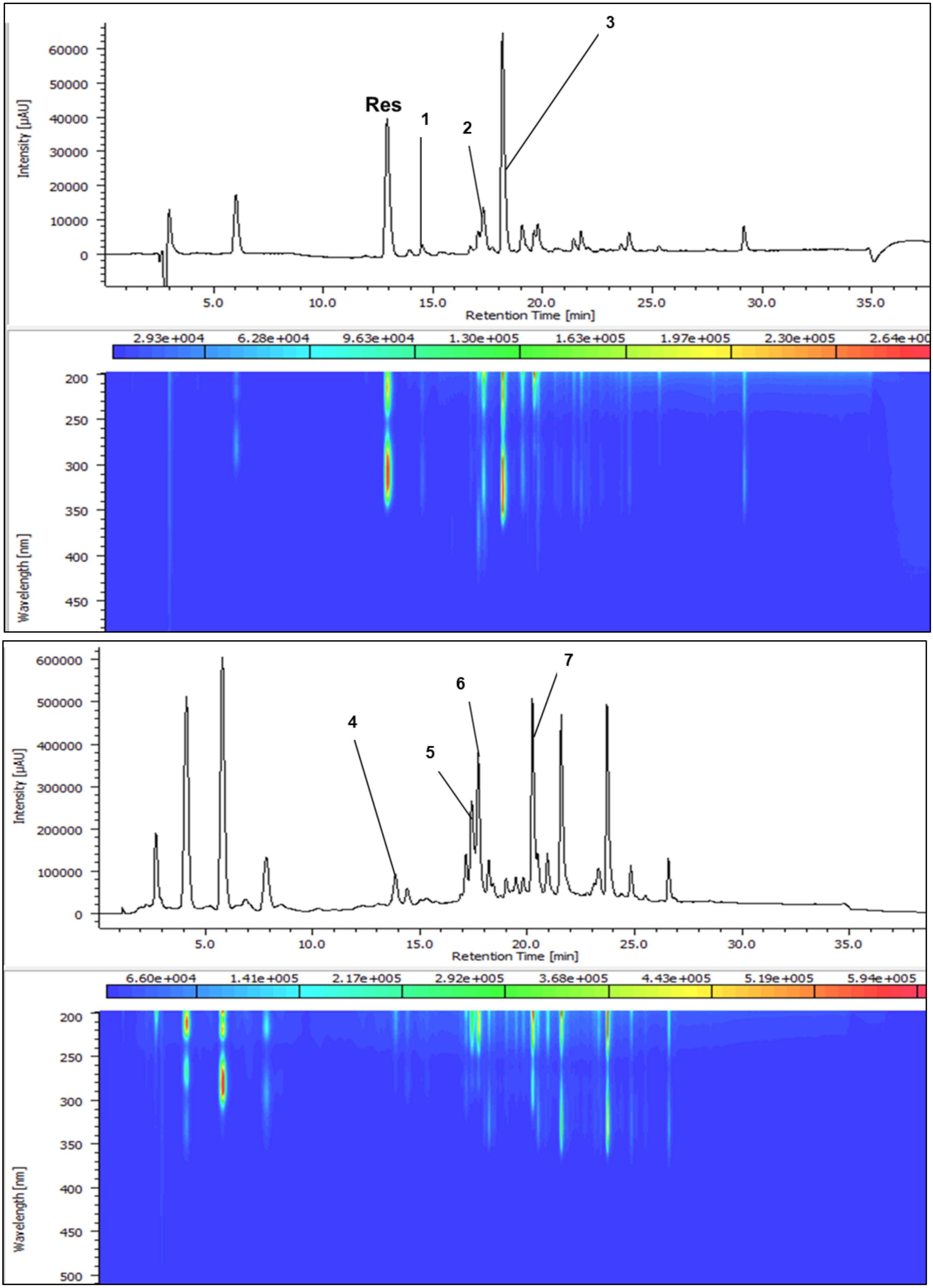

Figure S2. HPLC-PDA fingerprint of oxidized product mixture Ox3 (above) and Ox4 (below).

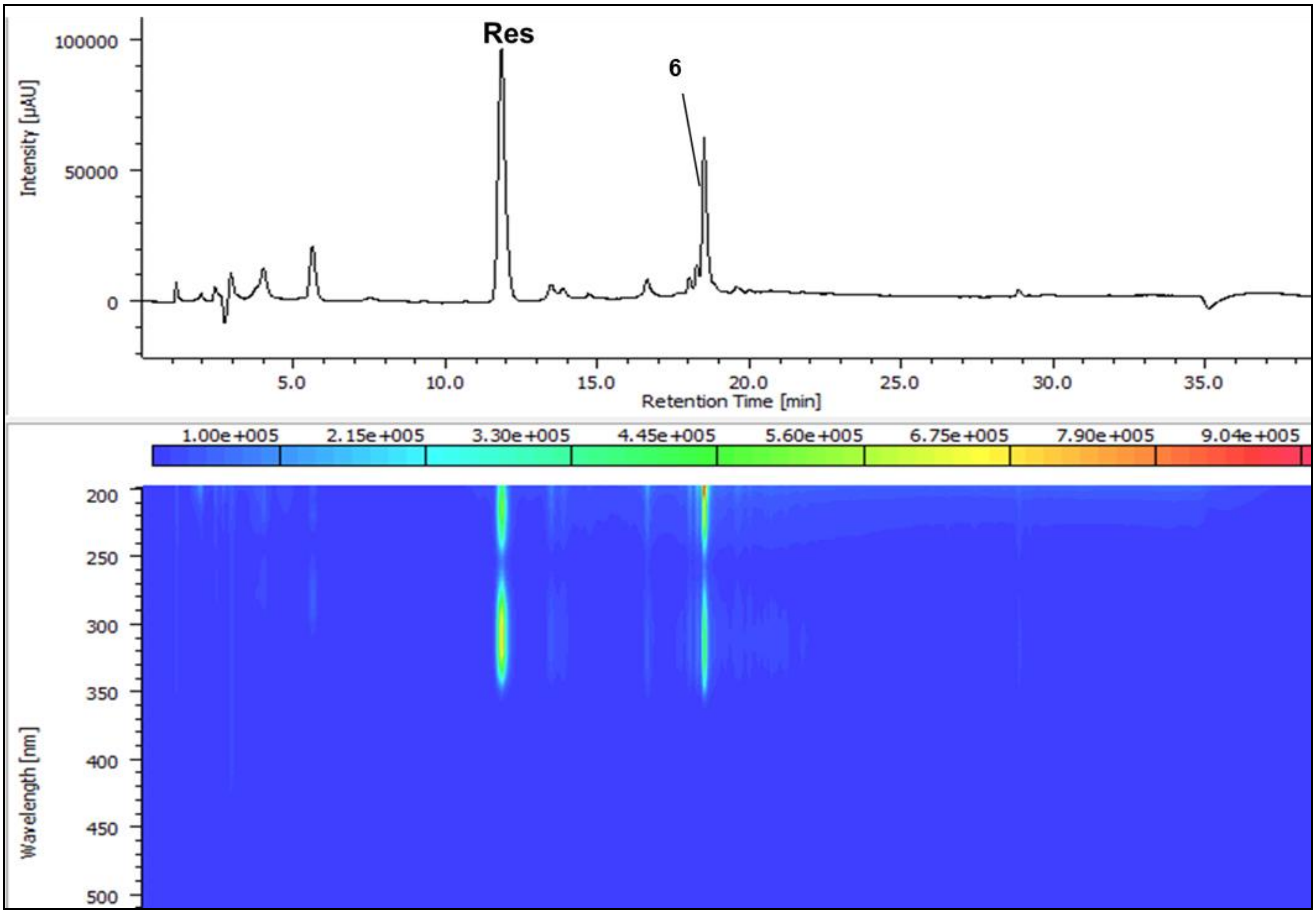

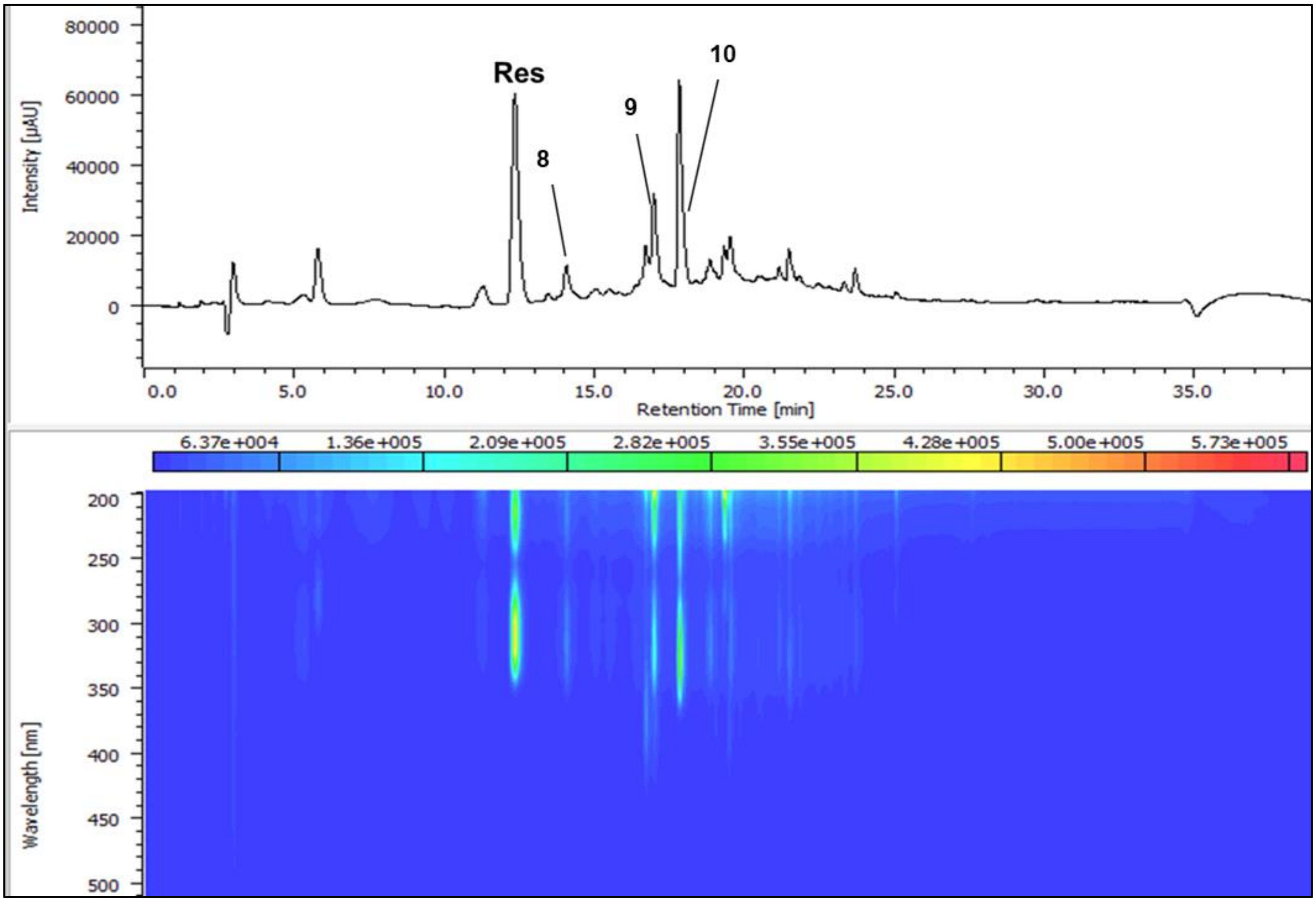

Figure S3. HPLC-PDA fingerprint of oxidized product mixture Ox5 (above) and Ox6 (below).

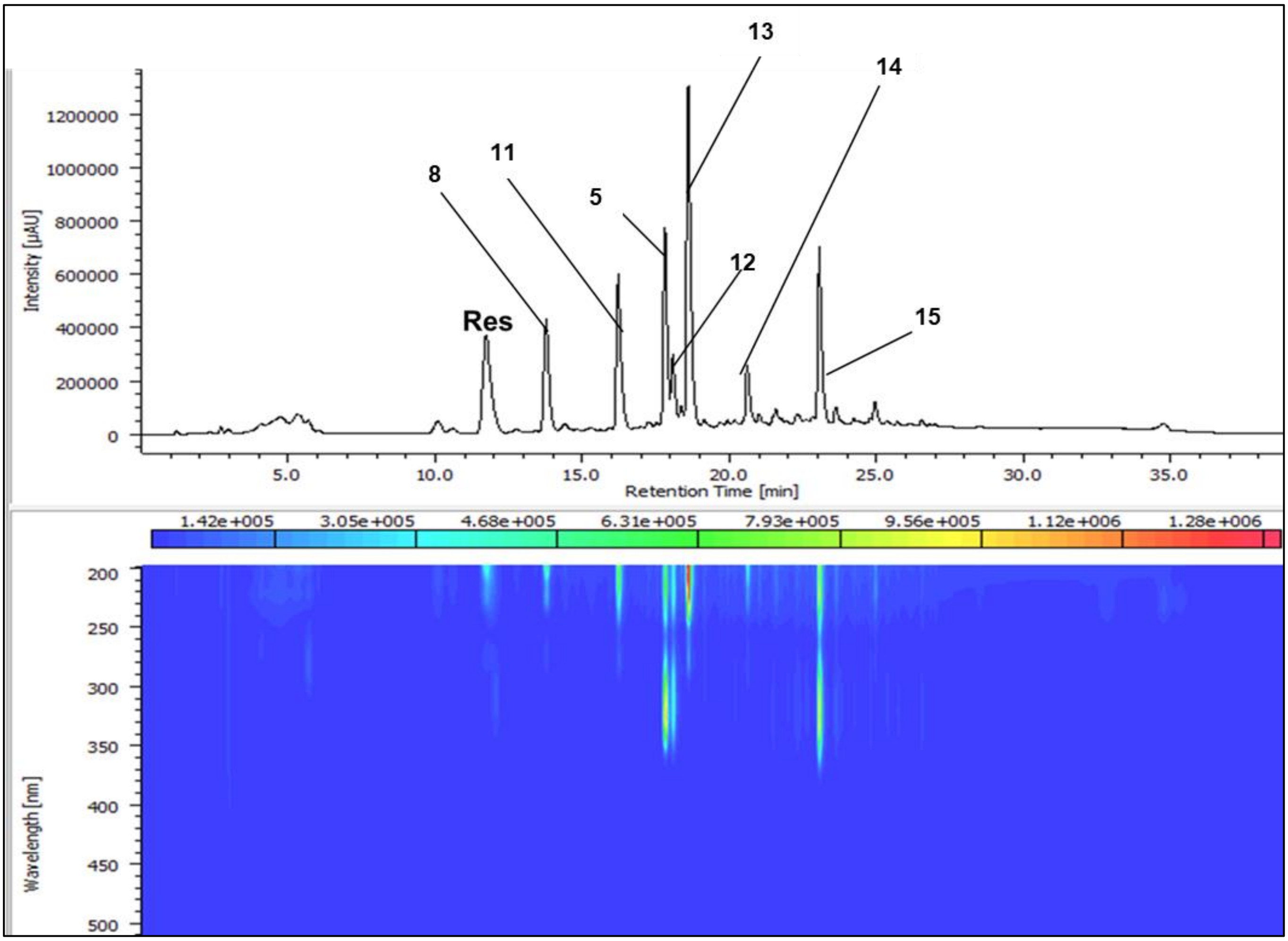

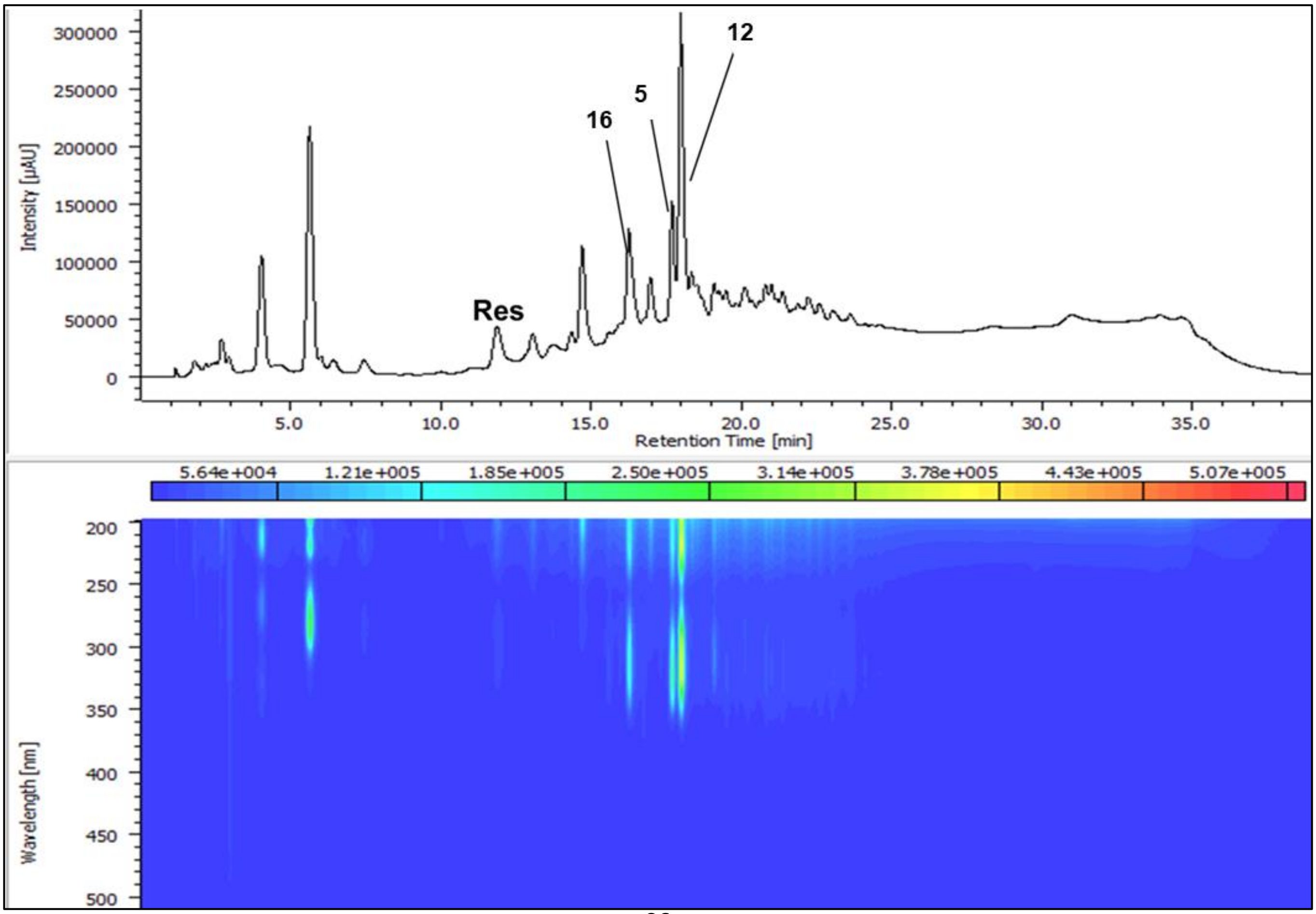

Figure S4. HPLC-PDA fingerprint of oxidized product mixture Ox7 (above) and Ox8 (below).

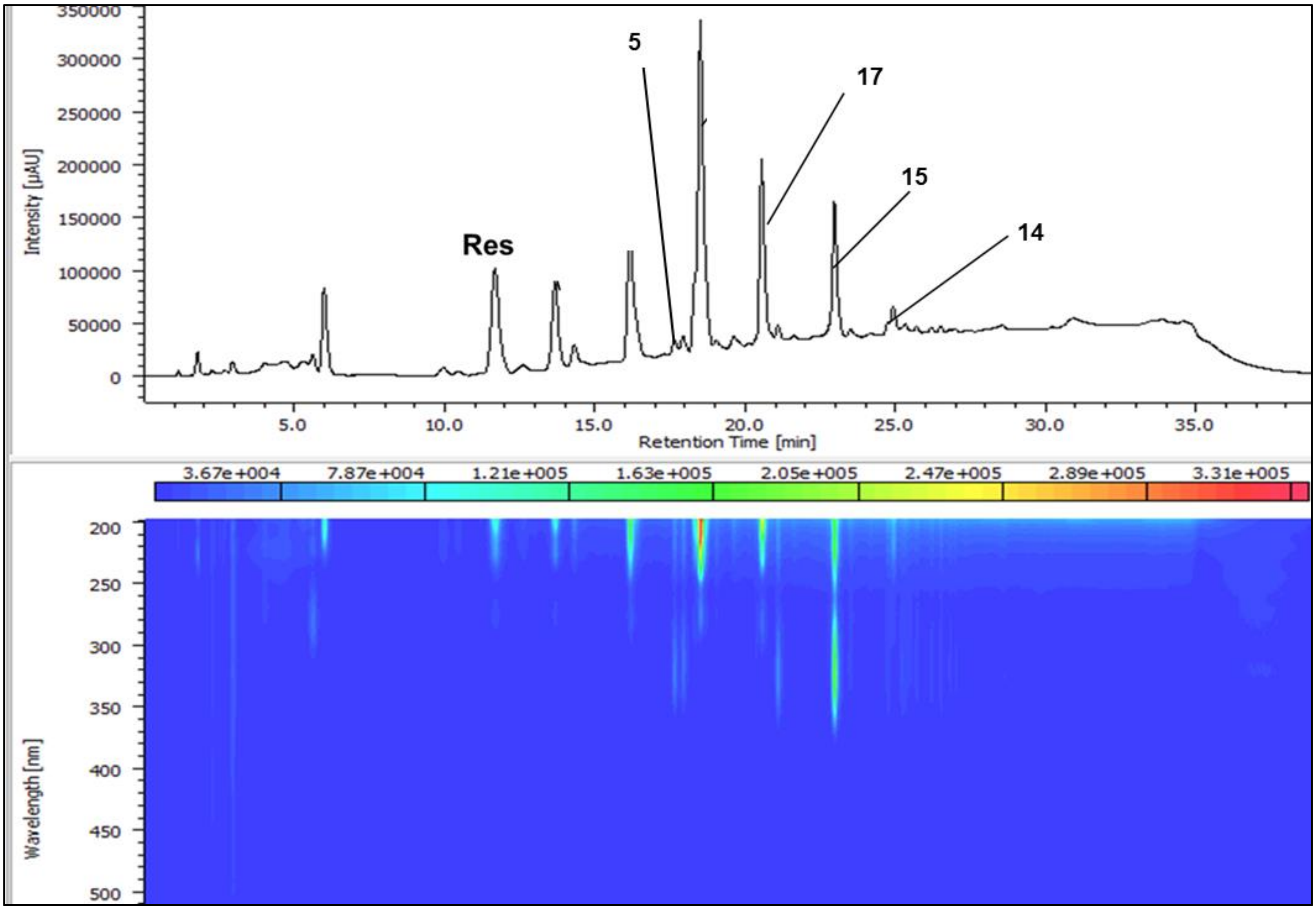

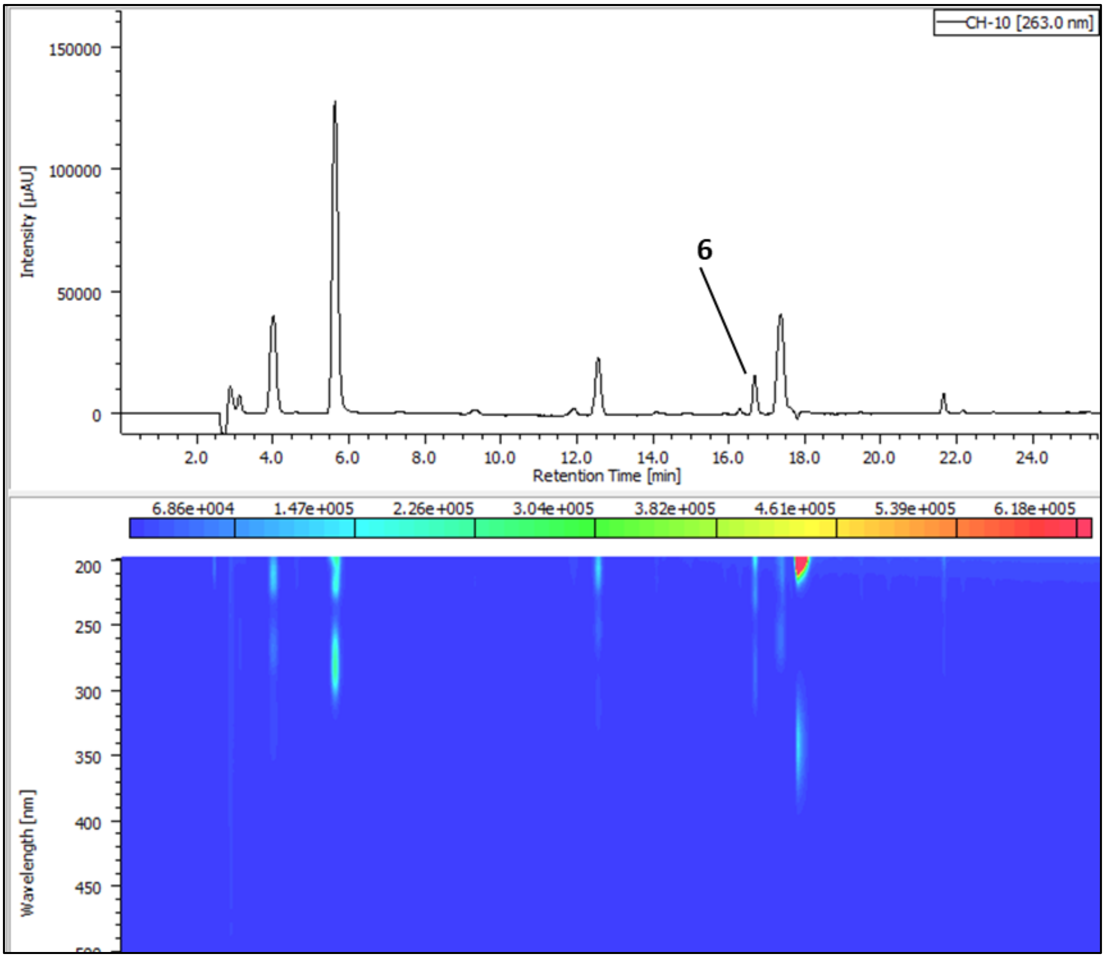

Figure S5. HPLC-PDA fingerprint of oxidized product mixture Ox9 (above) and Ox10 (below).

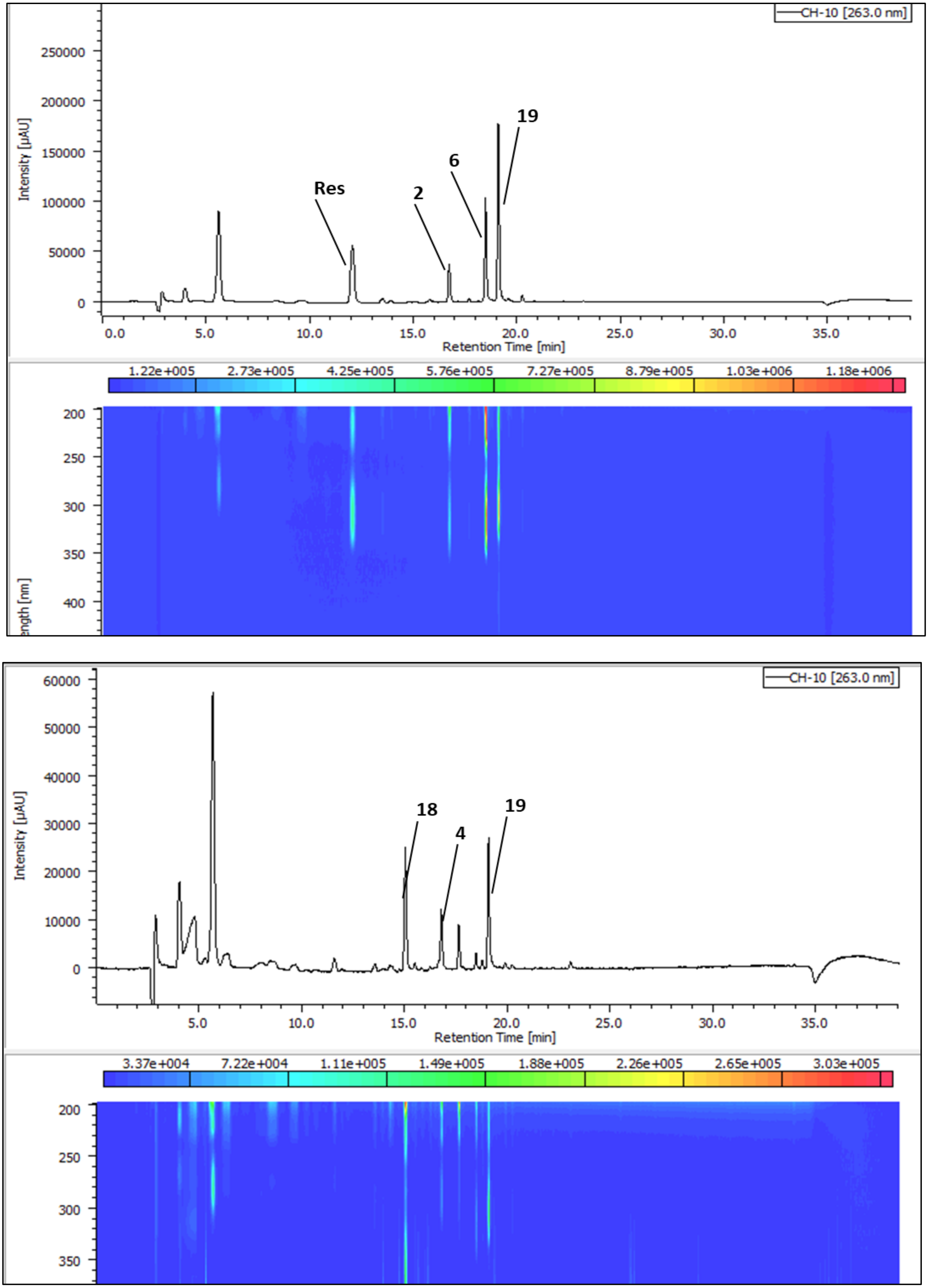

Figure S6. HPLC-PDA fingerprint of oxidized product mixture Ox11 (above) and Ox12 (below).

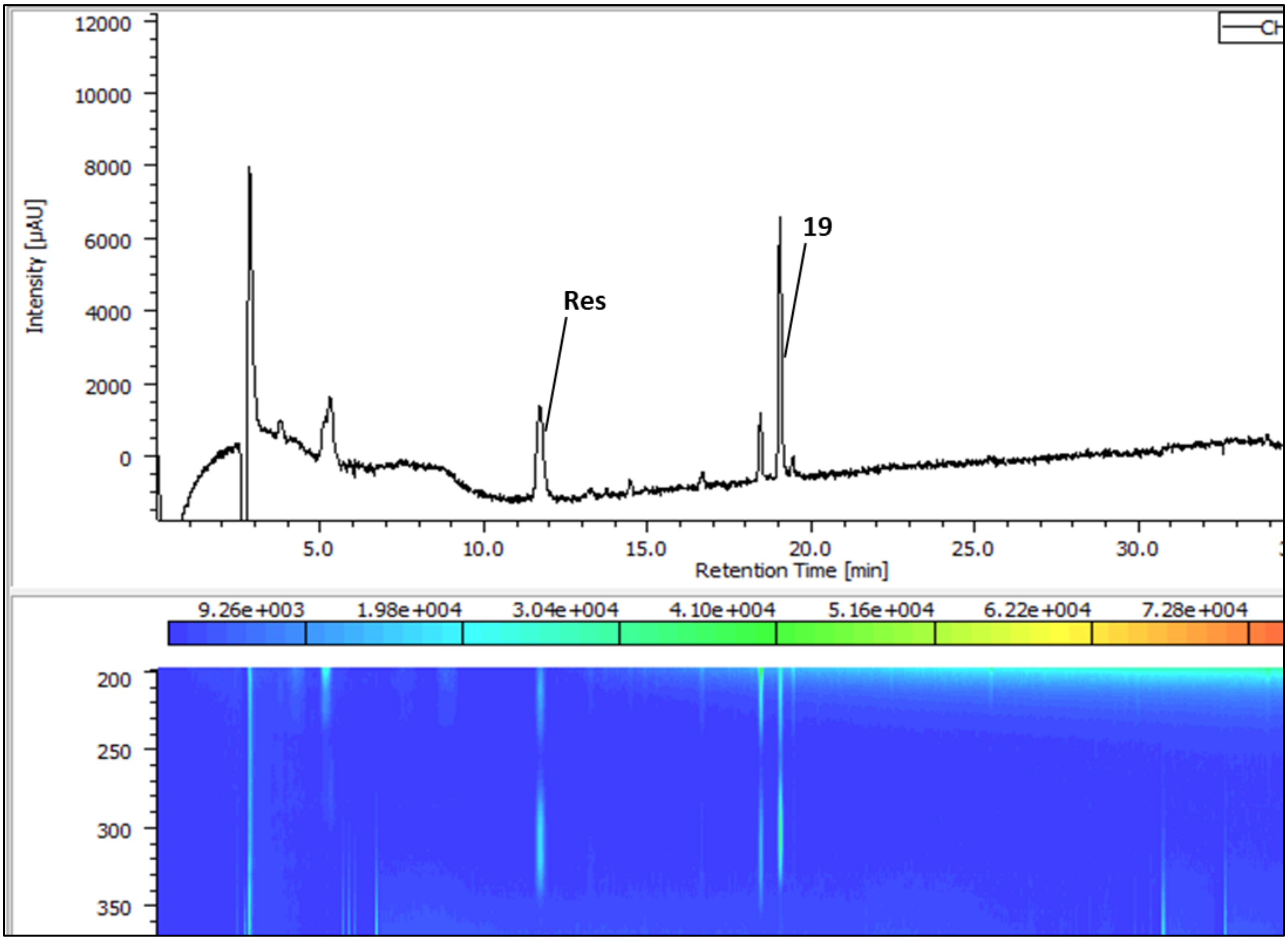

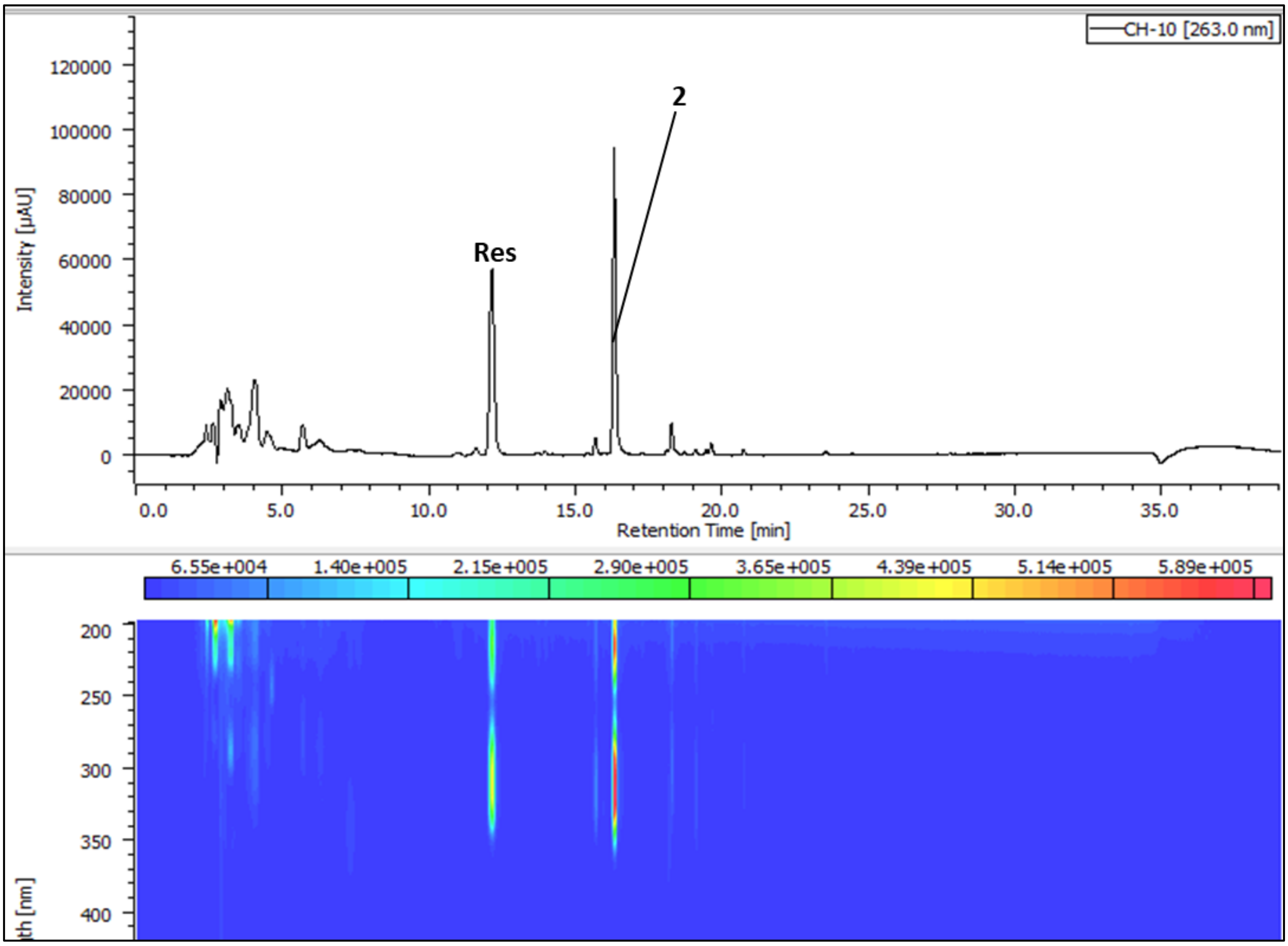

Figure S7. HPLC-PDA fingerprint of oxidized product mixture Ox13 (above) and Ox14 (below).

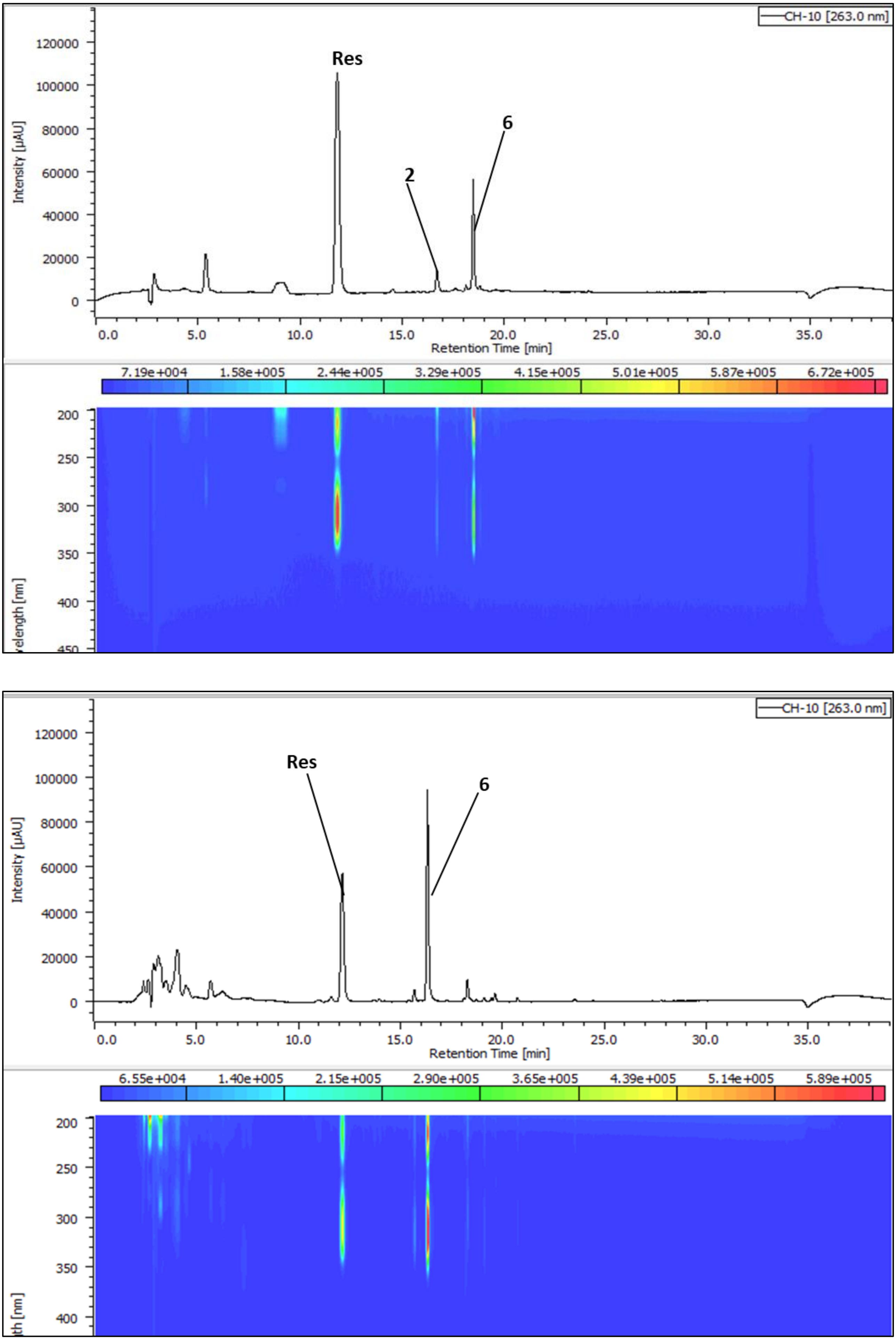

Figure S8. HPLC-PDA fingerprint of oxidized product mixture Ox15 (above) and Ox16 (below).

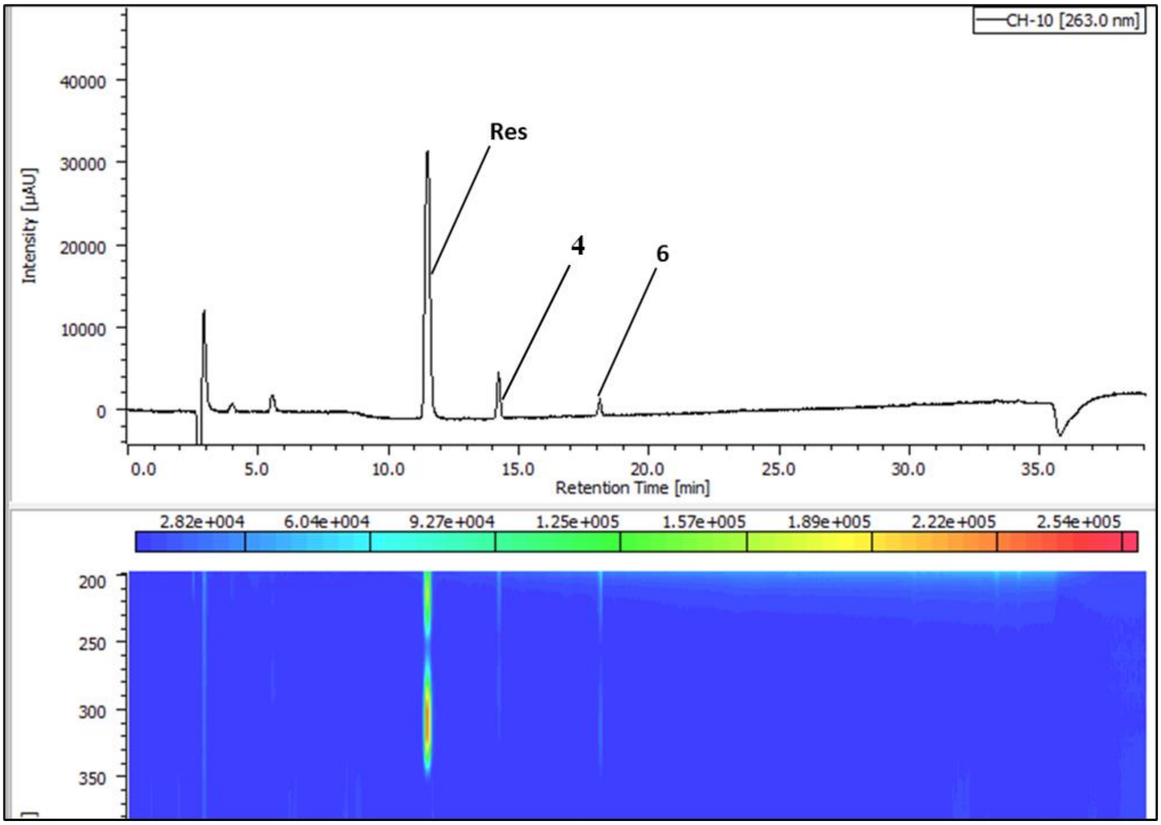

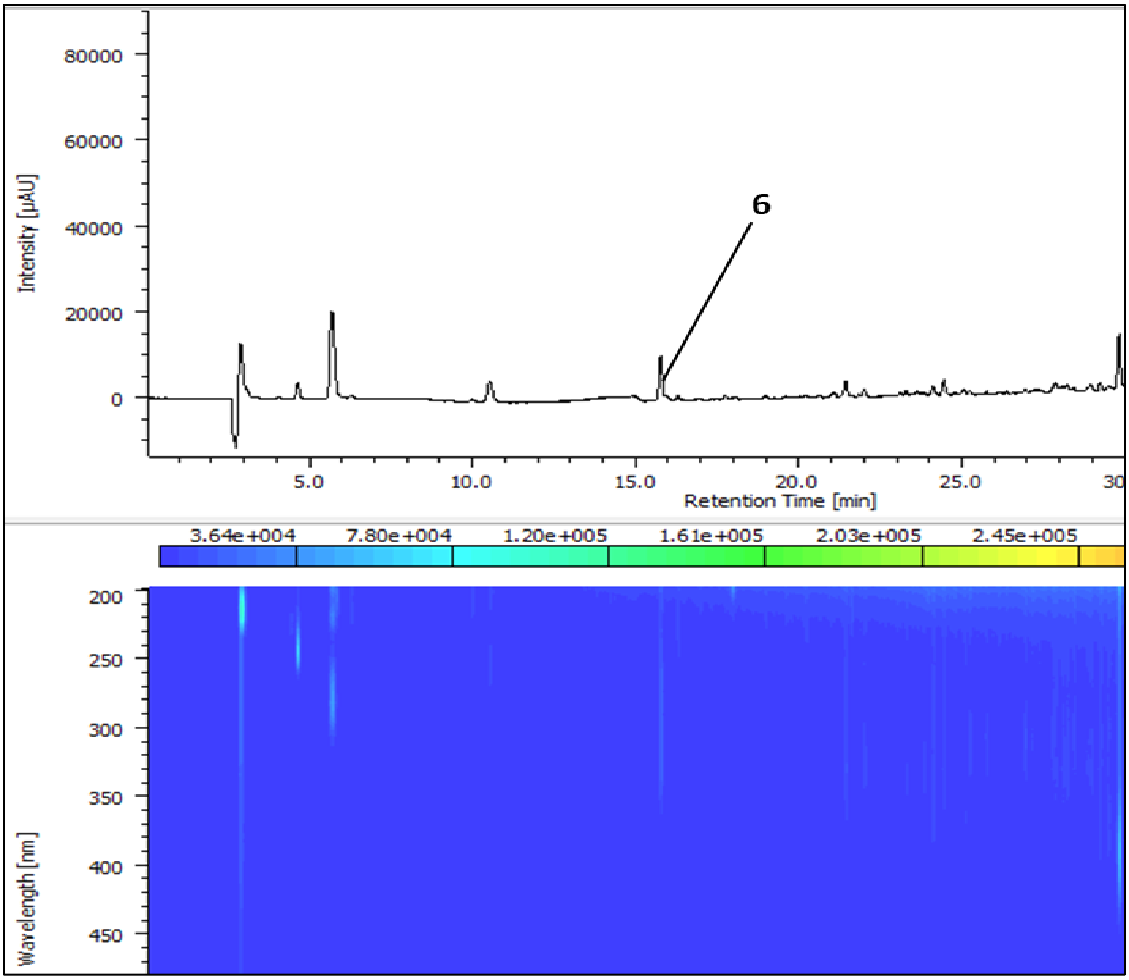

Figure S9. Metabolic profile (MS Data) of Ox1. Positive mode (above) & negative mode (below)

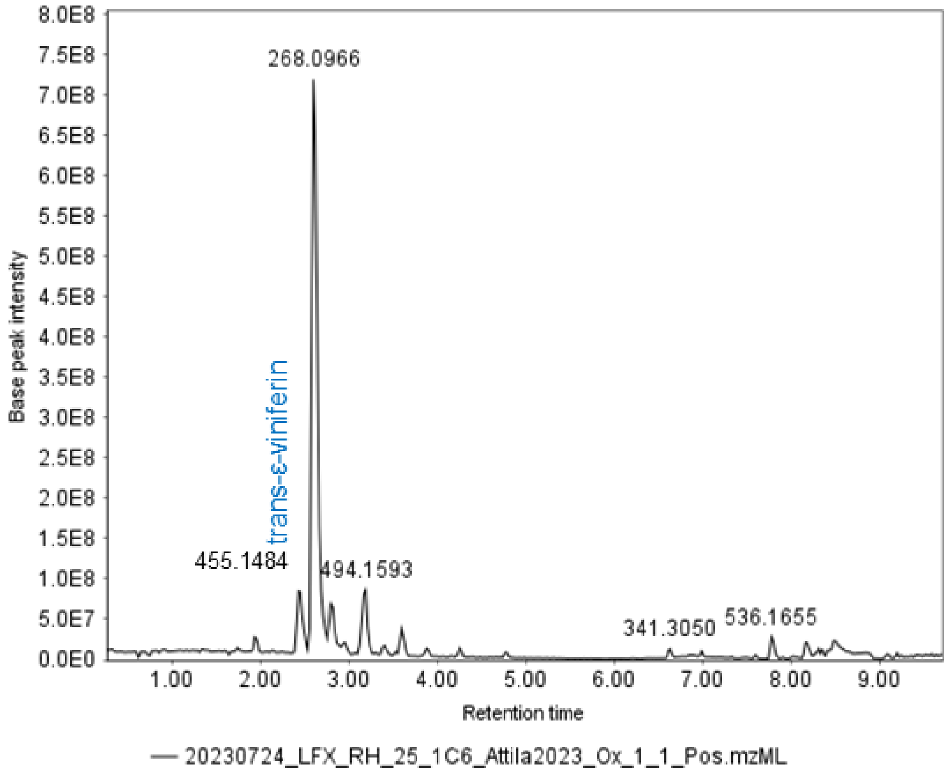

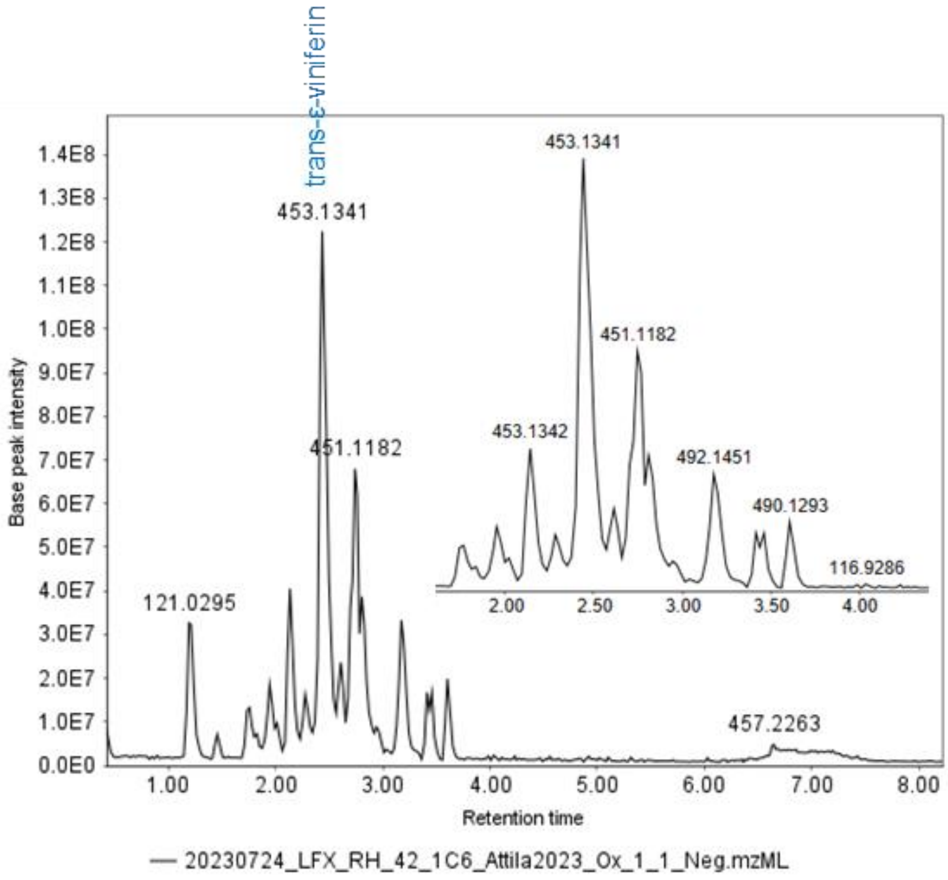

Figure S10. Metabolic profile (MS Data) of Ox2. Positive mode (above) & negative mode (below)

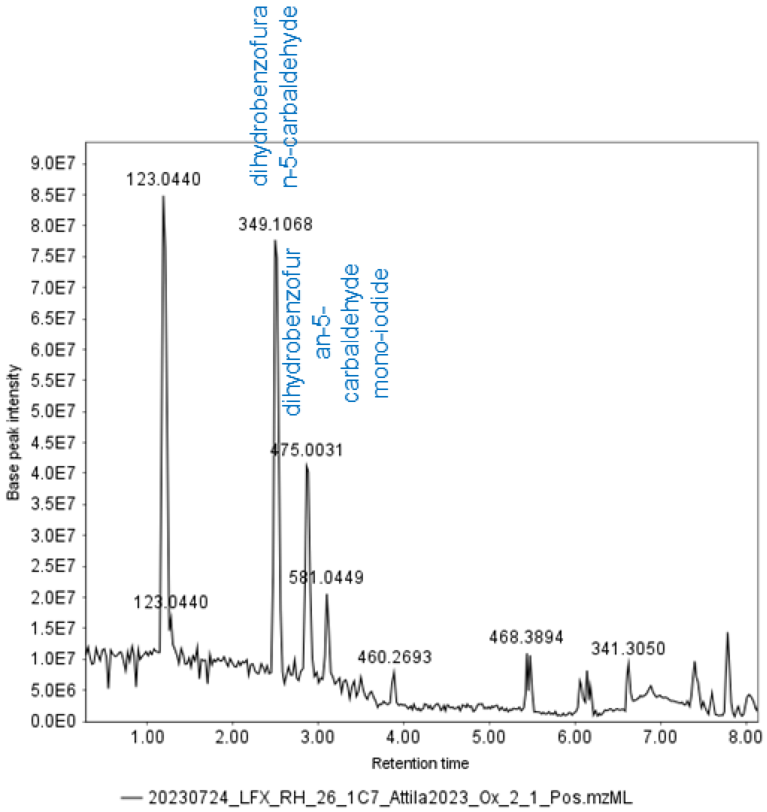

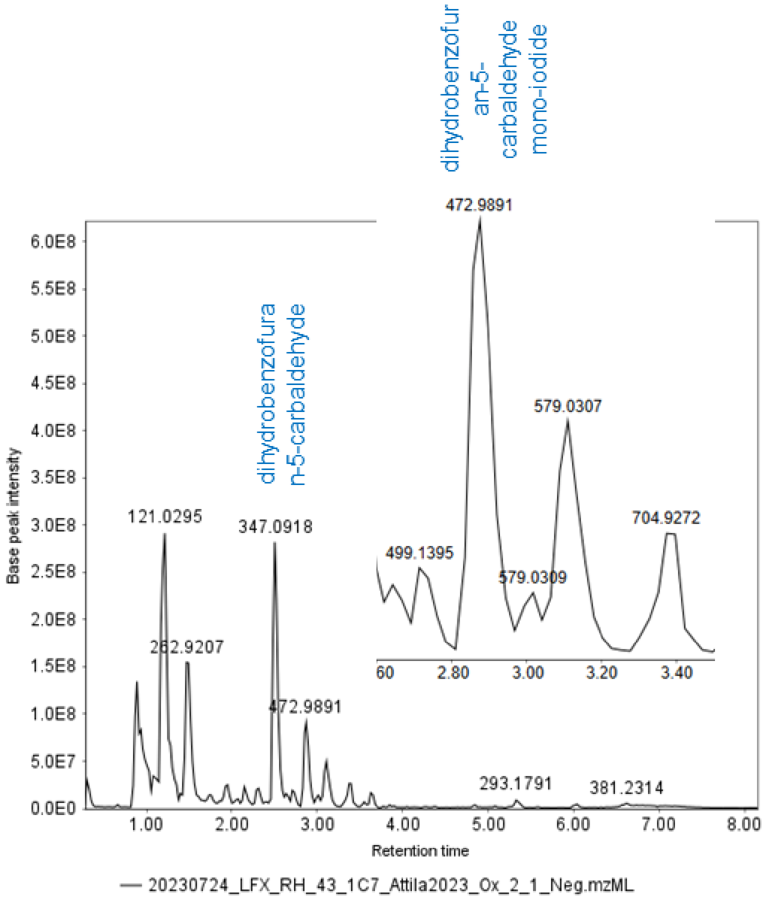

Figure S11. Metabolic profile (MS Data) of Ox3. Positive mode (above) & negative mode (below)

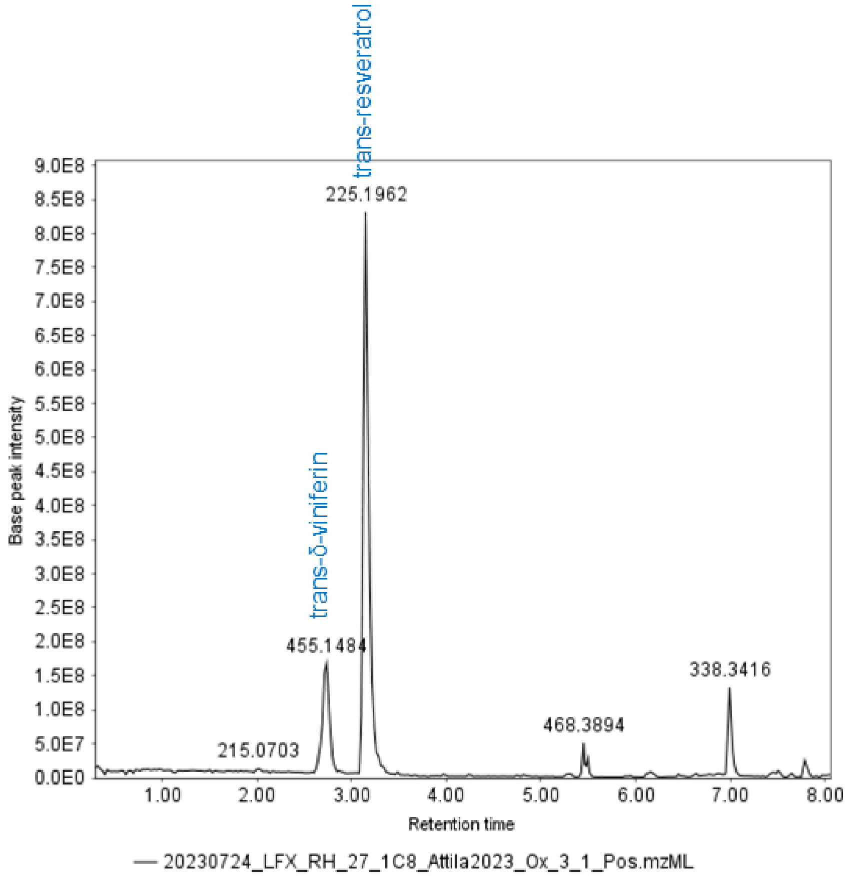

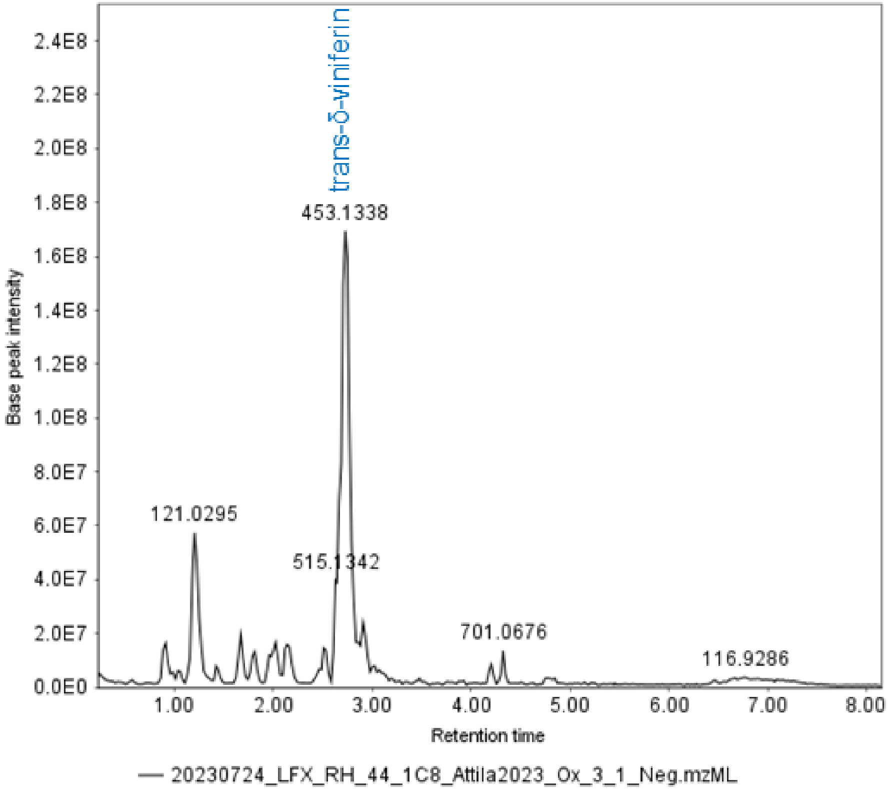

Figure S12. Metabolic profile (MS Data) of Ox4. Positive mode (above) & negative mode (below)

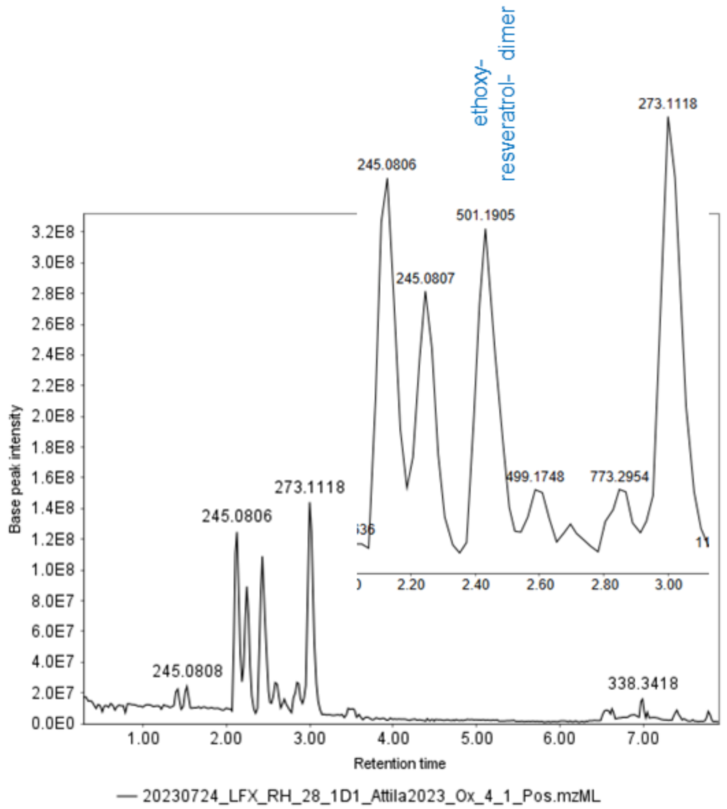

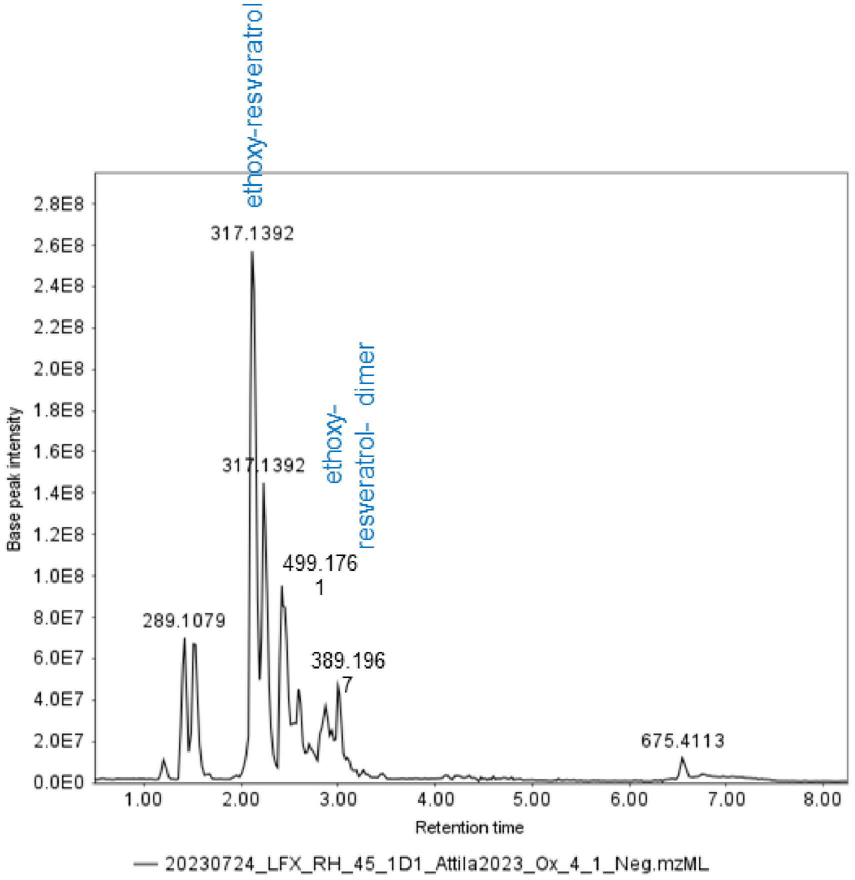

Figure S13. Metabolic profile (MS Data) of Ox5. Positive mode (above) & negative mode (below)

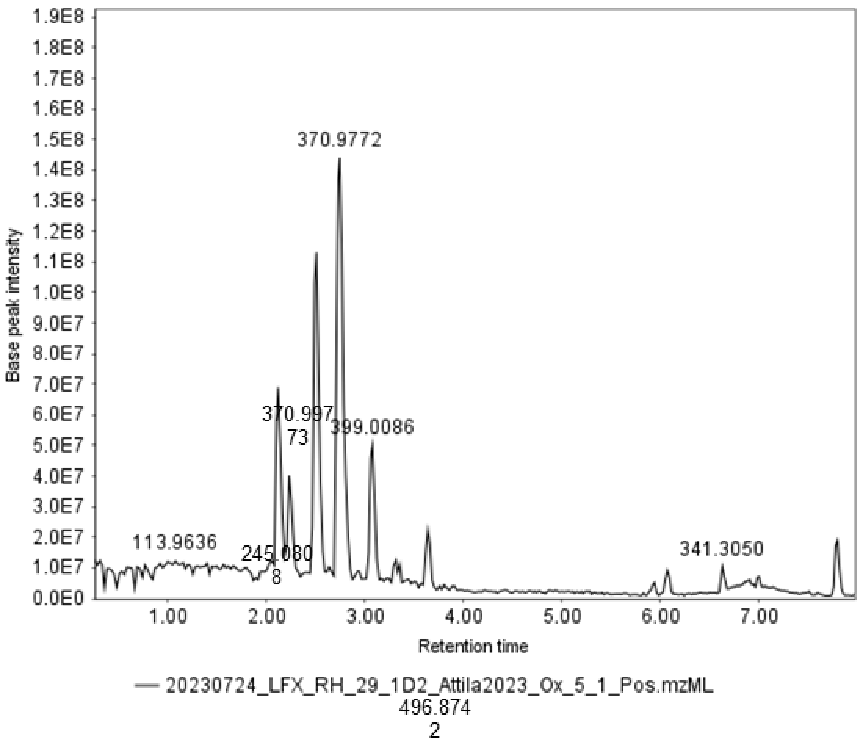

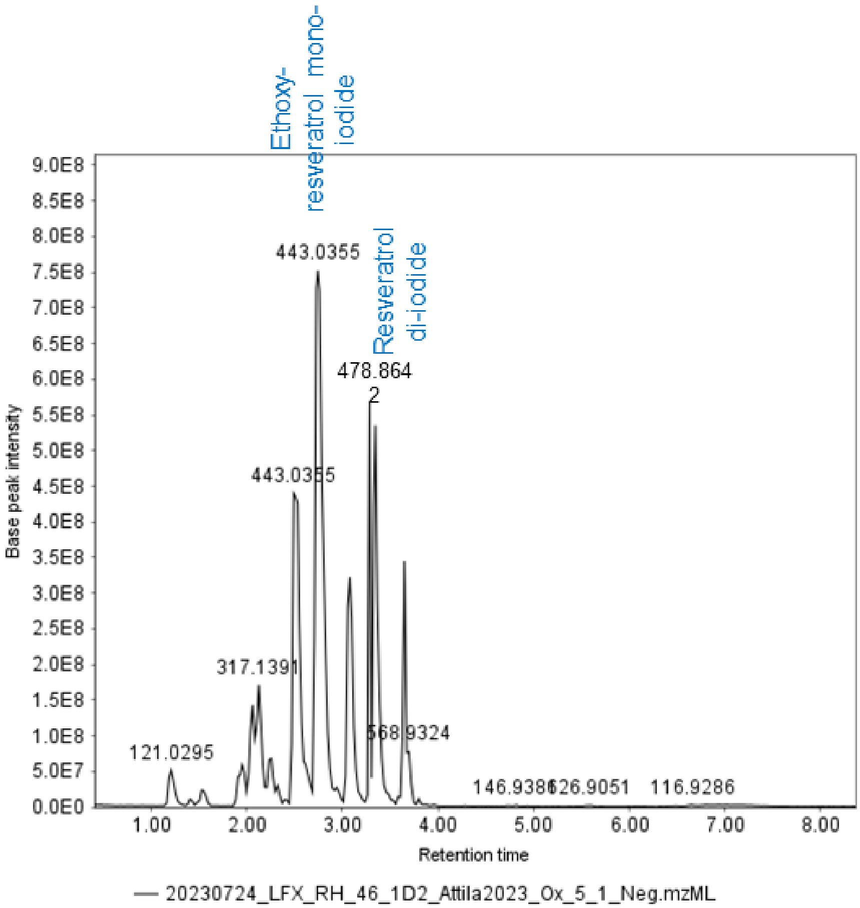

Figure S14. Metabolic profile (MS Data) of Ox6. Positive mode (above) & negative mode (method)

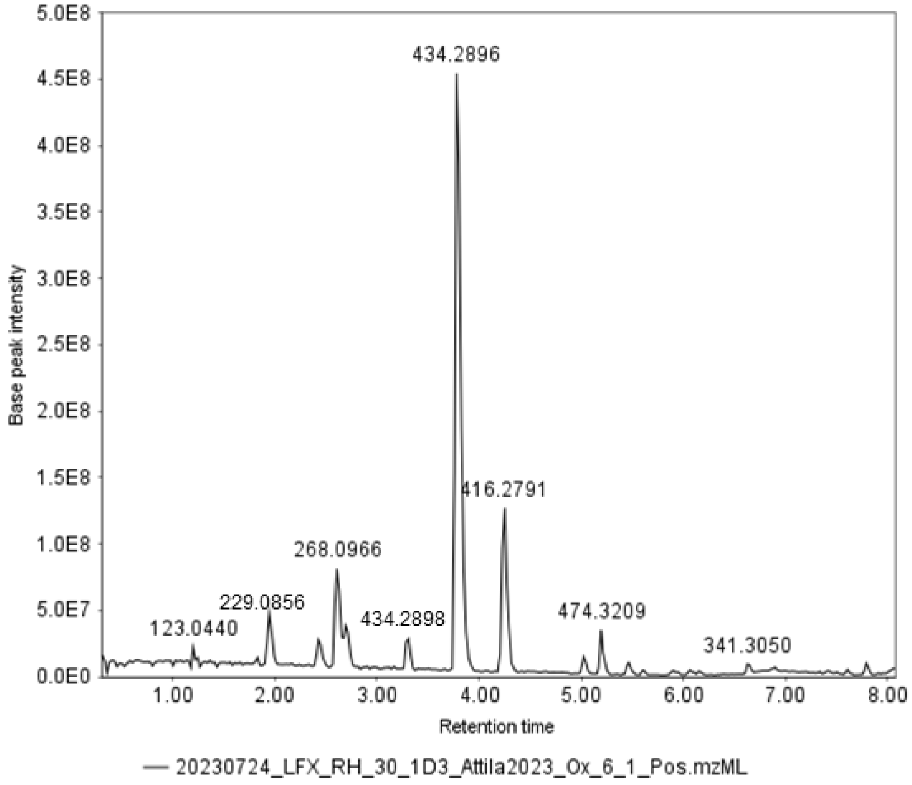

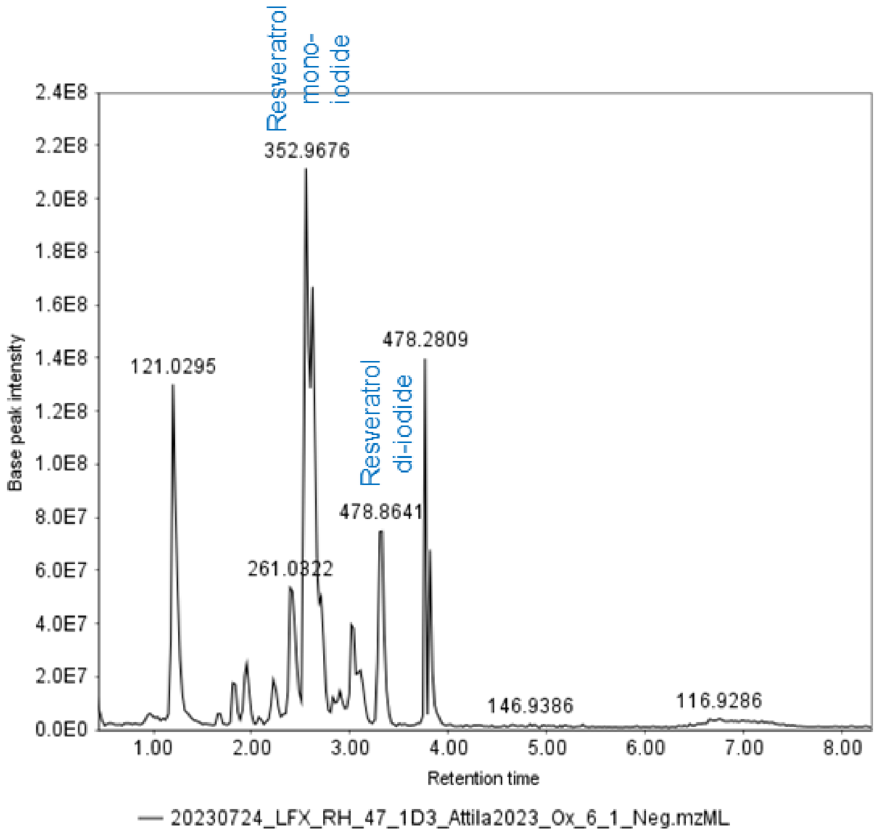

Figure S15. Metabolic profile (MS Data) of Ox7. Positive mode (above) & negative mode (below)

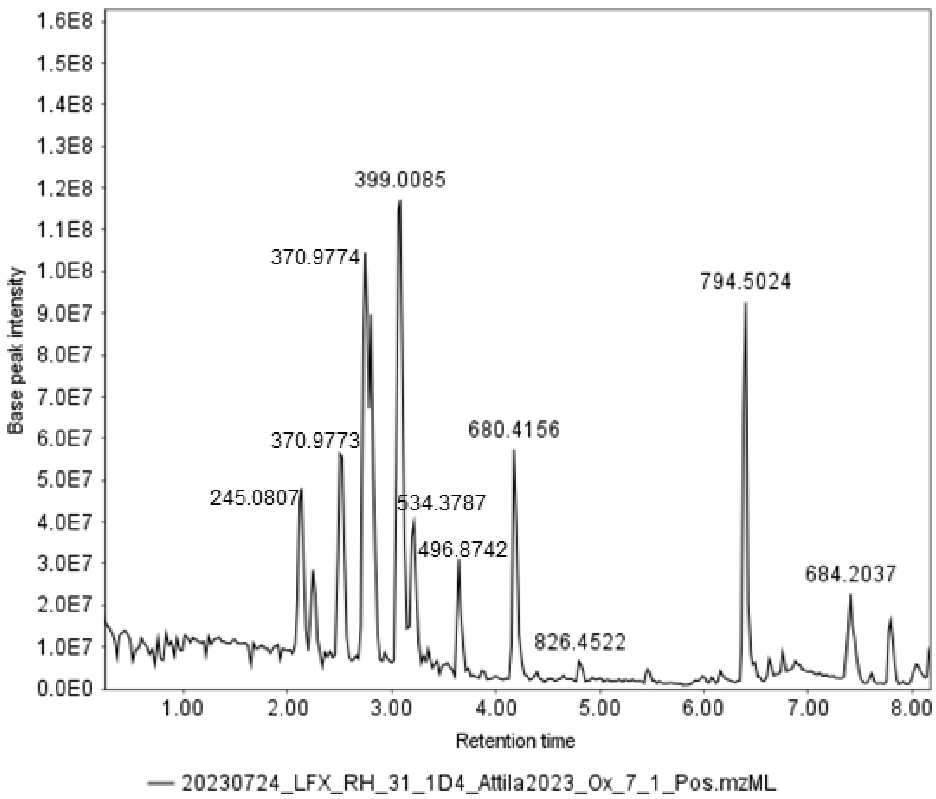

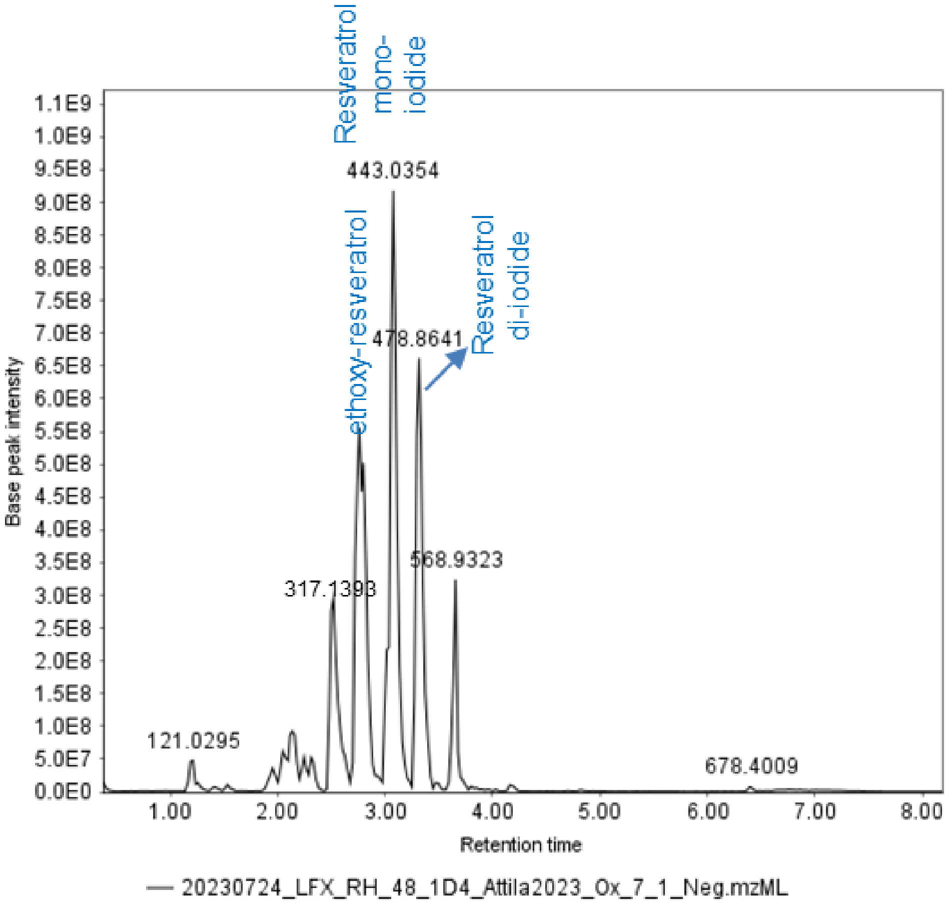

Figure S16. Metabolic profile (MS Data) of Ox8. Negative mode only

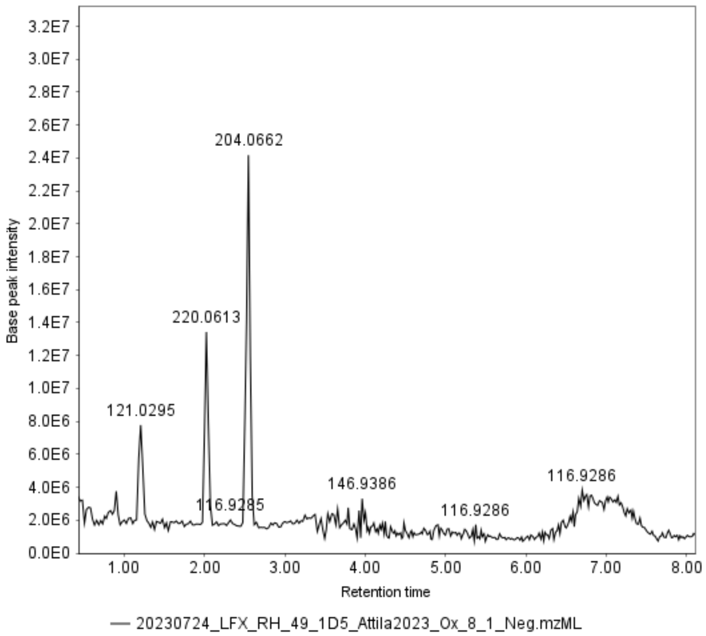

Figure S17. Metabolic profile (MS Data) of Ox9. Positive mode (above) & negative mode (below)

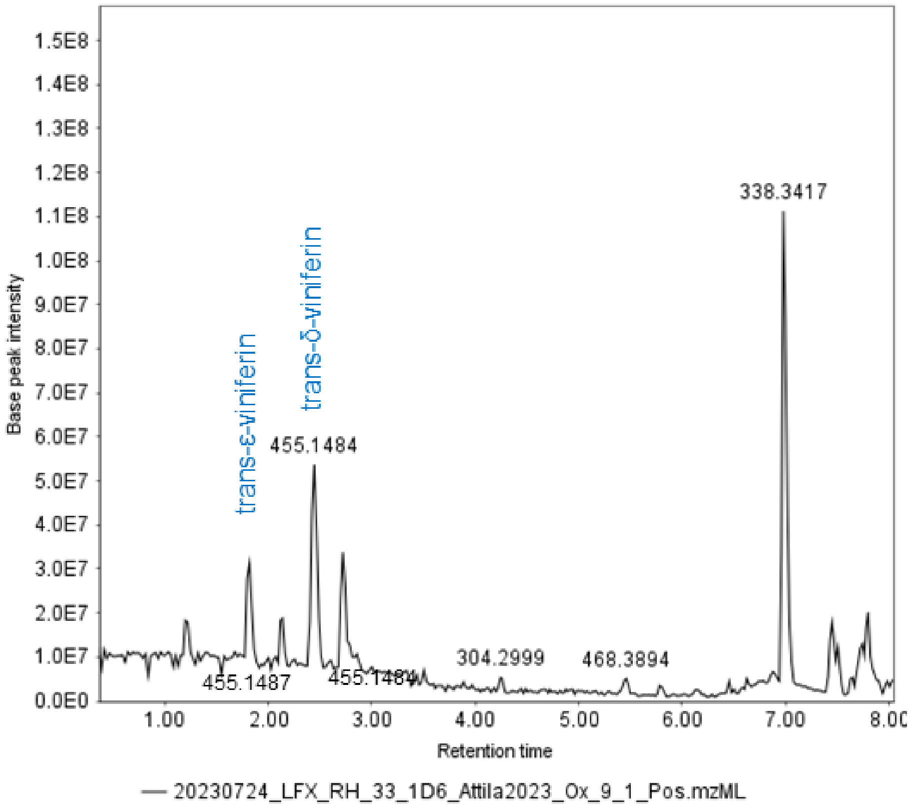

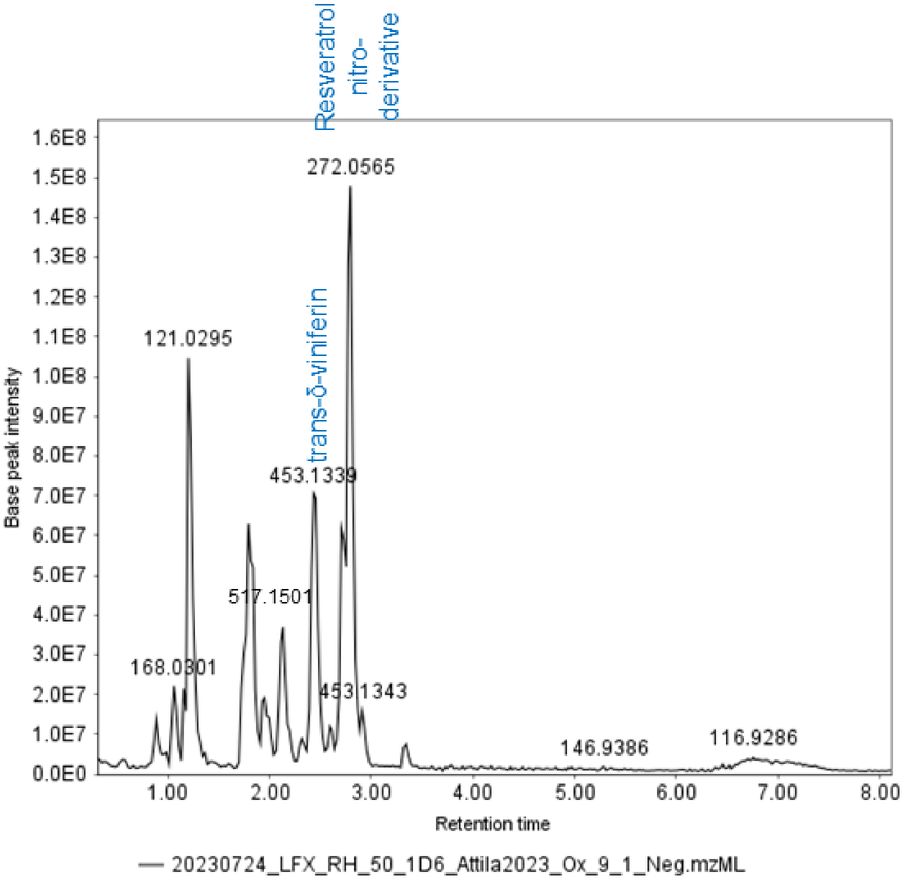

Figure S18. Metabolic profile (MS Data) of Ox10. Positive mode (above) & negative mode (below)

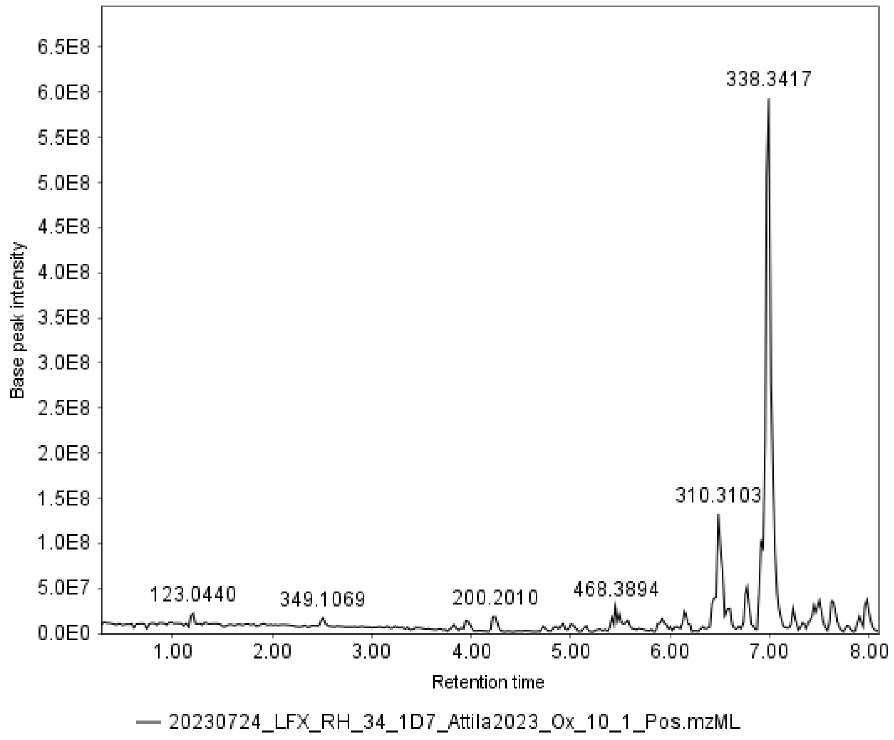

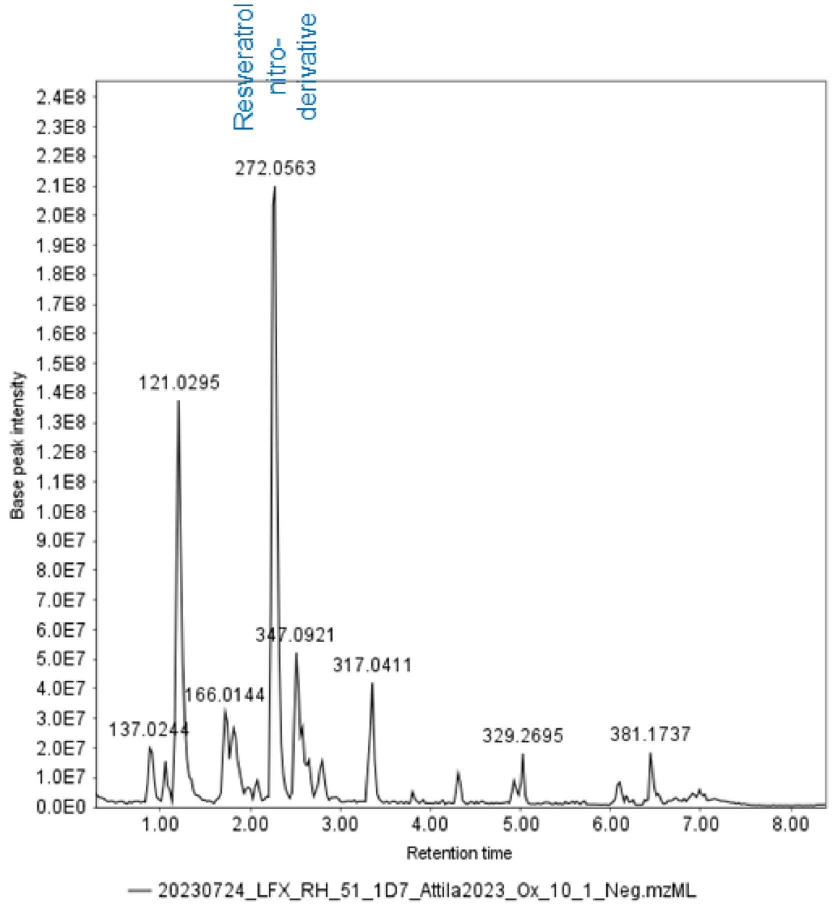

Figure S19. Metabolic profile (MS Data) of Ox11. Positive mode (above) & negative mode (below)

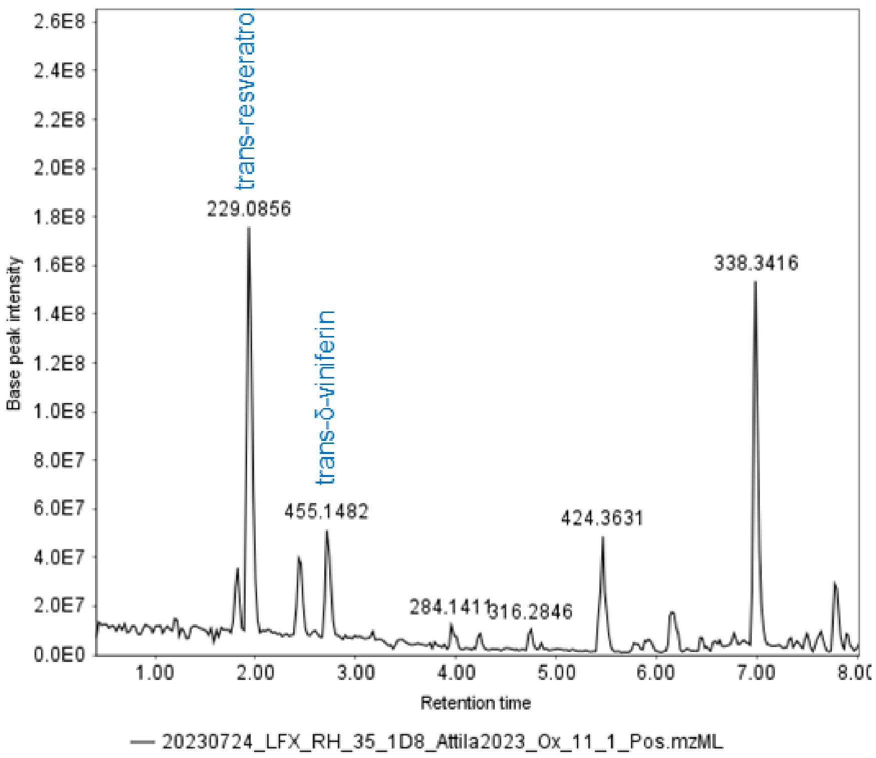

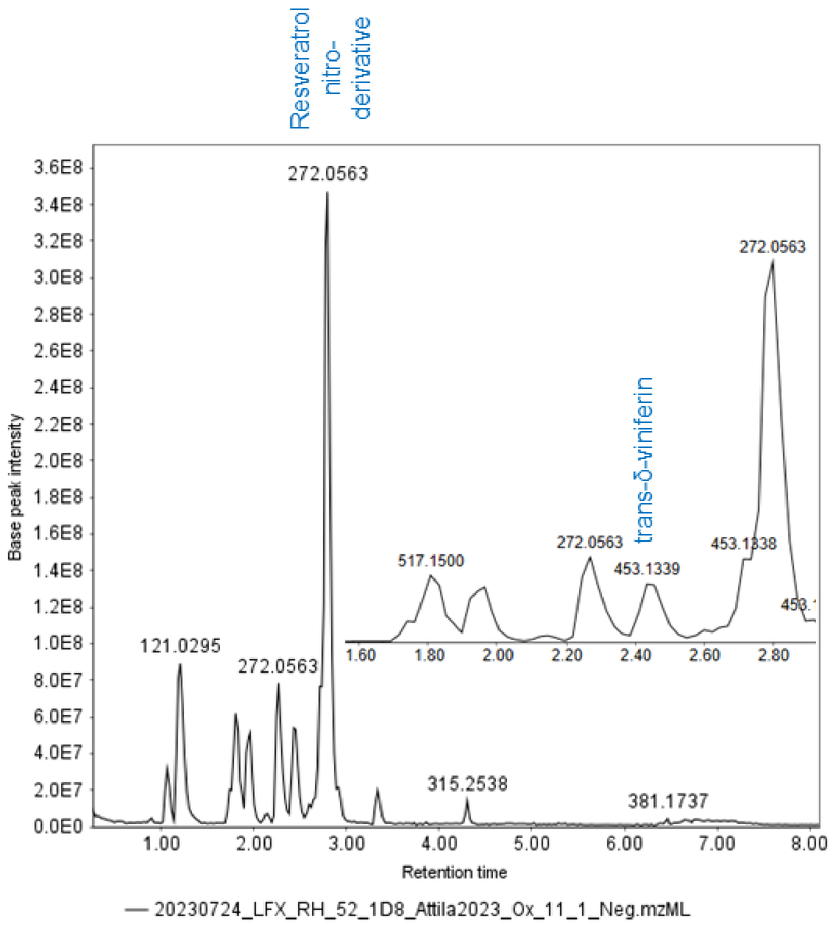

Figure S20. Metabolic profile (MS Data) of Ox12. Positive mode (above) & negative mode (below)

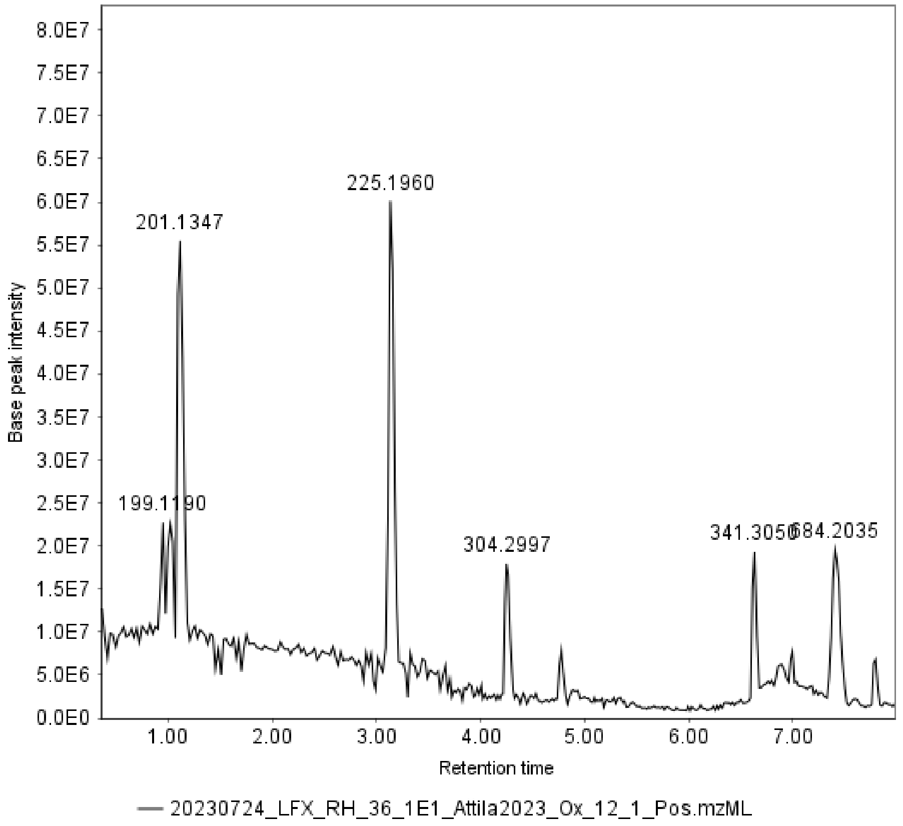

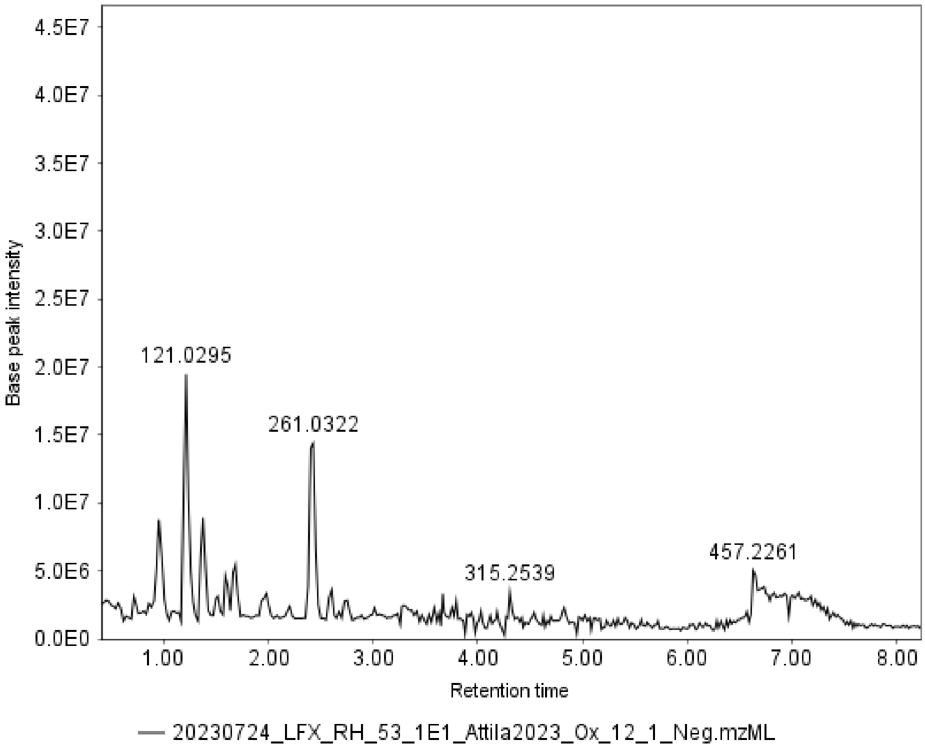

Figure S21. Metabolic profile (MS Data) of Ox13. Positive mode (above) & negative mode (below)

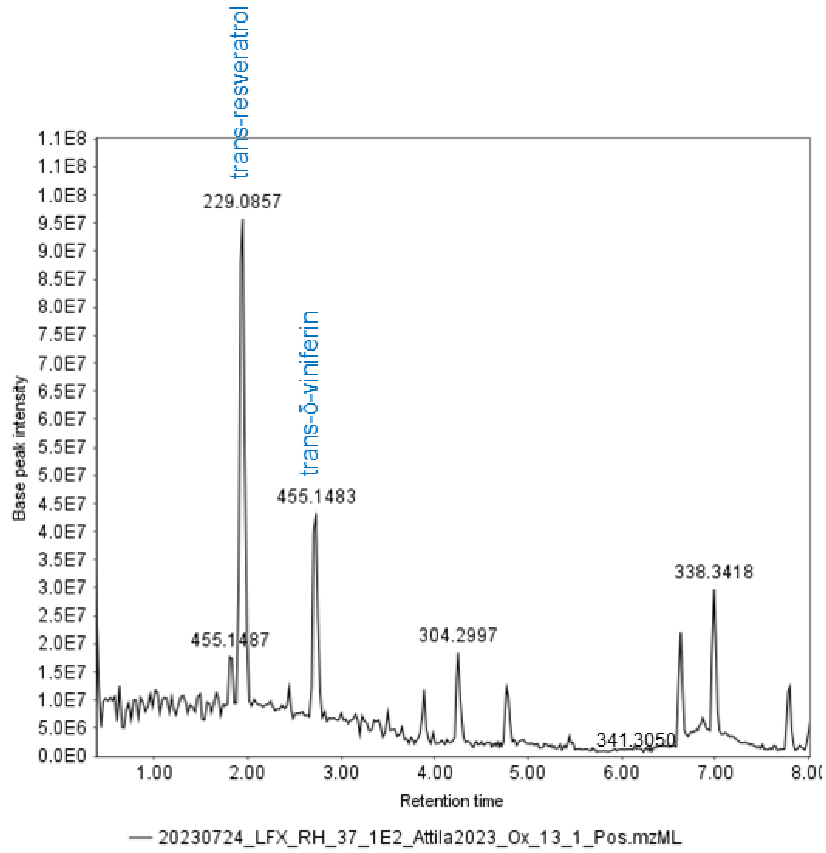

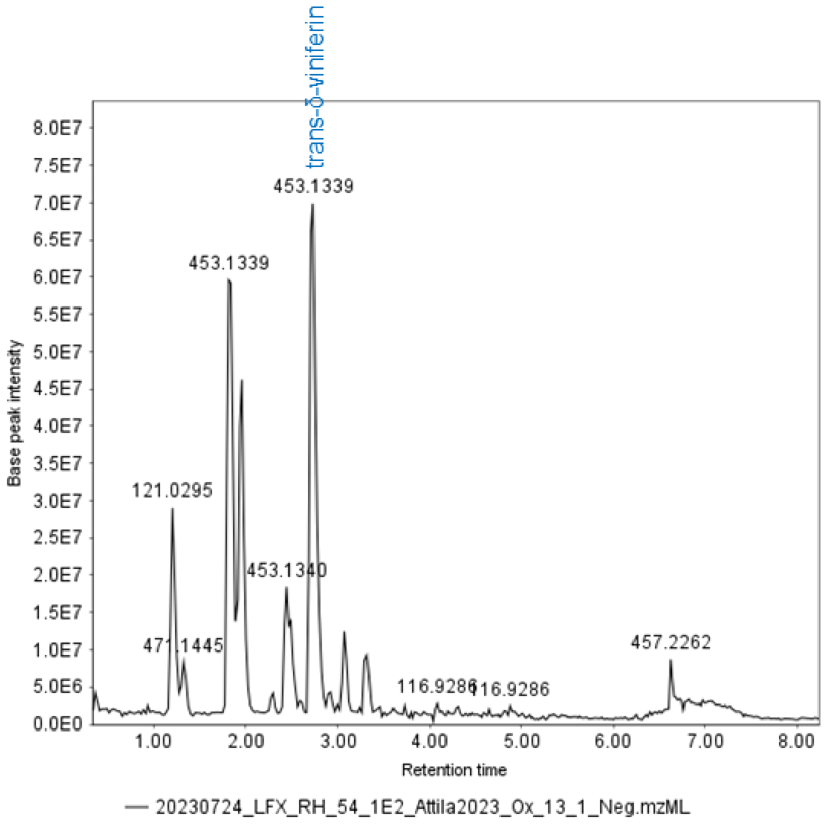

Figure S22. Metabolic profile (MS Data) of Ox14. Positive mode (above) & negative mode (below)

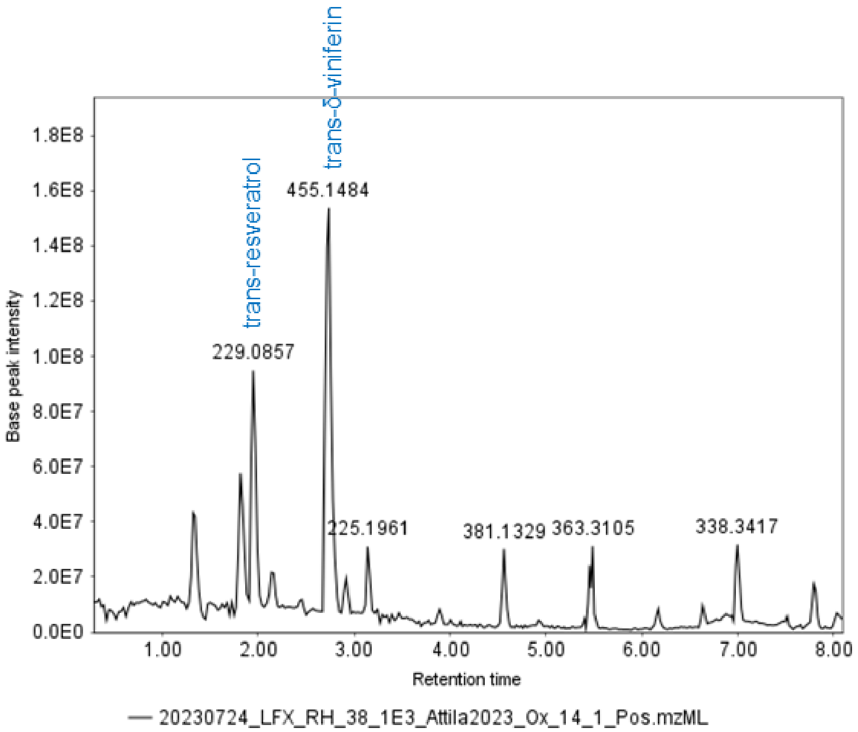

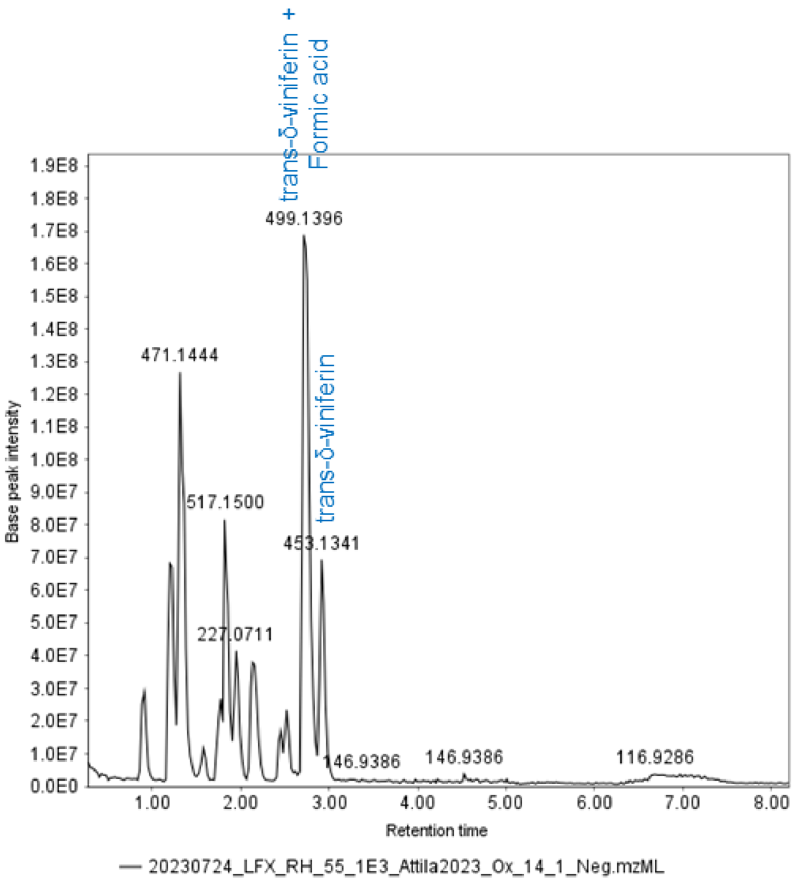

Figure S23. Metabolic profile (MS Data) of Ox15. Positive mode (above) & negative mode (below)

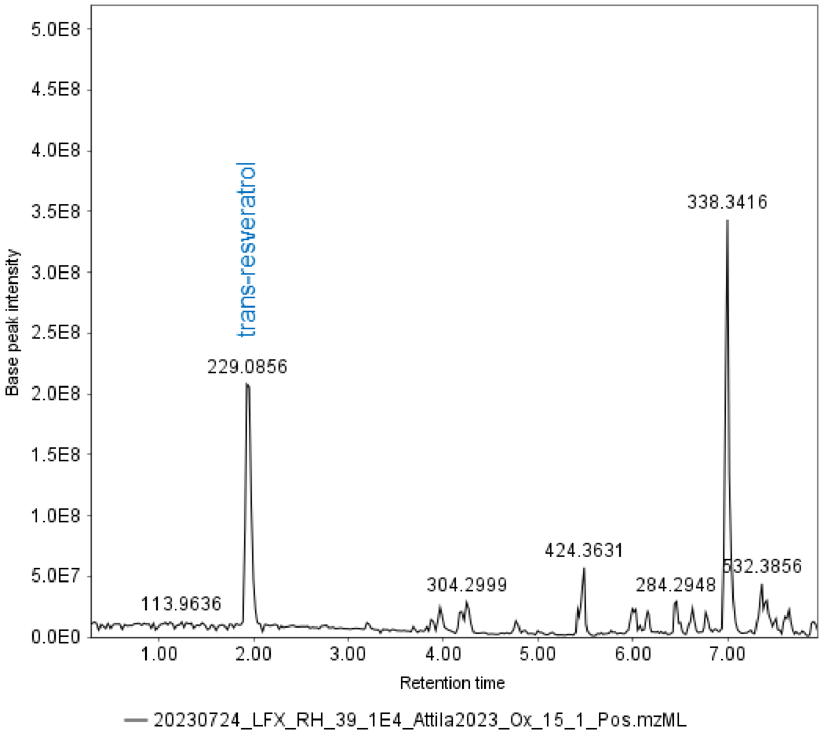

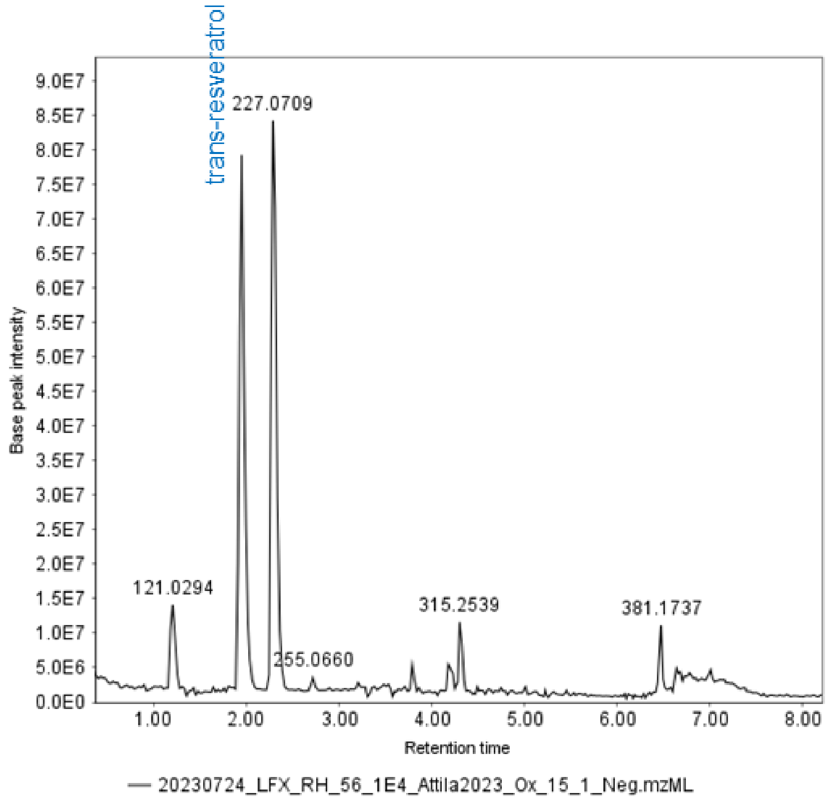

Figure S24. Metabolic profile (MS Data) of Ox16. Positive mode (above) & negative mode (below)

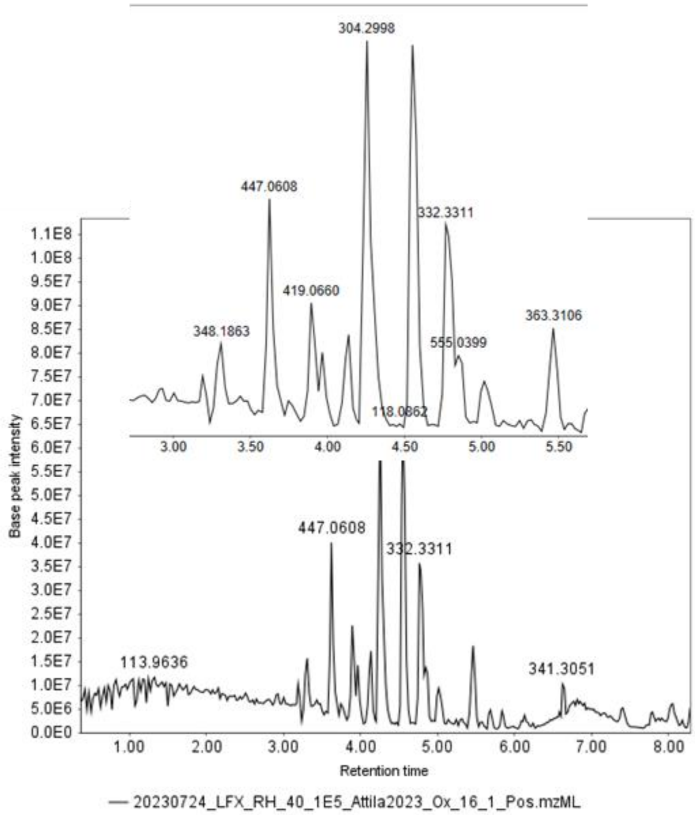

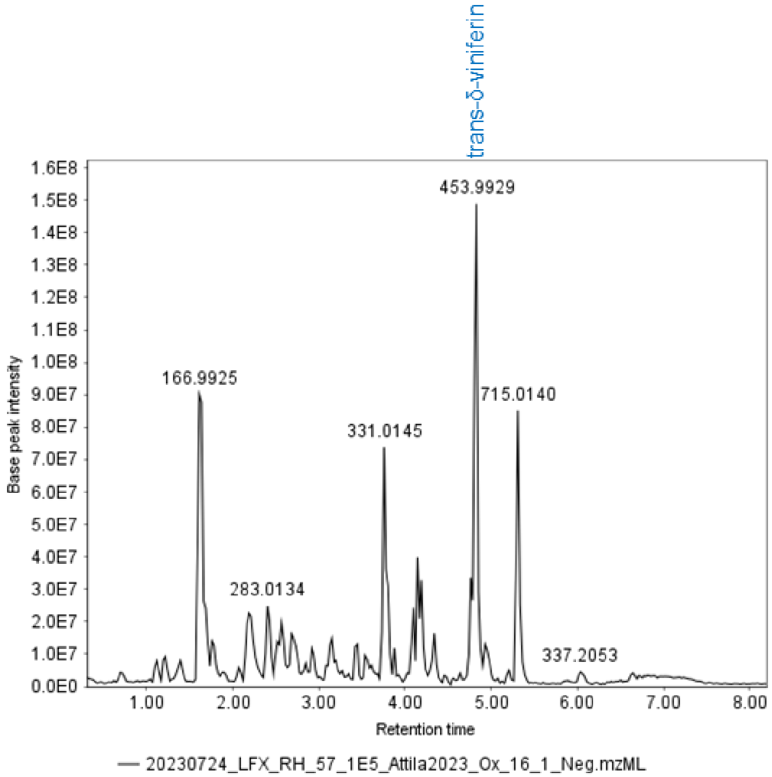

Figure S25. 3-D Metabolic profile charts of Ox1-Ox16; Positive mode (above) and Negative mode (below)

<u>Parameters</u>
MSn level filter >= 2, Polarity: +ve
Retention time resolution: 400
m/z resolution: 400

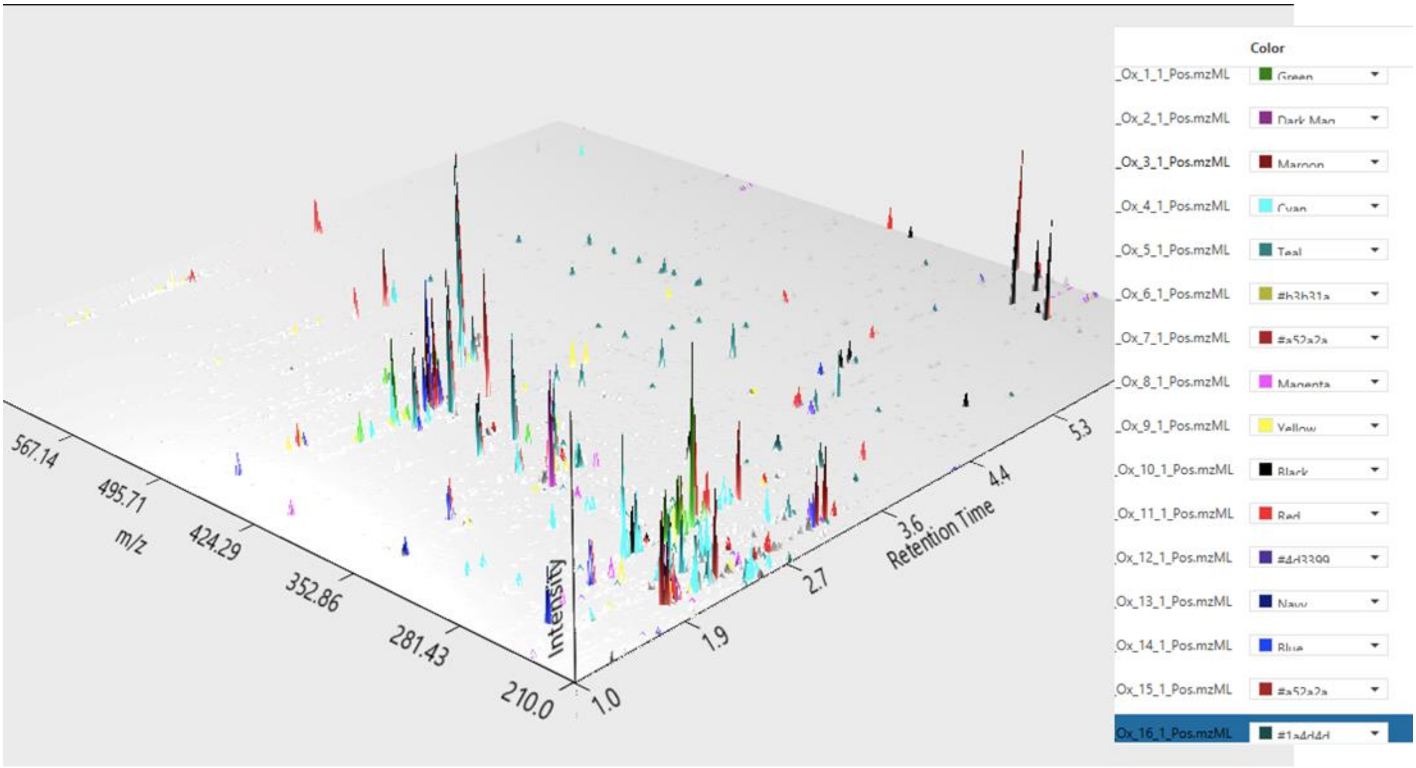

<u>Parameters</u>
MSn level filter >= 2, Polarity: -ve
Retention time resolution: 400
m/z resolution: 400

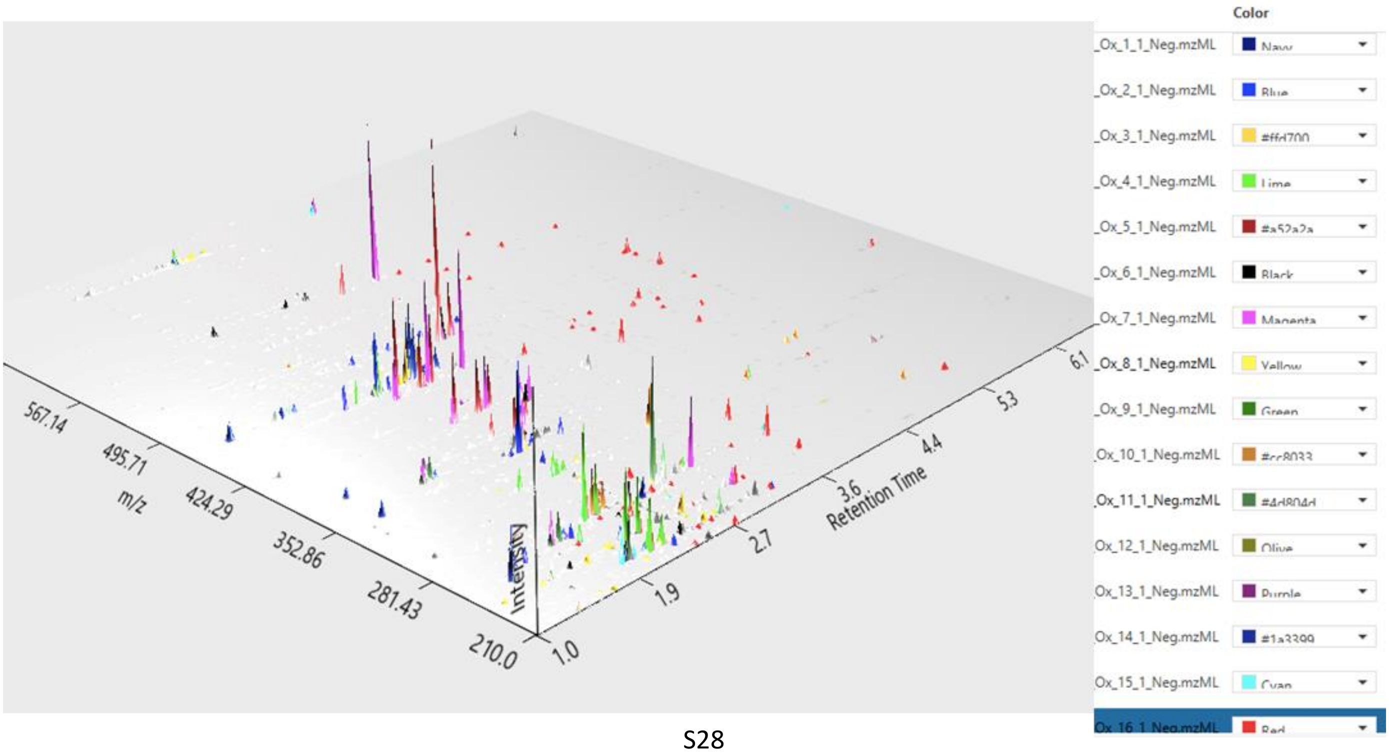

Figure S26. Compound **1**, HRMS (above; positive mode) and Compound **3**, HRMS (below; positive mode)

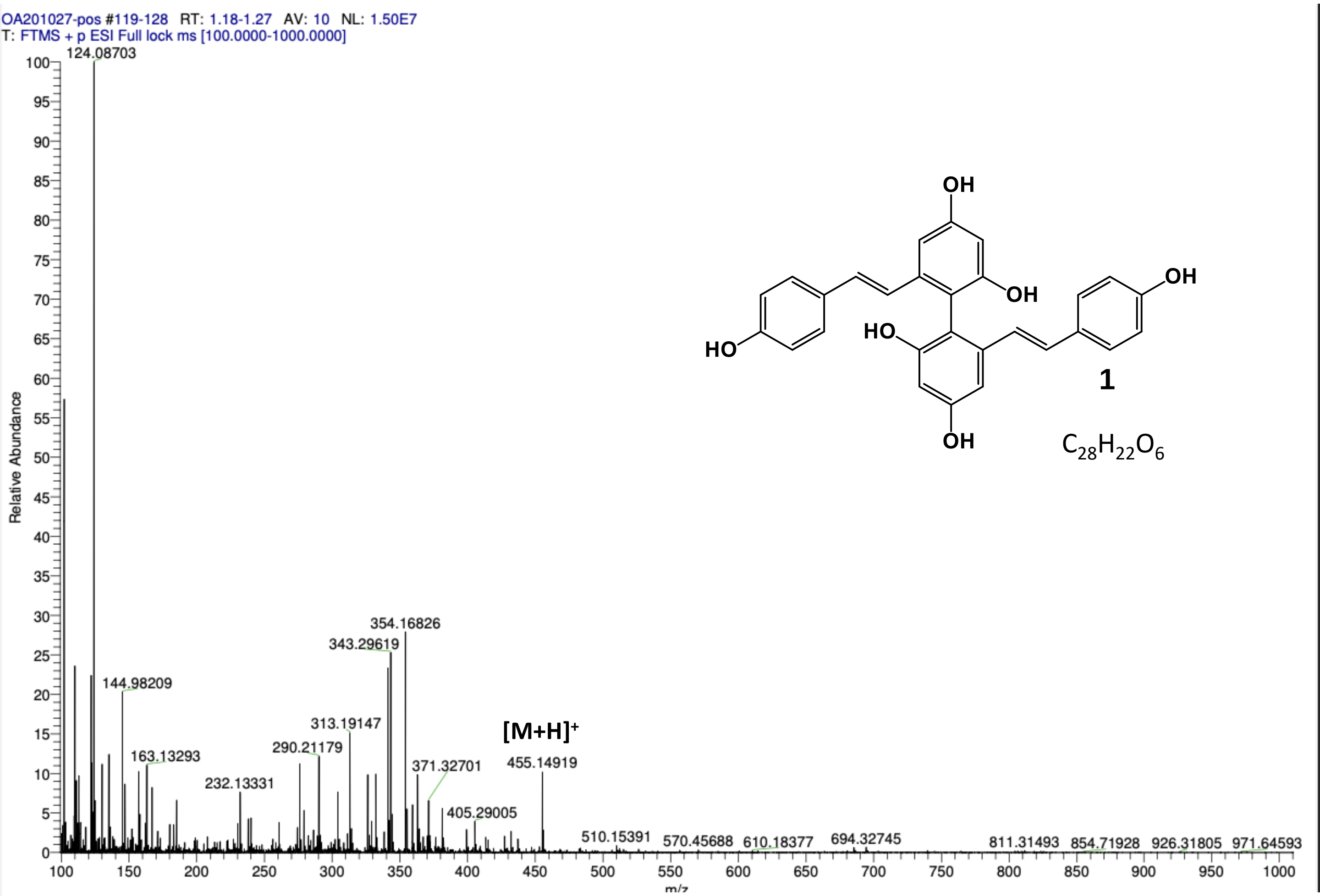

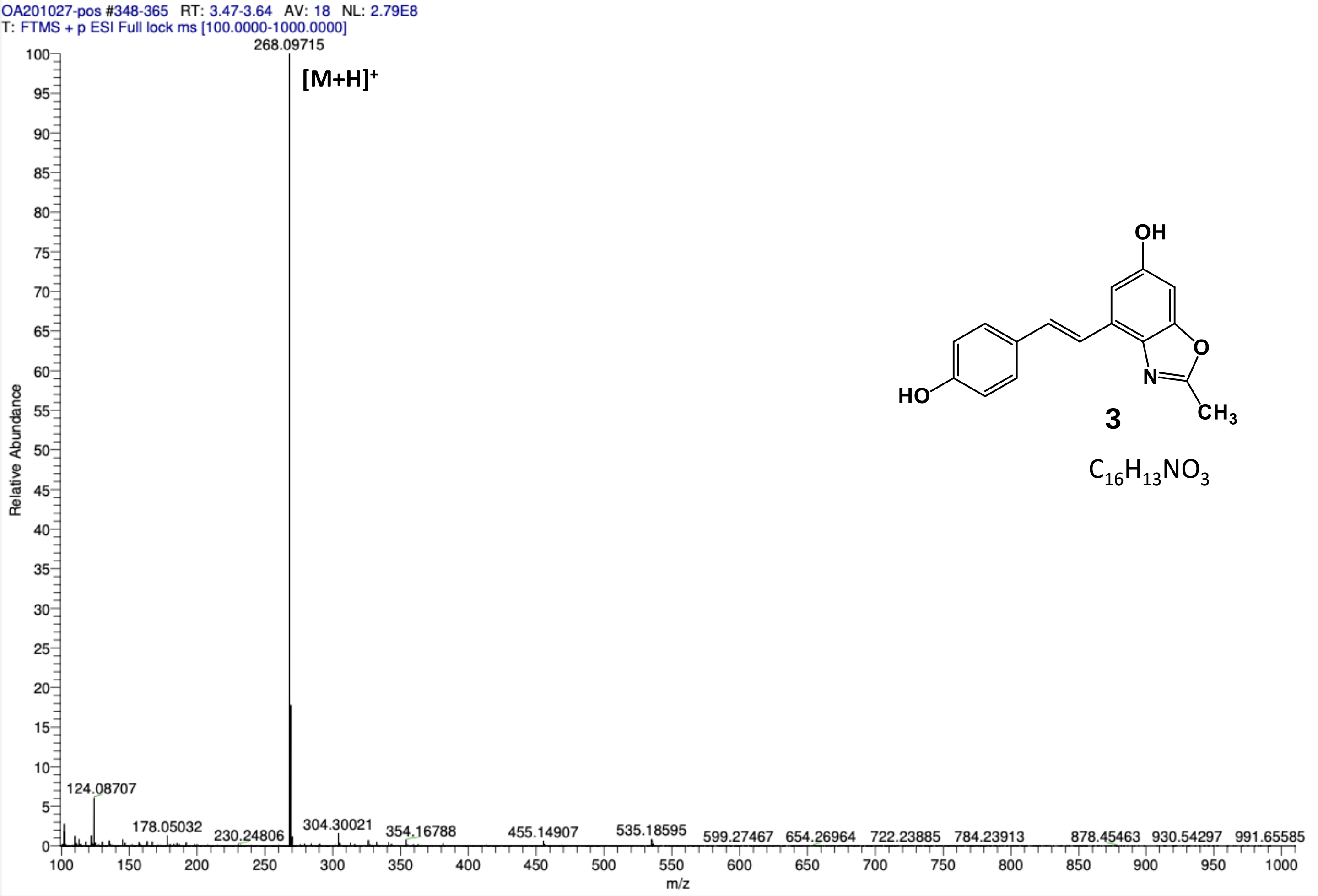

Figure S27. Compound **4**, HRMS (above; negative mode) and Compound **7**, HRMS (below; positive mode).

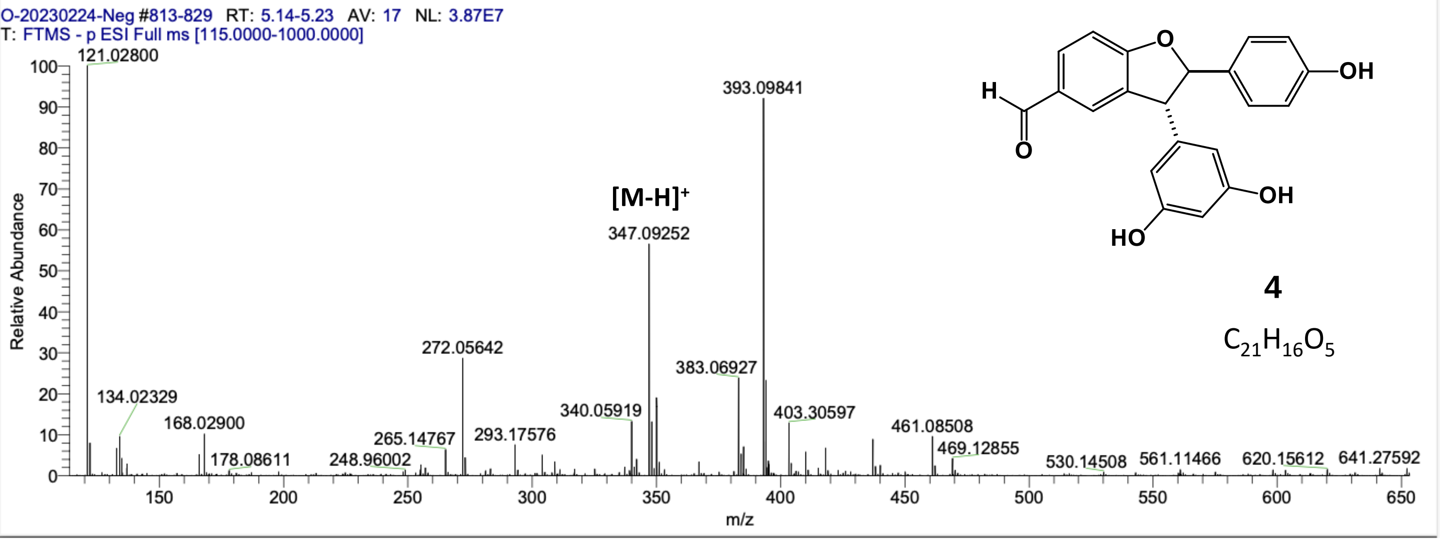

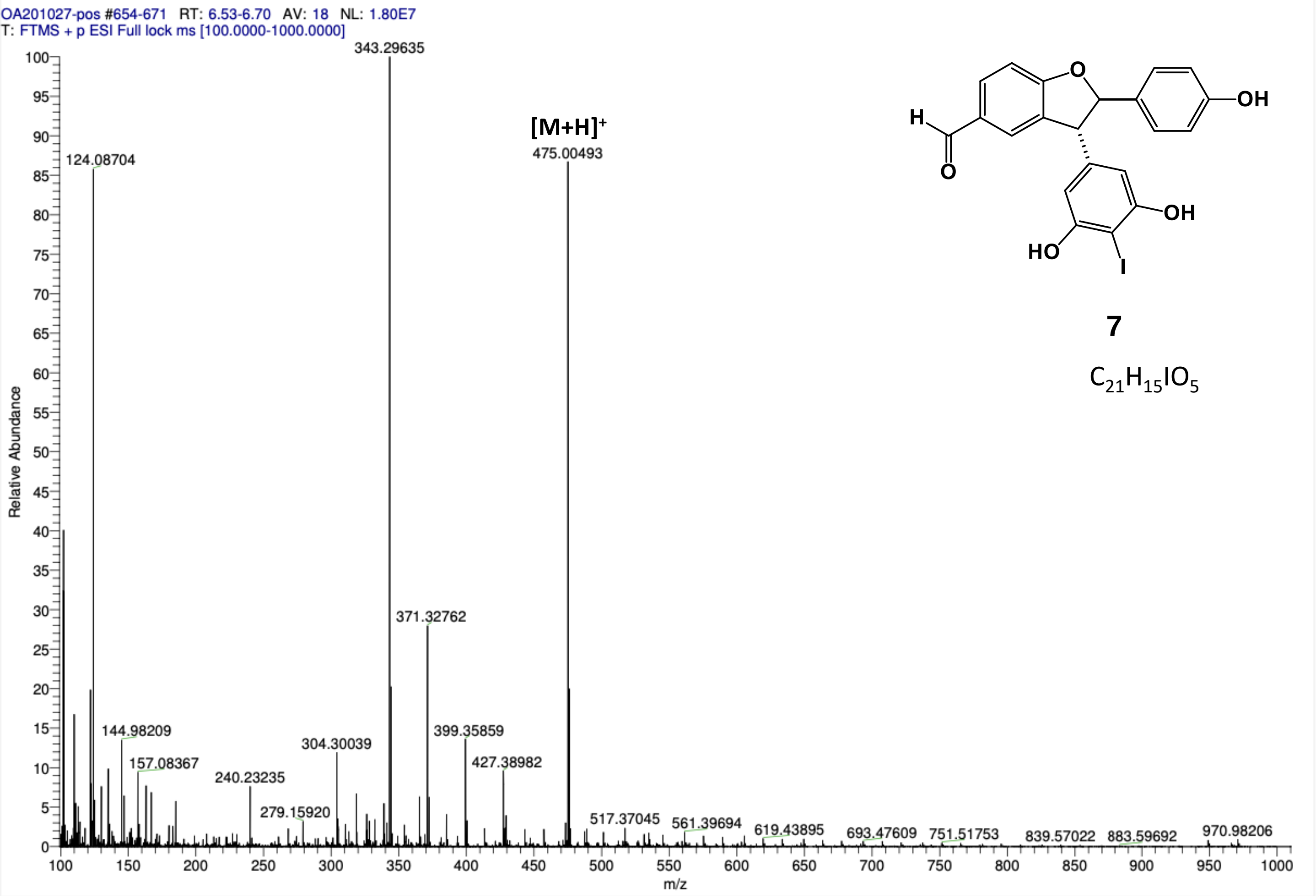

Figure S28. Compound **11**, HRMS (negative mode).

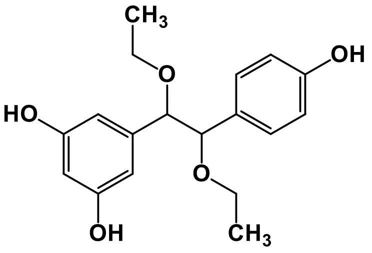

11
$C_{18}H_{22}O_5$

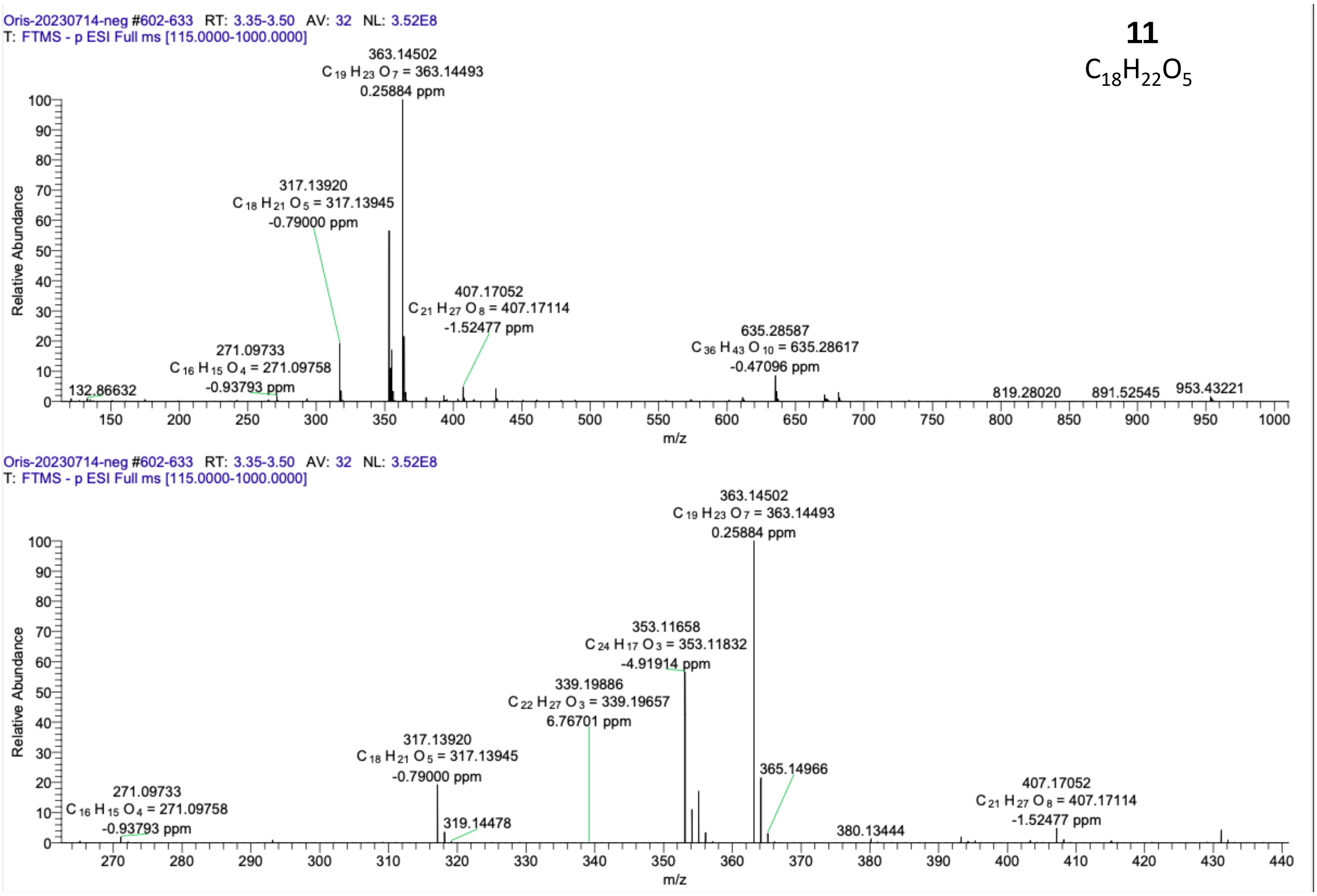

Figure S29. Compound **13**, HRMS (negative mode).

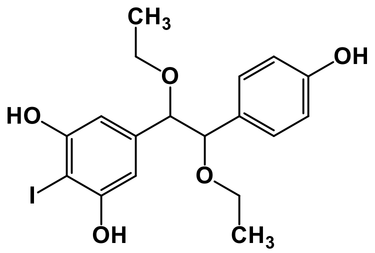

13

$C_{18}H_{21}IO_5$

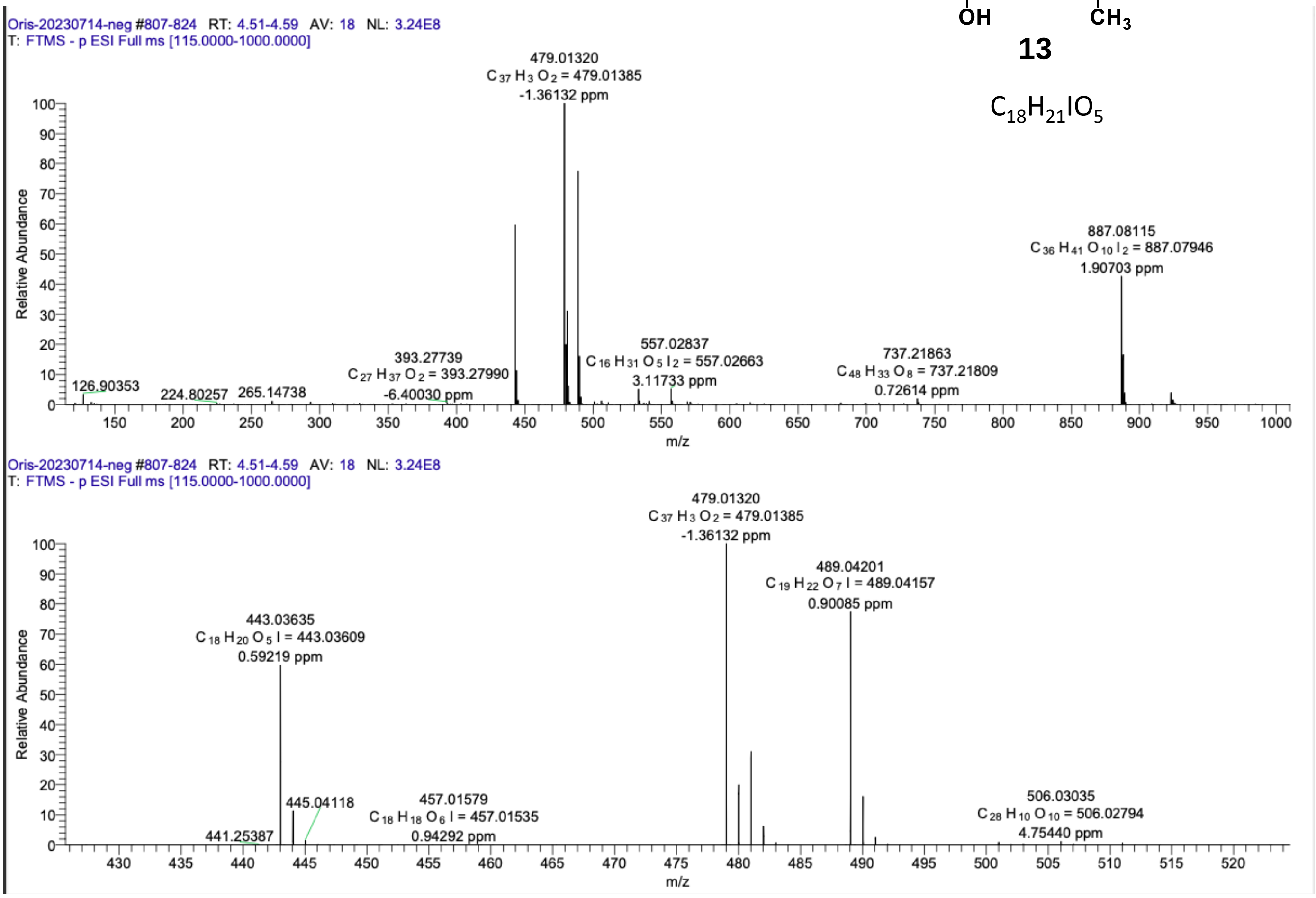

Figure S30. Compound **14**, HRMS in positive mode (above) and negative mode (below).

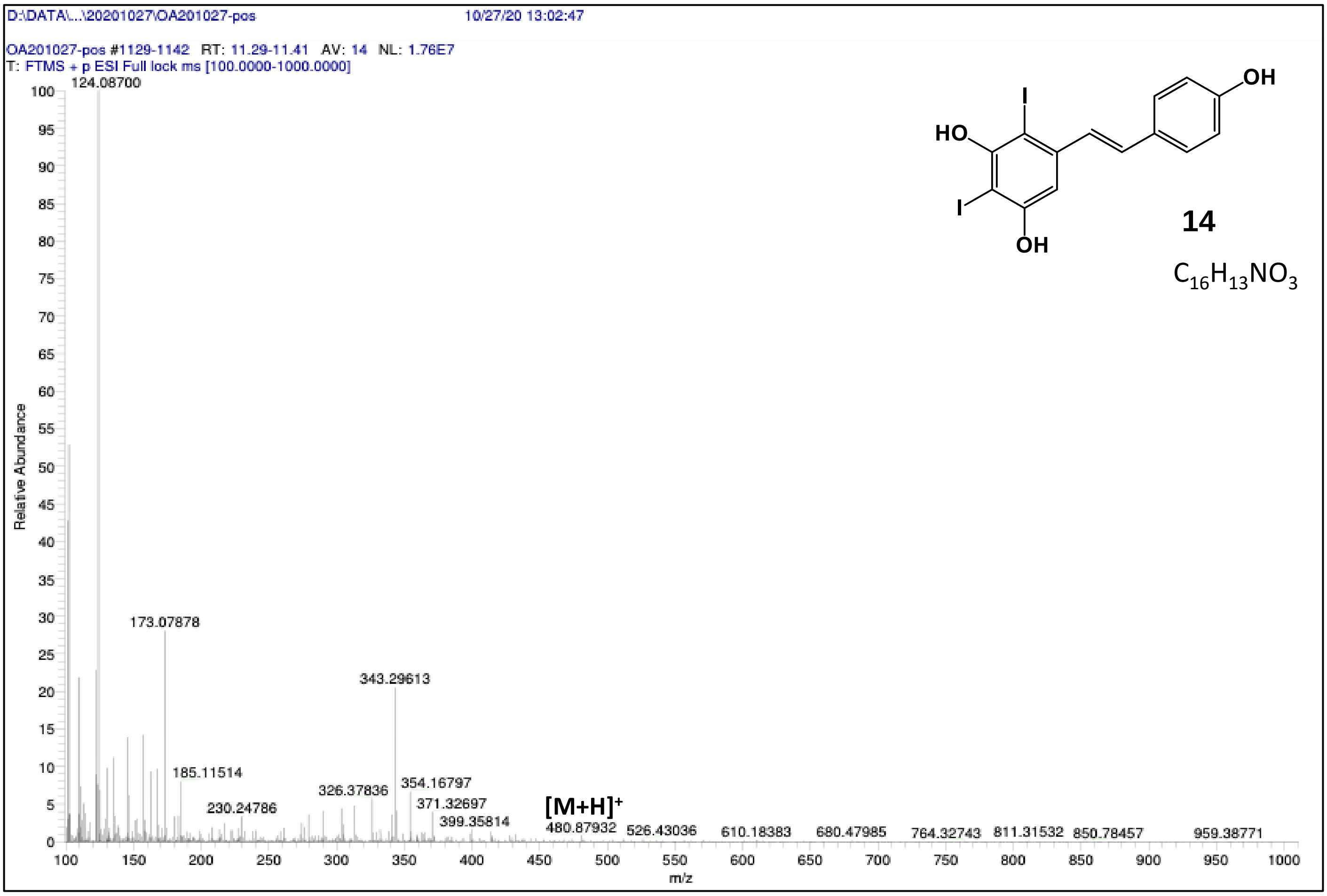

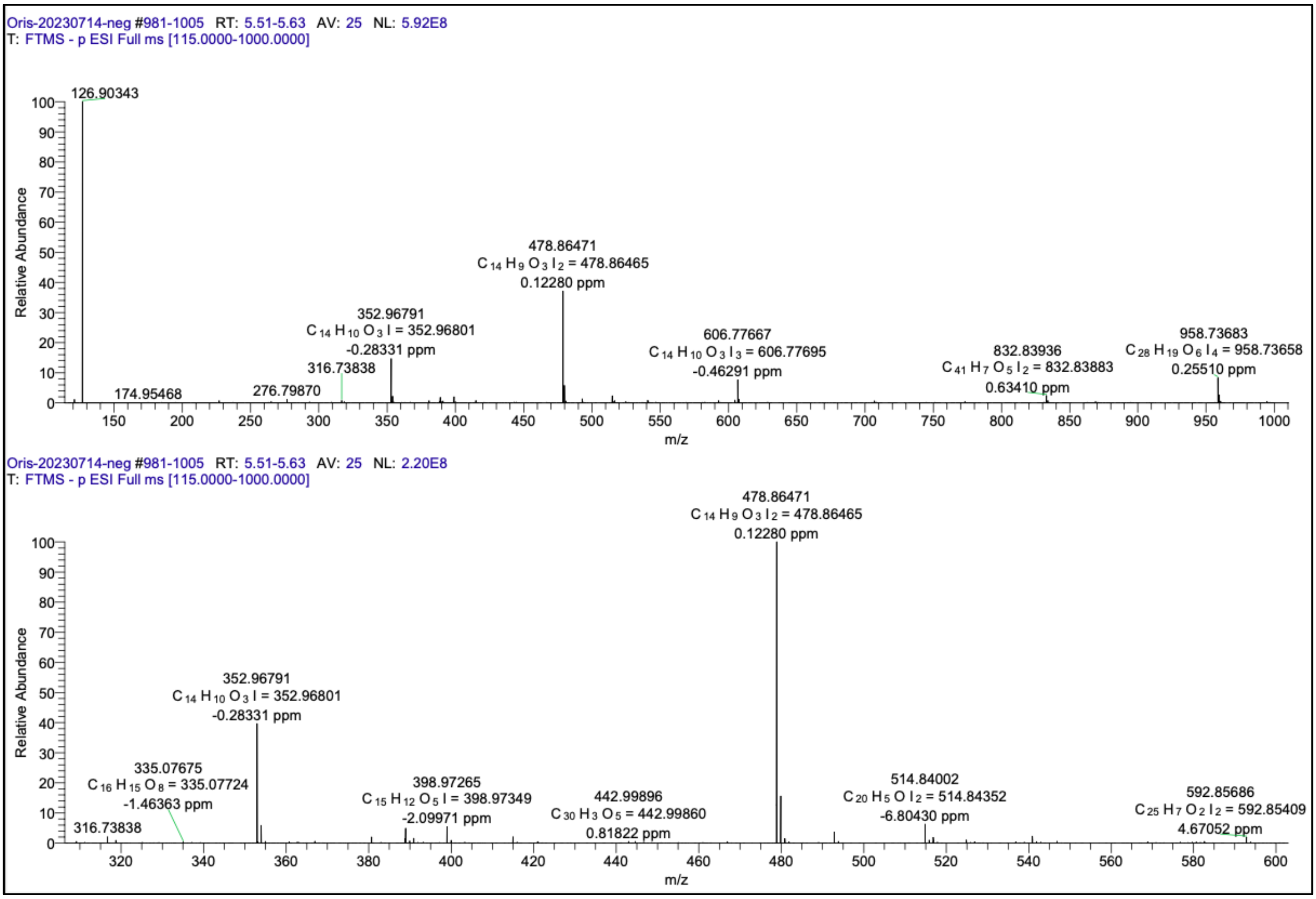

Figure S31. Compound **15**, HRMS (above; negative mode) and Compound **17**, HRMS (below; positive mode).

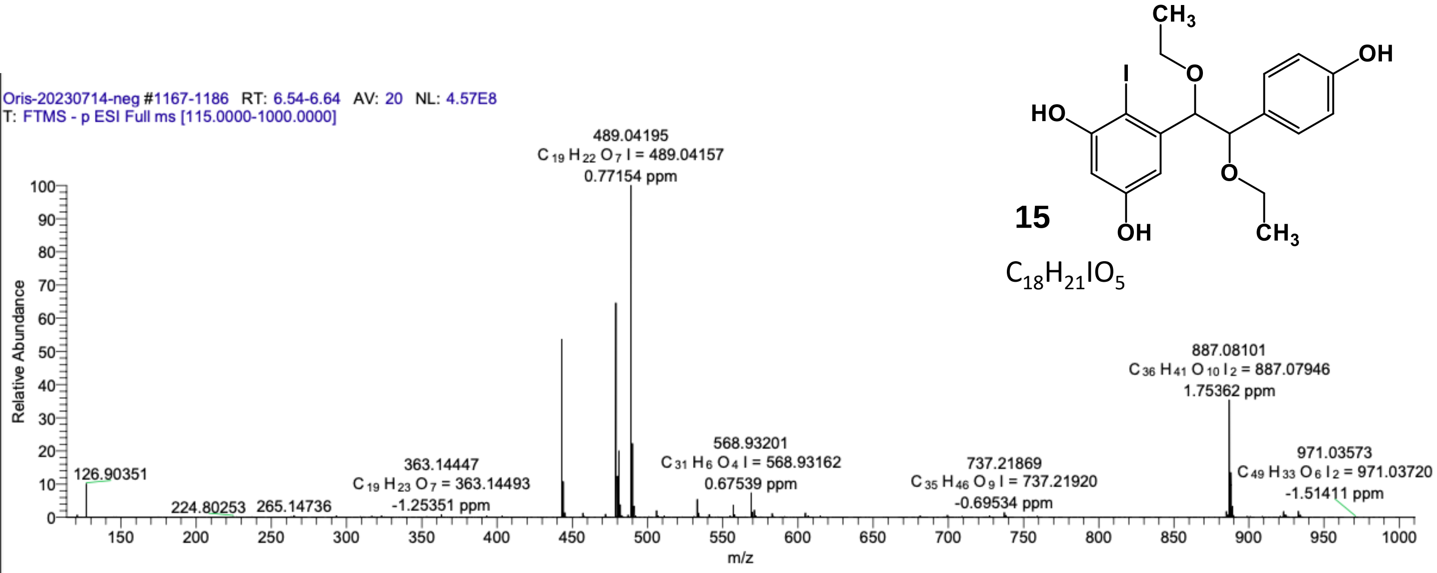

15

$C_{18}H_{21}IO_5$

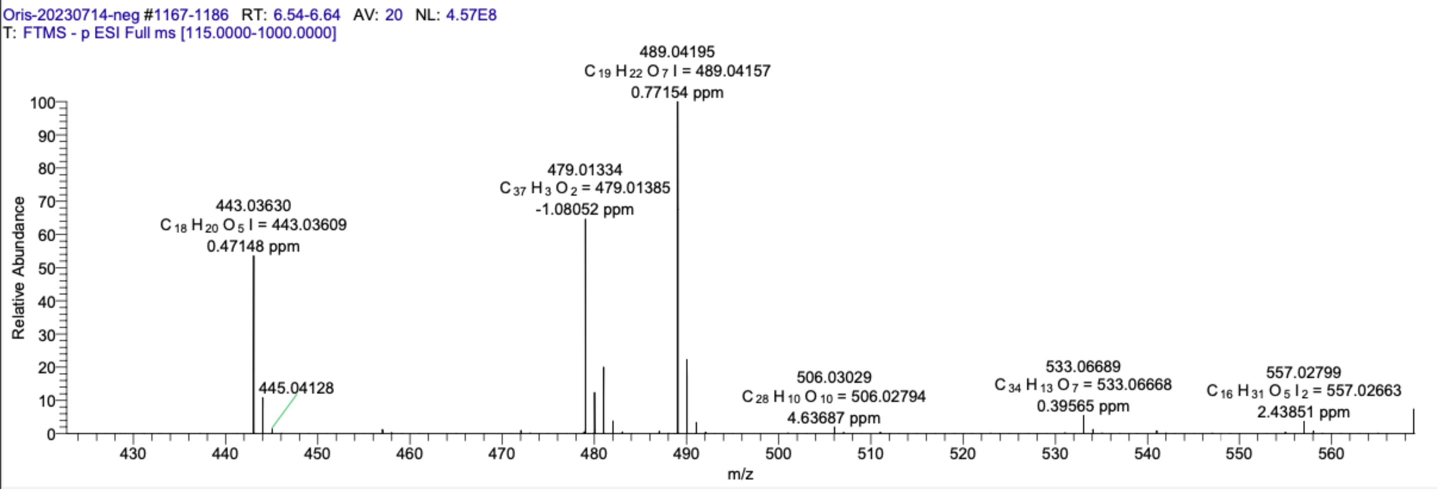

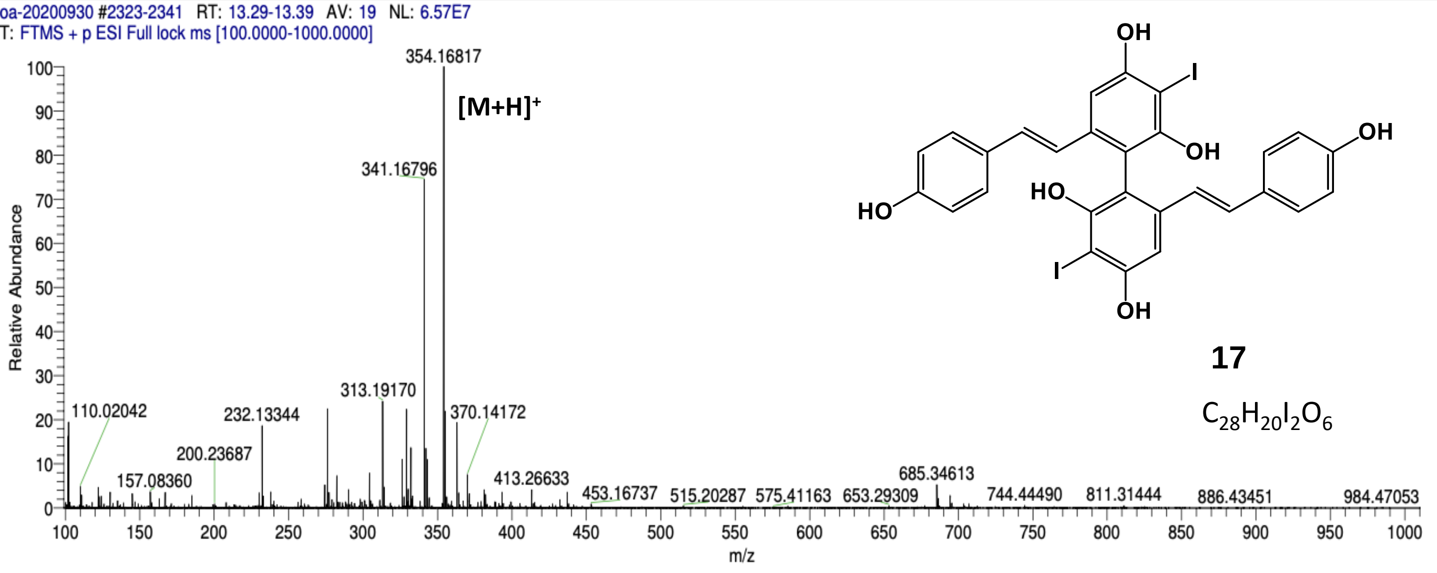

17

$C_{28}H_{20}I_2O_6$

Figure S32. Compound **18**, HRMS (above; negative mode) and Compound **19**, HRMS (below; negative mode).

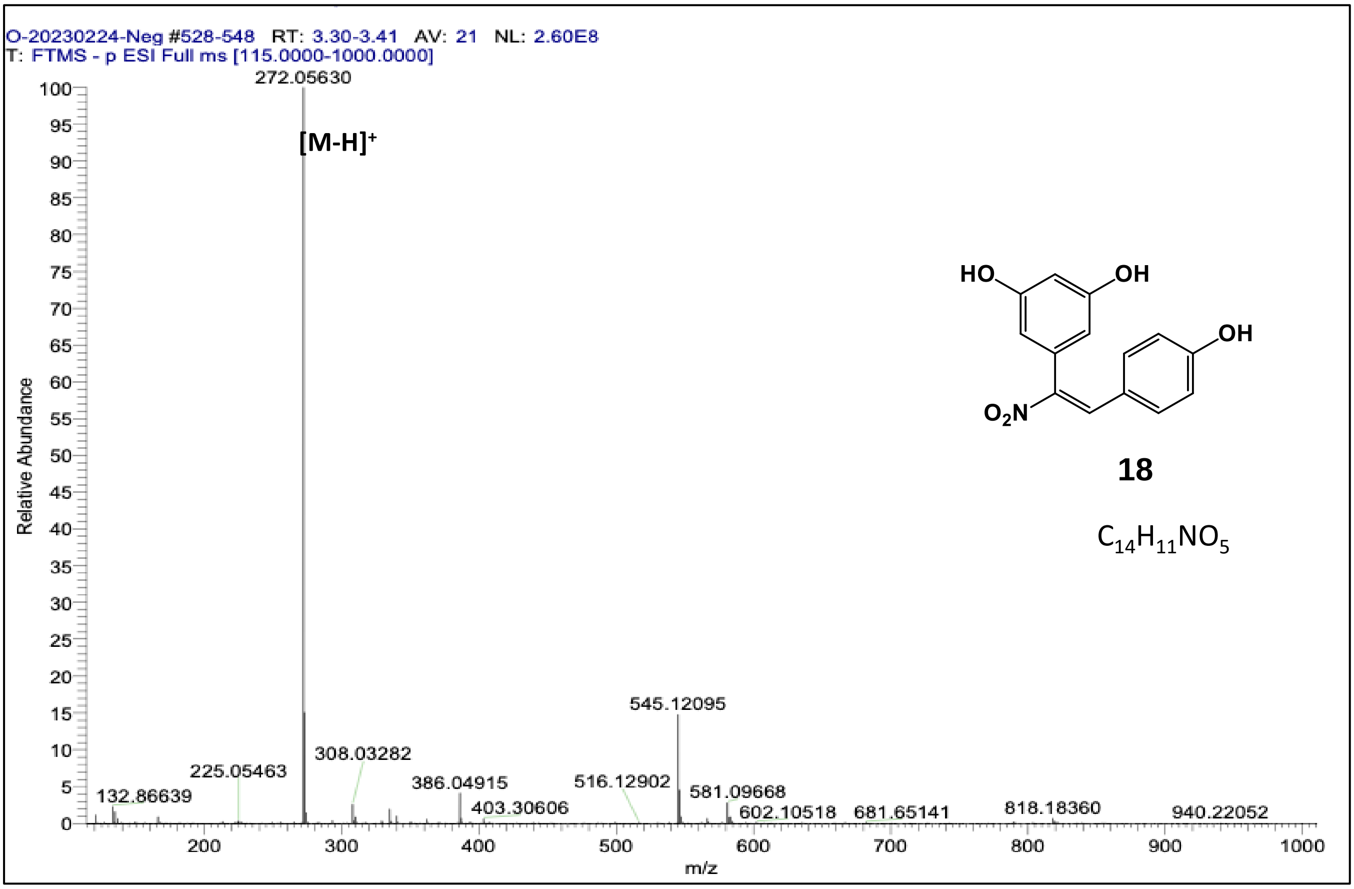

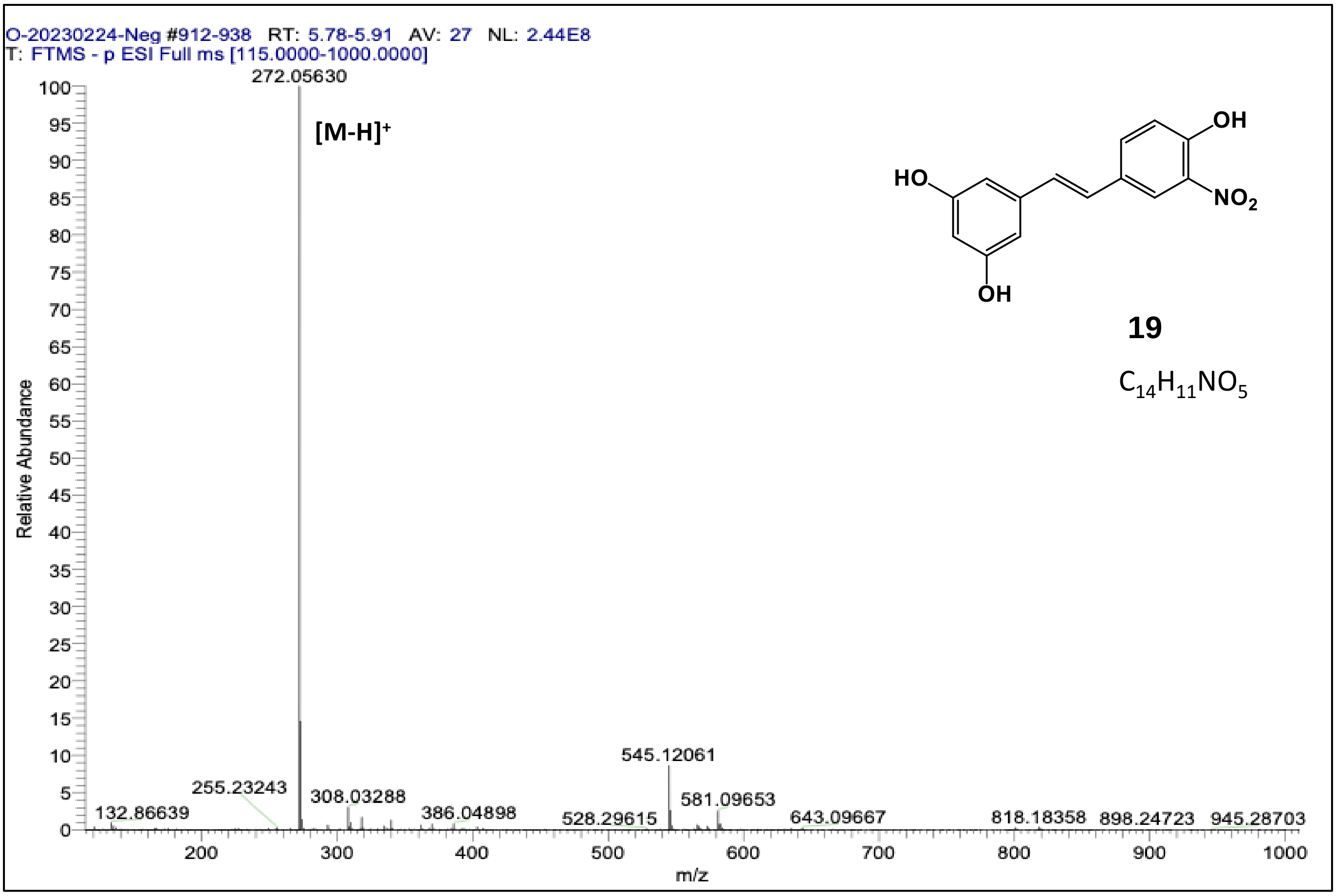

Figure S33. Compound **1**, ^{1}H NMR and ^{13}C APT NMR spectra.

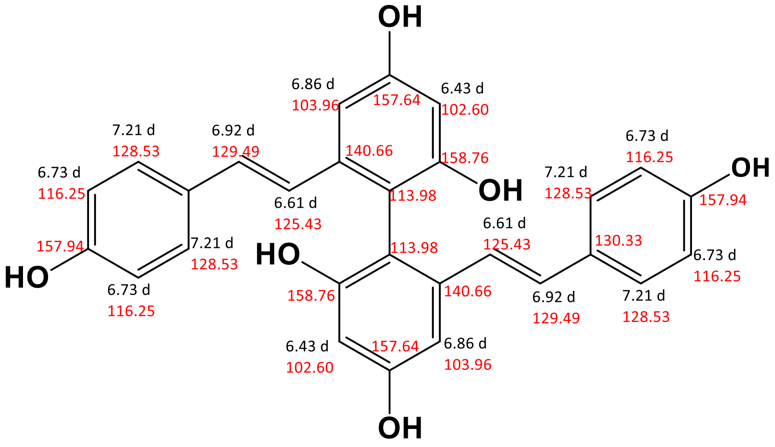

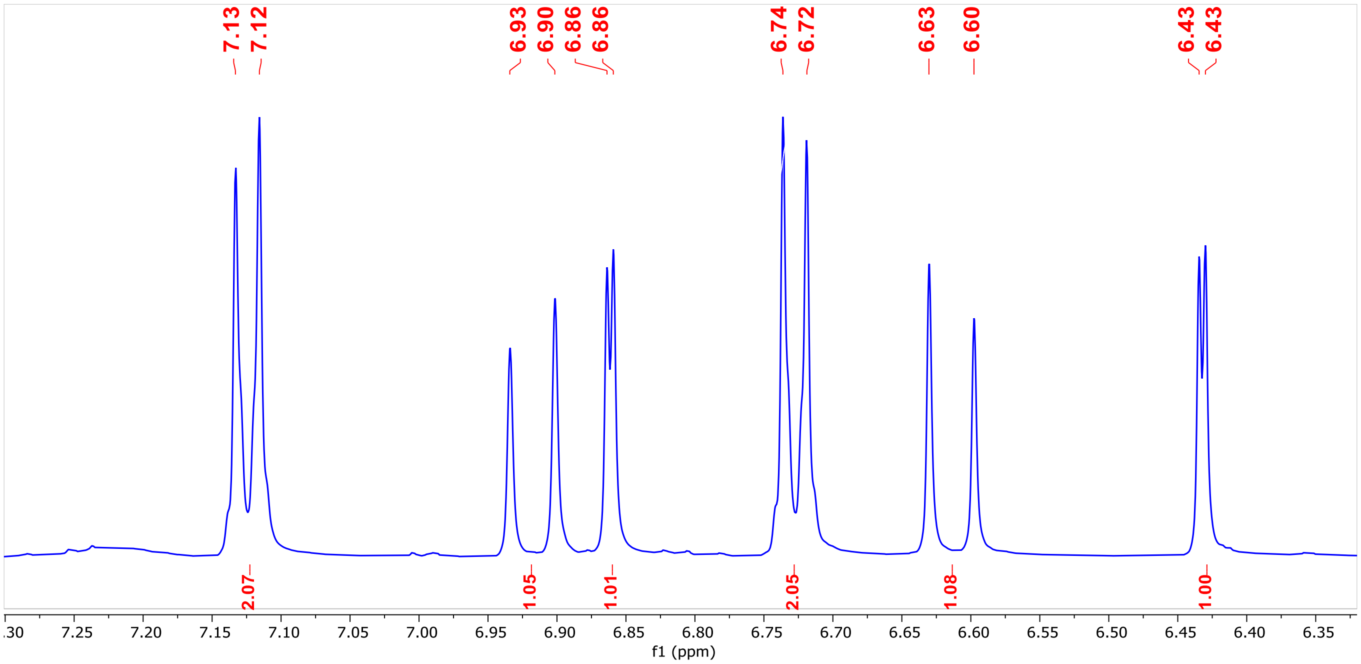

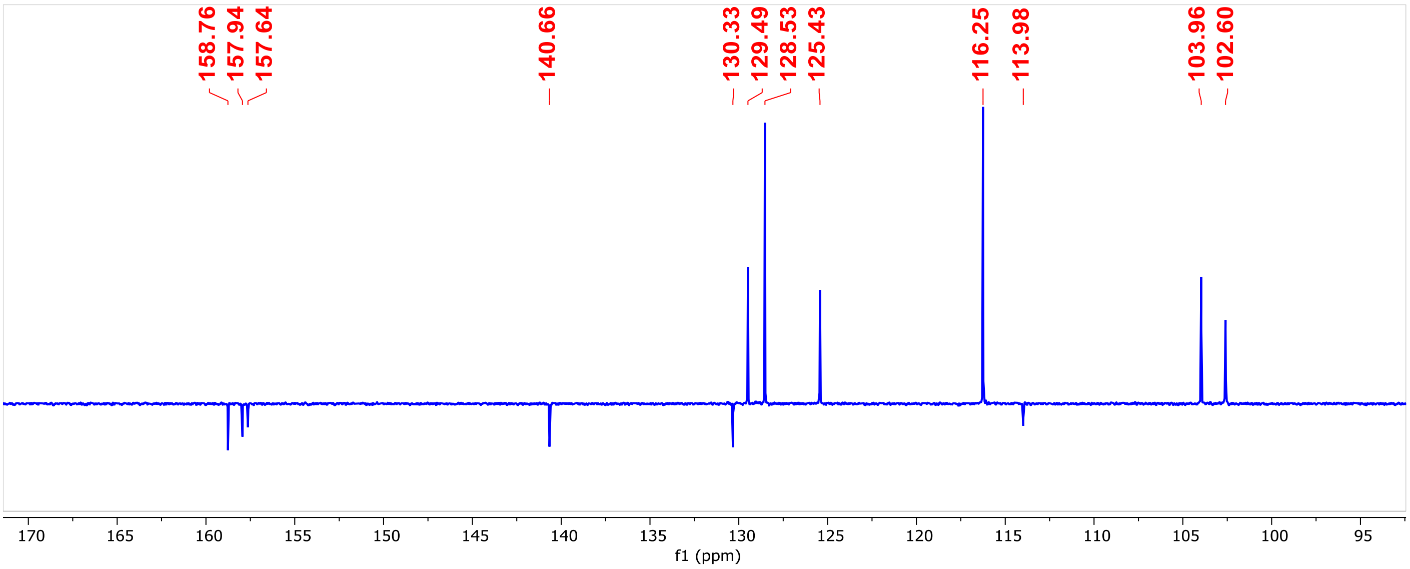

Figure S34. Compound **1**, HSQC and HMBC spectra.

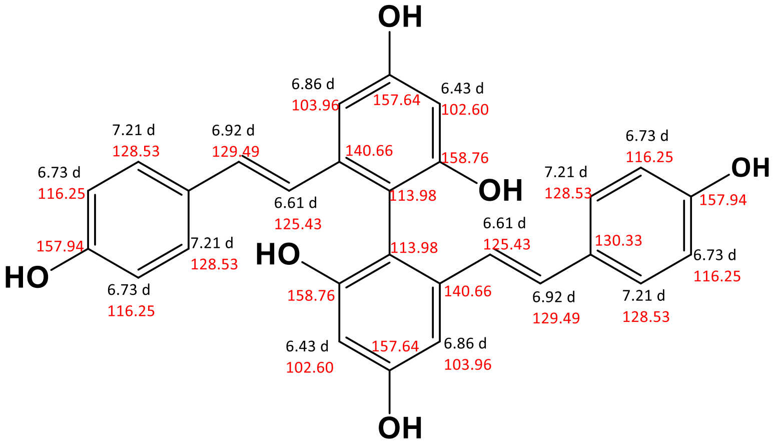

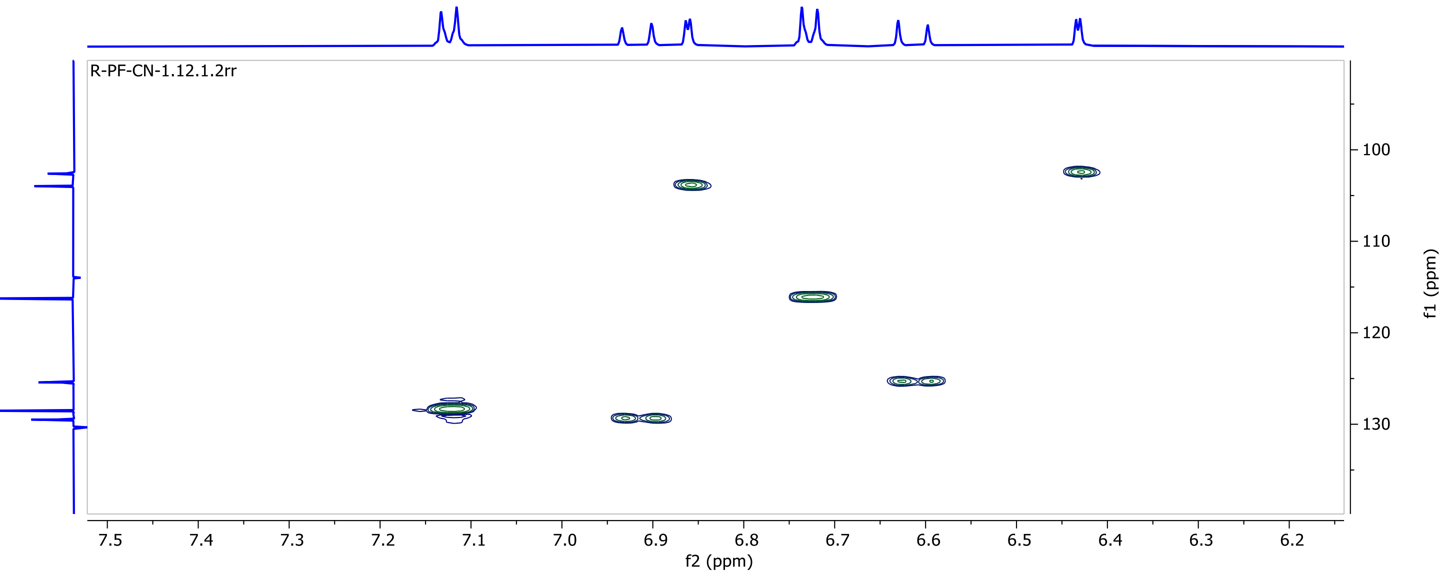

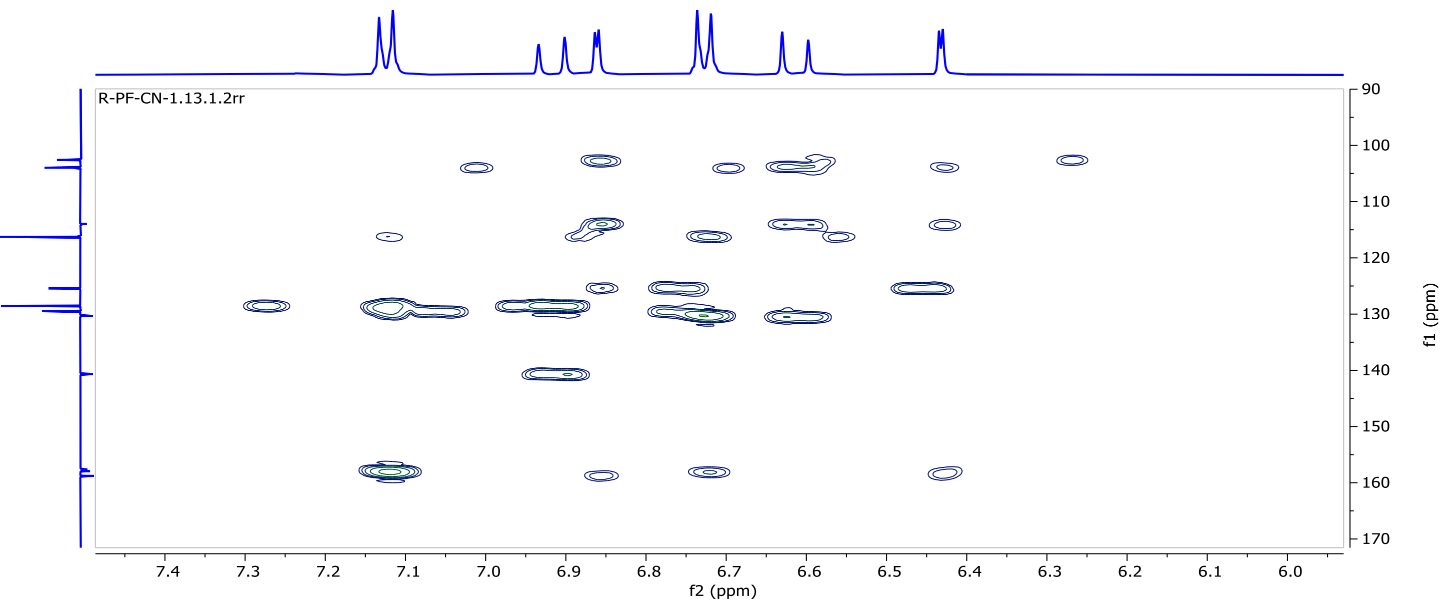

Figure S35. Compound **1**, NOESY and COSY spectra.

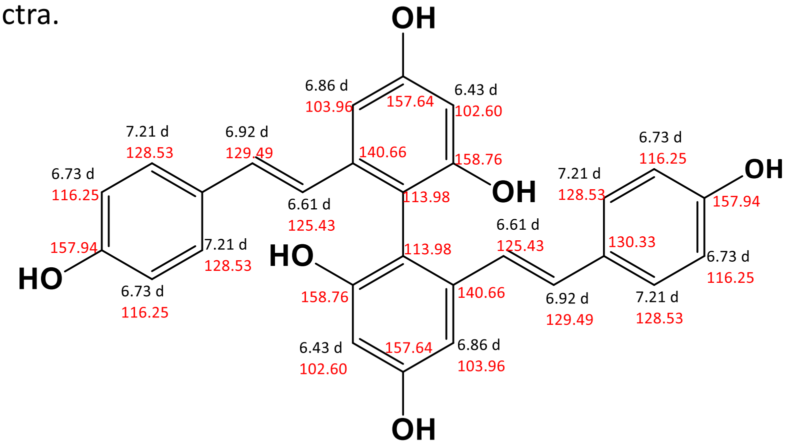

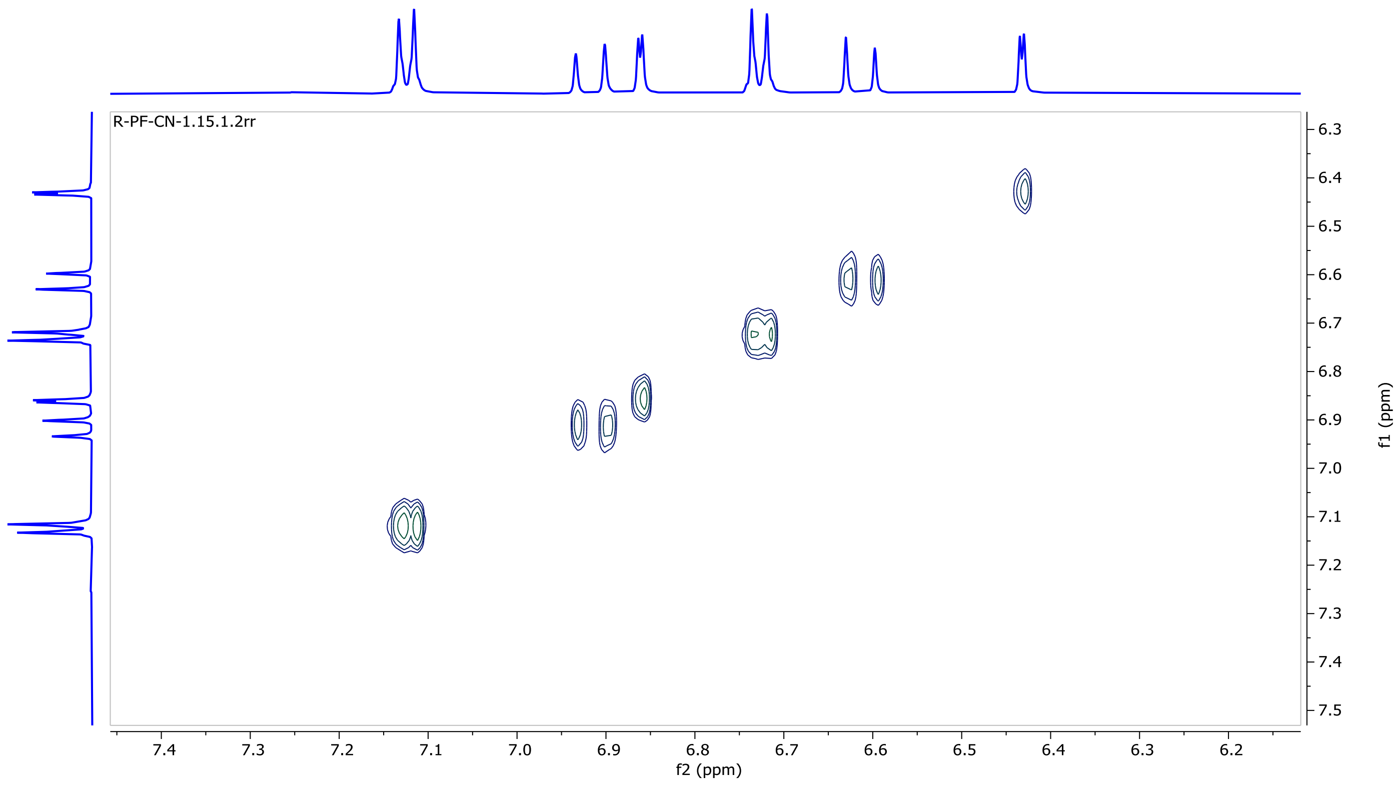

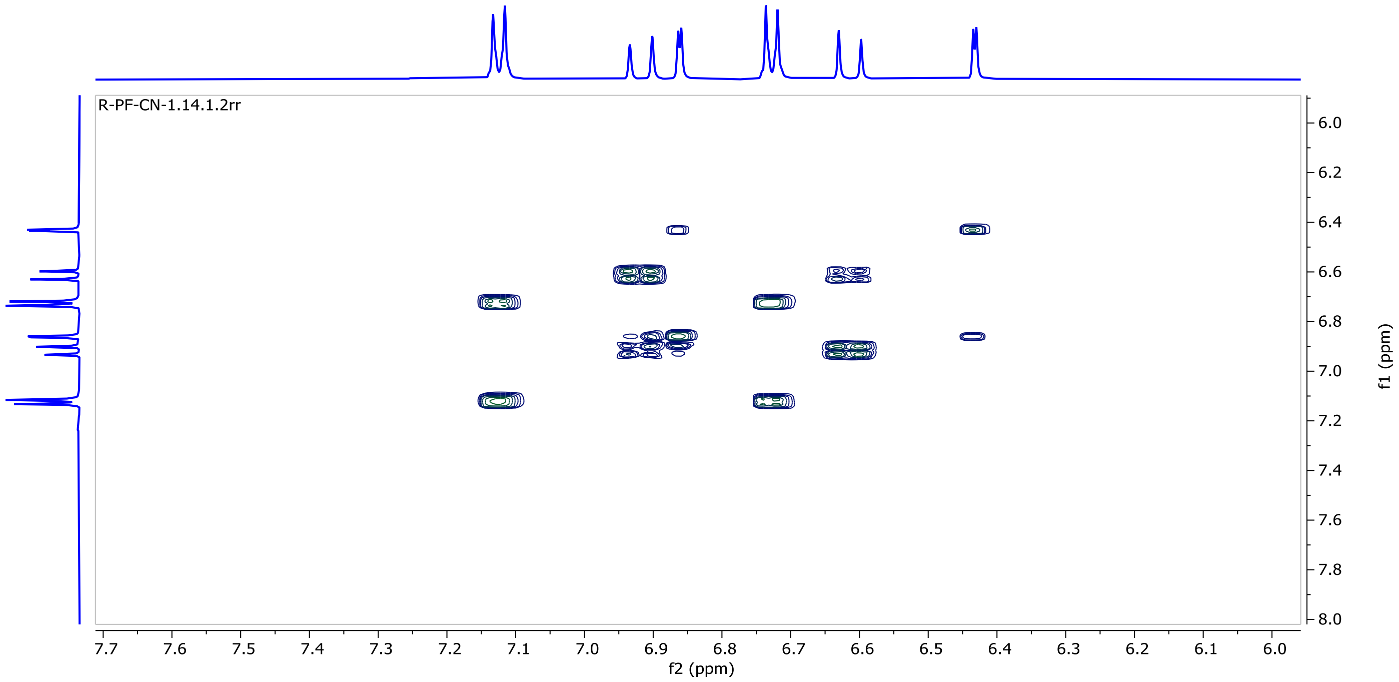

Figure S36. Compound **3**, ^{1}H NMR and ^{13}C, APT NMR spectra.

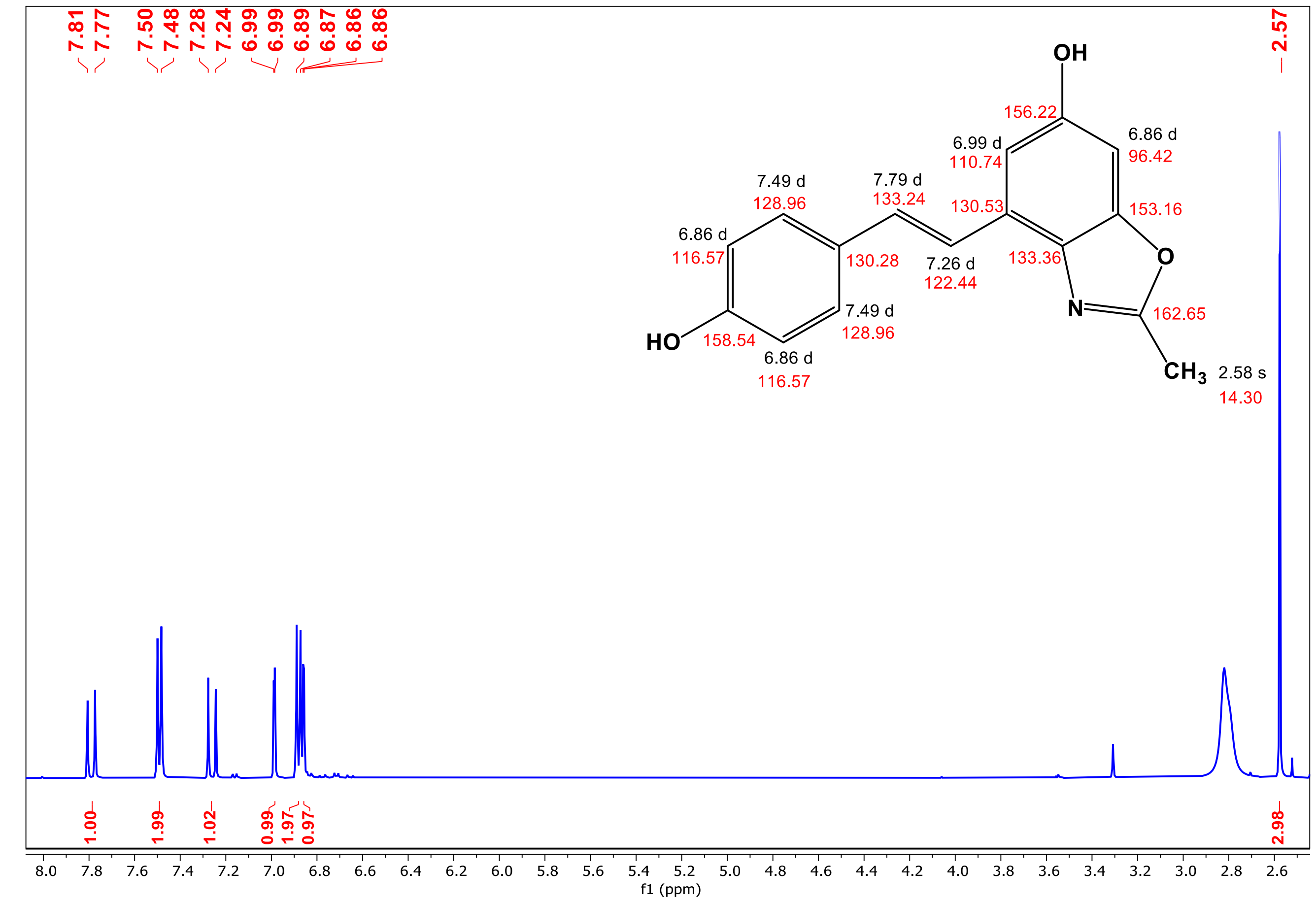

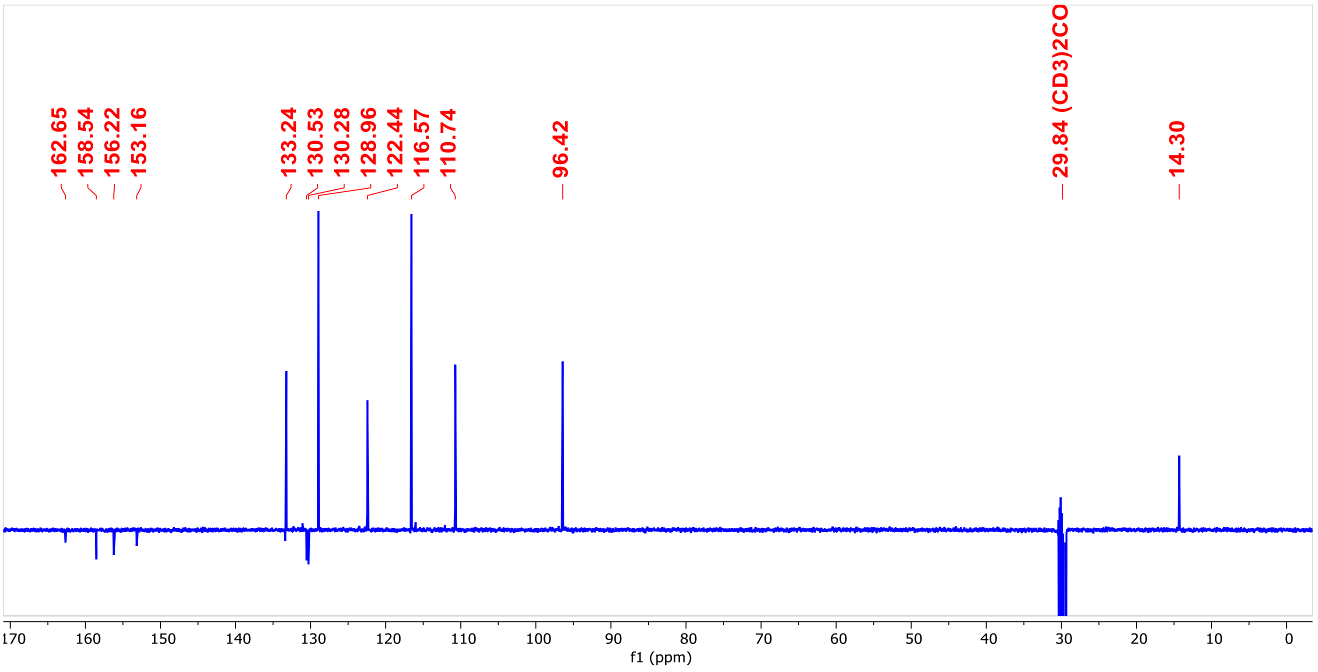

Figure S37. Compound **3**, HSQC and HMBC spectra.

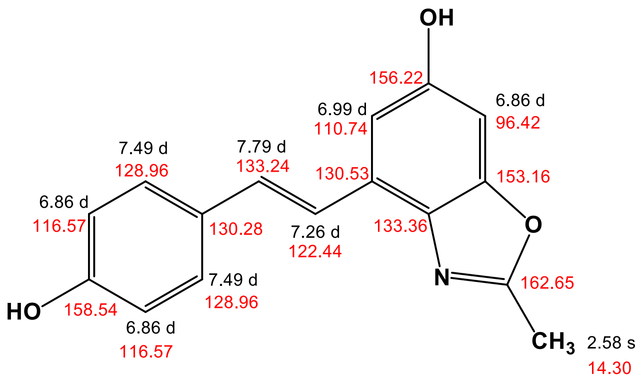

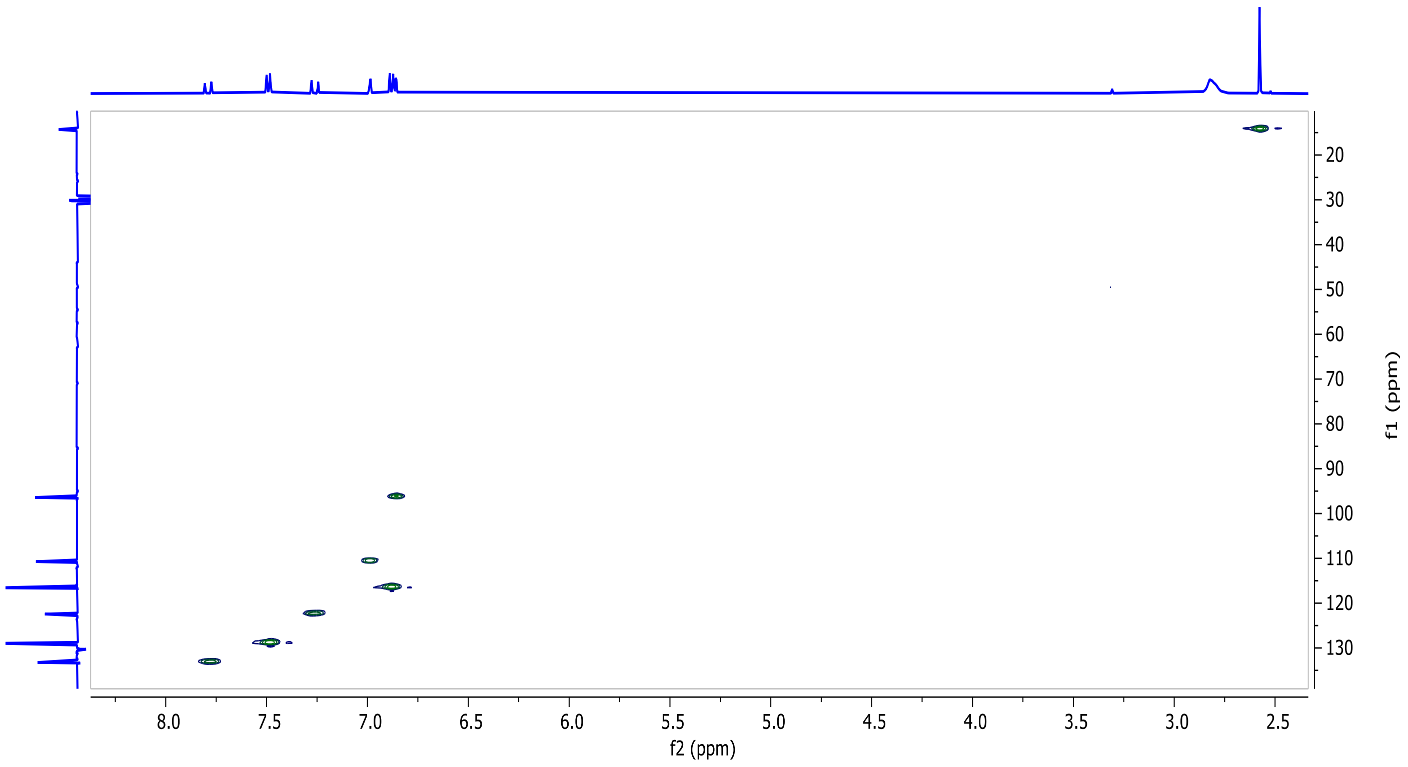

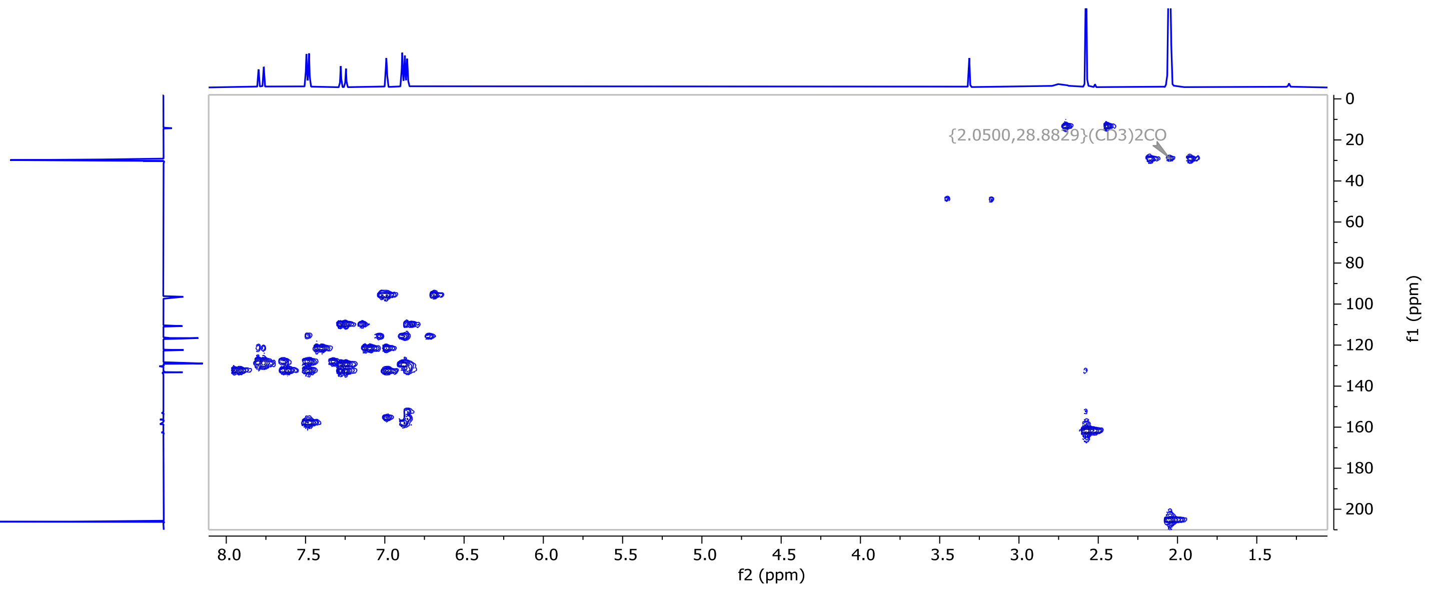

Figure S38. Compound **3**, COSY and NOESY spectra.

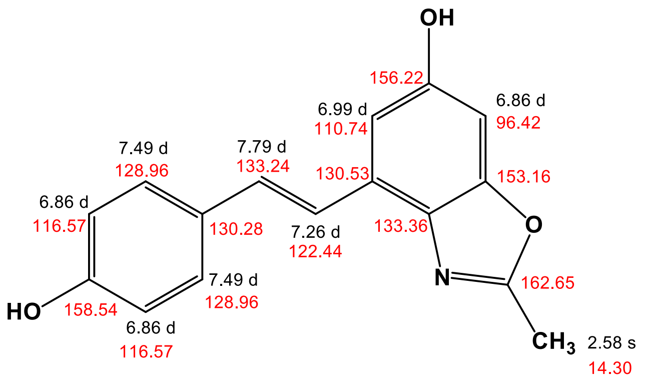

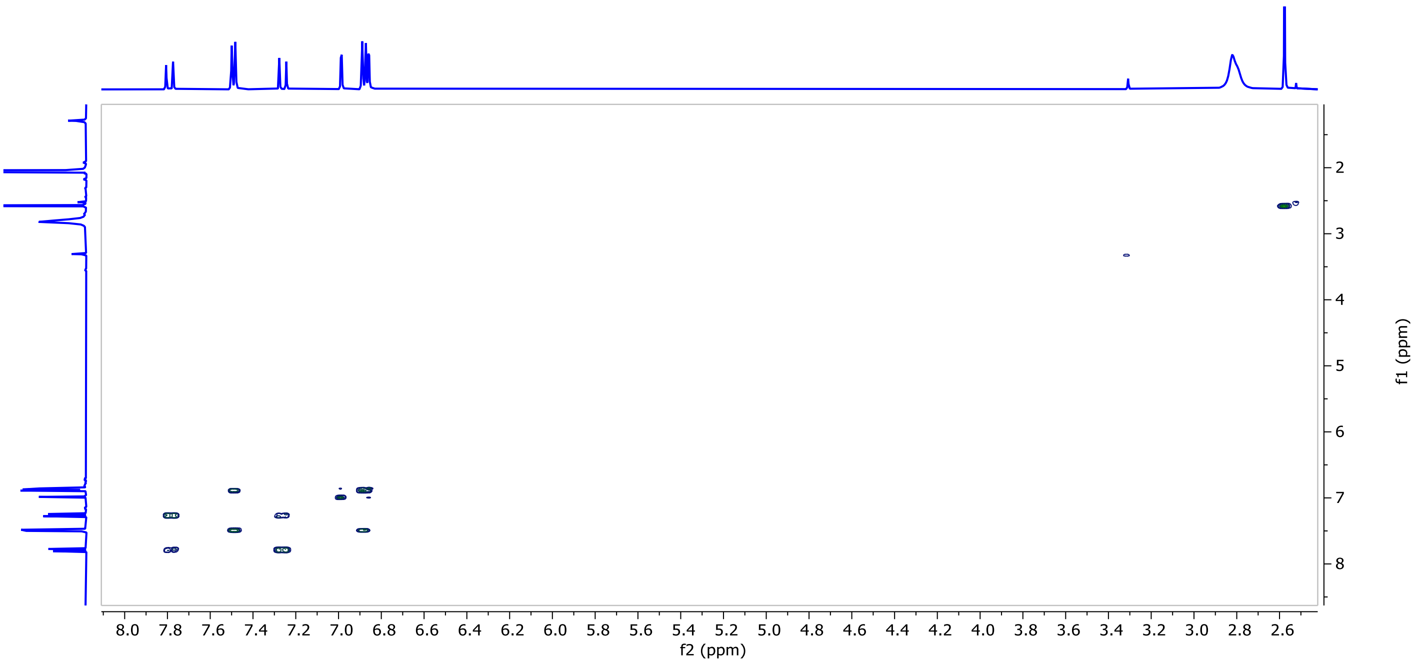

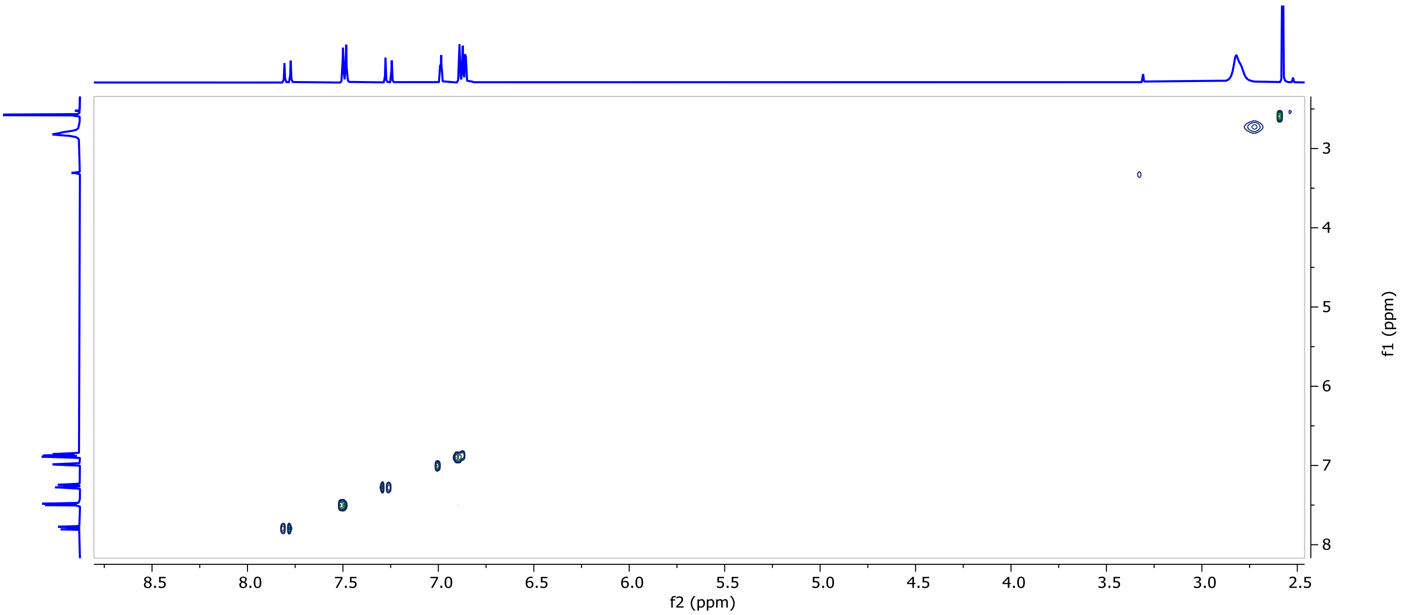

Figure S39. Compound **4**, ^{1}H NMR and ^{13}C APT NMR spectra.

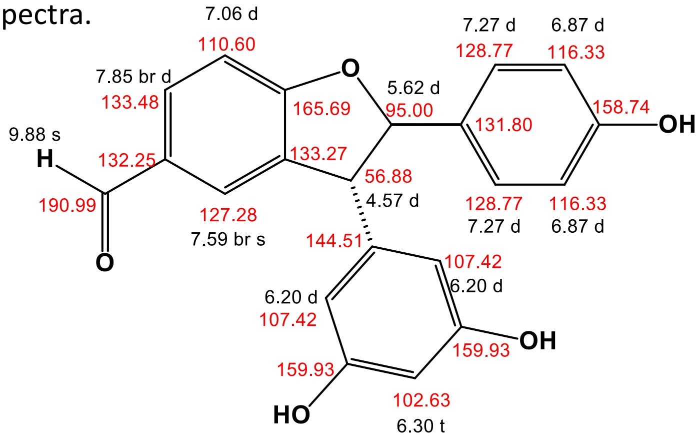

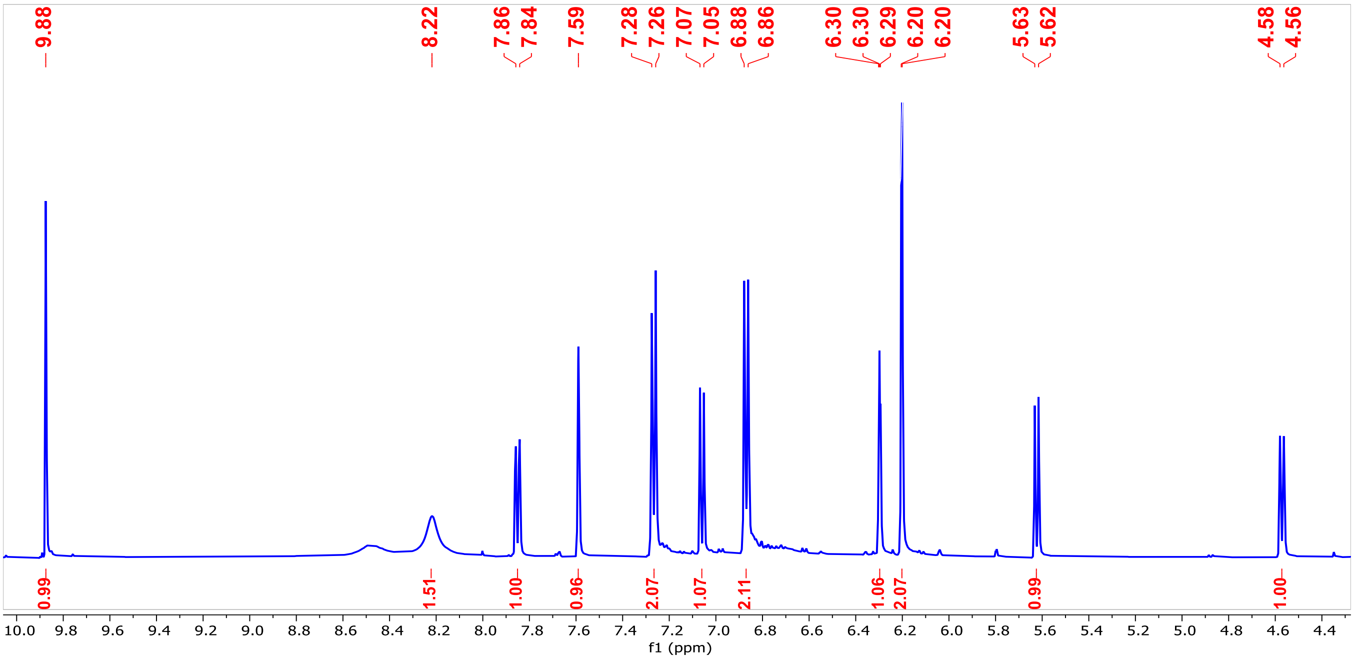

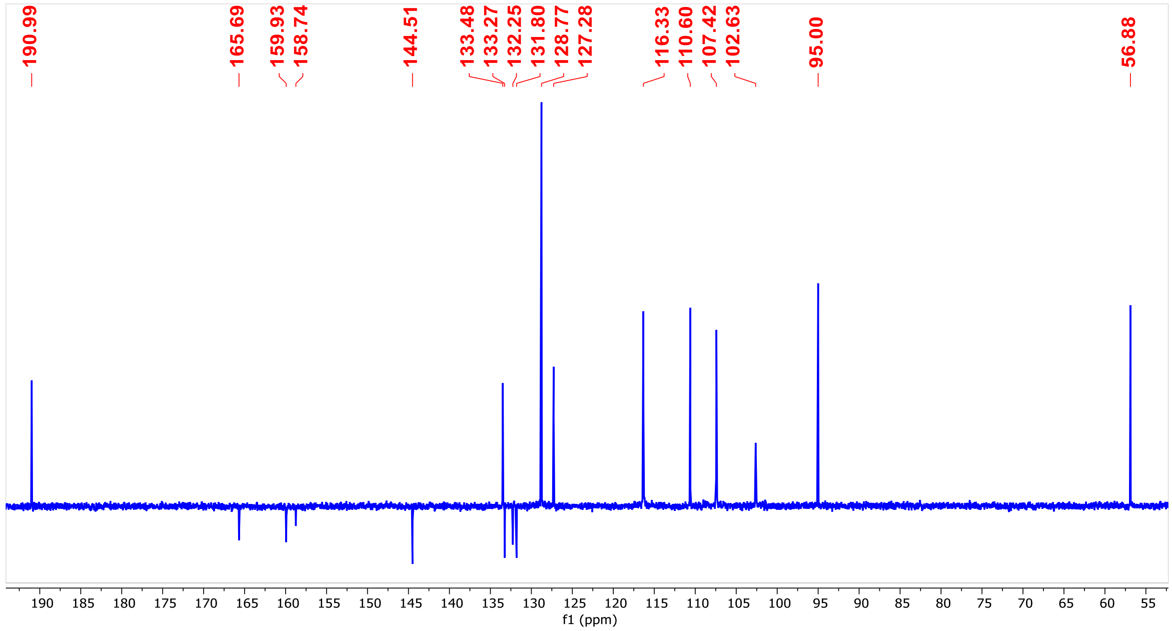

Figure S40. Compound **4**, HSQC and HMBC spectra.

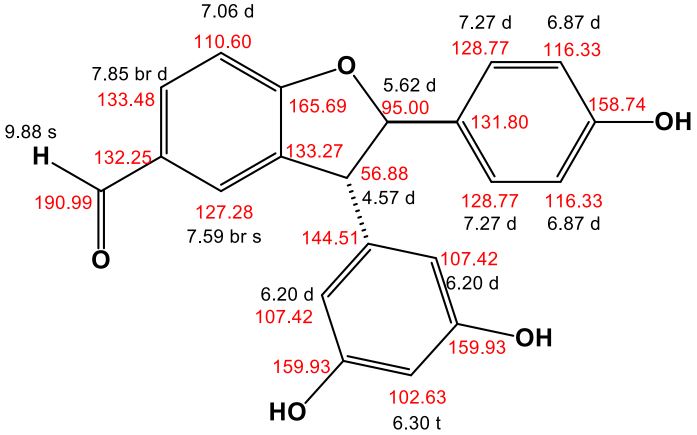

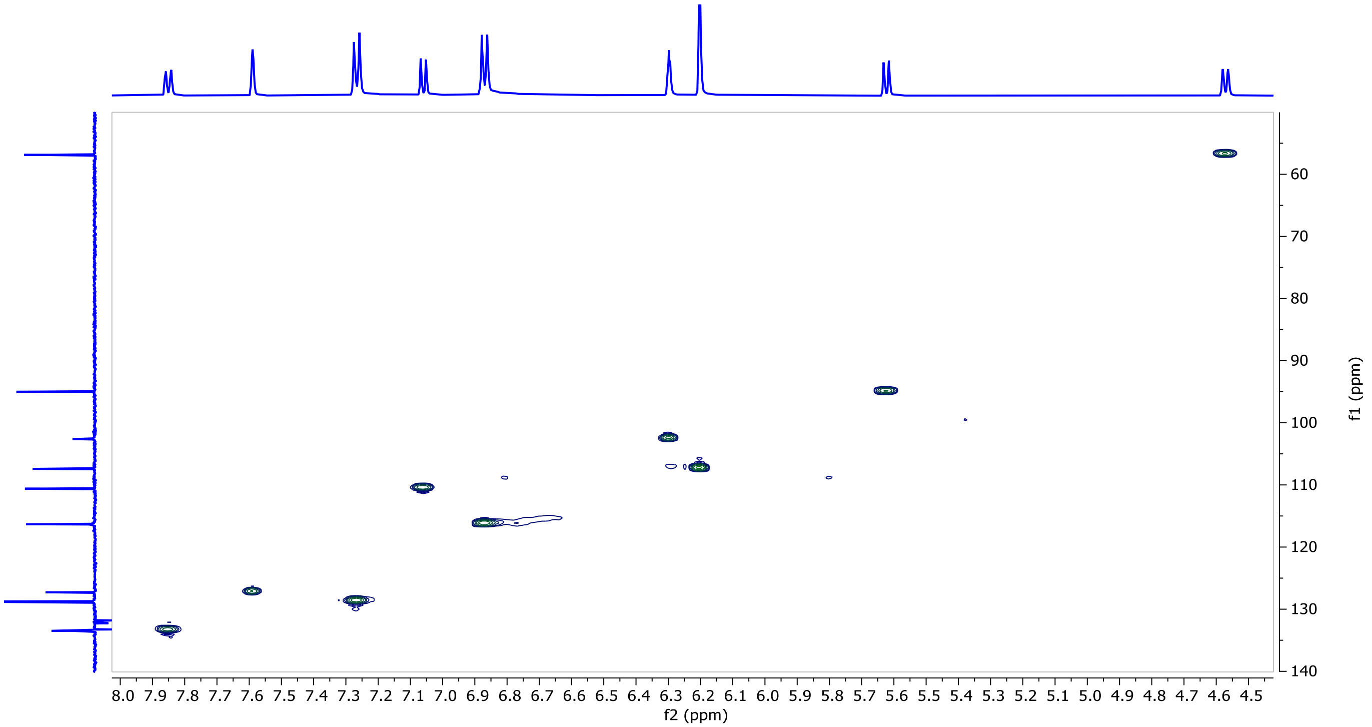

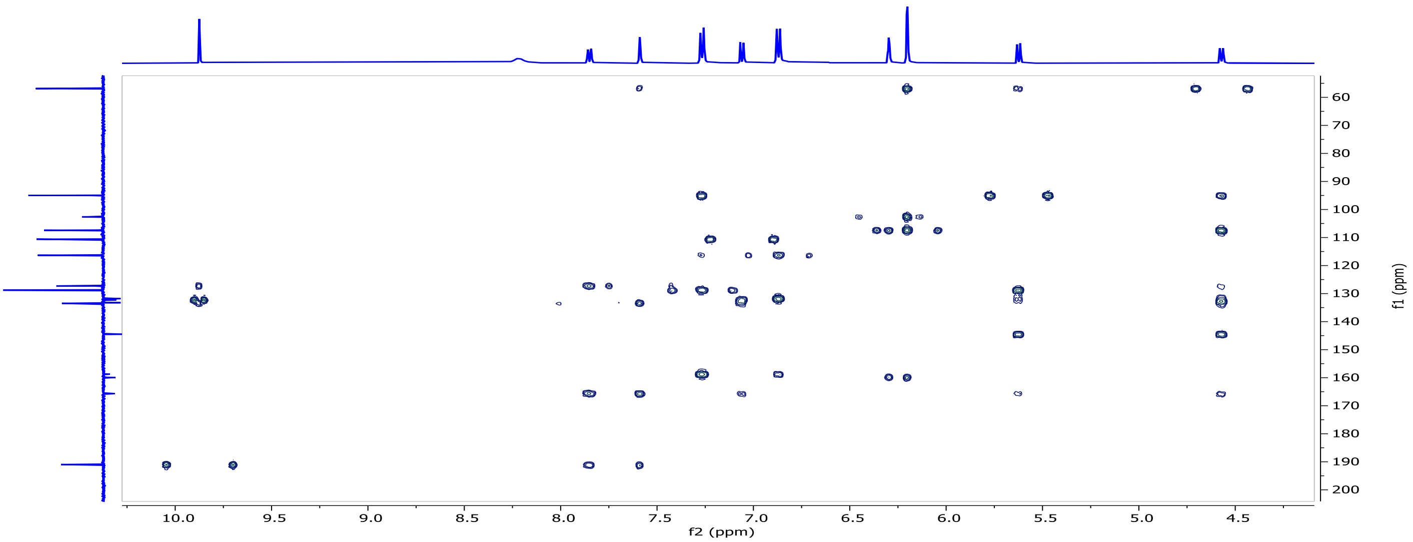

Figure S41. Compound **4**, COSY and NOESY spectra.

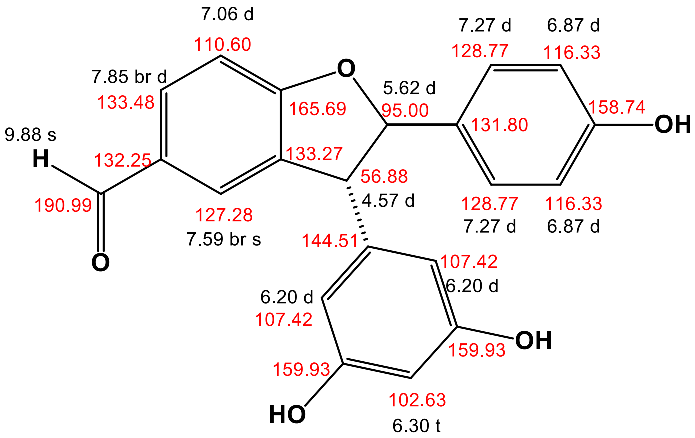

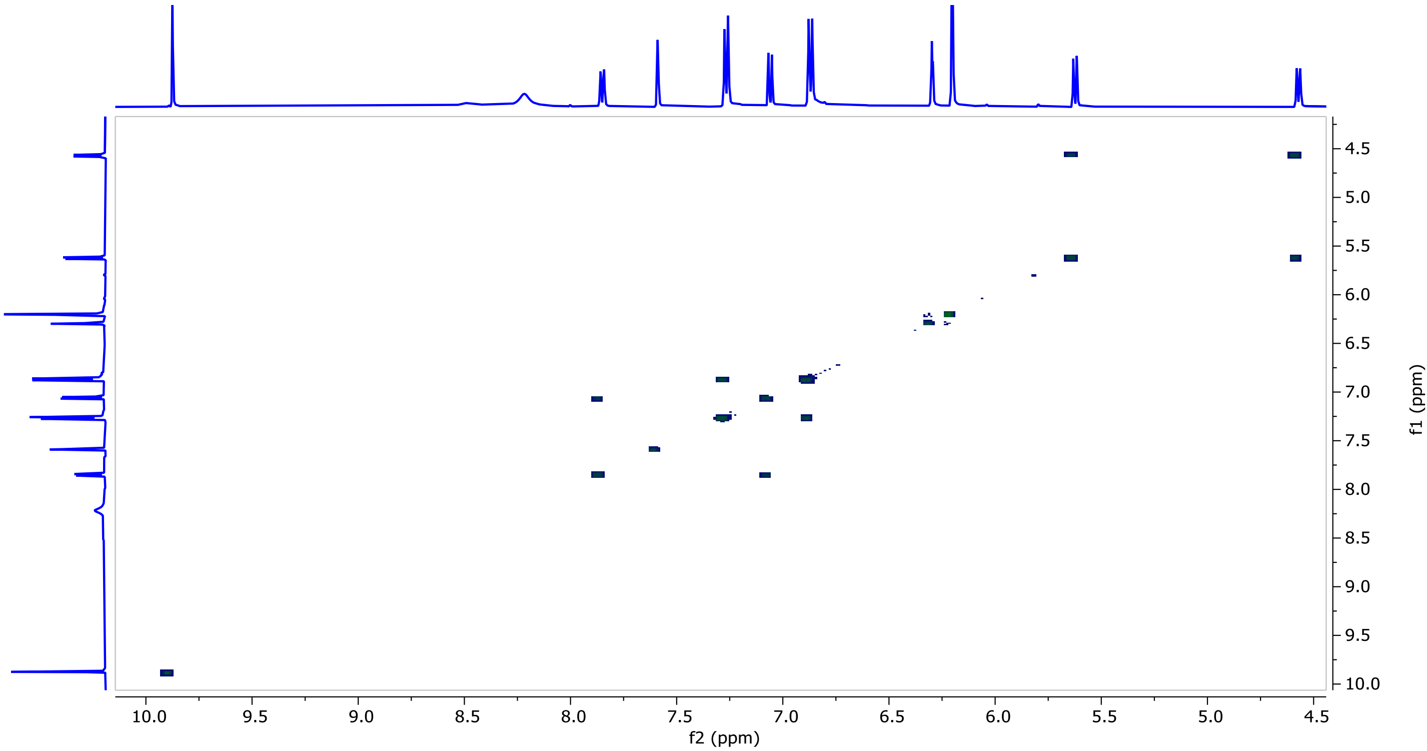

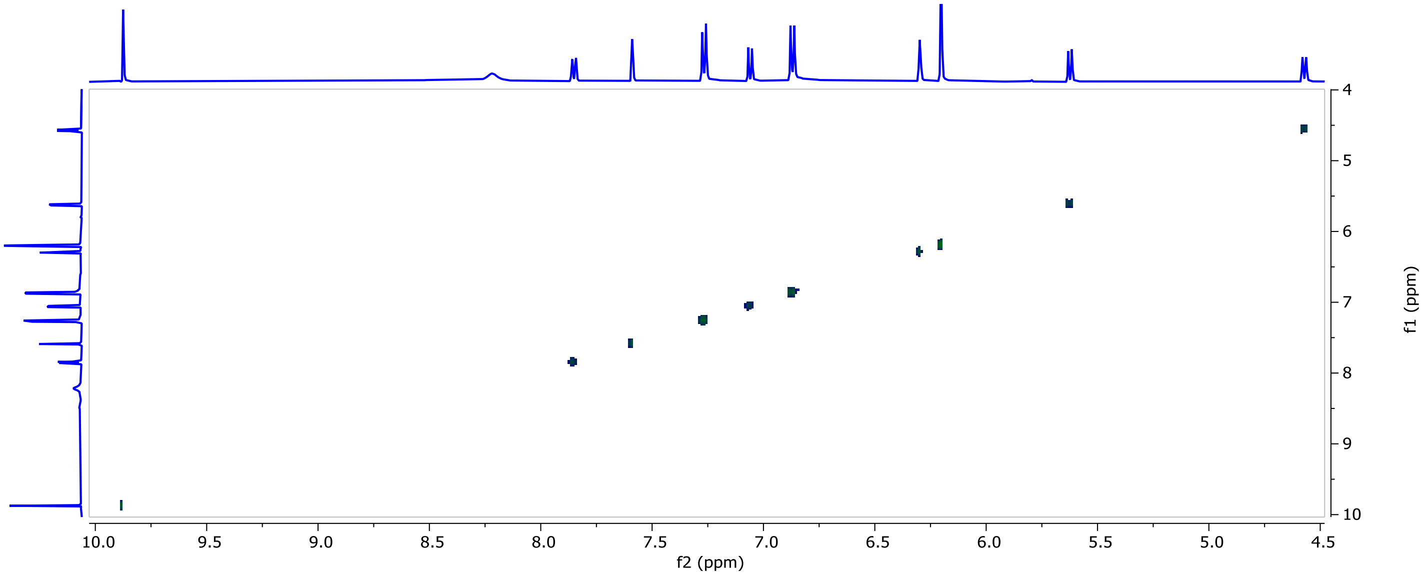

Figure S42. Compound **7**, ^{1}H NMR and ^{13}C, APT NMR spectra.

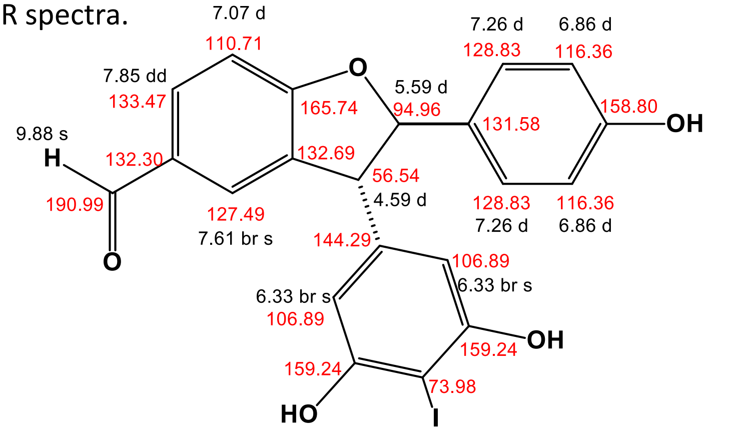

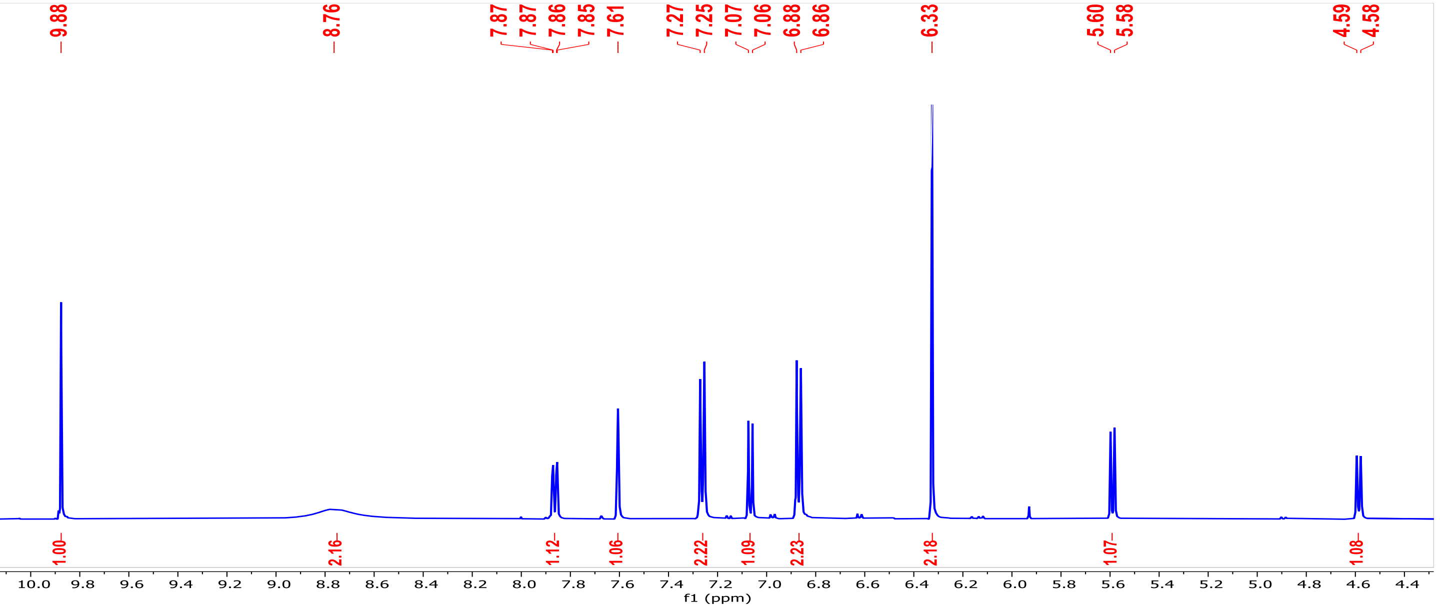

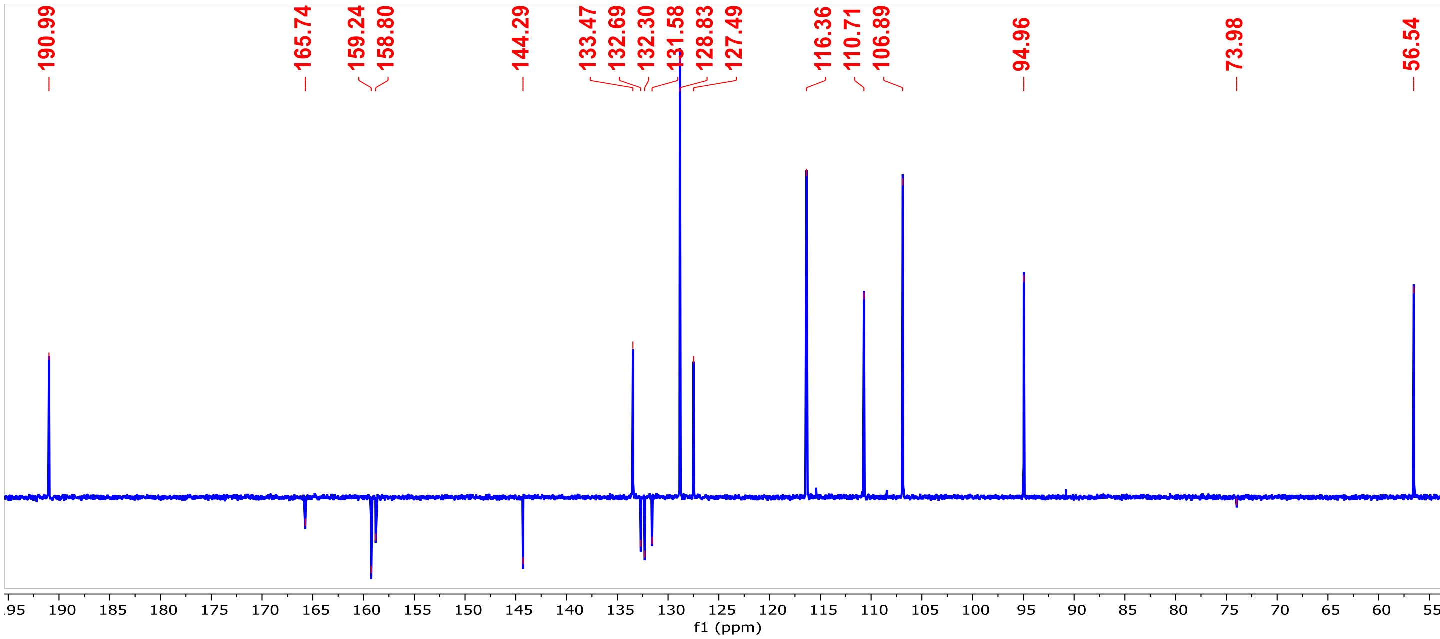

Figure S43. Compound **7**, HSQC and HMBC spectra.

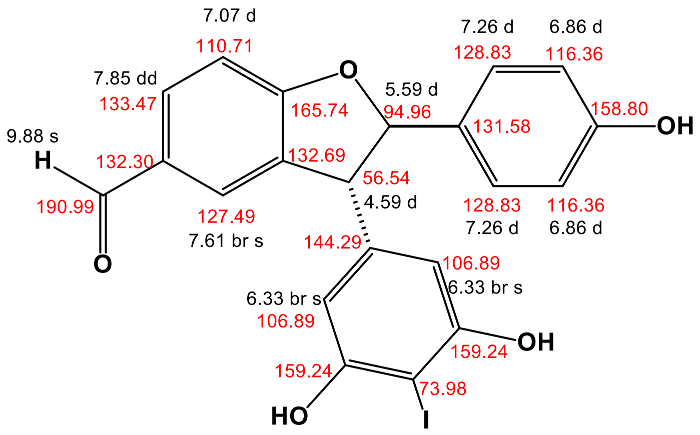

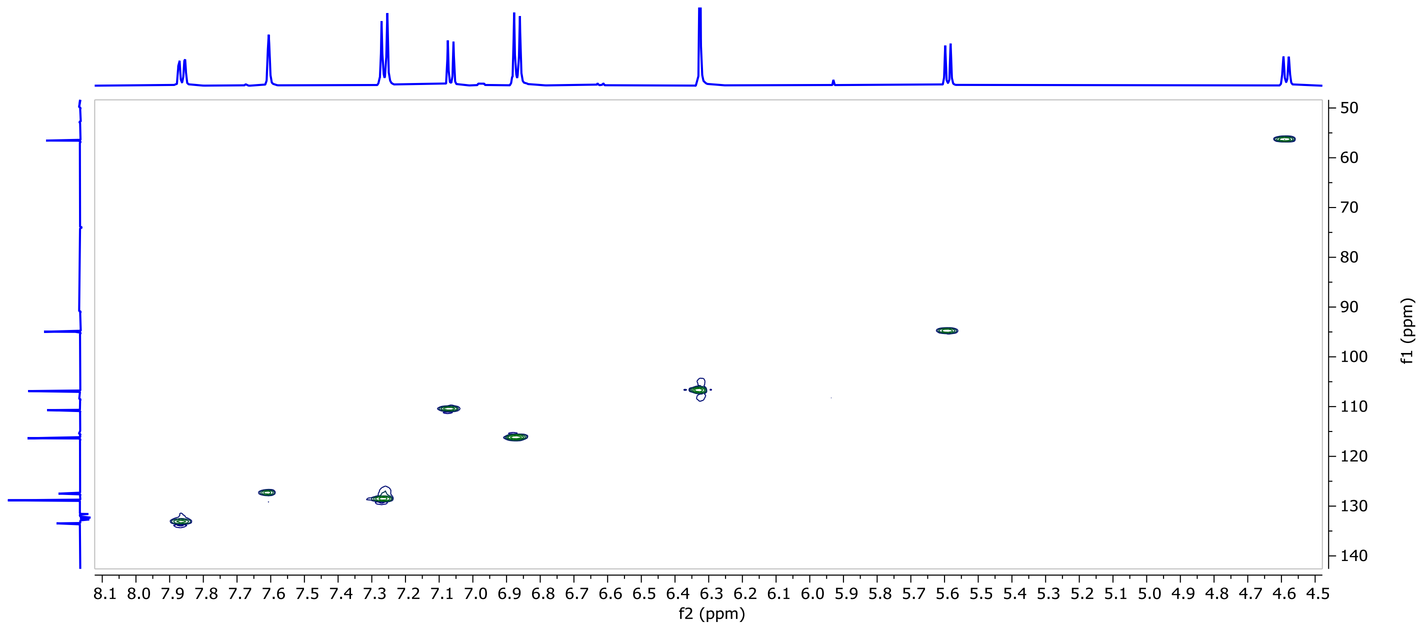

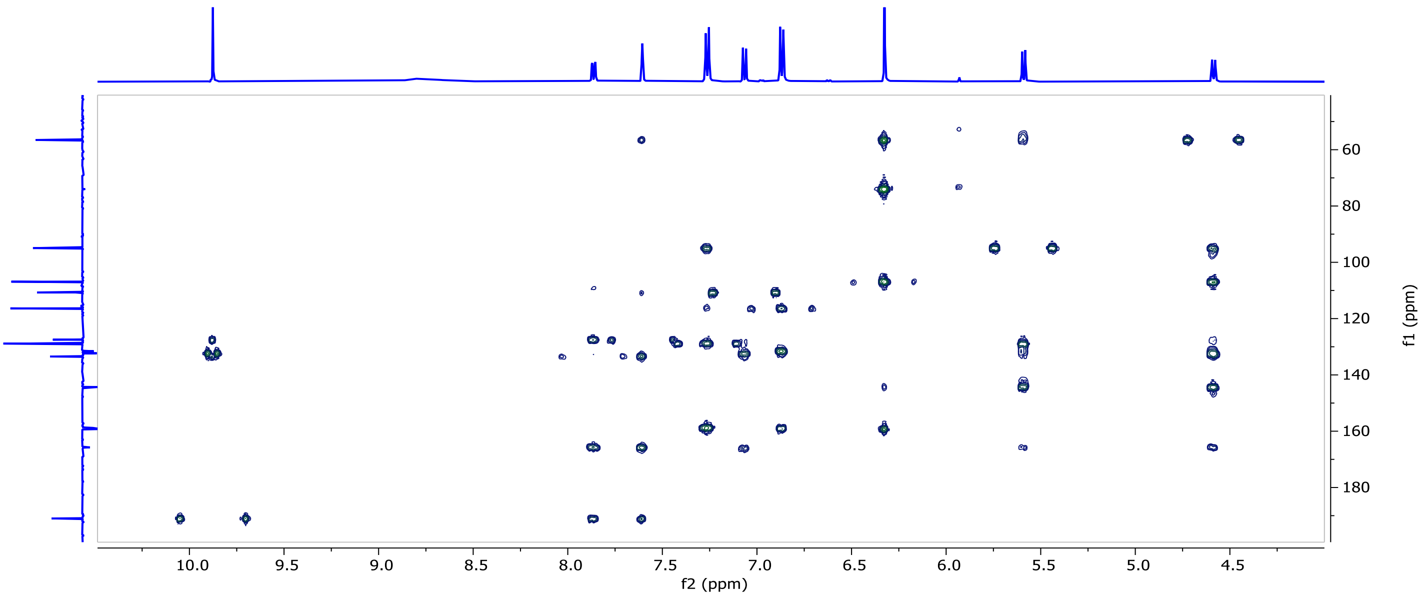

Figure S44. Compound **7**, COSY and NOESY spectra.

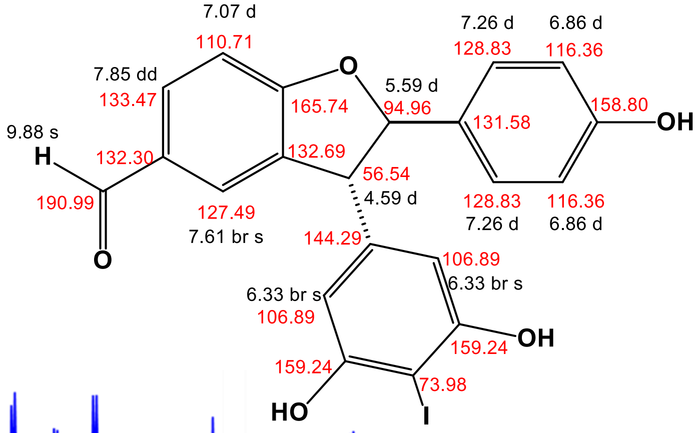

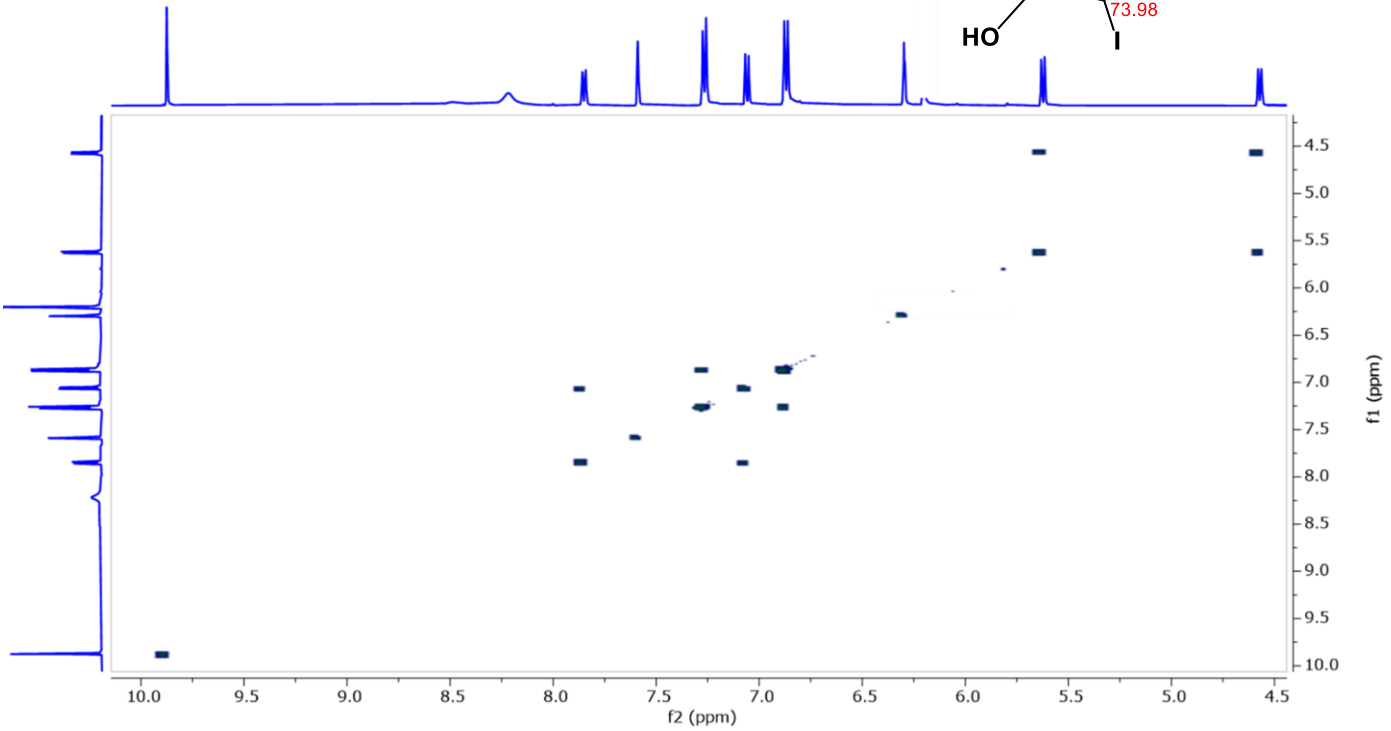

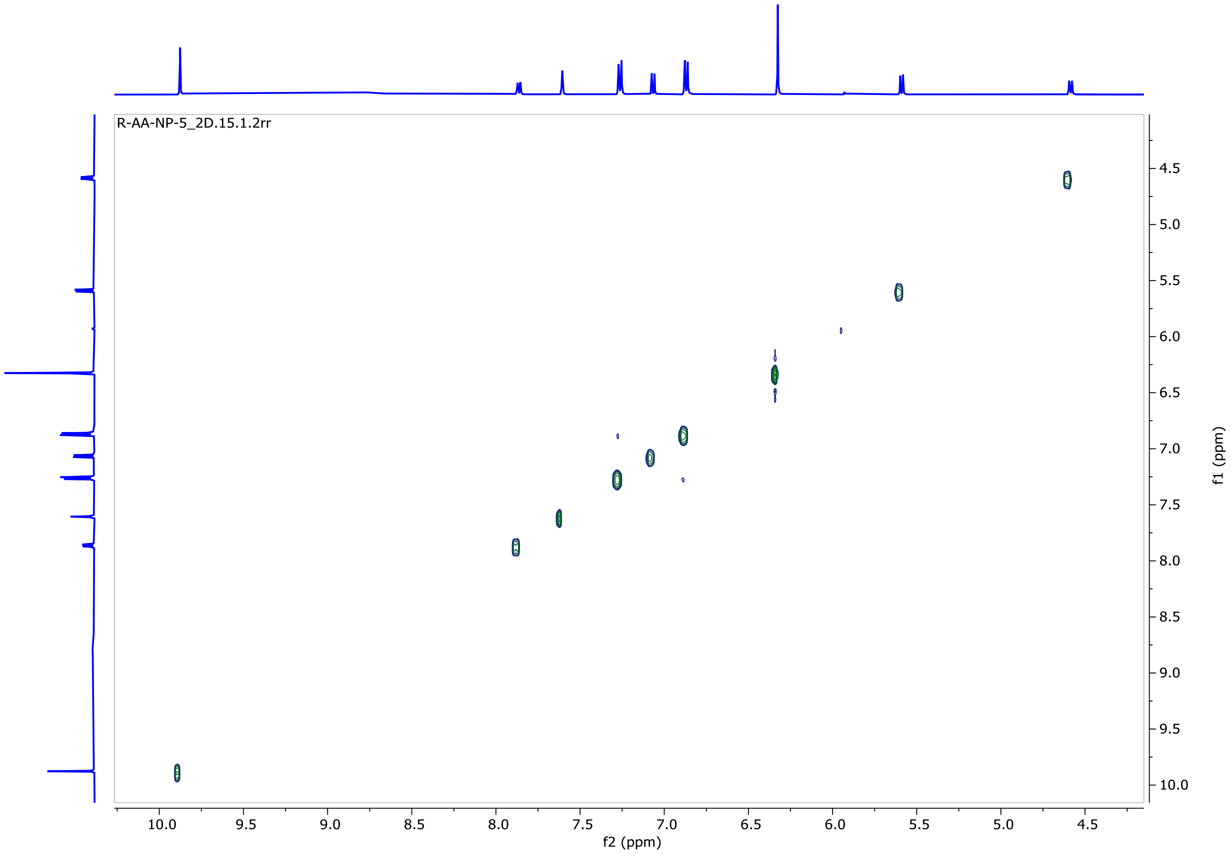

Figure S45. Compound **11**, ^{1}H NMR and ^{13}C, APT NMR spectra.

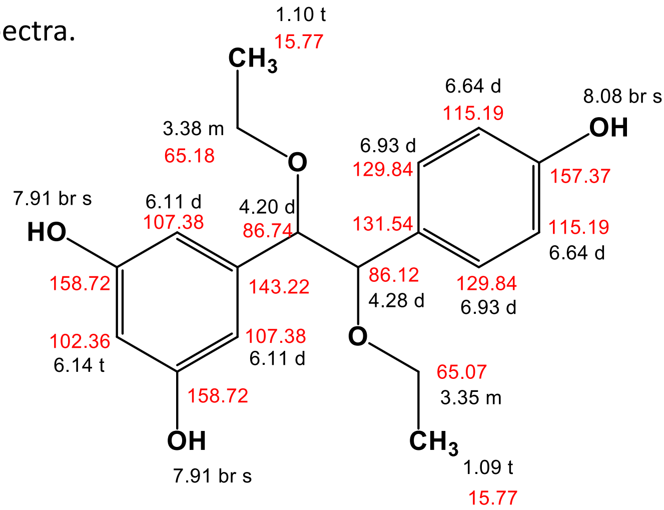

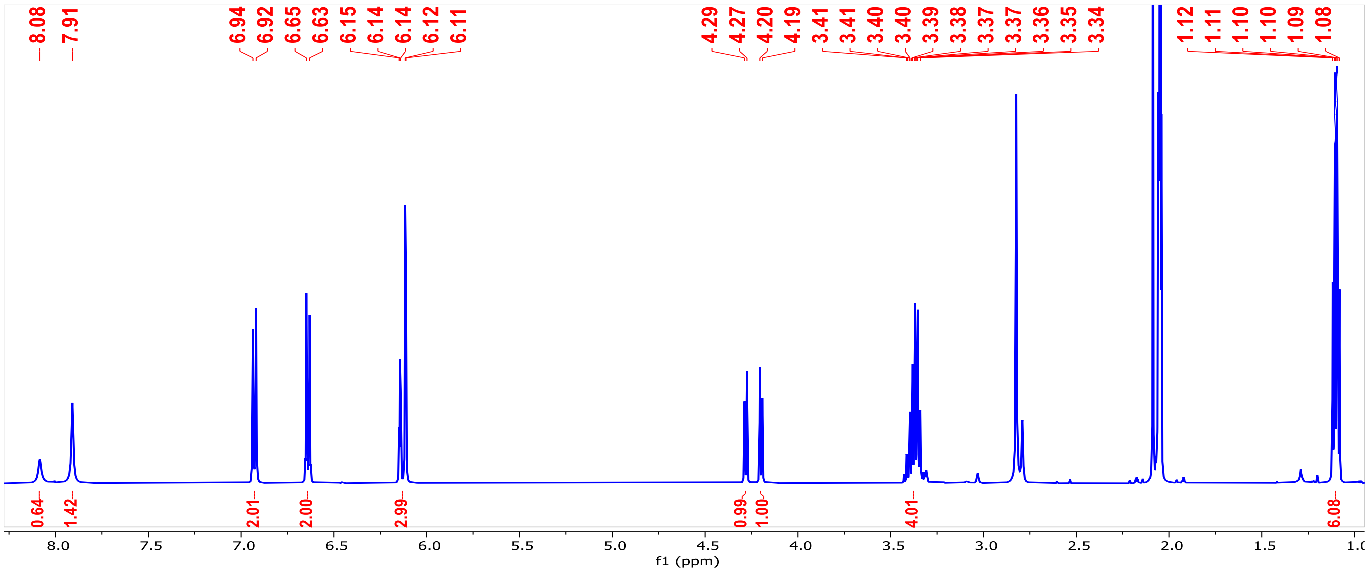

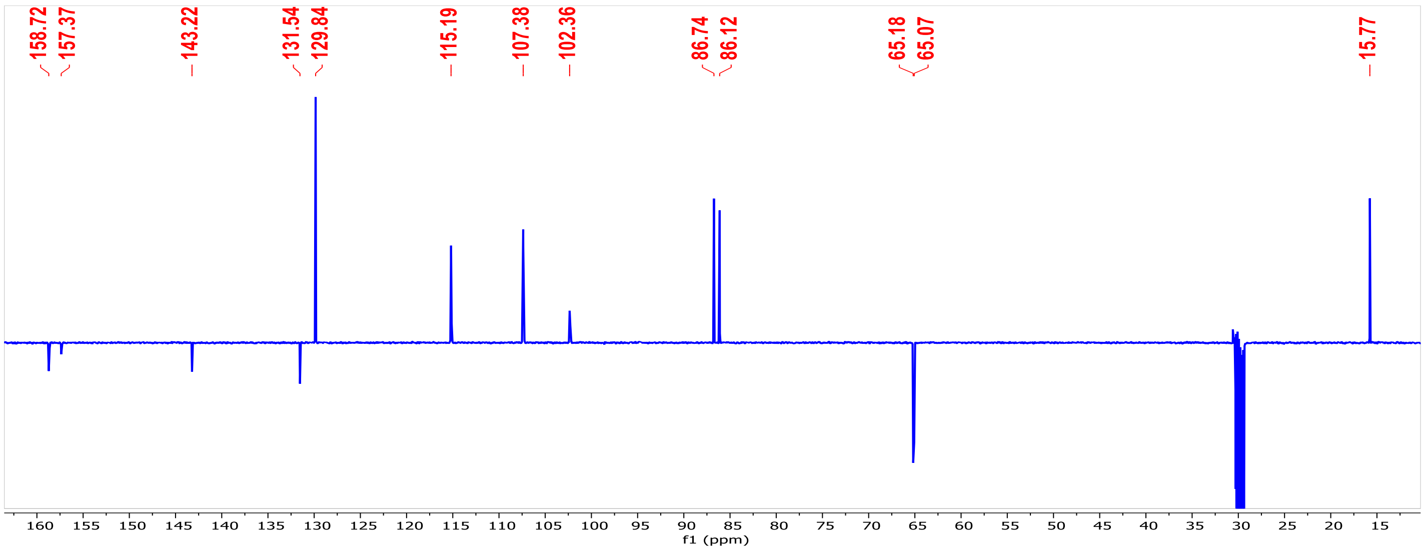

Figure S46. Compound **11**, HSQC and HMBC spectra.

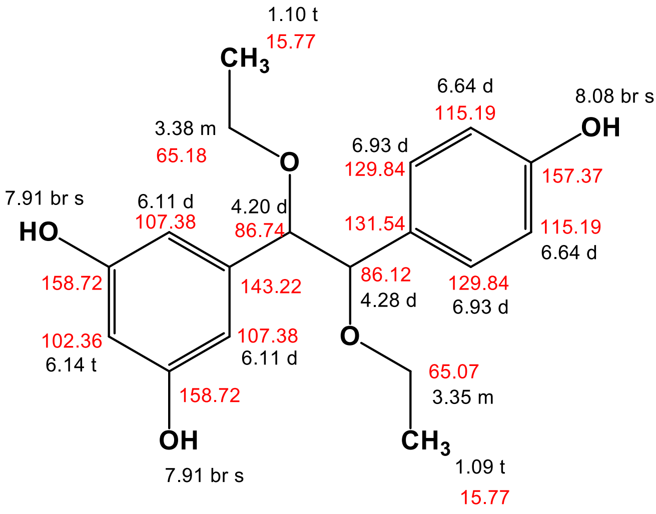

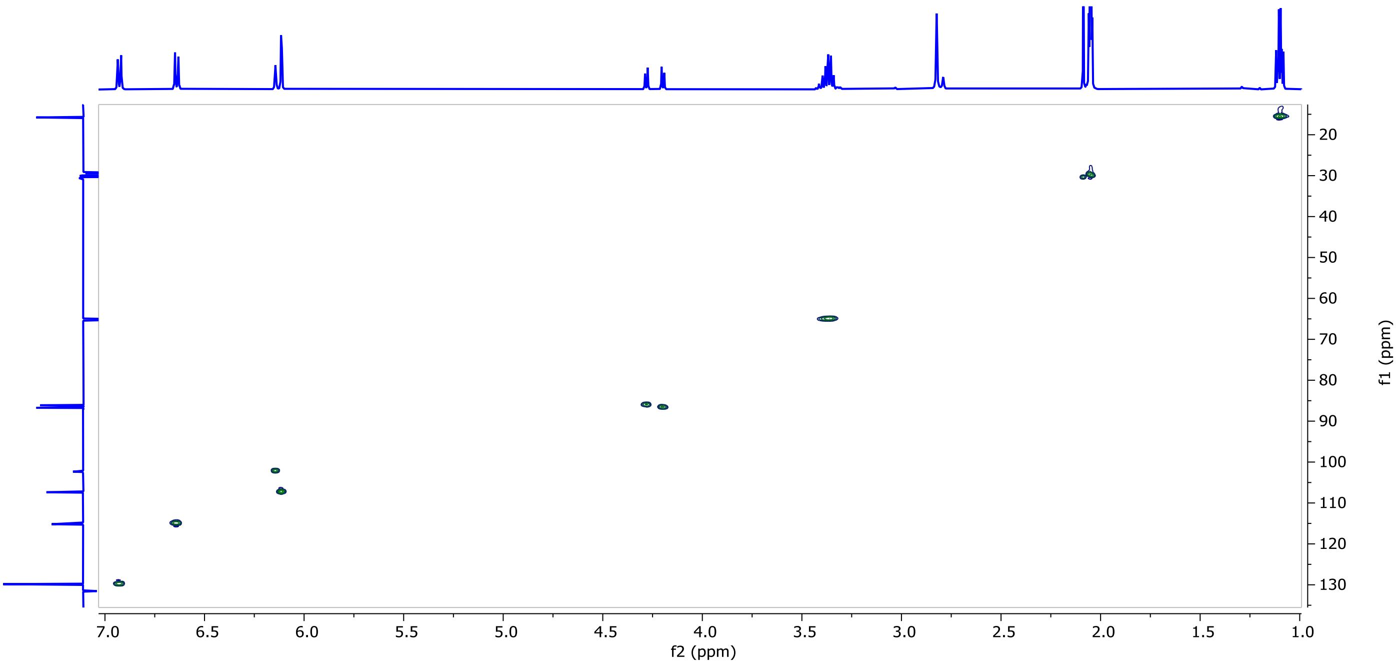

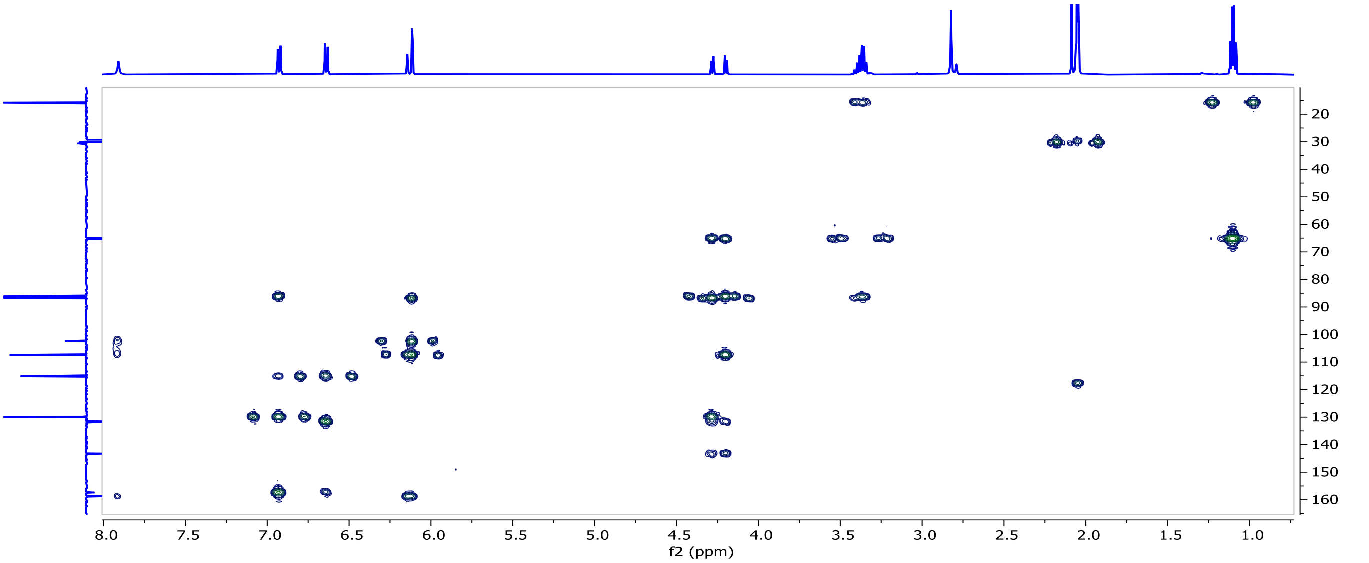

Figure S47. Compound **11**, COSY and NOESY spectra.

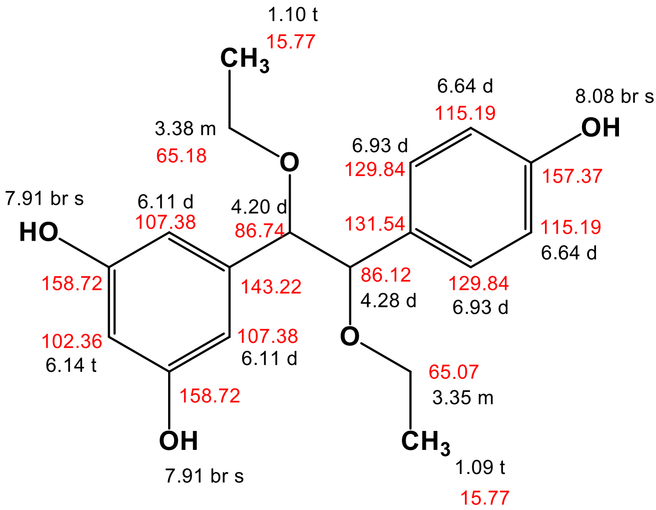

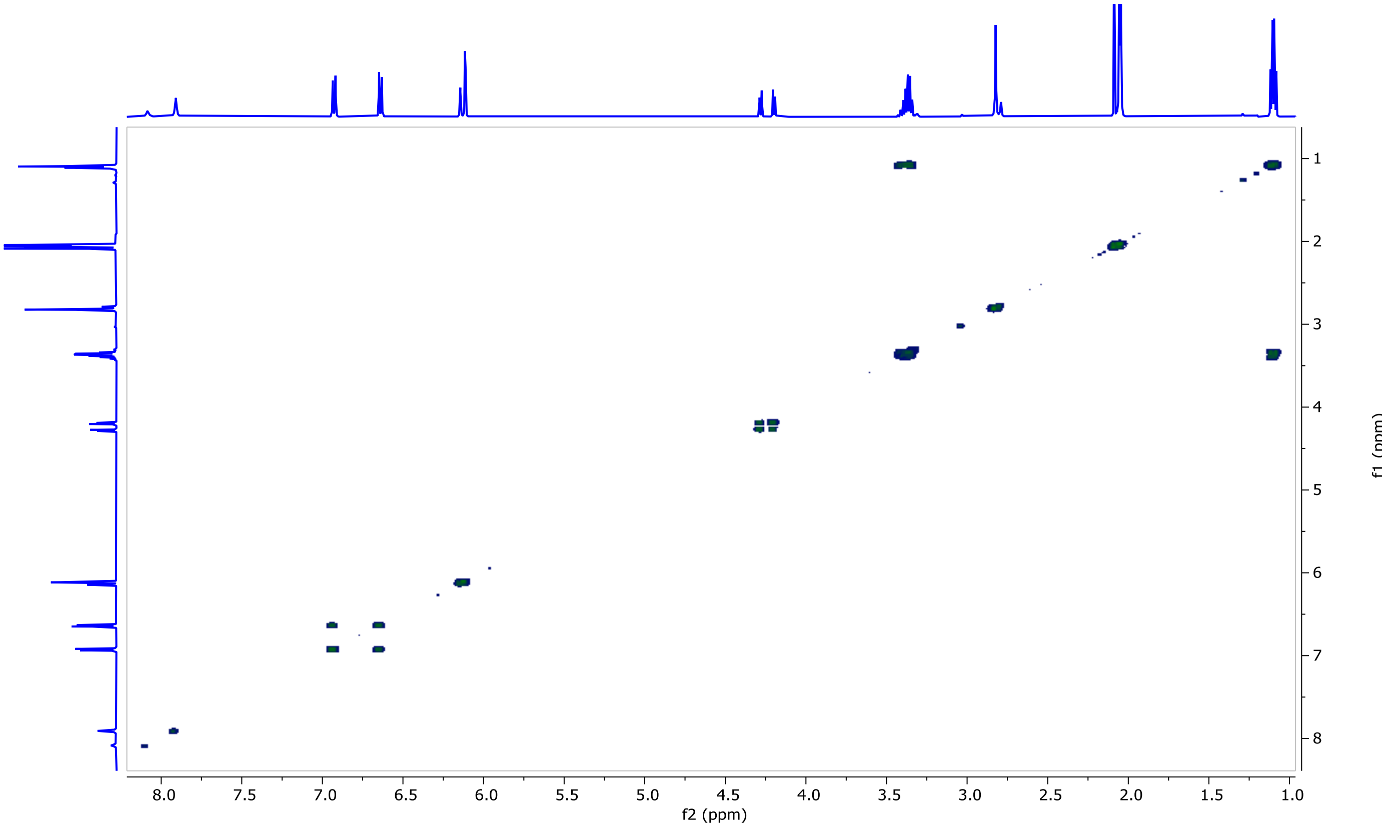

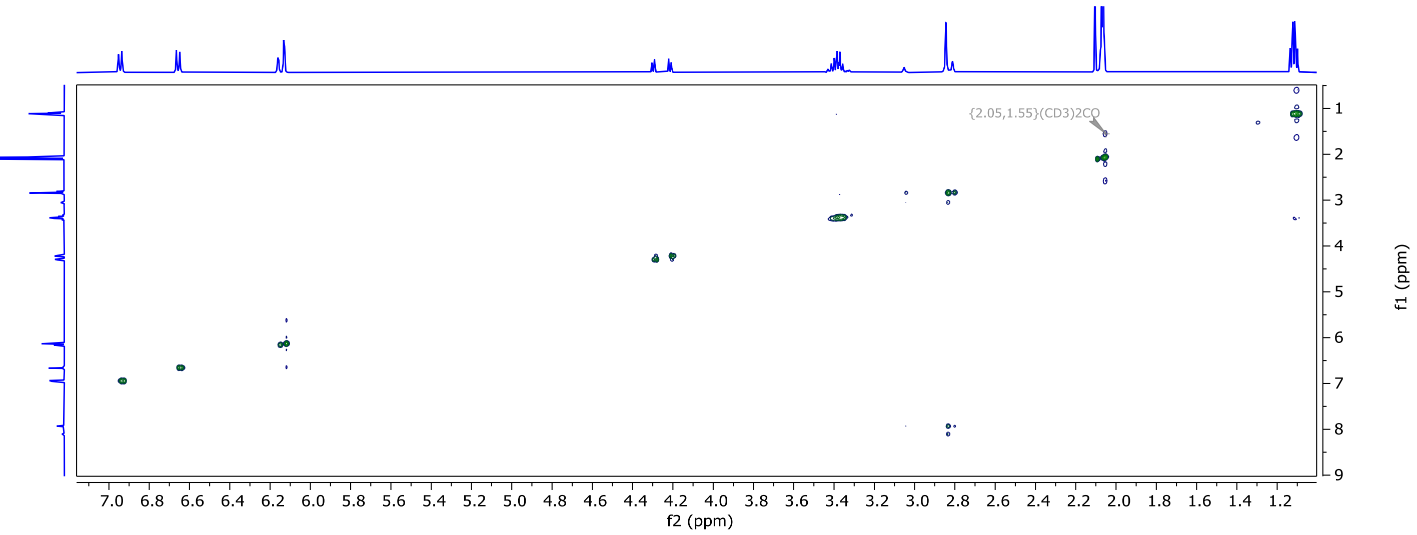

Figure S48. Compound **13**, ^{1}H NMR and ^{13}C, APT NMR spectra.

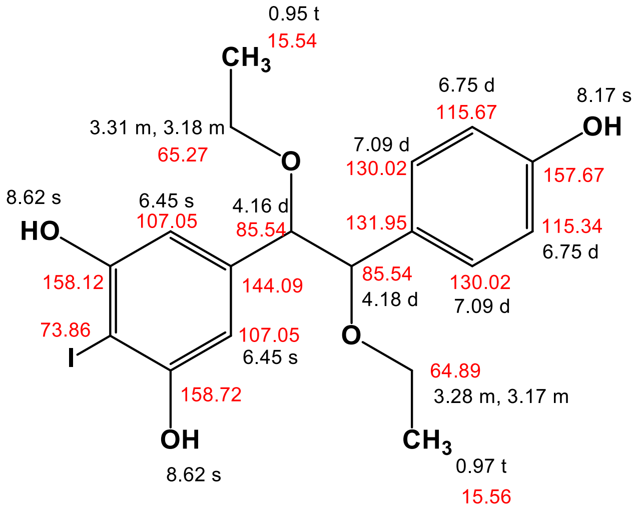

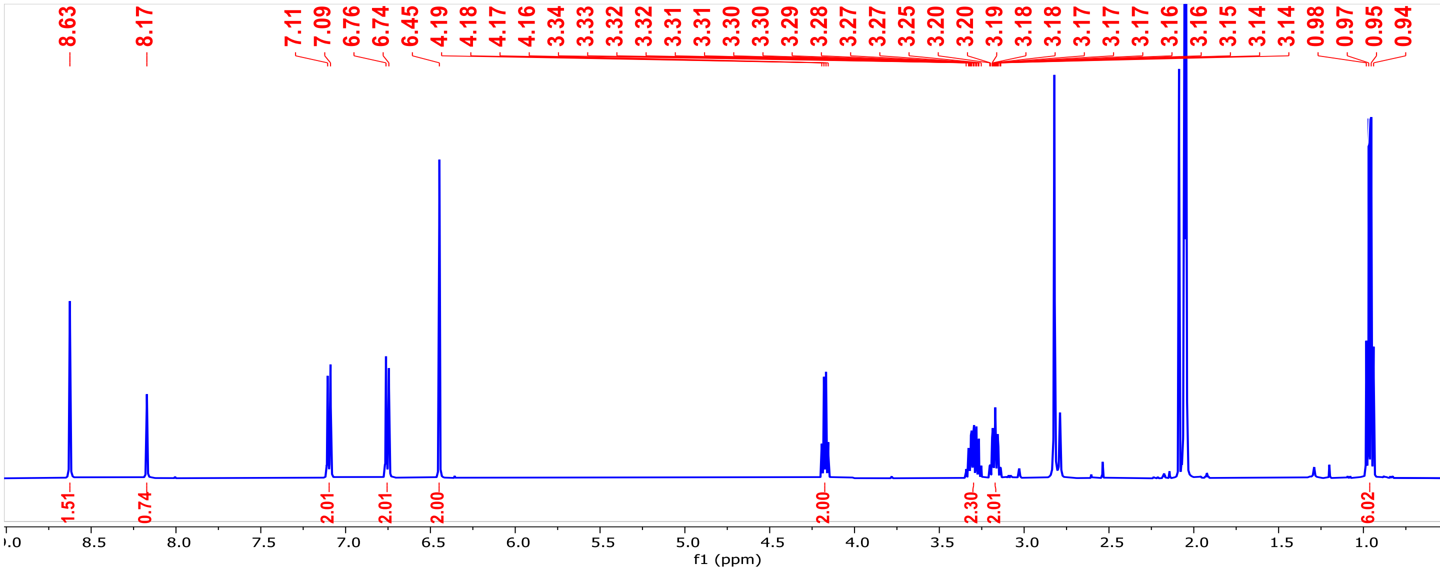

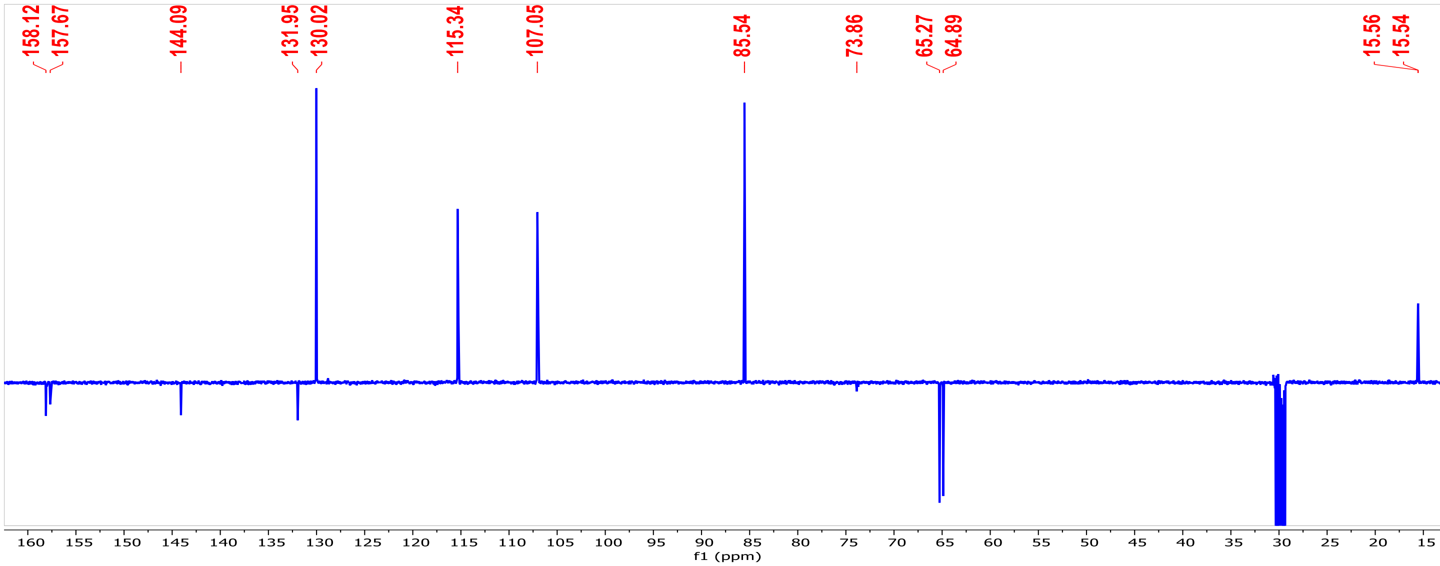

Figure S49. Compound **13**, HSQC, HMBC and COSY spectra.

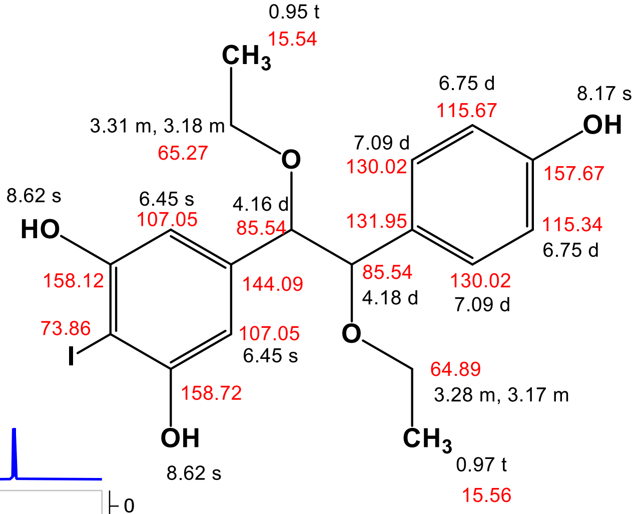

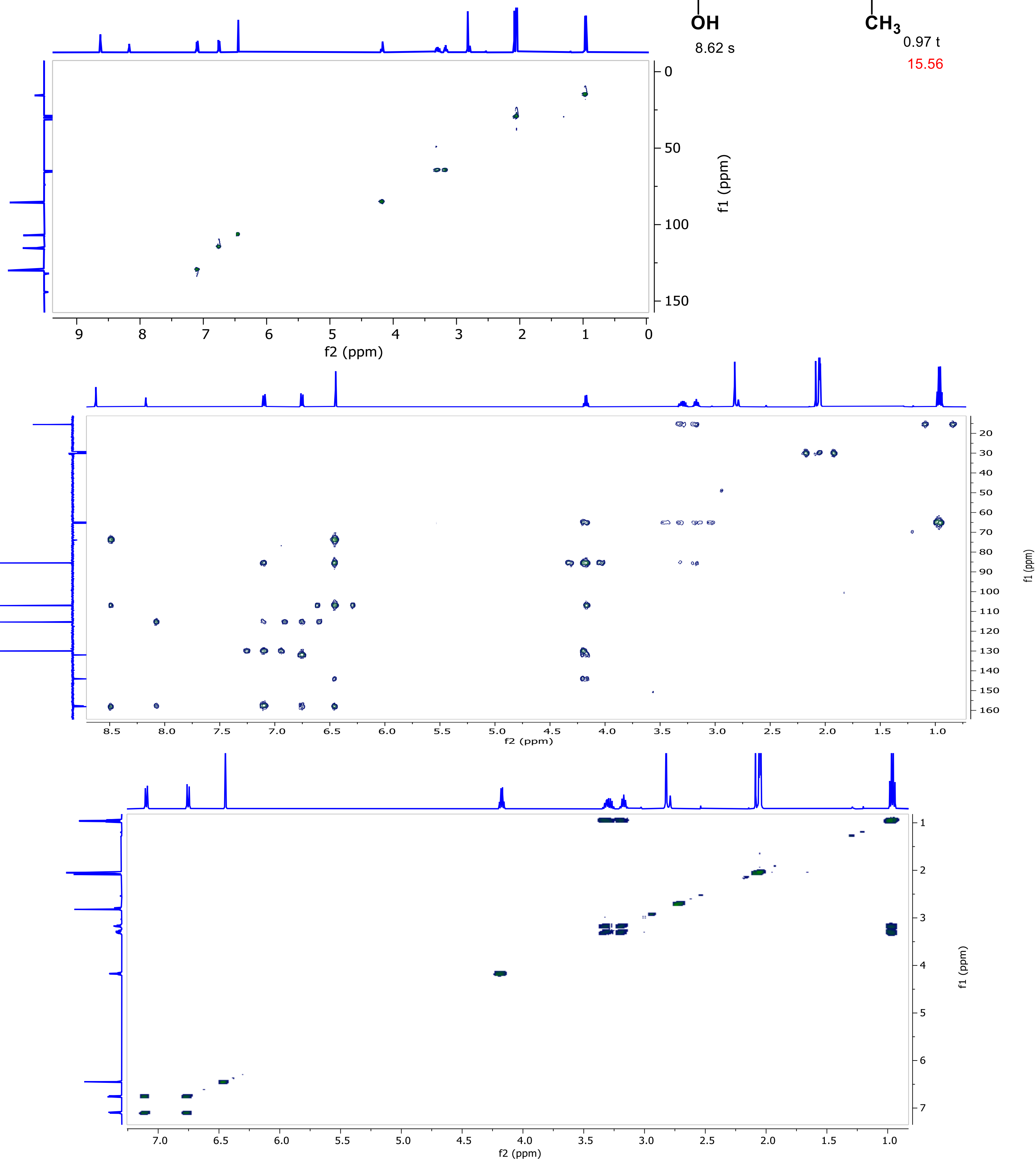

Figure S50. Compound **14**, ^{1}H NMR and ^{13}C, APT NMR spectra.

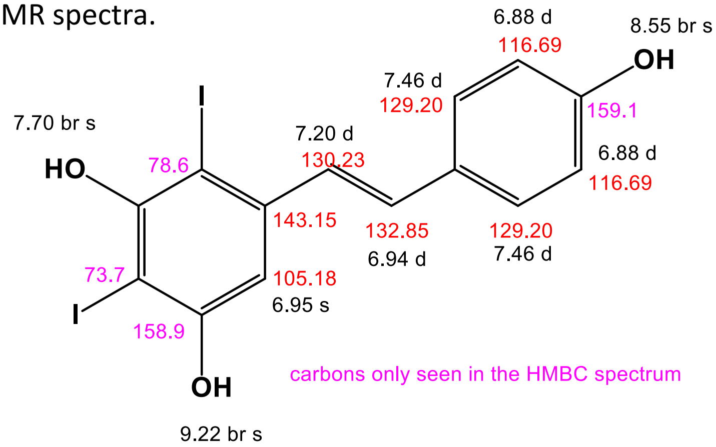

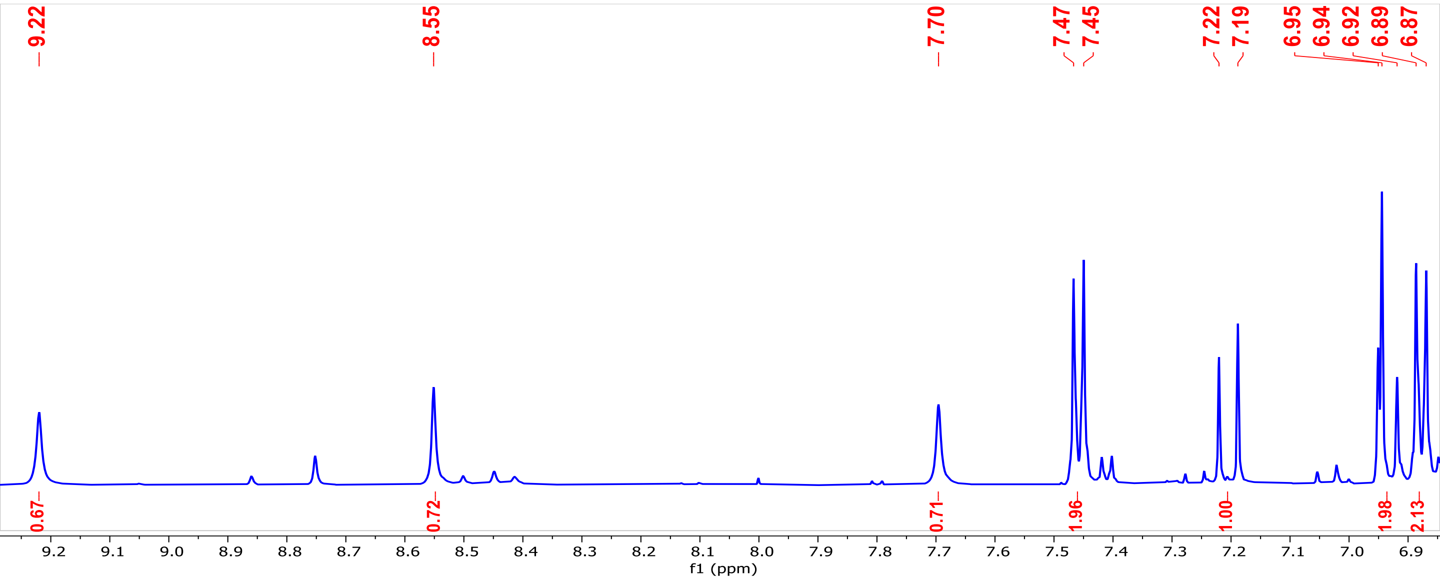

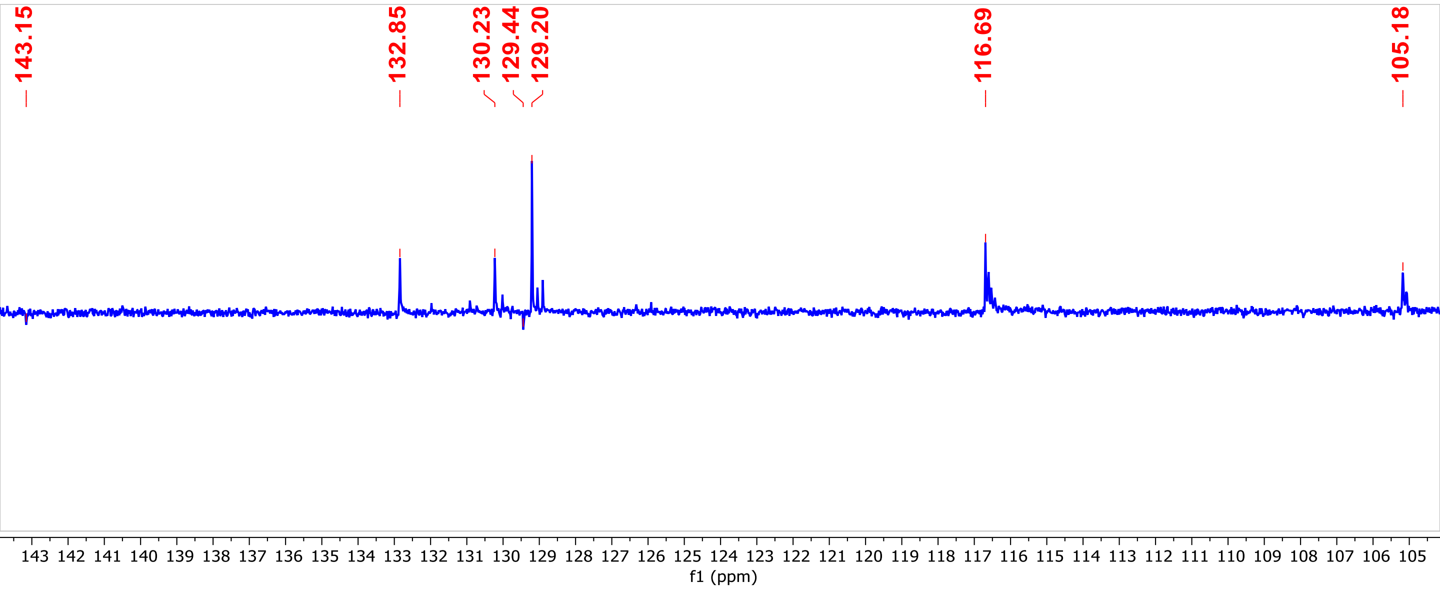

Figure S51. Compound **14**, HSQC and HMBC spectra.

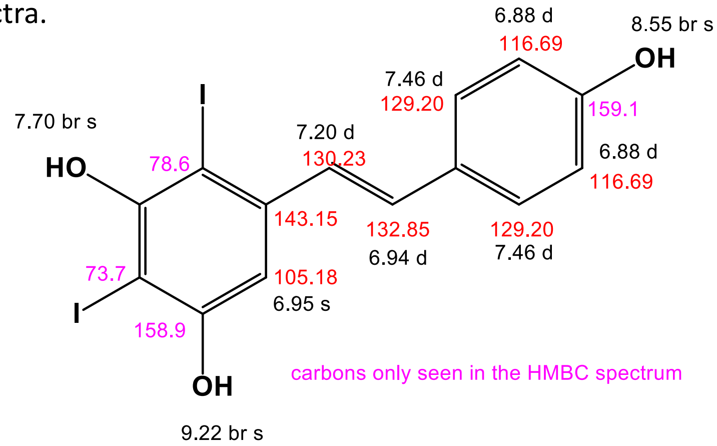

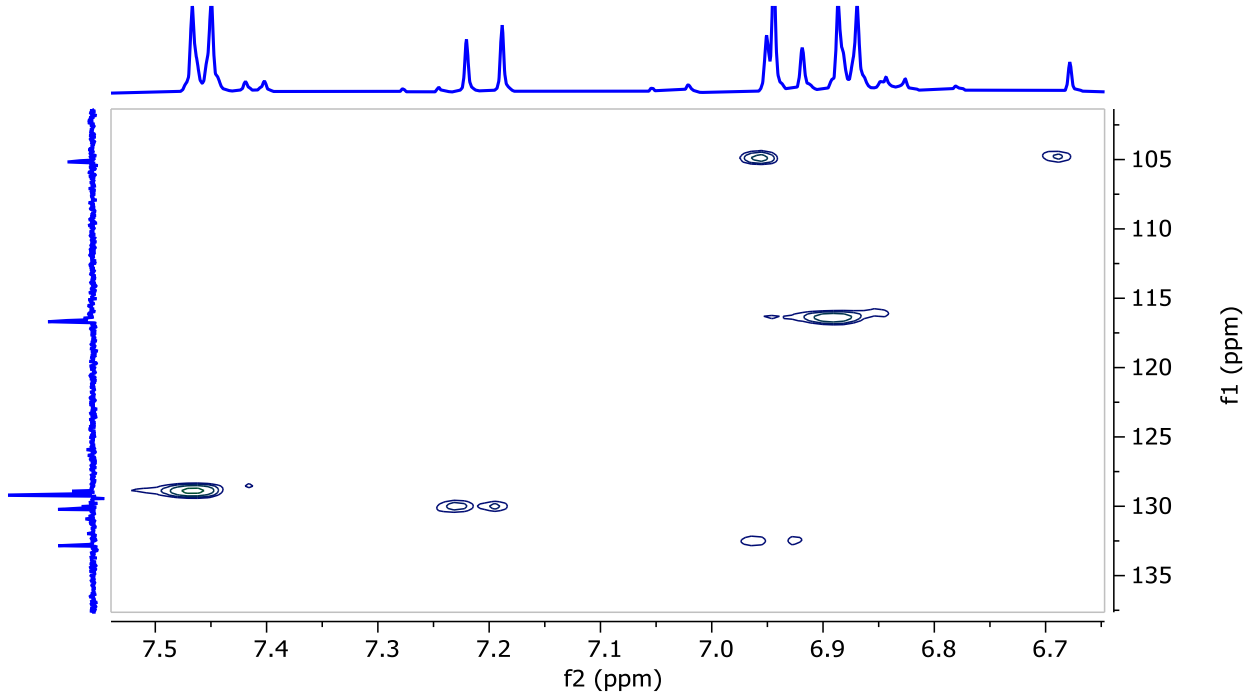

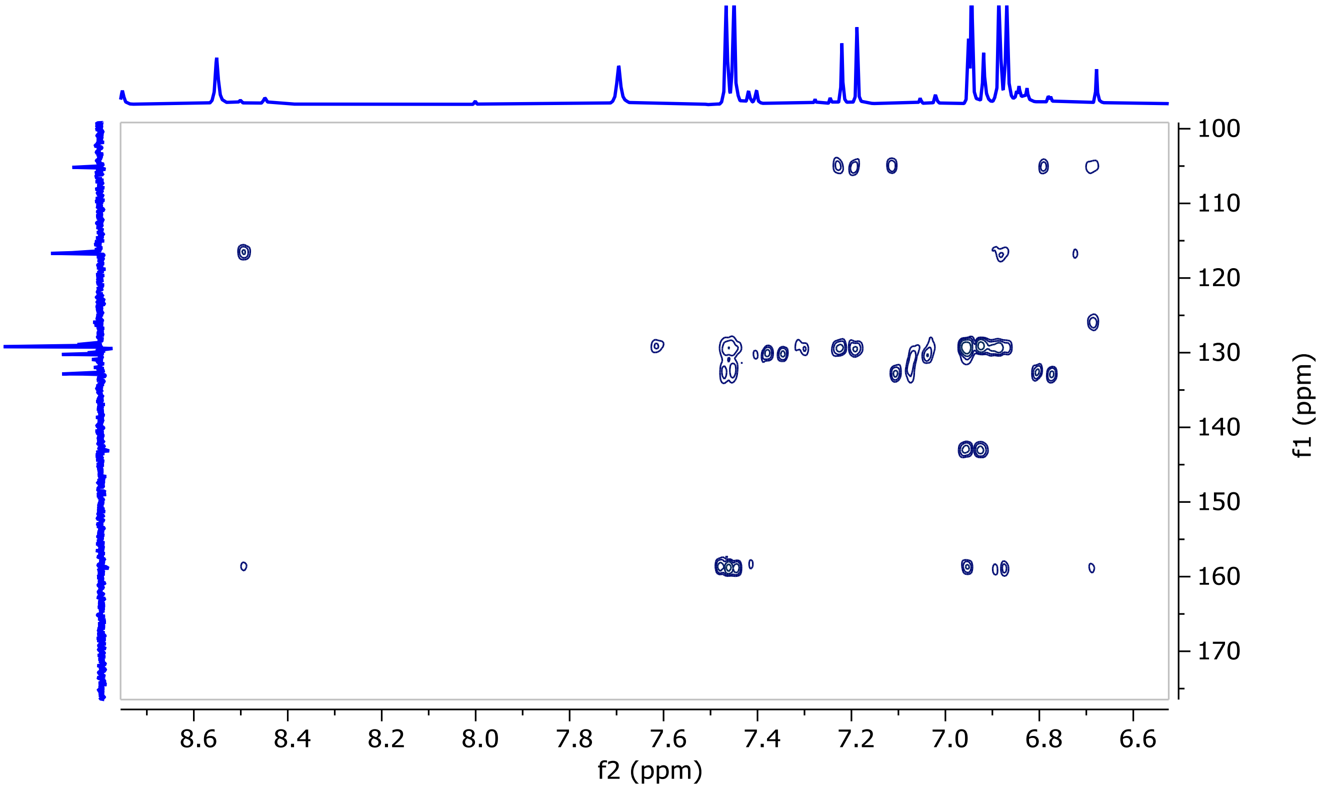

Figure S52. Compound **14**, COSY and NOESY spectra.

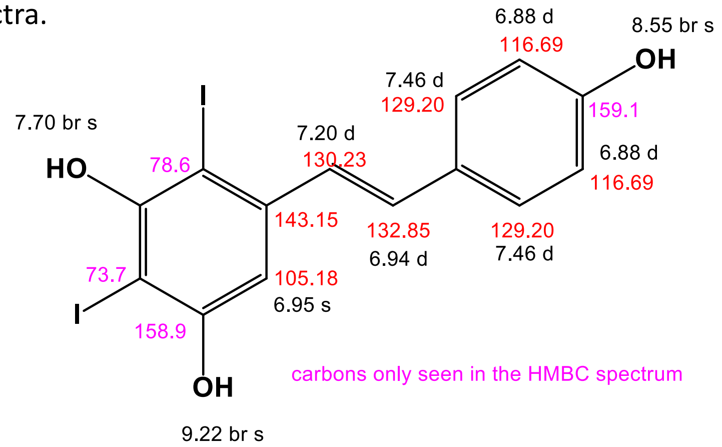

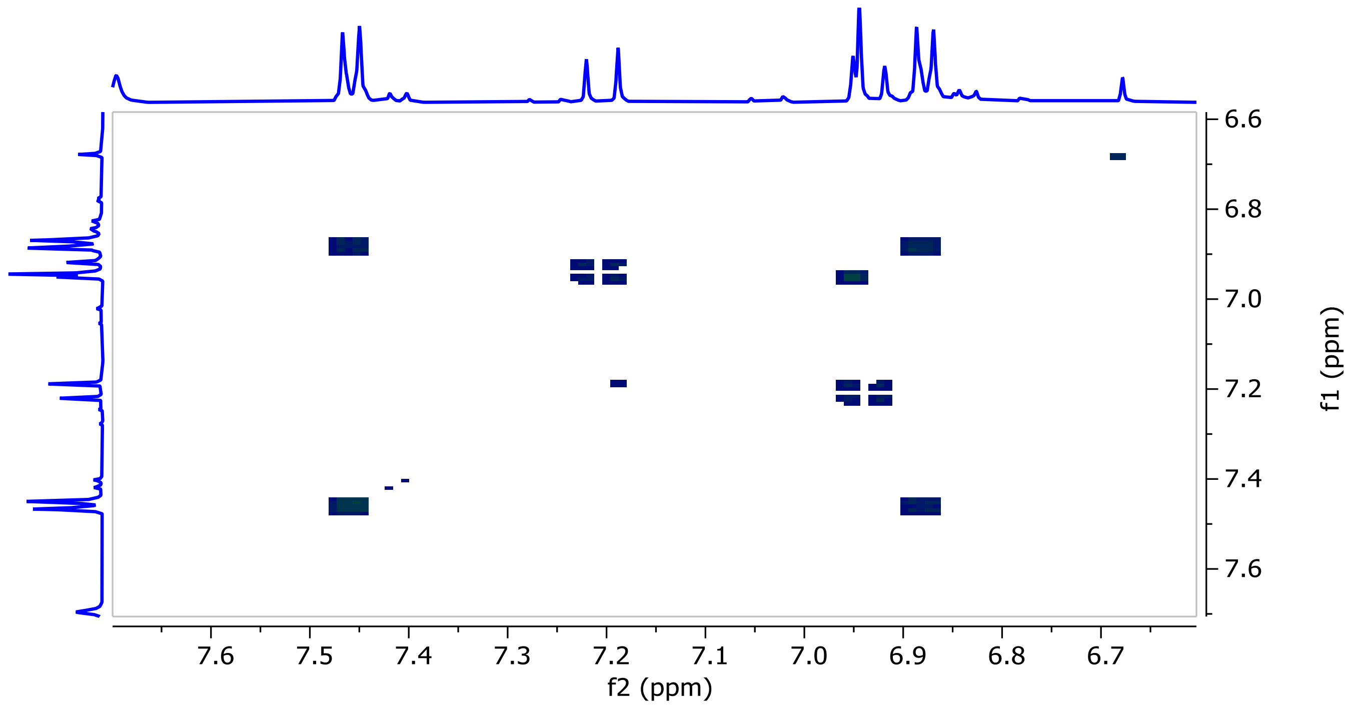

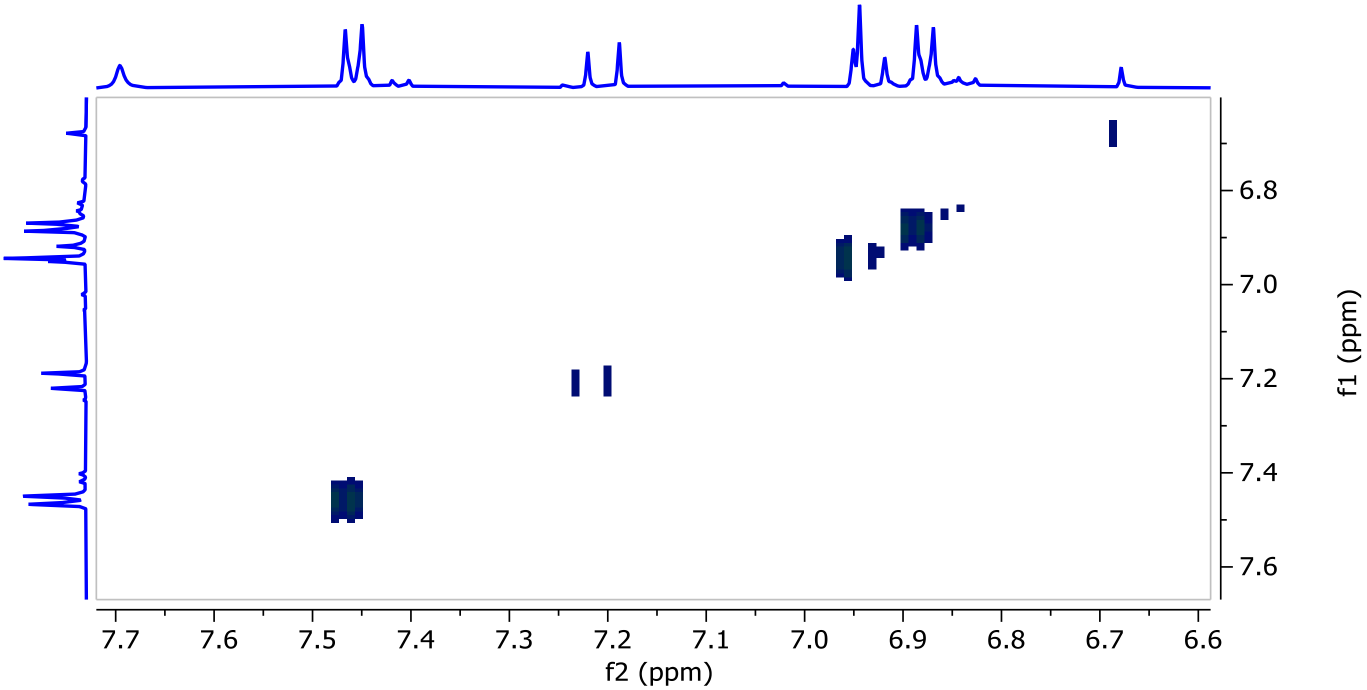

Figure S53. Compound **15**, ^{1}H NMR and ^{13}C, APT NMR spectra.

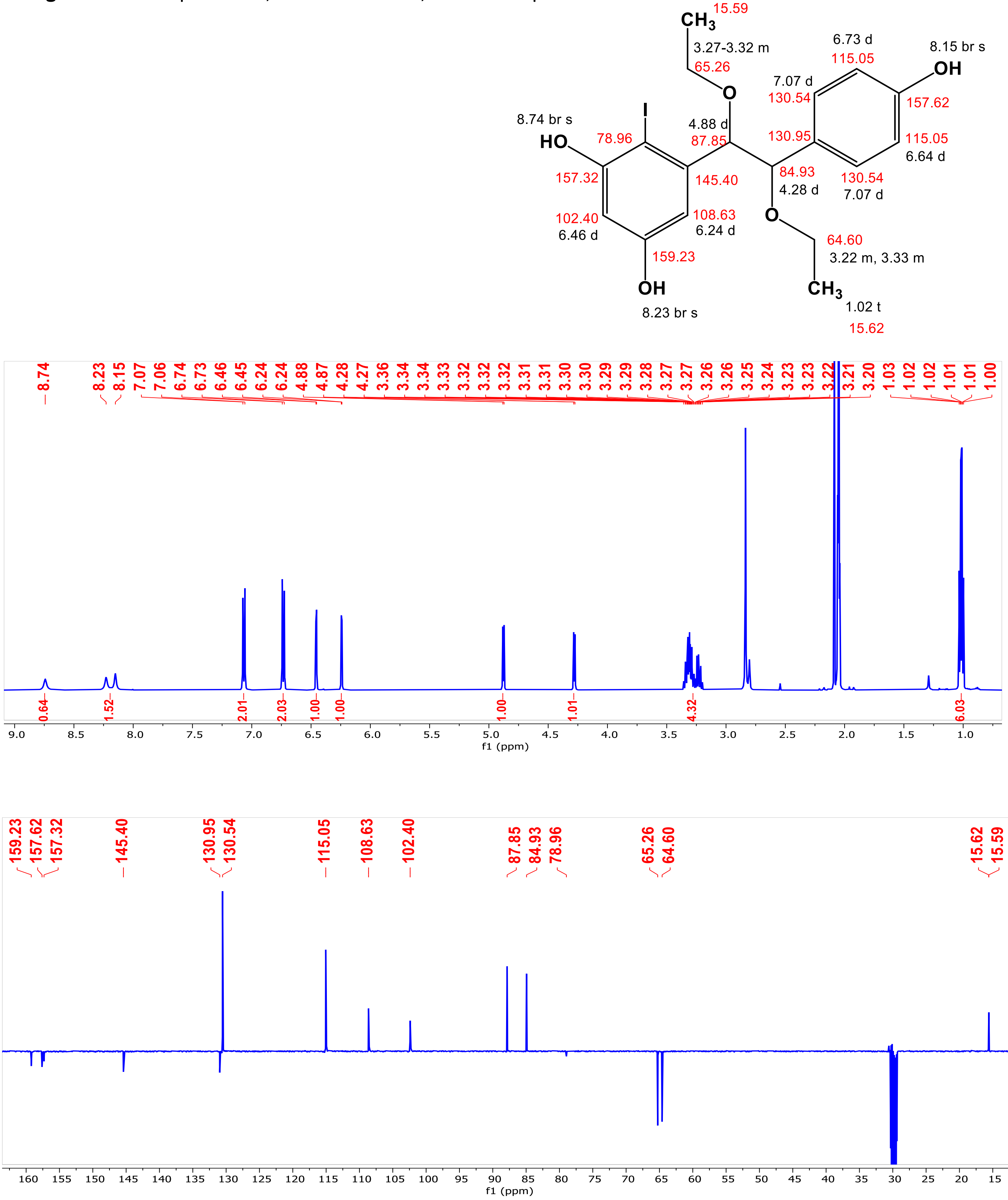

Figure S54. Compound **15**, HSQC and HMBC spectra.

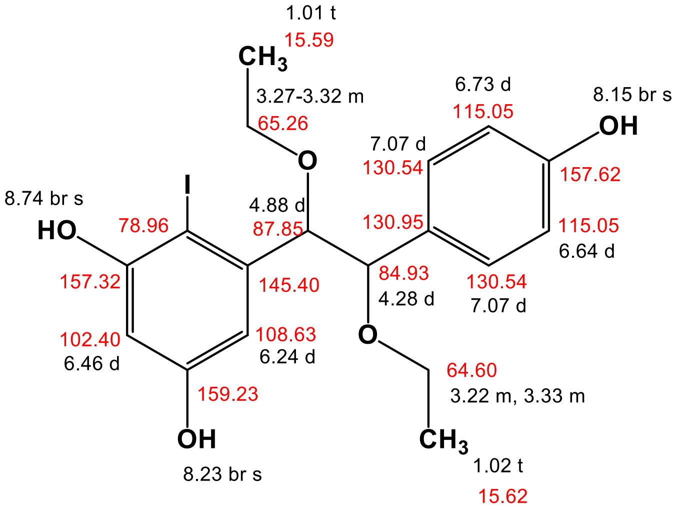

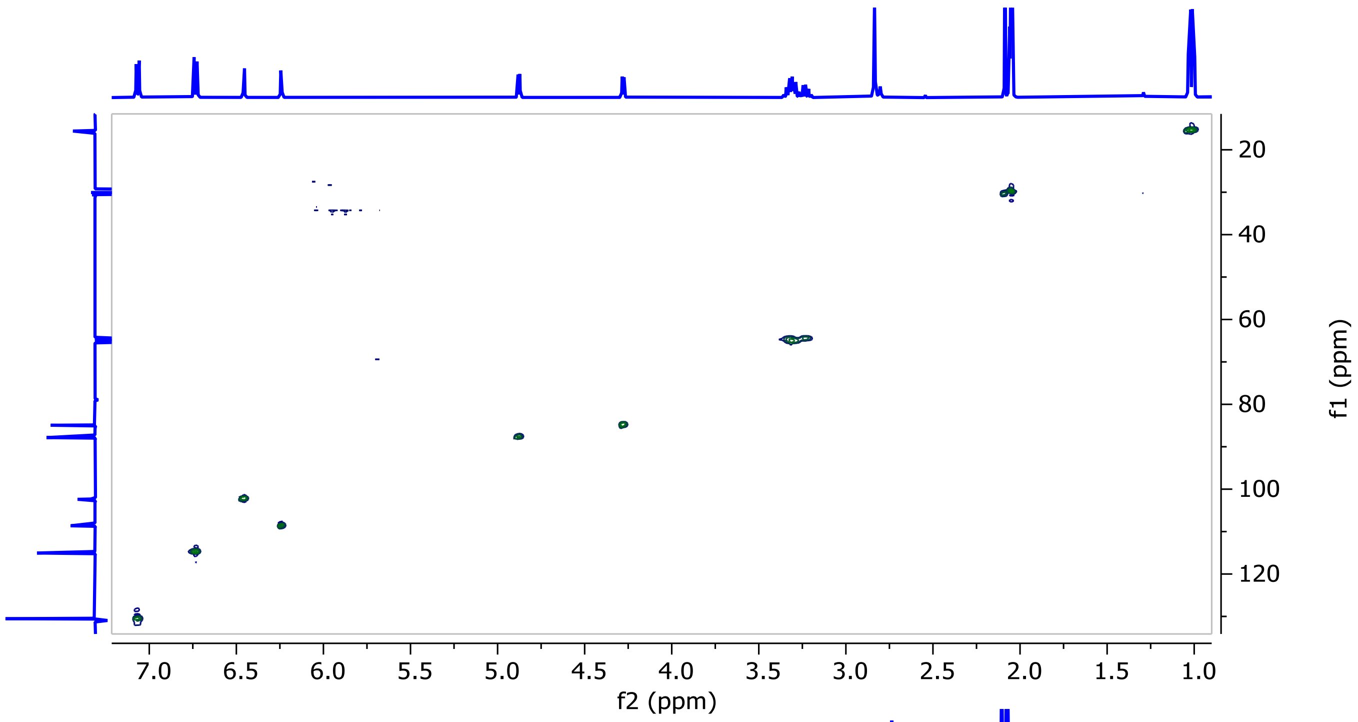

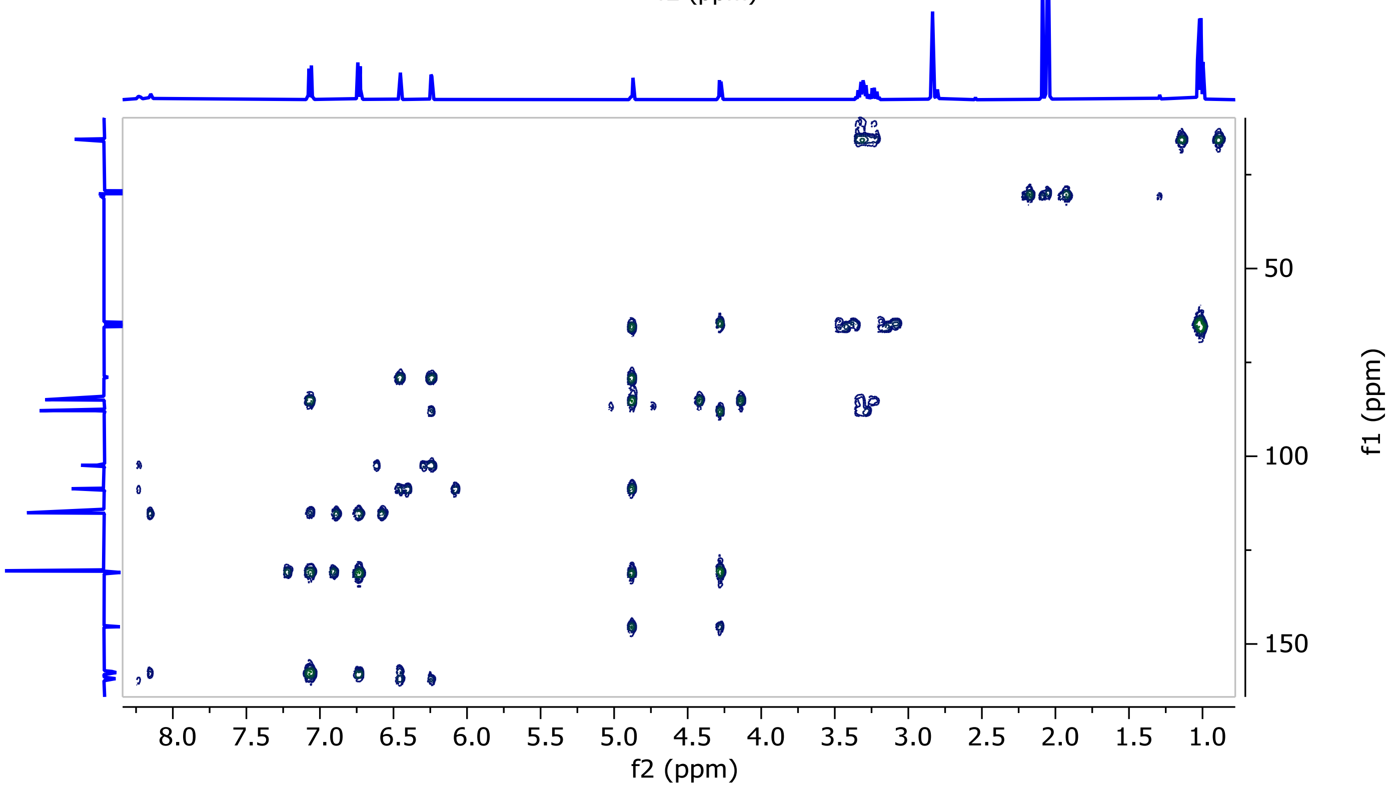

Figure S55. Compound **15**, COSY and NOESY spectra.

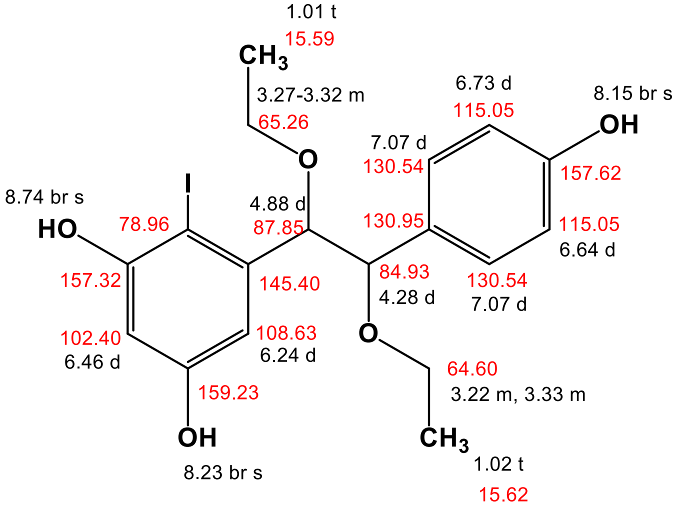

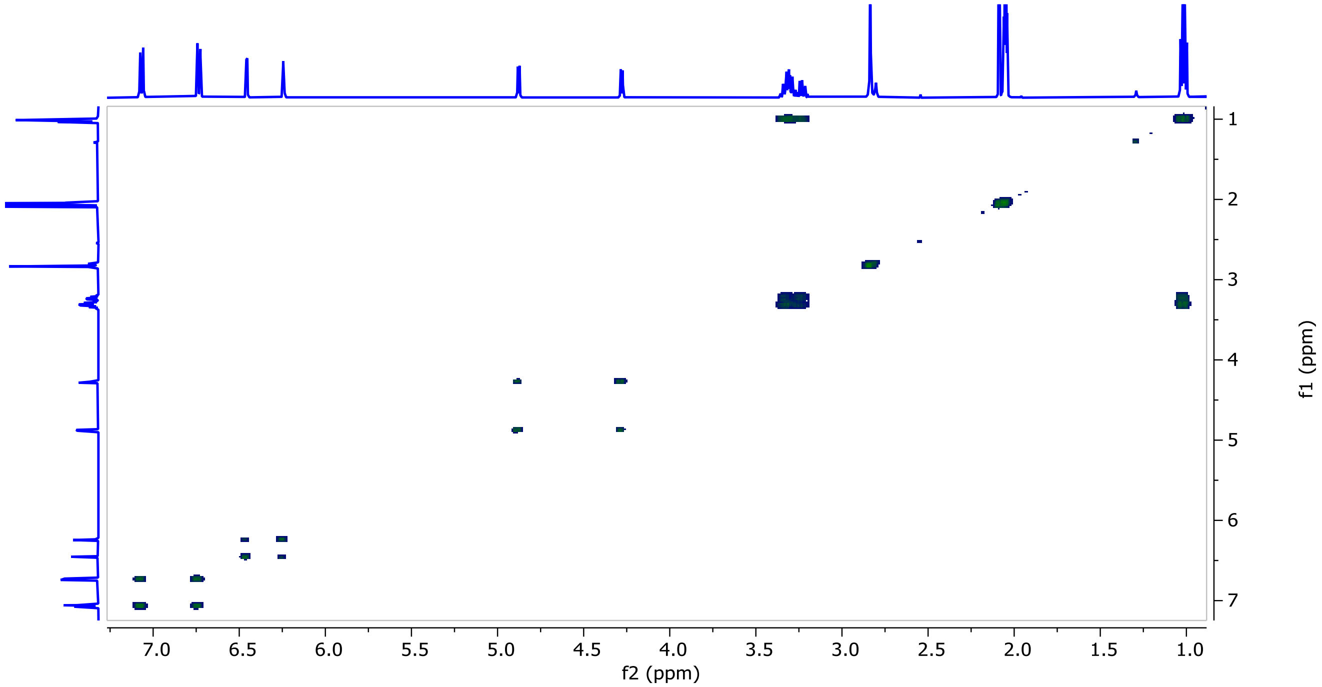

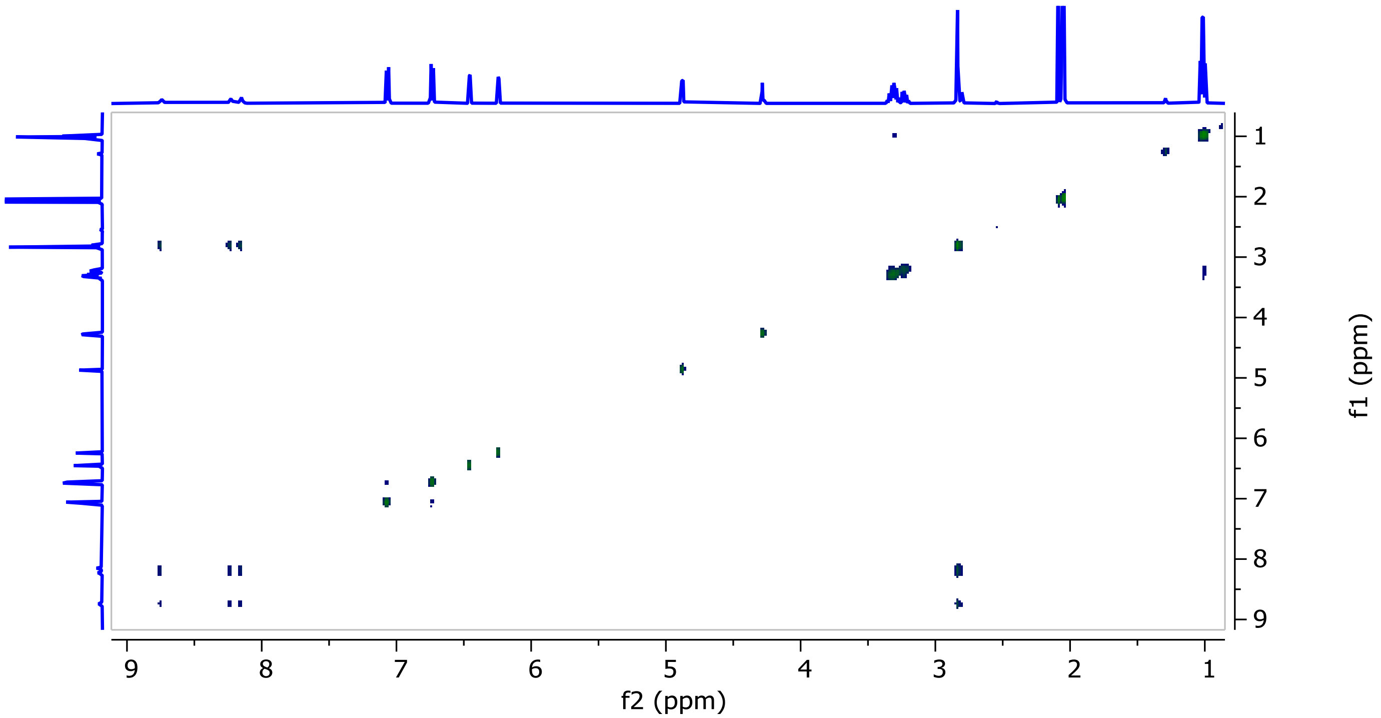

Figure S56. Compound **17**, ^{1}H NMR and ^{13}C, APT NMR spectra.

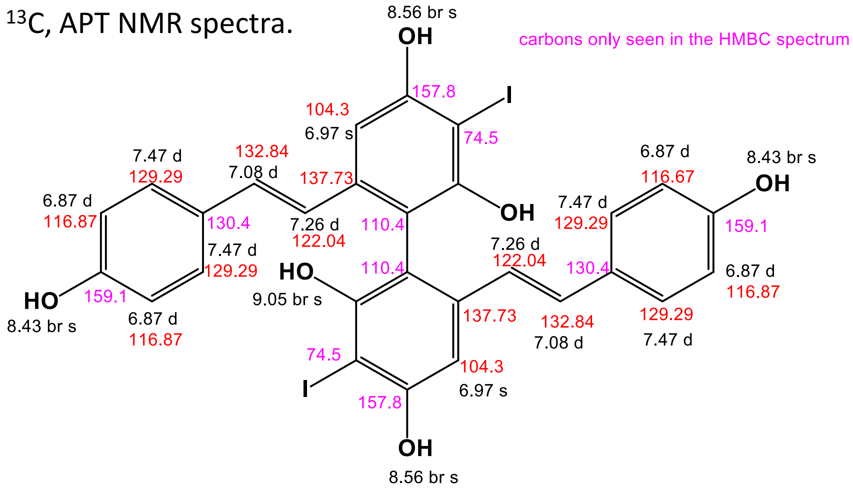

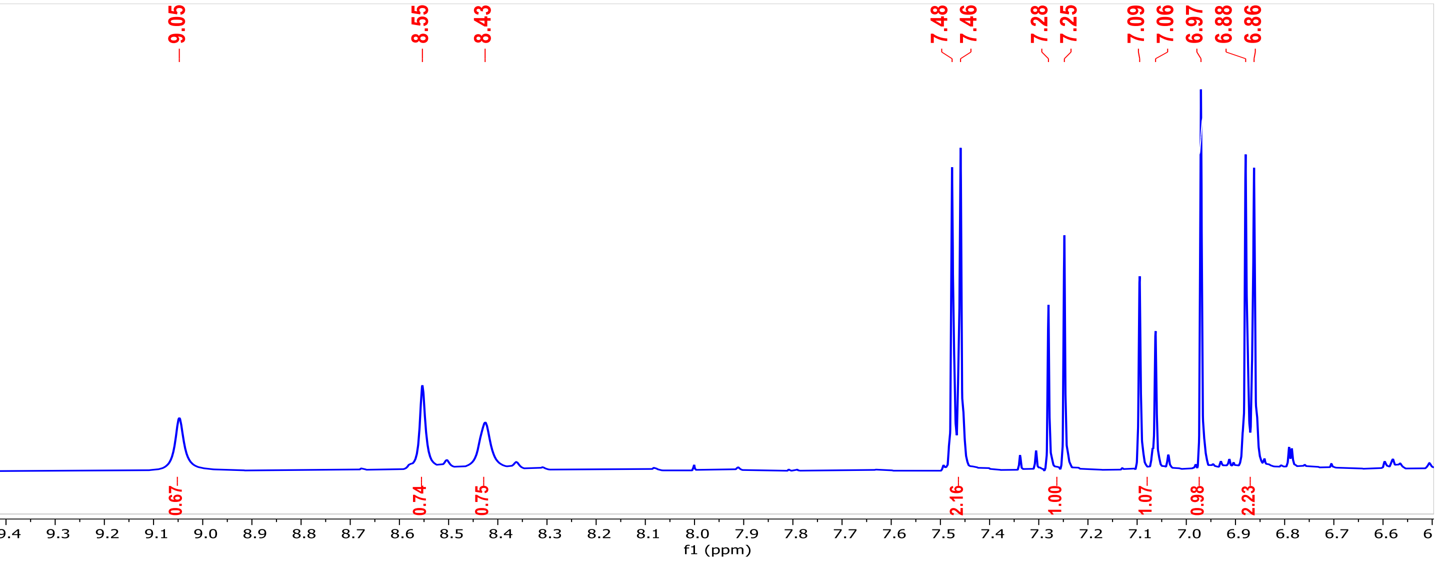

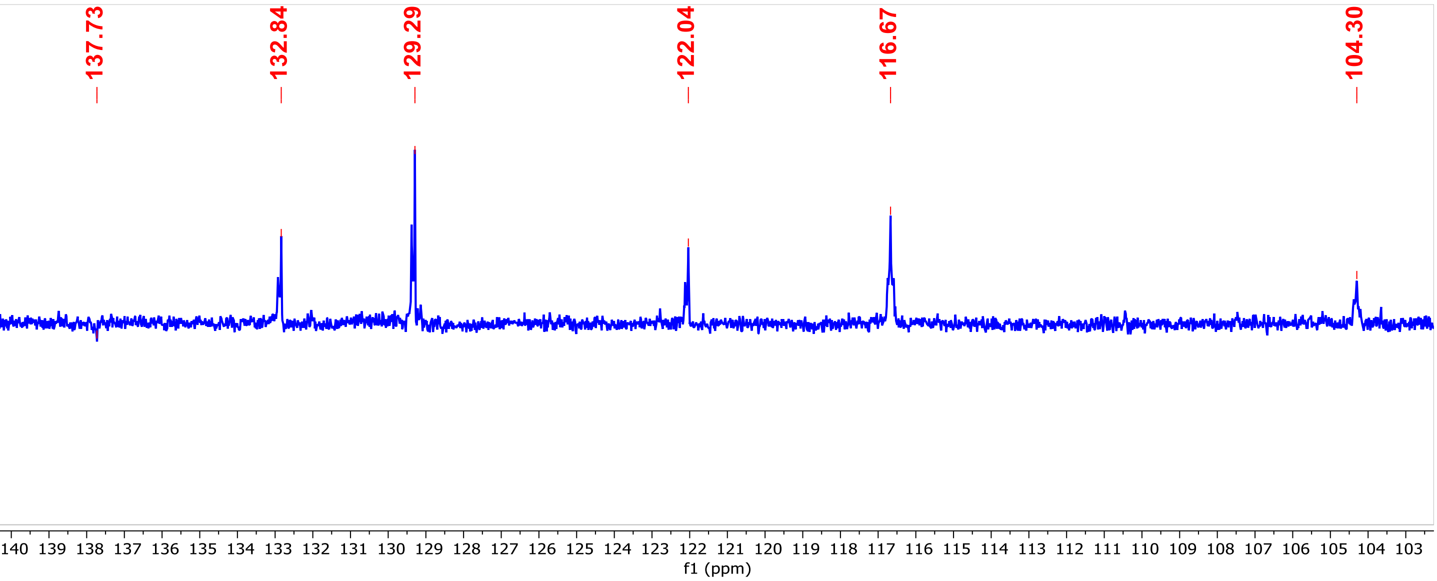

Figure S57. Compound **17**, HSQC, HMBC and COSY spectra.

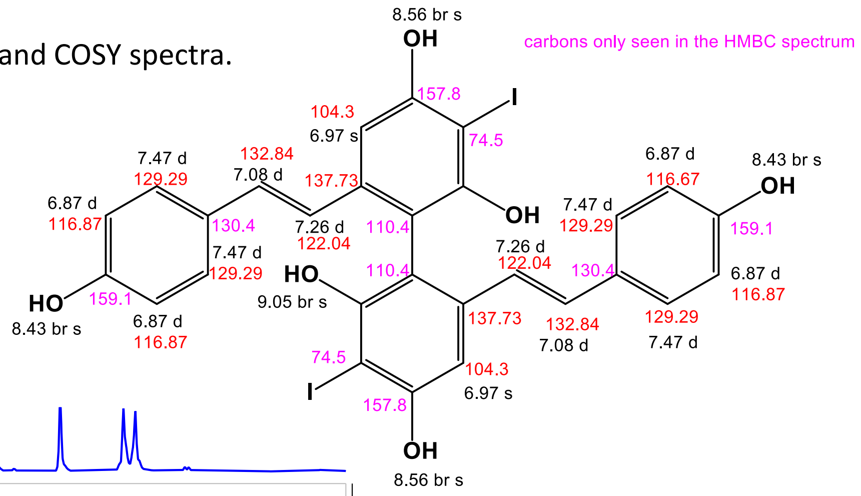

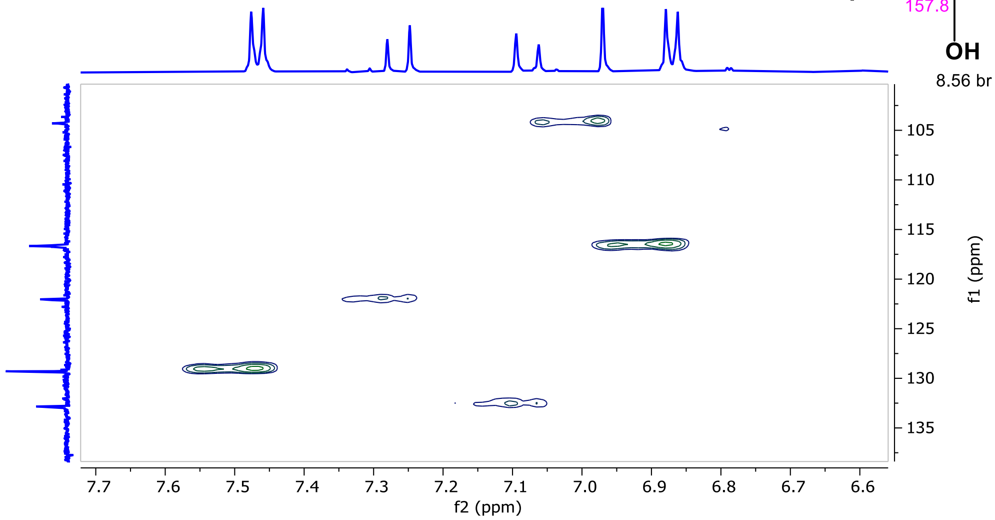

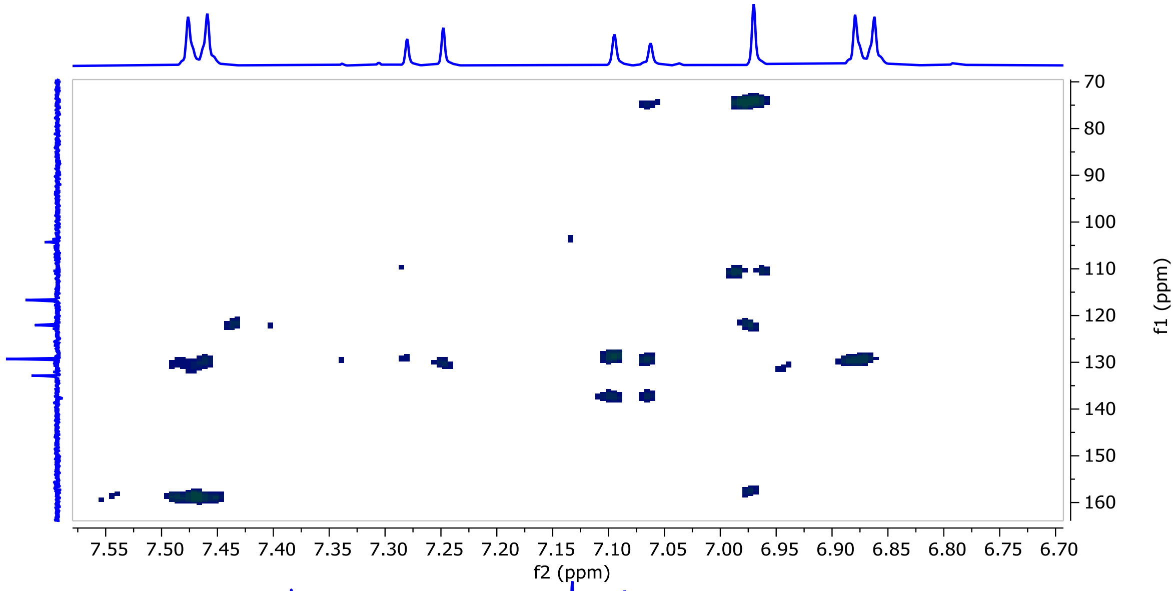

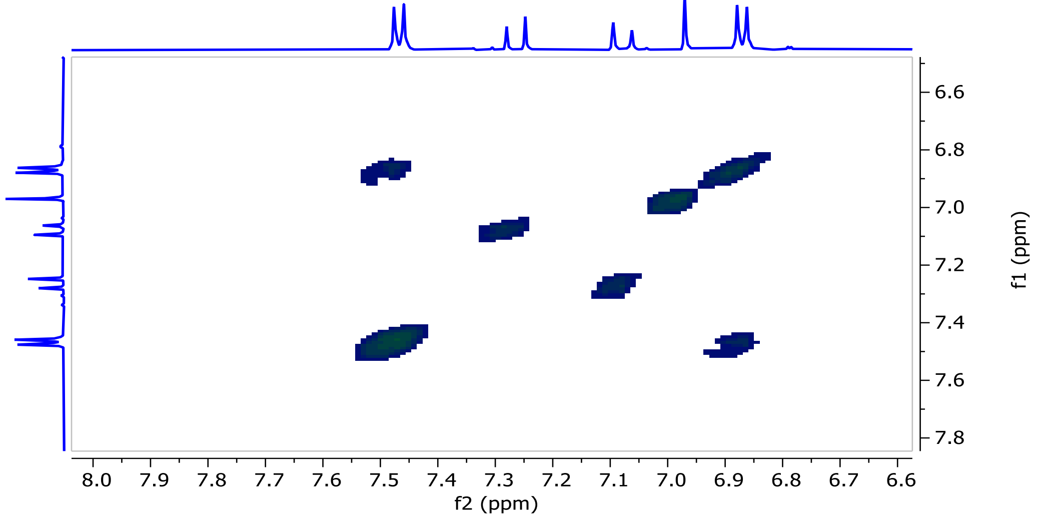

Figure S58. Compound **18**, ^{1}H NMR and ^{13}C, APT NMR spectra.

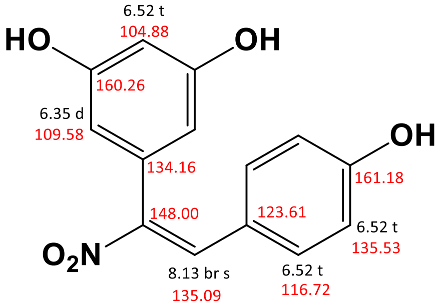

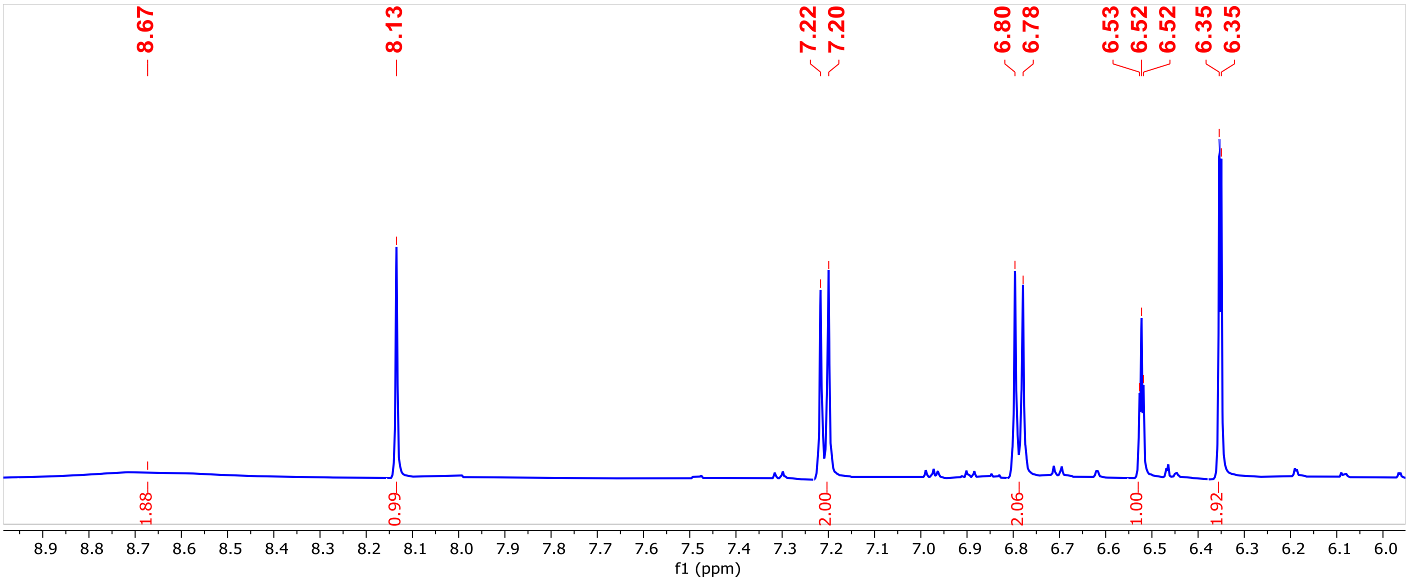

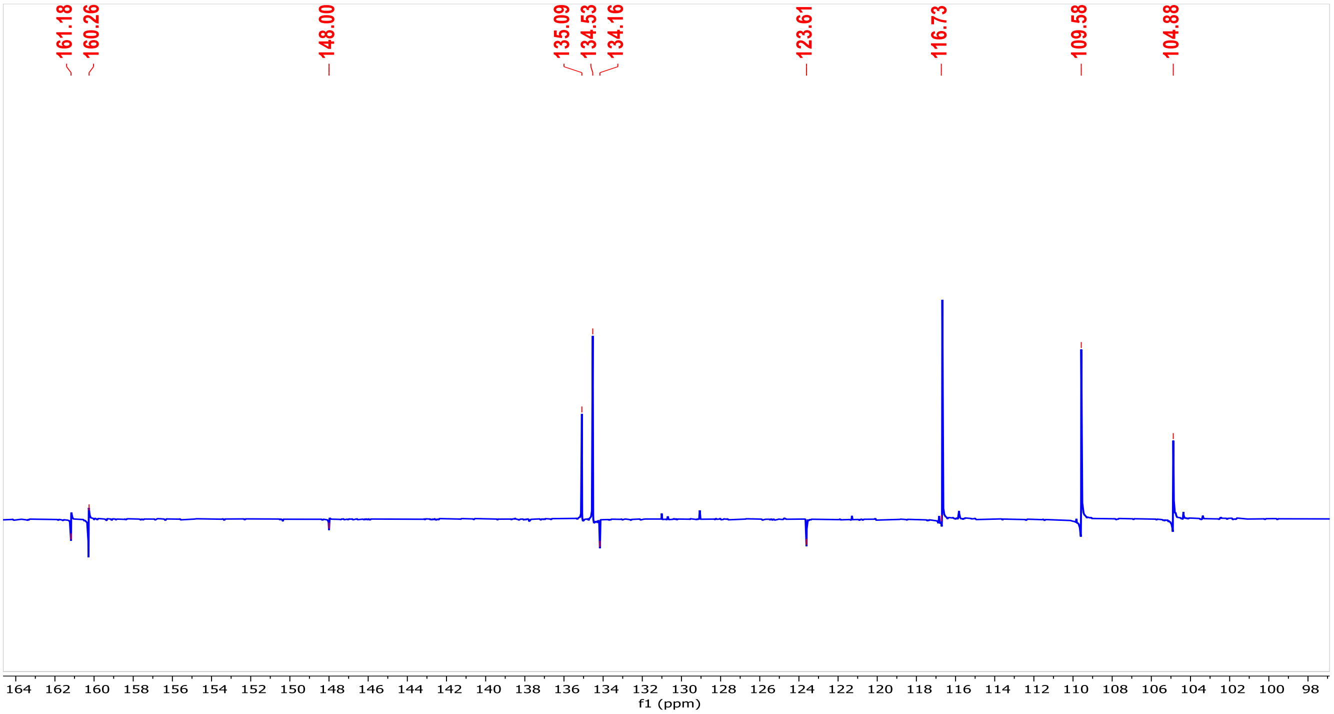

Figure S59. Compound **18**, HSQC and HMBC spectra.

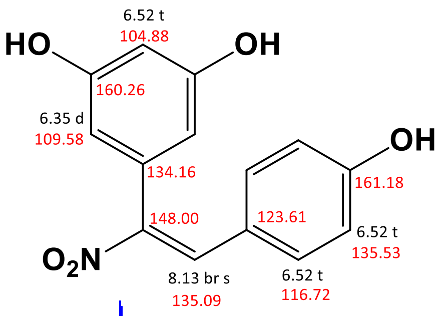

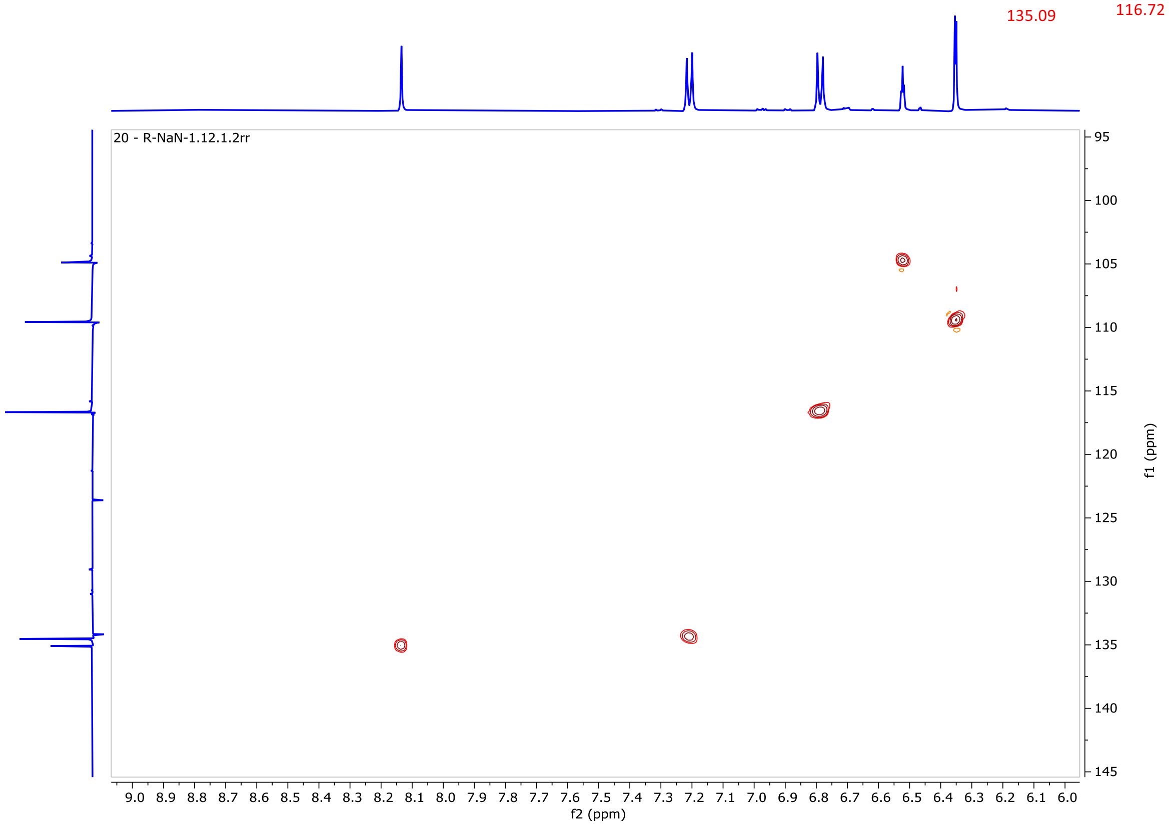

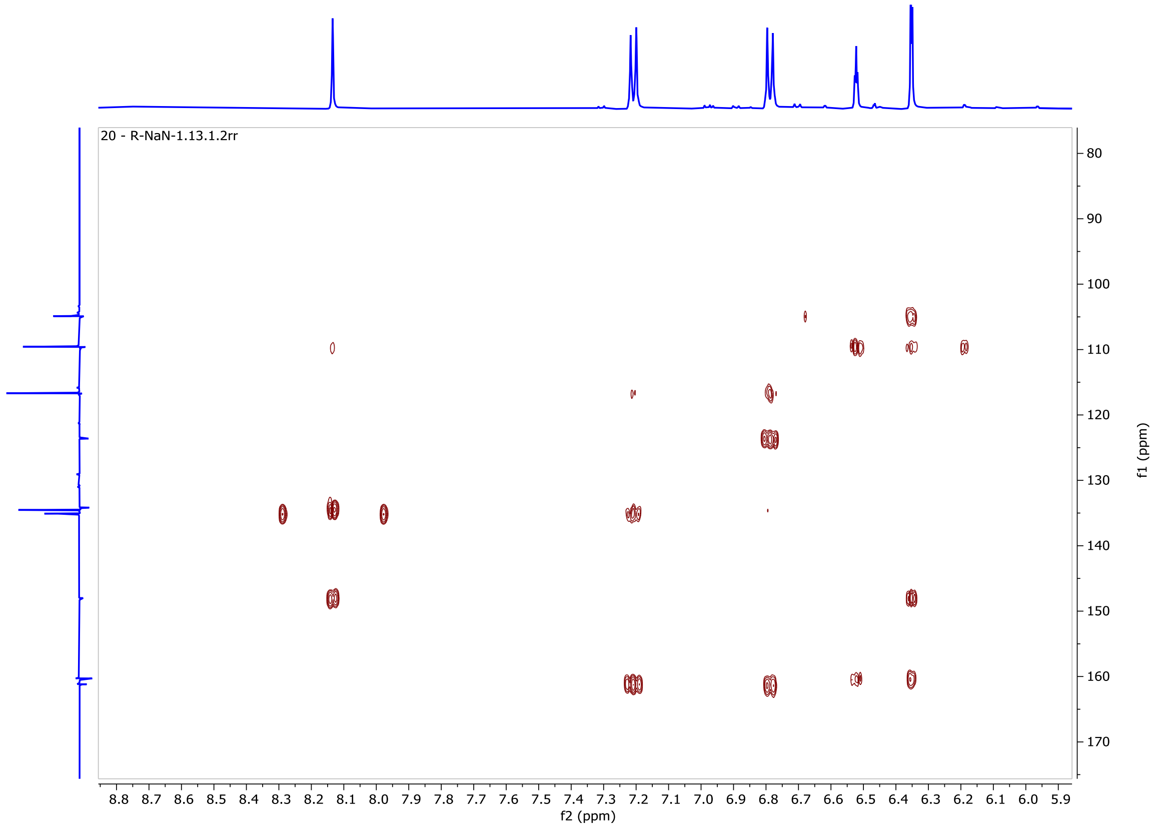

Figure S60. Compound **18**, COSY and NOESY spectra.

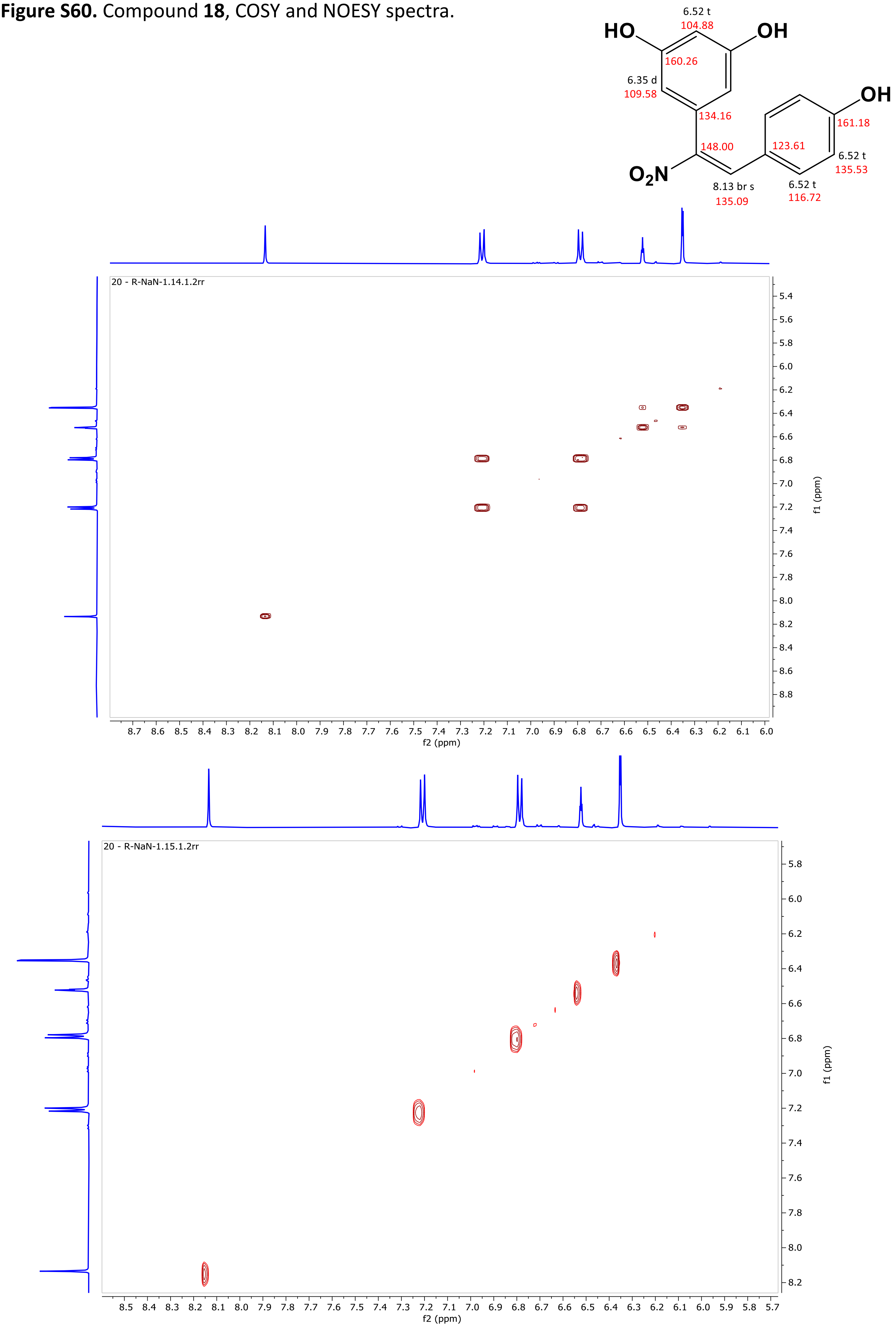

Figure S61. Compound **19**, ¹H NMR and ¹³C, APT NMR spectra.

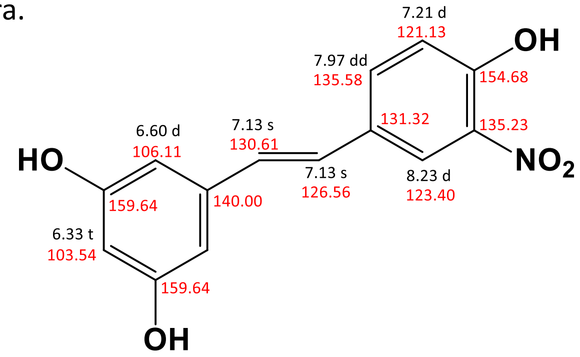

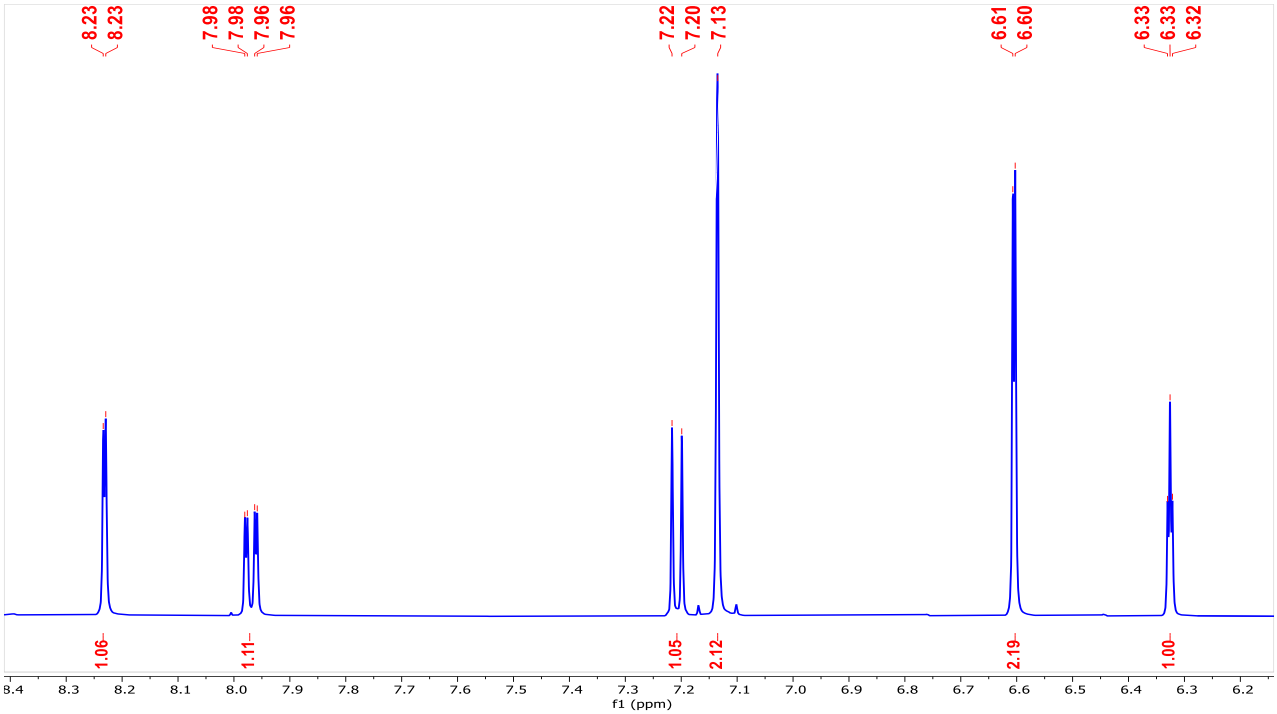

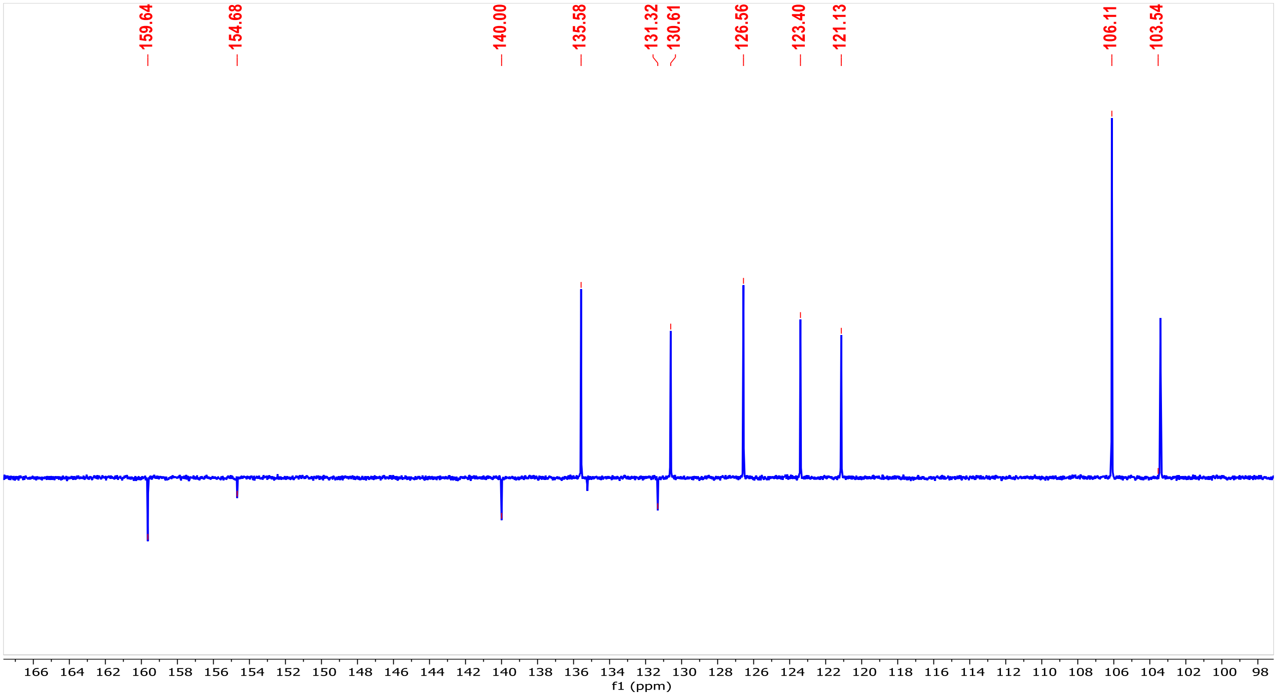

Figure S62. Compound **19**, HSQC and HMBC spectra.

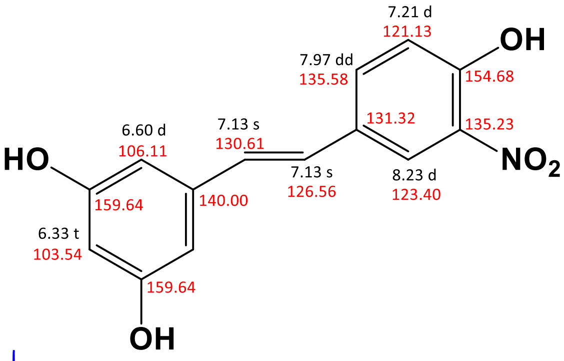

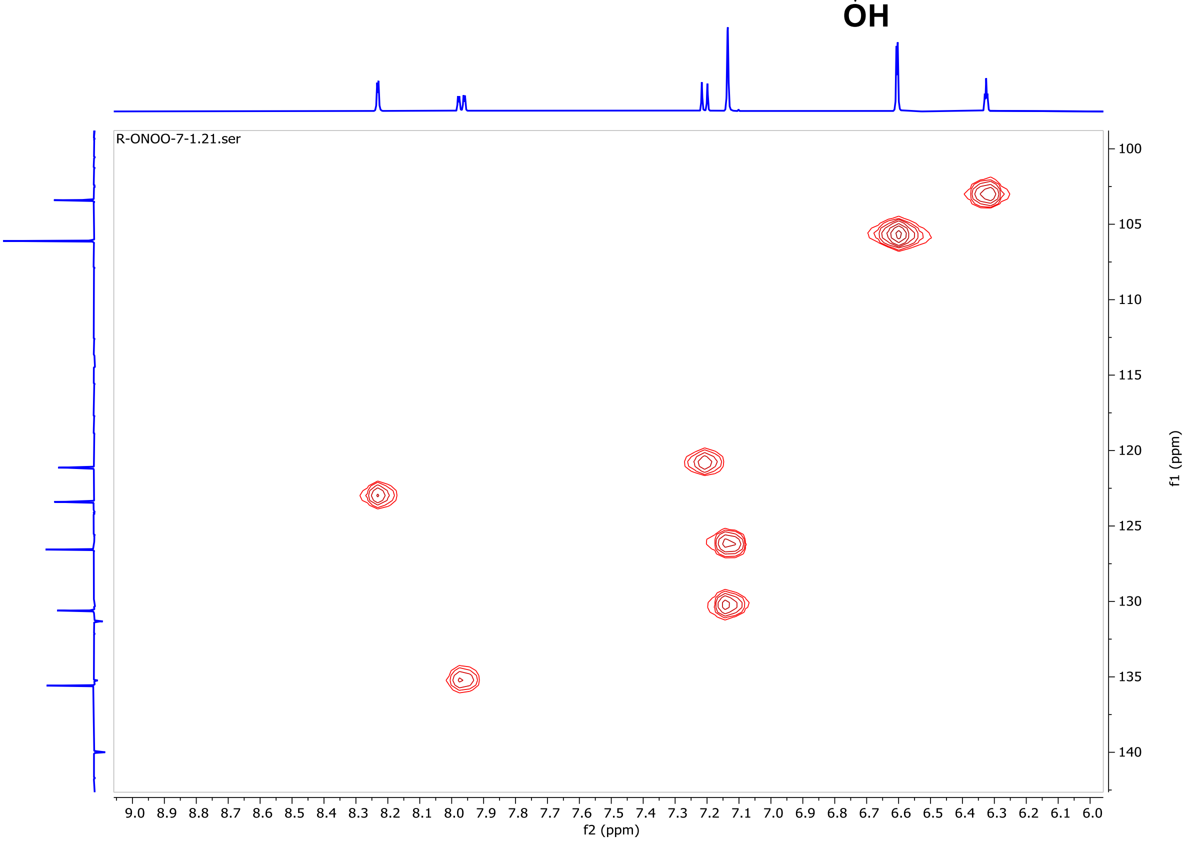

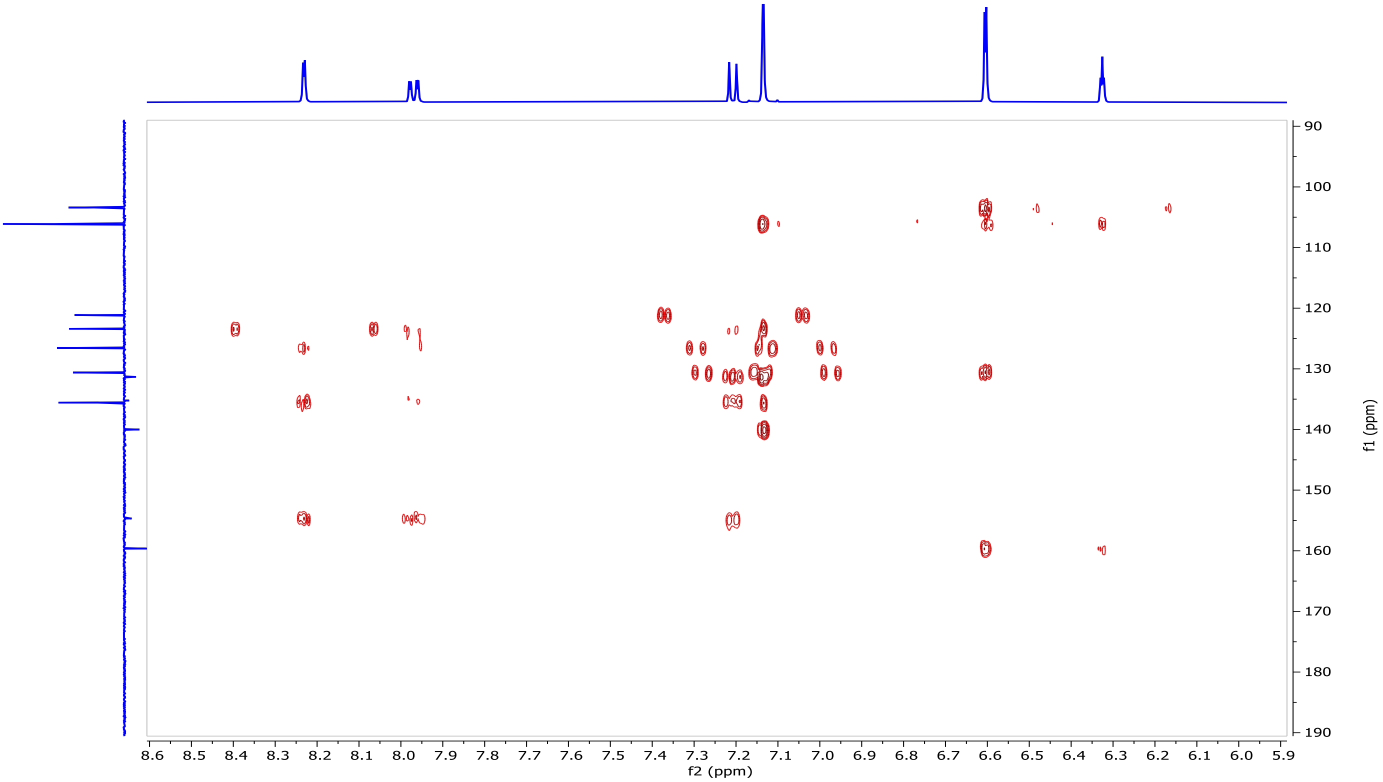

Figure S63. Compound **19**, COSY and NOESY spectra.

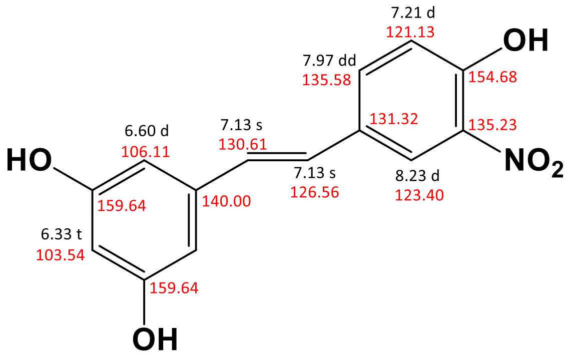

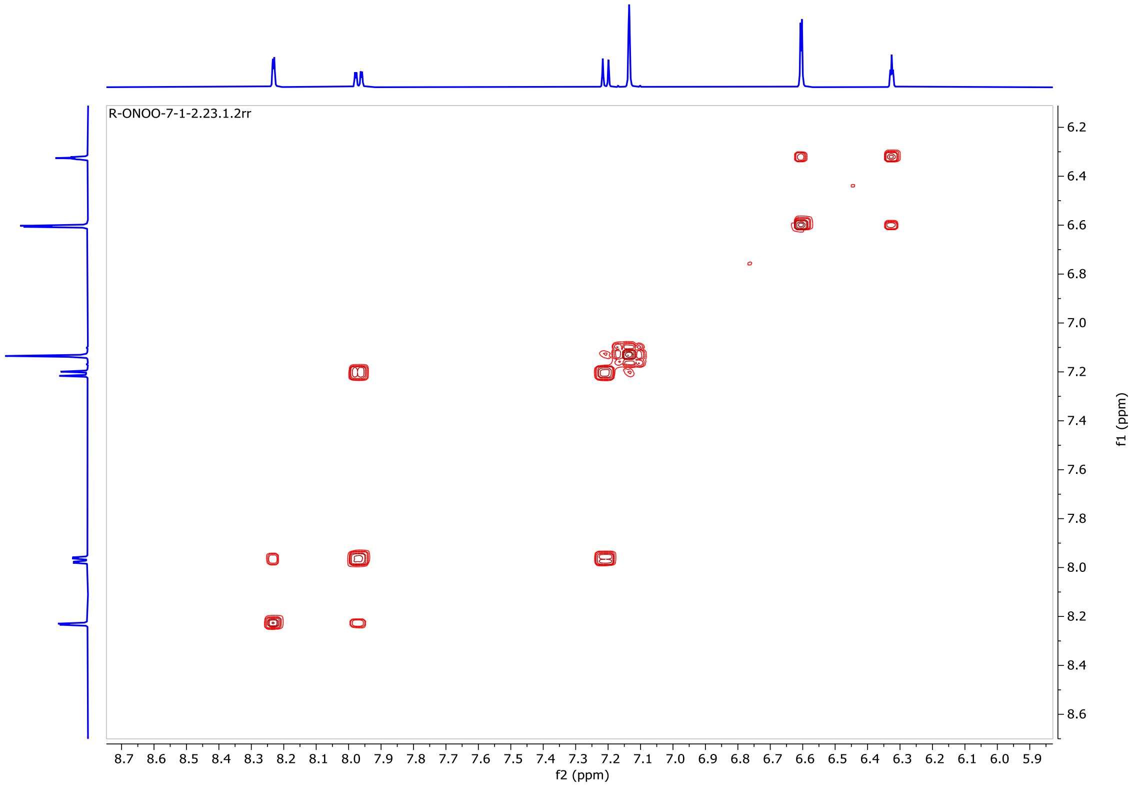

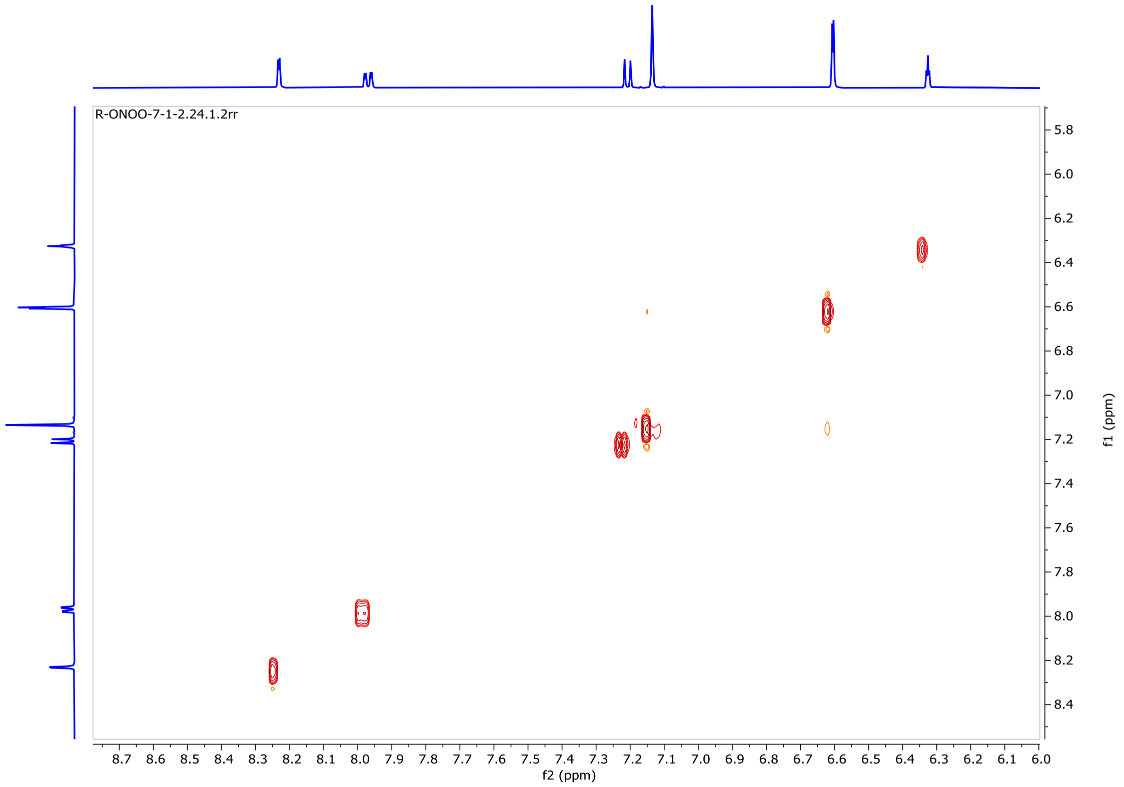

Figure S64. Lineweaver-Burk Plots of compound **6**

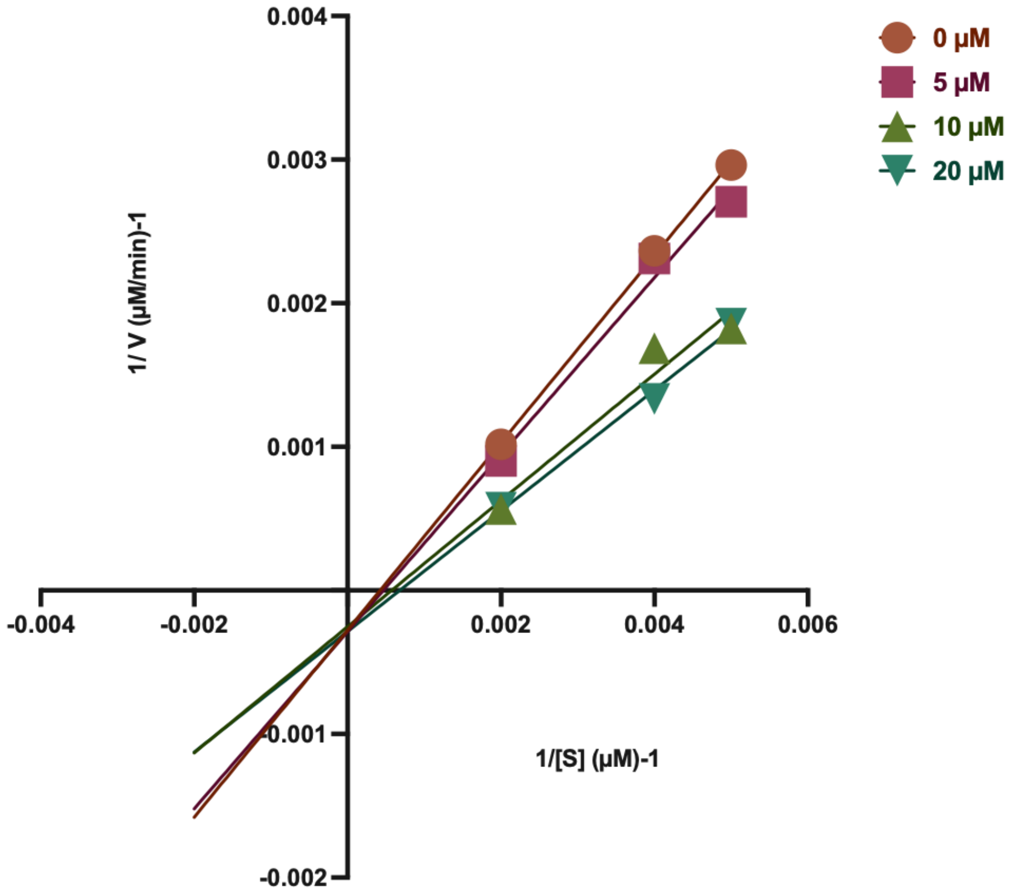

Concentration (µM)	Vmax (µM/min)	Km (µM)	Inhibition type
0	-3631.1	-2367.1	Competitive inhibition
5	-3481.9	-2146.6	
10	-3965.1	-1742.7	
20	-3505.0	-1473.2	

Figure S65. Lineweaver-Burk Plot of compound **12**

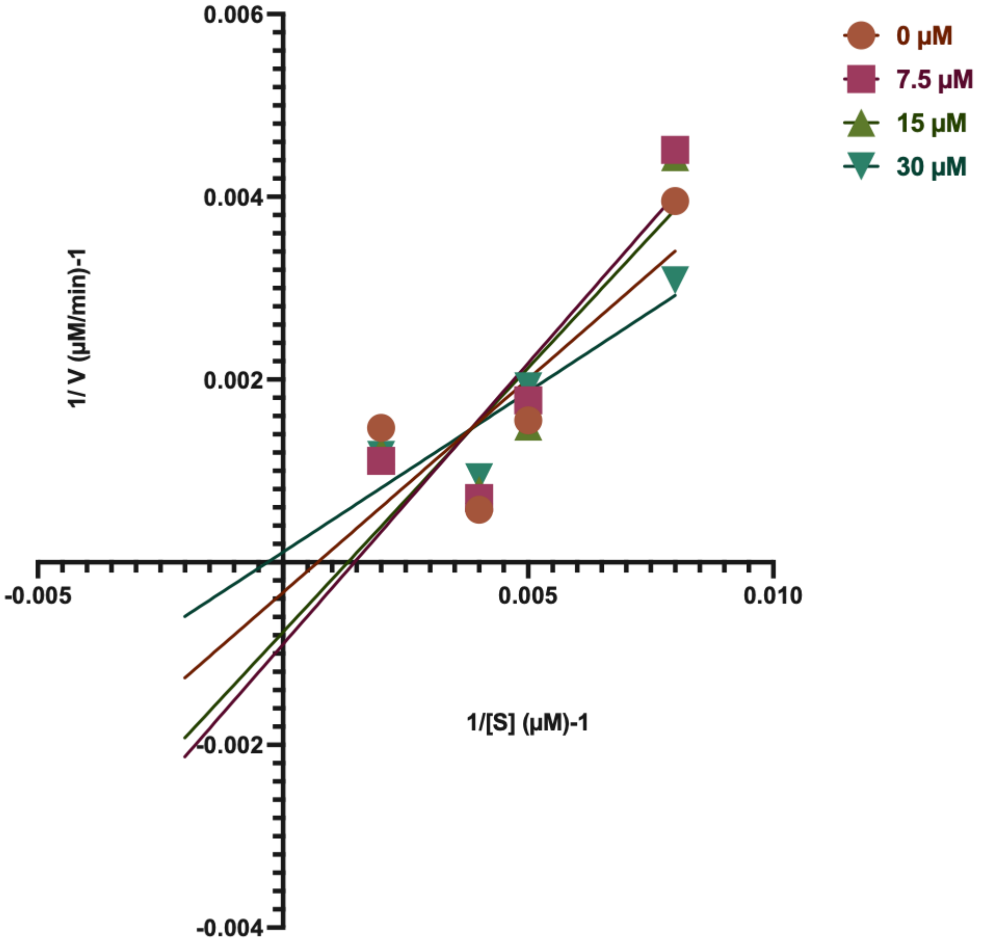

Concentration (µM)	Vmax (µM/min)	Km (µM)	Inhibition type
0	-3019.3	-1410.3	Mixed inhibition
7.5	-1112.8	-685.0	
15	-1306.8	-755.7	
30	-9107.7	-3199.4	

Figure S66. Chiral separation of **6** showing successive injections. Each 10 µL injection contained 20 µg sample

HPLC column: Chiralcel OD-H (250 x 4.6 mm, 5 µm)
Mobile phase system: Hex –EtOH, 82-18

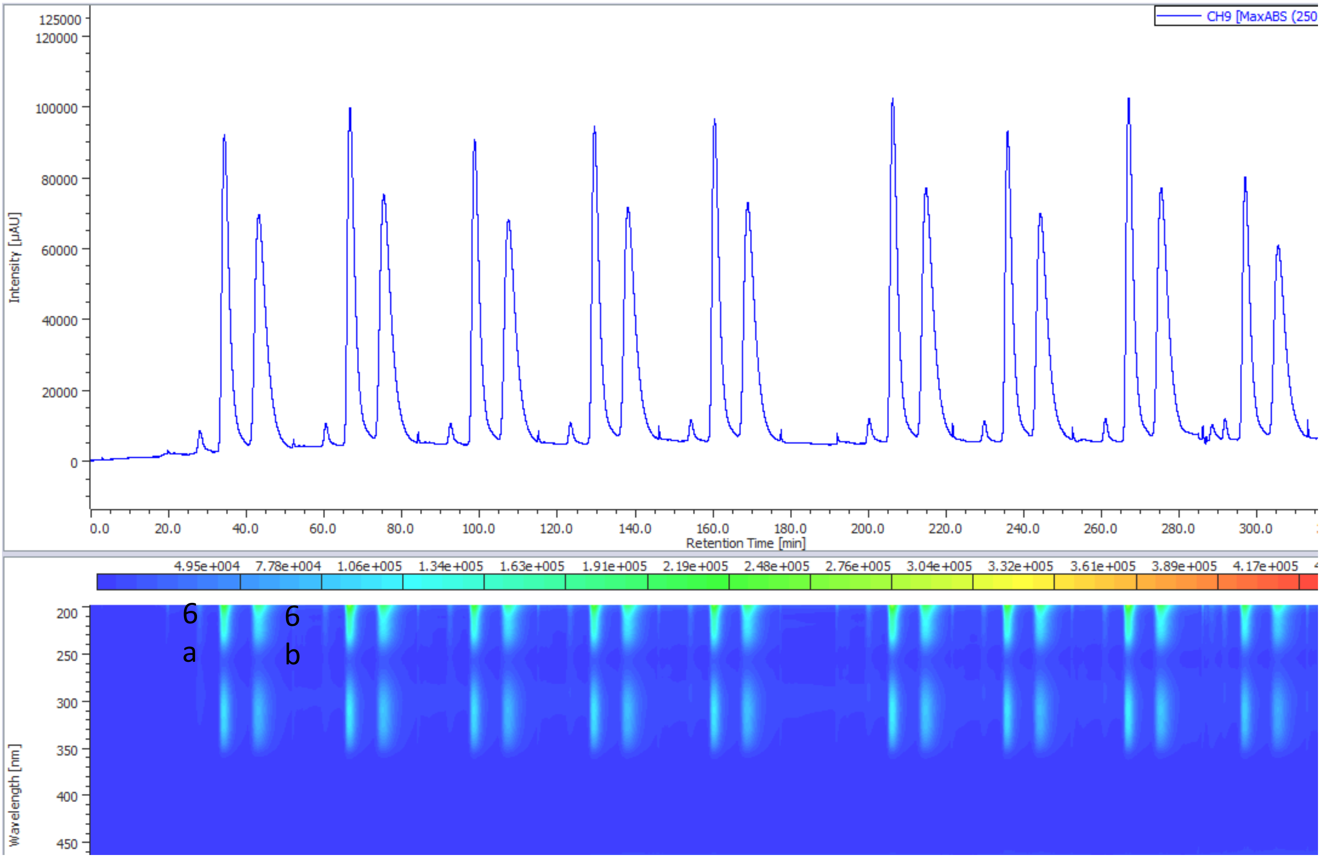

Figure S67. Structure of the lowest-energy conformer of the (2S, 3S) enantiomer of compound **6**

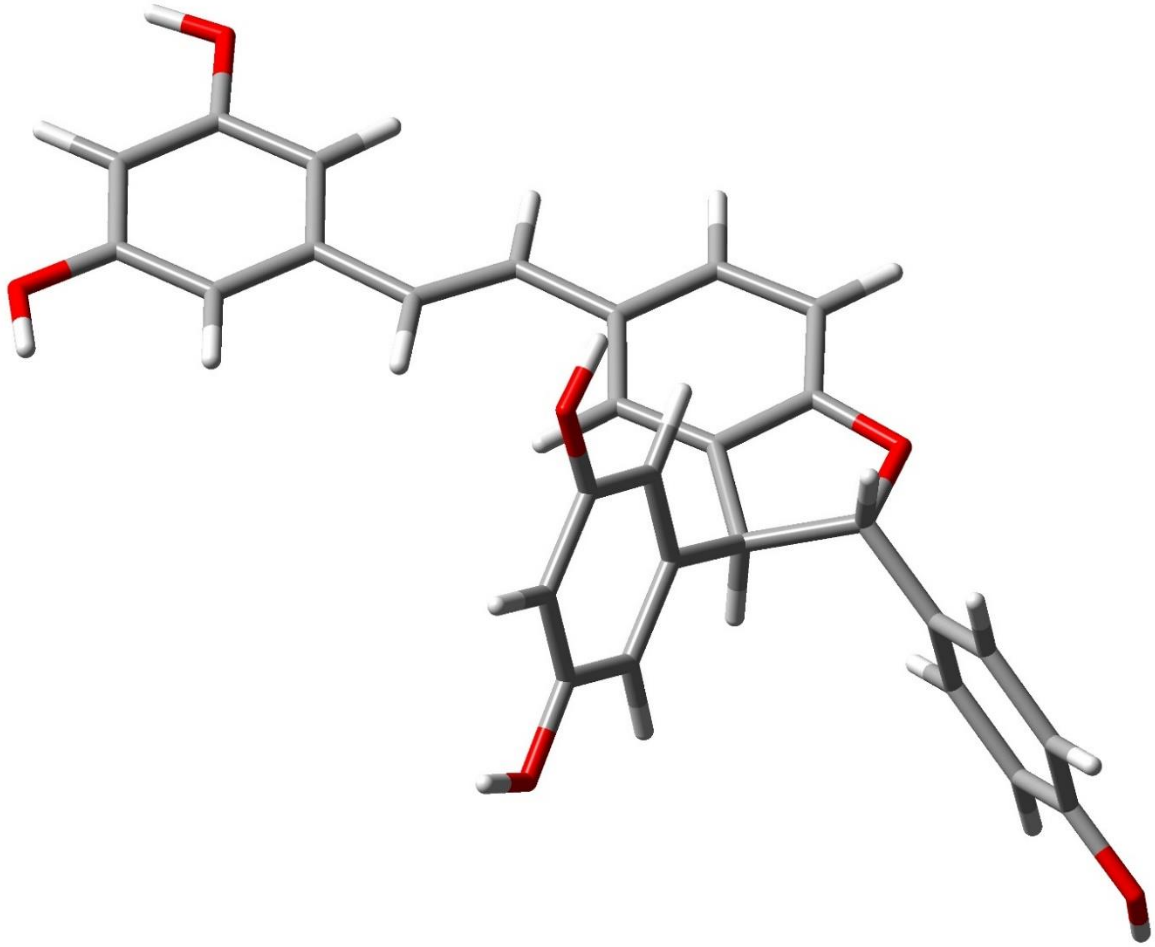

Table S1. Binding energies of 20 ga runs on cACE (PDB: 1O86) by compound **6a-(*R,R*)** (above) and **6b-(S,S)** (below).

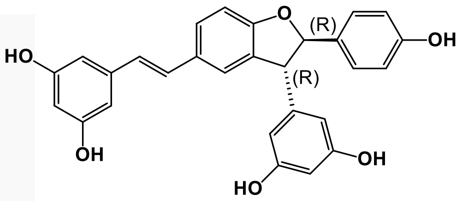

RMSD TABLE

Rank	Sub-Rank	Run	Binding Energy	Cluster RMSD	Reference RMSD	Grep Pattern
1	1	15	-10.38	0.00	64.31	RANKING
1	2	2	-10.26	1.47	65.21	RANKING
1	3	19	-10.19	0.27	64.27	RANKING
1	4	20	-10.19	0.22	64.34	RANKING
1	5	12	-9.99	0.43	64.45	RANKING
1	6	3	-9.59	1.33	64.96	RANKING
1	7	18	-9.21	1.95	65.96	RANKING
1	8	17	-9.15	1.58	65.13	RANKING
1	9	9	-9.15	1.78	65.64	RANKING
1	10	8	-9.07	1.57	65.09	RANKING
1	11	5	-9.01	1.68	65.26	RANKING
2	1	6	-9.68	0.00	68.54	RANKING
3	1	14	-9.27	0.00	65.88	RANKING
4	1	1	-8.91	0.00	68.25	RANKING
4	2	16	-8.82	0.32	68.26	RANKING
4	3	10	-8.81	0.21	68.27	RANKING
4	4	11	-8.60	0.90	68.31	RANKING
4	5	7	-8.57	0.86	68.26	RANKING
4	6	13	-8.51	0.89	68.30	RANKING
5	1	4	-8.72	0.00	65.50	RANKING

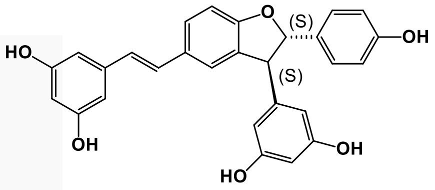

RMSD TABLE

Rank	Sub-Rank	Run	Binding Energy	Cluster RMSD	Reference RMSD	Grep Pattern
1	1	16	-10.02	0.00	41.72	RANKING
1	2	4	-9.82	0.72	41.77	RANKING
1	3	2	-9.67	0.81	41.81	RANKING
1	4	3	-9.37	0.76	41.80	RANKING
1	5	18	-8.73	1.12	41.43	RANKING
1	6	7	-8.52	1.30	41.57	RANKING
2	1	19	-9.42	0.00	44.50	RANKING
2	2	17	-8.97	1.87	43.79	RANKING
2	3	9	-8.96	0.38	44.46	RANKING
3	1	10	-9.28	0.00	43.52	RANKING
4	1	5	-9.24	0.00	44.63	RANKING
5	1	8	-9.10	0.00	41.60	RANKING
5	2	13	-9.07	1.04	41.74	RANKING
5	3	20	-9.04	0.20	41.50	RANKING
5	4	6	-8.93	1.01	41.71	RANKING
6	1	12	-8.92	0.00	43.81	RANKING
6	2	1	-8.64	1.04	43.70	RANKING
7	1	14	-8.90	0.00	44.34	RANKING
7	2	11	-8.78	0.29	44.28	RANKING
8	1	15	-8.87	0.00	44.32	RANKING

Table S2. Binding energies of 20 ga runs on nACE (PDB: 2C6N) by compound **6a-(*R*,*R*)** (above) and **6b-(S,S)** (below).

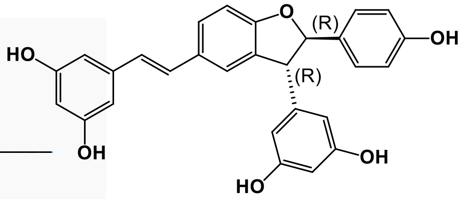

RMSD TABLE

Rank	Sub-Rank	Run	Binding Energy	Cluster RMSD	Reference RMSD	Grep Pattern
1	1	16	-9.99	0.00	72.73	RANKING
2	1	3	-9.83	0.00	64.97	RANKING
2	2	15	-9.72	0.25	65.12	RANKING
2	3	20	-9.65	0.19	65.03	RANKING
2	4	8	-9.59	0.81	65.14	RANKING
2	5	18	-9.44	0.84	64.96	RANKING
2	6	2	-9.35	0.23	64.93	RANKING
2	7	9	-9.06	1.21	64.65	RANKING
2	8	1	-9.03	1.59	64.08	RANKING
2	9	6	-9.01	1.16	64.50	RANKING
3	1	7	-9.38	0.00	63.79	RANKING
3	2	11	-9.33	0.20	63.82	RANKING
4	1	13	-9.13	0.00	63.72	RANKING
4	2	10	-8.12	1.96	63.15	RANKING
5	1	17	-8.66	0.00	71.67	RANKING
5	2	19	-8.65	1.91	70.76	RANKING
5	3	12	-8.63	1.84	70.62	RANKING
5	4	14	-8.57	1.60	71.23	RANKING
5	5	4	-8.51	1.92	70.69	RANKING
5	6	5	-8.49	0.53	71.66	RANKING

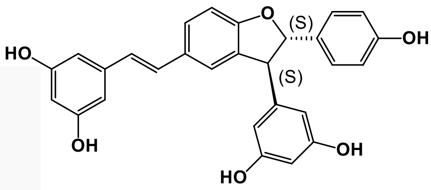

RMSD TABLE

Rank	Sub-Rank	Run	Binding Energy	Cluster RMSD	Reference RMSD	Grep Pattern
1	1	1	-10.07	0.00	48.21	RANKING
1	2	5	-10.06	0.52	48.39	RANKING
1	3	7	-10.03	0.47	48.32	RANKING
1	4	20	-9.83	1.27	48.17	RANKING
1	5	16	-9.74	0.99	48.03	RANKING
1	6	6	-9.68	0.80	48.42	RANKING
1	7	4	-9.67	0.87	47.90	RANKING
1	8	17	-9.65	1.32	48.15	RANKING
1	9	2	-9.60	1.18	48.14	RANKING
1	10	14	-9.59	1.77	48.75	RANKING
1	11	15	-9.56	1.23	48.17	RANKING
1	12	9	-9.53	1.04	47.94	RANKING
2	1	3	-9.65	0.00	48.73	RANKING
2	2	19	-9.61	0.34	48.67	RANKING
2	3	11	-9.59	0.09	48.72	RANKING
2	4	10	-9.55	0.18	48.75	RANKING
2	5	8	-9.51	0.81	48.91	RANKING
2	6	13	-9.51	1.06	48.96	RANKING
3	1	18	-8.91	0.00	45.29	RANKING
4	1	12	-8.56	0.00	47.37	RANKING

Figure S68. HPLC chromatogram of compound **6** at UV absorbance λ = 320 nm

Kinetex XB-C18 (250 x 4.6 mm, 5 µm), Elution: water (solvent A) and CH_3CN (solvent B): elution, linear gradient from 25% solvent B to 75% solvent B for 25 min and then isocratic mode for 75% solvent B for 2 min; flow rate, 1 mL/min;

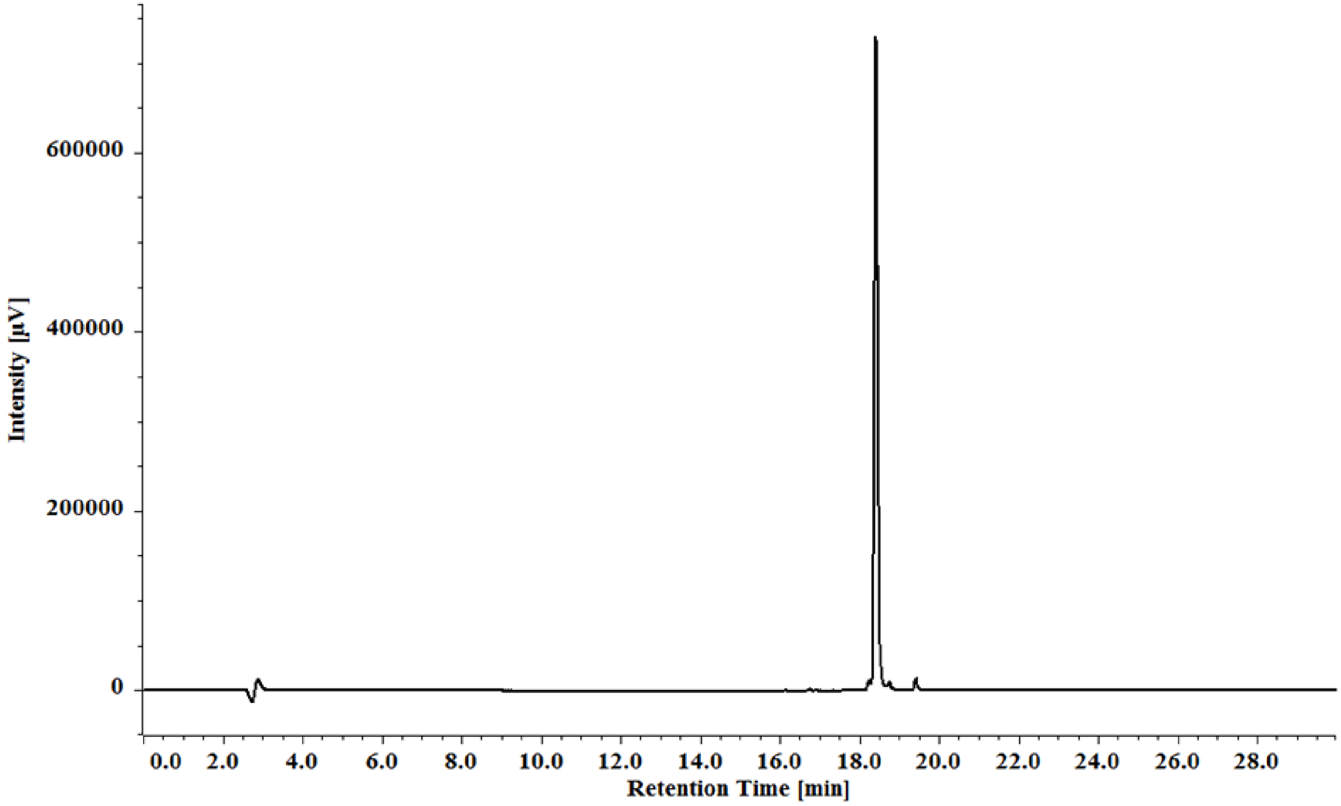

Purity 96.9%

Figure S69. HPLC chromatogram of compounds **6a**-(*R*,*R*) and **6b**-(*S*,*S*) at UV absorbance λ = 320 nm

Chiralcel OD-H (250 x 4.6 mm, 5 µm), Elution: Hexane –EtOH, 82-18%

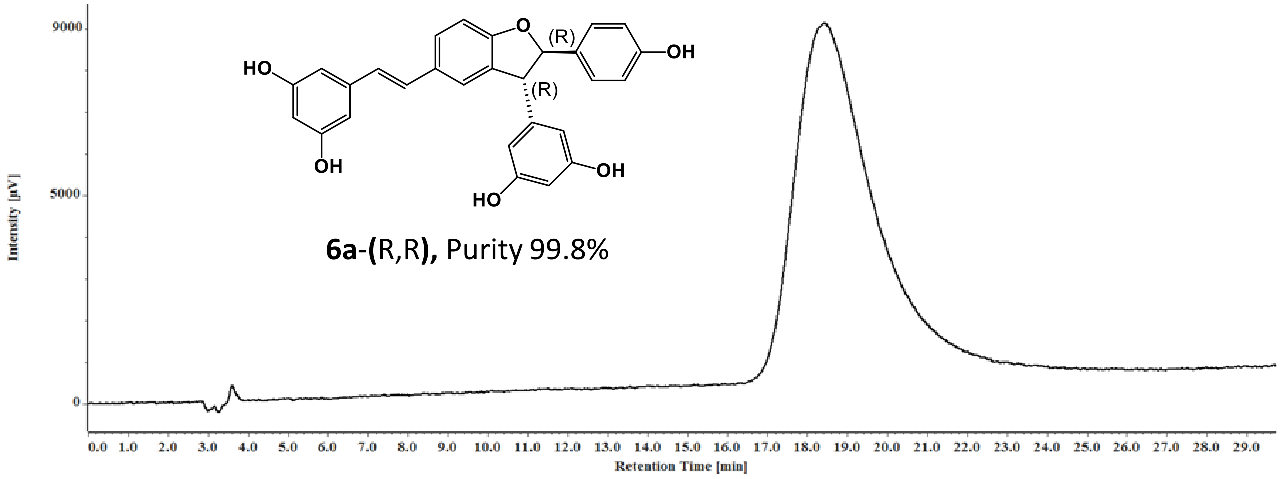

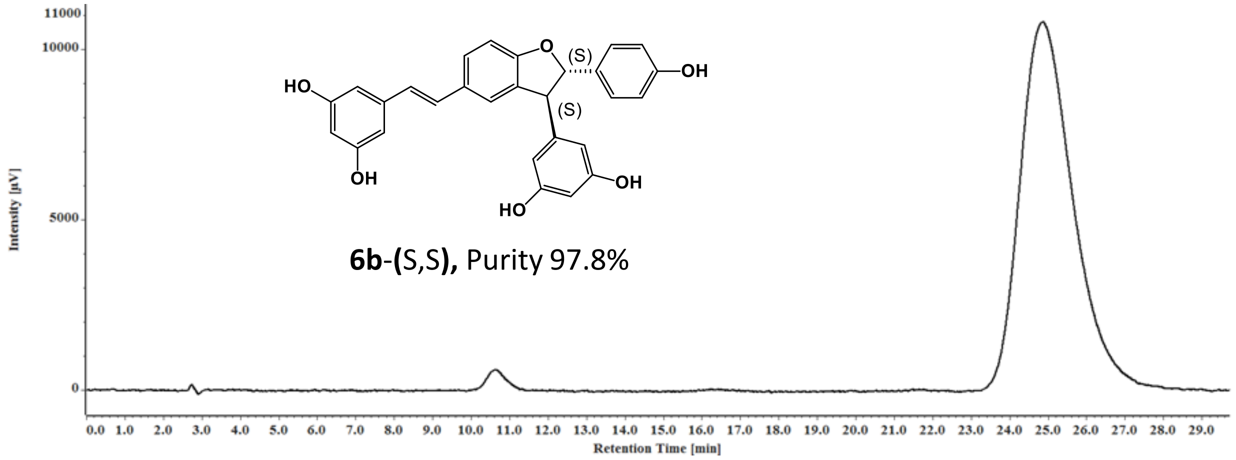

Figure S70. HPLC chromatogram of compounds **2** and **12** at UV absorbance λ = 320 nm

HPLC column: Kinetex XB-C18 (250 x 4.6 mm, 5 μm)
Mobile phase system: water (solvent A) and CH_3CN (solvent B): elution, linear gradient from 25% solvent B to 75% solvent B for 25 min and then isocratic mode for 75% solvent B for 2 min; flow rate, 1 mL/min;

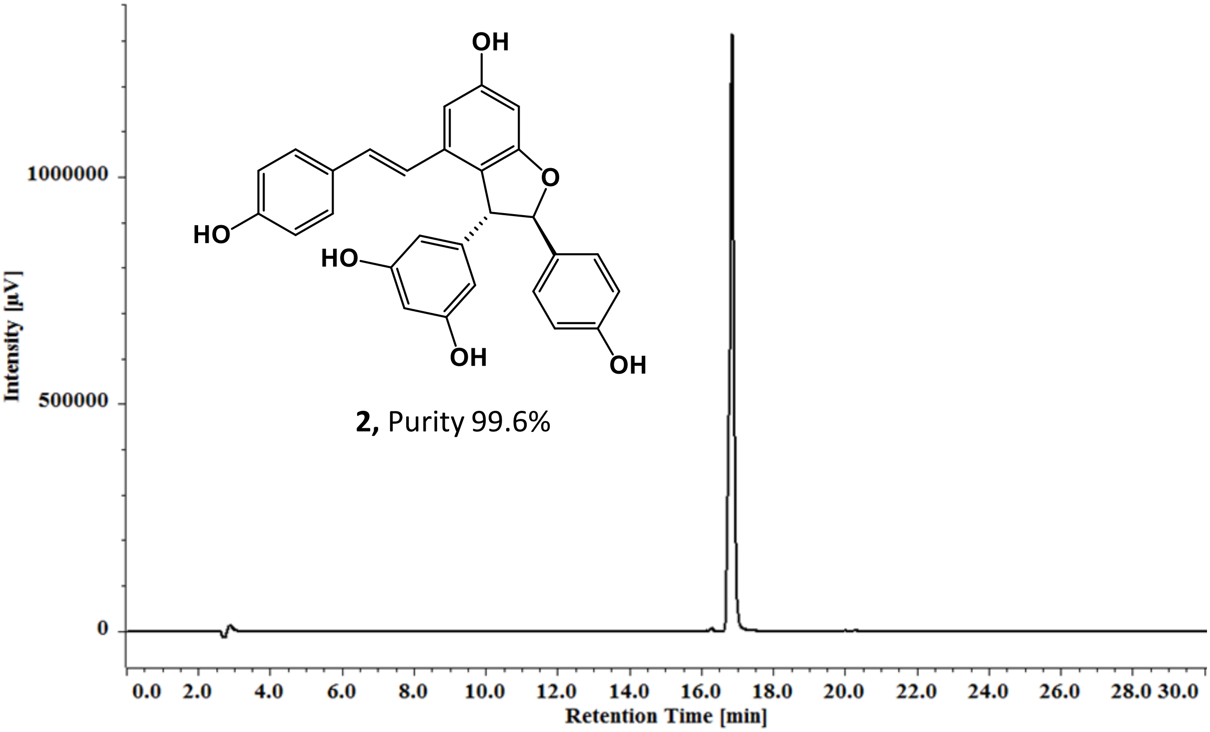

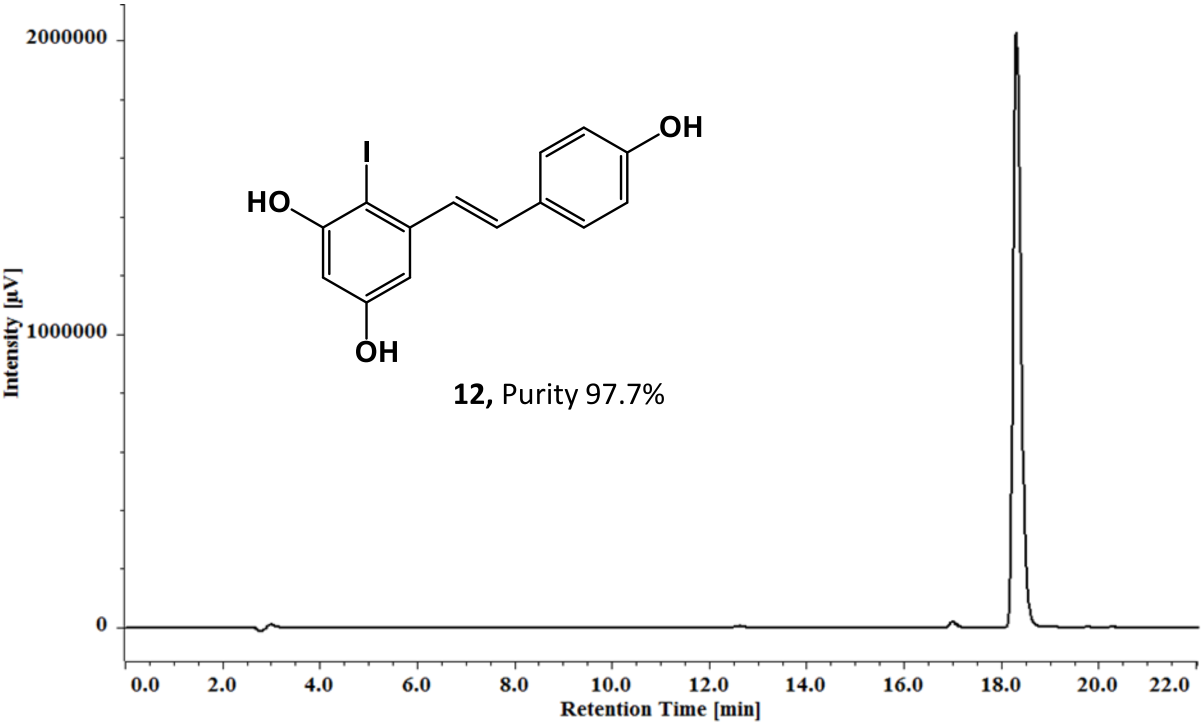

Supplement: Supplementary file 1 [file jm4c03061_si_001.pdf]
